# Cystatin A promotes the antitumor activity of T helper type 1 cells and dendritic cells in murine models of pancreatic cancer

**DOI:** 10.1002/1878-0261.13796

**Published:** 2025-01-10

**Authors:** Alessandro Nasti, Shingo Inagaki, Tuyen Thuy Bich Ho, Akihiro Seki, Keiko Yoshida, Kosuke Satomura, Yoshio Sakai, Shuichi Kaneko, Taro Yamashita

**Affiliations:** ^1^ Information‐Based Medicine Development Graduate School of Medical Sciences, Kanazawa University Japan; ^2^ System Biology, Graduate School of Advanced Preventive Medical Sciences Kanazawa University Japan; ^3^ Department of Gastroenterology Kanazawa University Hospital Japan

**Keywords:** antitumor, CD4+ T cells, CSTA, cystatin A, DCs, dendritic cells, PDAC, recruitment, Th1 response

## Abstract

Pancreatic ductal adenocarcinoma (PDAC) is a disease with poor prognosis due to diagnostic and therapeutic limitations. We previously identified cystatin A (CSTA) as a PDAC biomarker and have conducted the present study to investigate the antitumor effects of CSTA. PDAC murine models were established with genetically modified PAN02 tumor cell lines to evaluate the antitumor immune response. PDAC mouse survival was significantly longer with CSTA, and its antitumor effect was mediated mainly by CD4+ cells and partly by CD8+ cells. We also observed an increased infiltration of CD4+ and CD8+ cells in tumors of mice overexpressing *CSTA*. Phenotypically, we confirmed higher T helper type 1 (Th1) cell activity and increased frequency and activity of M1 macrophages and dendritic cells (DCs) in *CSTA*‐overexpressing mice. Gene expression analysis highlighted pathways related to interferon gamma (IFN‐γ) induction and Th1 lymphocyte activation that were induced by CSTA. Macrophages and DCs shifted toward proinflammatory antitumor phenotypes. Furthermore, activated splenocytes of PDAC model mice expressing *CSTA* had increased proapoptotic activity. CSTA also promoted the selective migration of CD4+ and CD11c+ immune cells in an *in vitro* migration assay. In conclusion, CSTA exerts antitumor effects by enhancing Th1‐mediated antitumor effects through promotion of DC and M1 macrophage activity, thereby increasing immune cell chemotaxis. CSTA could be a novel therapeutic candidate for PDAC.

## Graphical abstract

Pancreatic ductal adenocarcinoma (PDAC) is a disease with very poor prognosis due to therapeutic limitations. We investigated the antitumor effects of cystatin A (CSTA) in PDAC murine models. We are first to confirm that CSTA enhances T helper type 1‐mediated antitumor effects through promotion of dendritic cells and M1 macrophage activity. CSTA can be a novel therapeutic candidate for PDAC.

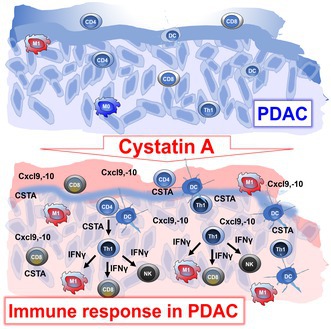

**Abbreviations**
Ab: antibody
CSTA: cystatin A
DAB: diaminobenzidine
DMEM: Dulbecco's modified Eagle's medium
FBS: fetal bovine serum
FCM: flow cytometry
IP: intraperitoneal
NK: natural killer
PBS: phosphate‐buffered saline
PDAC: pancreatic ductal adenocarcinoma
qRT‐PCR: quantitative real‐time PCR
SC: subcutaneous
SPCs: splenocytes
TICs: tumor‐infiltrating inflammatory cells
WT: wild‐type

## 1 Introduction

Pancreatic ductal adenocarcinoma (PDAC) is a fearsome disease with a very poor prognosis [1]. Most PDAC cases are discovered during the final stages of the disease [2] due to the initial lack of apparent symptoms and to inefficient assessment methods. Consequently, metastases develop aggressively in most patients [3], and there is typically no possibility of surgical treatment. Chemotherapy is the only possible option for these patients, but its efficacy is limited. Therefore, new treatments for PDAC patients are urgently needed [4, 5].

We previously confirmed that PDAC patients had increased expression of the cysteine protease inhibitor cystatin A (CSTA) in CD4+ T cells of the peripheral blood [6]. Furthermore, CSTA concentrations in blood serum are higher in PDAC patients than in healthy patients [6, 7]. High levels of CSTA have been correlated with tumor progression [8, 9, 10] and, in this regard, CSTA suppresses tumor invasion in various types of cancer [11, 12, 13, 14, 15, 16]. However, there is no clear evidence of the antitumor effects of CSTA in the context of PDAC.

We previously confirmed a role for CSTA as a biomarker [6], but we wanted to further investigate how CSTA would impact PDAC by analyzing both systemic and local immune responses and to determine if CSTA could be used as a novel immunostimulant treatment for PDAC. In the present study, we produced murine PDAC tumor cell lines able to continuously overexpress *CSTA* or to express *CSTA* upon selective induction. We used these cell lines to establish mouse PDAC models to examine the effect of CSTA on tumor growth and the immune response. We observed that CSTA induced a major anticancer effect and prolonged survival. This effect was due to CSTA‐mediated enhancement of the antitumor activity of Th1, together with the proinflammatory activity of M1 macrophages and dendritic cells (DCs). Furthermore, we also confirmed *in vitro* that CSTA indirectly induces cell‐selective chemotaxis. We concluded that CSTA could be a candidate for the immunotherapy of PDAC.

## 2 Materials and methods

### 2.1 Cell line establishment and culture

The PDAC PAN02 cell line (RRID: CVCL_D627; DCTD Tumor Repository, NCI‐Frederick, Frederick, MD, USA) was used to establish PAN02‐CSTA, PAN02‐CSTA‐GFP, PAN02‐GFP, Retro‐PAN02‐CSTA, and Retro‐PAN02‐CSTA‐GFP cell lines. Briefly, for the generation of PAN02‐CSTA, PAN02‐CSTA‐GFP, and PAN02‐GFP cell lines, a vector system with a *Csta1* mouse untagged clone (NM_001033239; Cat. No.: MC212520, OriGene Technologies, Inc., Rockville, MD, USA), *Csta1* mouse tGFP‐tagged ORF clone (NM_001033239; Cat. No.: MG220055, TrueORF®, OriGene Technologies, Inc.), or control pCMV6‐AC‐GFP vector (Cat. No.: PS100010; OriGene Technologies, Inc.) were used for overexpression experiments. The PAN02 cells were transfected with plasmids using Lipofectamine™ 2000 Transfection Reagent (Invitrogen, Carlsbad, CA, USA) according to the manufacturer's protocol. Because the vectors have geneticin resistance, the successfully transfected cells were positively selected by adding 1 mg·mL^−1^ of Geneticin™ Selective Antibiotic (G418 Sulfate; Gibco, Thermo Fisher Scientific Inc., Waltham, MA, USA) to Dulbecco's modified Eagle's medium (DMEM; Nacalai Tesque, Inc., Kyoto, Japan) supplemented with 10% heat‐inactivated fetal bovine serum (FBS; Gibco, Thermo Fisher Scientific Inc.). Flow cytometry (FCM) analysis was used to confirm the cellular expression of GFP‐tagged Csta1 and to measure the efficiency of the transfection. FCM was performed using a BD Accuri™ C6 flow cytometer (BD Biosciences, Franklin Lakes, NJ, USA).

Regarding Retro‐PAN02‐CSTA and Retro‐PAN02‐CSTA‐GFP generation, mouse *Csta1* or *GFP*‐tagged mouse *Csta1* was cloned into a pRetroX‐TetOne‐Puro vector (Cat. No.: 634307; Clontech Laboratories, Inc., Mountain View, CA, USA) using an In‐Fusion^®^ HD Cloning Kit (Cat. No.: 639648; Takara Bio Inc., Kusatsu, Japan) as per the protocol. To generate retroviruses, pRetroX‐TetOne‐Puro‐*Csta1* or pRetroX‐TetOne‐Puro‐*Csta1‐GFP* constructs were each cotransfected with an envelope vector into the GP2‐293 cell line (Cat. No.: 631458; Clontech Laboratories, Inc.) by using Xfect Transfection Reagent; the retroviral supernatant was harvested 48 h after transfection and the titer determined. The PAN02 cell line was then transduced with RetroX‐TetOne‐Puro‐*Csta1* or RetroX‐TetOne‐Puro‐*Csta1‐GFP* retrovirus as per the protocol, and clones were selected by using Puromycin selection. Finally, Retro‐PAN02‐CSTA and Retro‐PAN02‐CSTA‐GFP lines were established, which express *CSTA* or *CSTA‐GFP* upon doxycycline induction.

Subsequently, all cell lines were cultured in DMEM supplemented with 10% FBS and 100 μg·mL^−1^ penicillin and streptomycin (Life Technologies, Carlsbad, CA, USA) in a 5% CO_2_ incubator at 37 °C. The PAN02, PAN02‐CSTA, PAN02‐CSTA‐GFP, PAN02‐GFP, Retro‐PAN02‐CSTA, and Retro‐PAN02‐CSTA‐GFP cell lines were authenticated within the past 3 years by using the FTA Sample Collection Kit for Mouse Cell Authentication Service (137‐XV™, ATCC, Gaithersburg, MD, USA); the authentication service uses mouse short tandem repeat profiling. All cell lines were confirmed to be mycoplasma‐free using a TaKaRa PCR Mycoplasma Detection Set (Cat. No.: 6601; Takara Bio Inc.).

### 2.2 DNA microarray analysis of PAN02 and PAN02‐CSTA cell lines

RNA was isolated from PAN02 and PAN02‐CSTA cells using an ISOSPIN Cell & Tissue RNA Kit (Nippon Gene, Tokyo, Japan). The DNA microarray method was performed as previously described [17, 18]. BRB‐ArrayTools [19, 20] v.4.6.2 – Beta_1 was used to analyze gene expression: quantile normalization was performed, spots were excluded if the intensity was below 1, probes were removed if data from a spot were missing, and multiple probes were reduced to one gene per symbol by choosing the maximally expressed probe determined by average intensity. The number of genes passing the filtering criteria was 16 846. By comparing PAN02‐CSTA and PAN02 cell lines, we identified 2454 differentially expressed genes: 1204 genes were upregulated while 1250 genes were downregulated in PAN02‐CSTA compared with PAN02. These gene sets were used in DAVID enrichment analyses (Tables S2 and S3; *P* < 0.05; david software; https://david.ncifcrf.gov/, accessed in November 2023) [21, 22].

### 2.3 Establishment of murine PDAC models of PDAC and treatments

The Institutional Review Board of Kanazawa University approved the procedures for the care and use of laboratory animals in this study (approval numbers: AP‐173850 and AP‐224359). Mice were housed in clean, well‐ventilated cages, in rooms equipped with filters to maintain air quality and prevent contamination. Each cage was provided with appropriate bedding material and changed regularly to maintain hygiene. Experiments were conducted only at the locations specified in the experimental plans. Highly invasive surgeries, and postoperative observation and management, were performed only by experimenters provided with animal handling knowledge and experience. In high‐distress experiments, euthanasia was performed in line with humanitarian endpoints. Eight‐week‐old female C57BL/6J mice or female athymic nude BALB/c‐nu mice (The Jackson Laboratories Japan, Yokohama, Japan) were anesthetized before the establishment of PDAC models. The mice were intraperitoneally administered a mixture of 4 mg·kg^−1^ midazolam (10 mg/2 mL Dormicum^®^; Astellas Pharma, Inc., Tokyo, Japan), 0.3 mg·kg^−1^ medetomidine (1 mg·mL^−1^ Domitor^®^; Nippon Zenyaku Kogyo, Koriyama, Japan), and 5 mg·kg^−1^ butorphanol (5 mg·mL^−1^ Vetorphale^®^; Meiji Seika Pharma, Tokyo, Japan).

For the establishment of intraperitoneal (IP) PDAC models, mice were injected intraperitoneally with 1 × 10^6^ PAN02 or PAN02‐CSTA‐GFP cells suspended in 200 μL phosphate‐buffered saline (PBS; FUJIFILM Wako Pure Chemical Industries, Osaka, Japan) as previously described [23]. The IP PDAC models were used for survival tests and, in one of the experiments, the PAN02‐CSTA‐GFP IP PDAC model received the following antibody (Ab) treatments (200 μg of Ab in 200 μL of PBS) by IP injection: no treatment (only PBS), Rat IgG2a, κ (isotype control), Rat IgG2b, κ (isotype control), anti‐CD4 Ab ([GK1.5] rat IgG2b, κ), anti‐CD8a Ab ([53–6.7] rat IgG2a, κ), and anti‐Ly‐6G/Ly‐6C Ab ([RB6‐8C5] rat IgG2b, κ) (Table S1 for antibody details). For the establishment of subcutaneous (SC) PDAC models, mice were injected subcutaneously into the right leg with either Retro‐PAN02‐CSTA or Retro‐PAN02‐CSTA‐GFP cells suspended in 50 μL of PBS. The longest and shortest diameters of tumors were measured and volume calculated as previously reported [24]. Once the tumors had reached an average size of about 800 mm^3^, mice were given water supplied with 5% sucrose (FUJIFILM Wako Pure Chemical Industries) and 9 mg·mL^−1^ doxycycline hyclate (Sigma‐Aldrich, St. Louis, MO, USA) to induce the expression of *CSTA* in tumors.

### 2.4 Histological and immunohistochemical analyses

Immunohistochemistry was performed on OCT‐frozen tissue sections and paraffin‐embedded tissue sections. As previously described [25], OCT‐frozen sections were fixed with 4% formaldehyde and then incubated with primary Abs (Table S1). Next, the samples were incubated with a secondary Ab conjugated to horseradish peroxidase‐labeled dextran polymer (Histofine^®^ Simple Stain™ Mouse MAX PO, Rat, or Rabbit; Nichirei Corporation, Tokyo, Japan). Following the addition of diaminobenzidine substrate solution (DAB^®^; Dako ChemMate EnVision Kit/HRP; Dako, Kyoto, Japan), the sections were then stained with hematoxylin. ImageJ software was used to quantify the DAB‐positive area using the color deconvolution method [26].

### 2.5 DNA microarray analysis

Doxycycline was supplied to the water of Retro‐PAN02‐CSTA SC PDAC model mice from day 65 to day 84 for the induction of *CSTA*. Tumors were then sampled on day 84 and RNA was isolated by using an ISOSPIN Cell & Tissue RNA Kit (Nippon Gene, Tokyo, Japan). The DNA microarray method was performed as previously described [17, 18, 23]. BRB‐ArrayTools v. 4.6.0 – Beta_2 [19, 20] was used to analyze gene expression (http://linus.nci.nih.gov/BRB‐ArrayTools.html): quantile normalization was performed for each array, and spots were excluded if the intensity was below the minimum value of 1. If data from a specific spot were missing, the entire relative probe was removed across the arrays. Furthermore, multiple probes identifying the same gene were reduced to one per gene symbol by using the maximally expressed probe determined by average intensity across the arrays. The number of genes that passed the filtering criteria was 19667. Gene class comparison was performed to identify the 2455 differentially expressed genes among classes (with the nominal significance level of each univariate test set at 0.05). Out of the 2455 genes, the 1684 genes upregulated in tumor tissue by CSTA and 771 genes downregulated in tumor tissue by CSTA were used for enrichment analysis by pathway maps (*P* < 0.01; MetaCore, https://portal.genego.com/, version 21.2.70500; Clarivate Analytics, Philadelphia, PA, USA). BRB‐ArrayTools Geneset Class Comparison Analysis was performed to identify cell type‐related gene sets.

### 2.6 Quantitative real‐time PCR

RNA was isolated from tumors or from isolated [17] tumor‐infiltrating inflammatory cells (TICs) by using an ISOSPIN Cell & Tissue RNA Kit (Nippon Gene). We analyzed gene expression by quantitative real‐time PCR (qRT‐PCR) by using the QuantStudio 12 K Flex RealTime PCR system (Applied Biosystems, Foster City, CA, USA). For cDNA production, 100 ng RNA was reverse‐transcribed with the High‐Capacity cDNA Reverse Transcription Kit (Applied Biosystems) as previously described [27]. The cDNA was mixed with qPCR MasterMix Plus^®^ (Eurogentec, Seraing, Belgium) and PCR was performed with the following hydrolyzed TaqMan^®^ Gene Expression Assay probes (Thermo Fisher Scientific Inc.): *Ifng* (Mm01168134_m1), *Stat1* (Mm01257286_m1), *Pdcd1* (Mm01285676_m1), *Ccr5* (Mm01216171_m1), *Cxcl10* (Mm00445235_m1), *Ccl2* (Mm00441242_m1), *Ccl5* (Mm01302427_m1), *Tnf* (Mm00443258_m1), *Il1b* (Mm00434228_m1), *Il6* (Mm00446190_m1), *Il12a* (Mm00434169_ml), *Il12b* (Mm01288989_ml), *Arg1* (Mm00475988_m1), *Tgfb1* (Mm01178820_m1), *Mmp9* (Mm00442991_m1), *Il2ra* (Mm01340213_m1), *Cd69* (Mm01183378_m1), *Pdcd1* (Mm01285676_m1), *Lag3* (Mm00493071_m1), *Havcr2* (Mm01294183_m1), and *Tigit* (Mm03807522_m1). Mouse *GAPD* (*GAPDH*; Mm99999915_g1) Endogenous Control (VIC™/MGB probe, primer limited; Thermo Fisher Scientific Inc.) was used as a reference gene for relative expression levels determined by using the 2^−∆∆Ct^ method.

### 2.7 Isolation of TICs and FCM

PDAC tumors were isolated, washed in PBS, and minced. The tissues were digested with an enzyme mix suspension (Tumor Dissociation Kit; Miltenyi Biotec, Bergisch Gladbach, Germany) as previously described [17]. TICs were obtained, suspended in PBS/2% bovine serum albumin (Sigma‐Aldrich), and stained with Abs (Table S1) to detect surface molecules. Next, an Inside Stain Kit (Miltenyi Biotec) was used for intracellular staining. The samples were analyzed with a FACS ARIA II^®^ cytometer (BD Biosciences).

### 2.8 Isolation of splenocytes for FCM and apoptosis assay

Splenocytes (SPCs) were isolated from Retro‐PAN02‐CSTA PDAC model mice that did or did not receive doxycycline for the induction of CSTA. For the FCM analysis, SPCs were suspended in PBS/2% bovine serum albumin (Sigma‐Aldrich) and stained with Abs (Table S1) to detect surface molecules and immune checkpoint markers. The samples were analyzed with a FACS ARIA II^®^ cytometer (BD Biosciences). For the apoptosis assay, 1 × 10^5^ SPCs were activated at 37 °C for 48 h with 200 μL of RPMI 1640 medium (Nacalai Tesque, Inc.), 10% FBS, 2 mM GlutaMAX™ Supplement, 100 units·mL^−1^ of human recombinant IL‐2 (FUJIFILM Wako Pure Chemical Industries), and 1 × 10^5^ beads/2.5 μL of pre‐washed Dynabeads™ Mouse T‐Activator CD3/CD28 (bead‐to‐cell ratio of 1 : 1; Gibco, Thermo Fisher Scientific) in a 48‐well plate. Beads were removed with a DynaMag™‐2 magnet rack (Thermo Fisher Scientific) and cells were centrifuged at 800 ***g*** for 3 min. Then, viable 1 × 10^5^ anti‐CD3/CD28 bead‐activated SPCs were resuspended in 52 μL of culture medium supplemented with 100 units·mL^−1^ of murine rIL‐2 (PeproTech) and added to a suspension of PAN02 cells (5000 cells in 20 μL) at a ratio of 20 : 1. All cells were cocultured at 37 °C for 20 h in a Falcon™ Round‐Bottom Polypropylene Tube (Thermo Fisher Scientific). We quantified apoptotic PAN02 cells by gating the CD45^neg^ population and by using the APC Annexin V Apoptosis Detection Kit I (BD Pharmingen, San Diego, CA, USA) as previously described [23]. The FCM measurements were performed with a BD Accuri™ C6 Cytometer (BD Biosciences).

Next, to identify the most active subpopulations in SPCs that had been primed by CSTA, we performed a second apoptosis assay. We followed the above protocol except for the following changes: we isolated SPCs, and we used a FACS ARIA II^®^ cytometer (BD Biosciences) to selectively sort the CD3+CD11c^neg^ SPCs and CD11c+CD3^neg^ SPCs from Retro‐PAN02‐CSTA PDAC model mice that did or did not receive doxycycline for CSTA induction. We obtained CD3+CD11c^neg^ SPCs‐CSTA‐primed, CD11c+CD3^neg^ SPCs‐CSTA‐primed, CD3+CD11c^neg^ SPCs‐CSTA‐not‐primed, and CD11c+CD3^neg^ SPCs‐CSTA‐not‐primed. Then, we cocultured the following cell combinations: 1 × 10^6^ CD3+CD11c^neg^ SPCs‐CSTA‐primed with 2.5 × 10^5^ CD11c+CD3^neg^ SPCs‐CSTA‐not‐primed, and vice versa (i.e., 1 × 10^6^ CD3+CD11c^neg^ SPCs‐CSTA‐not‐primed with 2.5 × 10^5^ CD11c+CD3^neg^ SPCs‐CSTA‐primed). The combined cocultures were activated at 37 °C for 7 days with 200 μL of RPMI 1640 media (Nacalai Tesque, Inc.), 10% FBS, 2 mm GlutaMAX™ Supplement, 100 units·mL^−1^ of human recombinant IL‐2 (FUJIFILM Wako Pure Chemical Industries), and 1 × 10^5^ beads/2.5 μL of prewashed Dynabeads™ Mouse T‐Activator CD3/CD28 (Gibco, Thermo Fisher Scientific) in a 96‐well round‐bottom plate. Then, the magnetic beads were removed, the samples were processed with 7‐aminoactinomycin D (7‐AAD; BD Pharmingen), and an Annexin V assay was performed and analyzed as described above.

### 2.9 Migration assay

A migration assay was adapted from the established design [28]. Briefly, PAN02 or PAN02‐CSTA cells were plated on day 0 in a 12‐well plate (Nunc™; Thermo Fisher Scientific Inc.); 800 μL of DMEM supplemented with 10% FBS plus 100 μg·mL^−1^ penicillin and streptomycin was used as cell medium, and the cells were maintained in a 5% CO_2_ incubator at 37 °C. On day 1, Falcon^®^ permeable Boyden chambers with a 3.0‐μm high‐density PET membrane (Corning Inc., Corning, NY, USA) were inserted into the wells on top of the PAN02 or PAN02‐CSTA cells. SPCs isolated from male C57BL/6 mouse were added to the top compartment of the chambers at a concentration of 5 × 10^6^ SPCs·mL^−1^ in a medium volume of 500 μL. After a 4‐h incubation, the cell inserts were gently removed and discarded. The SPCs that migrated through the membrane and were now suspended in the lowermost compartment were recovered from the wells and used for total cell counting and for FCM analysis. Migrated SPCs were stained as previously described [17]. The Abs used for the staining are detailed in Table S1.

### 2.10 Statistical analysis

Kaplan–Meier curves were used to estimate survival while a log‐rank test was used to determine *P*‐values. Prism 8 software (GraphPad Software, San Diego, CA, USA) and Real Statistics Resource Pack [29] were used for graphing and overall statistical analyses. Each test used is described in the figure legends.

## 3 Results

### 3.1 Effect of CSTA on survival in intraperitoneal PDAC models

We successfully established PAN02‐CSTA, PAN02‐CSTA‐GFP, and PAN02‐GFP cell lines. These cell lines continuously overexpressed CSTA, CSTA‐GFP protein, and GFP, respectively (Fig. S1). We confirmed in PAN02‐CSTA‐GFP cultures that CSTA affected cell proliferation, which was now slower when compared with that in PAN02 cell cultures (Fig. S1B). Nonetheless, direct cell damage was not detected by gene expression analysis (Tables S2 and S3). When we analyzed PAN02‐CSTA and PAN02 cell lines by DNA microarray analysis, we identified 2454 genes with differential expression: 1204 genes were upregulated in PAN02‐CSTA (downregulated in PAN02) while 1250 genes were downregulated in PAN02‐CSTA (upregulated in PAN02).

We performed DAVID Enrichment Analysis [21, 22] in these two gene sets (Tables S2 and S3). The analysis of the 1204 genes upregulated by CSTA yielded clusters of terms (Table S2) associated with the following: “C2 domain”, a functional domain that, if expressed, might enhance the binding of perforin to the cell membrane [30]; “Cystatin A, and negative regulation of peptidase regulation”, functions that we expected to be upregulated; “Retinol metabolism”, “Reactive oxygen species (ROS) and reactive nitrogen species (RNS)”, and “Chemokines” release, which suggest anticancer [31] and proinflammatory [32] effects; and “Cell junctions” signaling upregulation, which could slow down cell proliferation and evasion.

Among the upregulated chemokines induced by CSTA (Table S2), *Ccl3* (*MIP‐1a*), *Ccl5* (*RANTES*), and *Ccl9* (*MIP‐1 gamma*) induce the migration of macrophages, T cells, and DCs and stimulate their interaction [33, 34, 35]. On the other hand, analysis of the 1250 genes downregulated by CSTA yielded clusters of terms (Table S3) associated with the following: “Regulation of transcription”; “Interleukin 8”, which is associated with immune suppression, cancer plasticity, and angiogenesis [36]; “Epidermal growth factor (EGF)” and “Growth factors” terms, whose related genes would slow proliferation when downregulated [37]; and, finally, dysregulation in “Extracellular matrix (ECM) organization”, which is possibly involved in cell growth and apoptosis [38]. Consequently, the slowed proliferation effect induced by CSTA is not related to a direct cytocidal effect but rather to a dysregulation of cell junctions (Table S2) and extracellular matrix deposition, with a concomitant downregulation of growth factor production (Table S3).

**Fig. 1:**
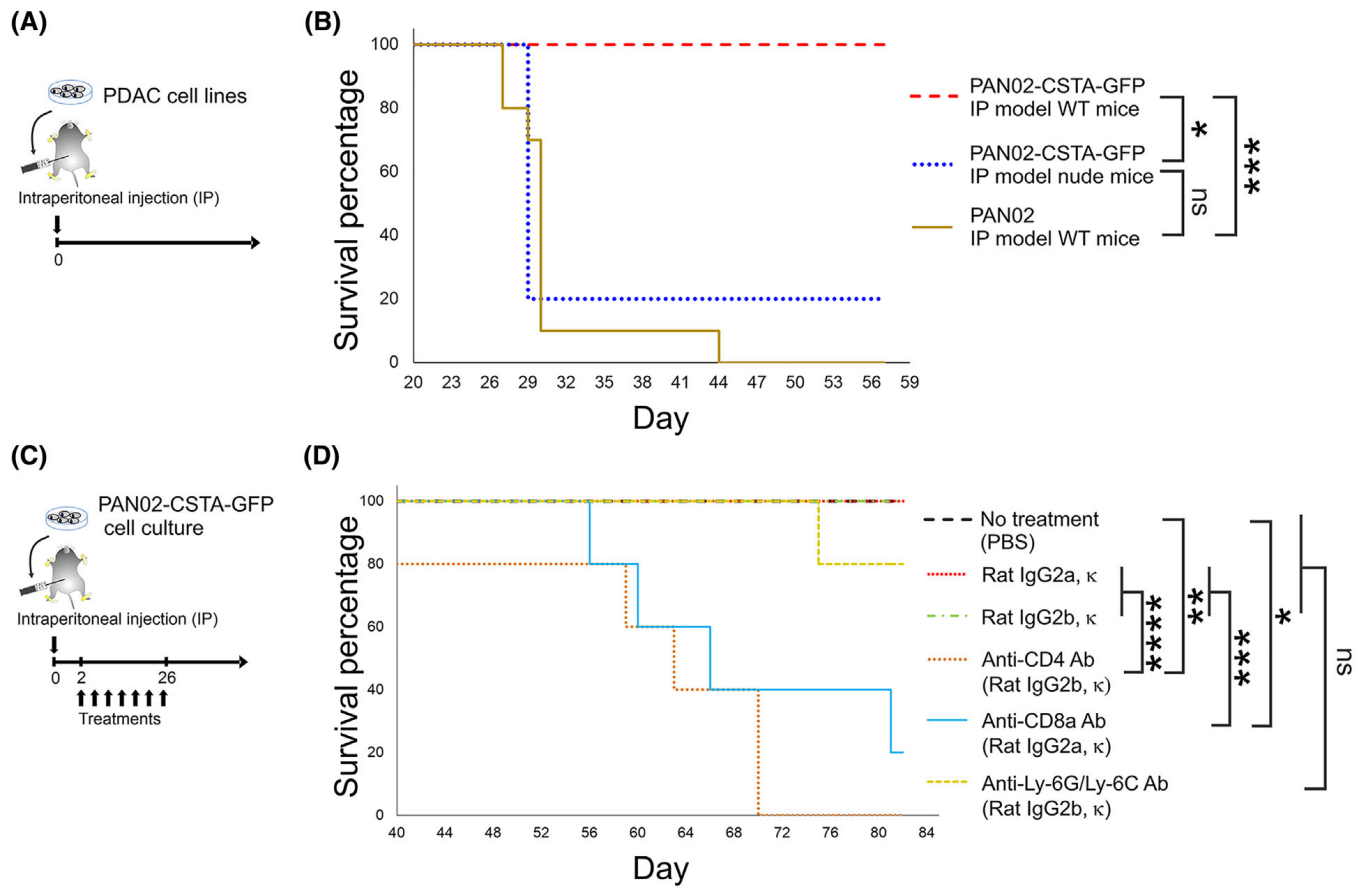
Survival of intraperitoneal (IP) pancreatic ductal adenocarcinoma (PDAC) model mice established with the PAN02‐CSTA‐GFP cell line. (A) Experimental schedule showing the establishment of IP PDAC model mice with the PAN02‐CSTA‐GFP or PAN02 cell line; survival was observed until day 57. (B) Survival curves of IP PDAC model mice. The following conditions were compared: PAN02‐CSTA‐GFP IP PDAC model of wild‐type (WT) mice (*n* = 5), PAN02‐CSTA‐GFP IP PDAC model of nude mice (*n* = 5), and PAN02 IP PDAC model of WT mice (*n* = 10). The survival test was repeated in a second independent experiment (see Fig. S2A). (C) Experimental schedule showing the establishment of IP PDAC model mice and the timing of antibody (Ab) treatments: PAN02‐CSTA‐GFP IP PDAC model mice were treated seven times in total, every 4 days, starting on day 2; survival was monitored until day 82. (D) Survival curves of PAN02‐CSTA‐GFP IP PDAC model mice that received: no treatment (only phosphate‐buffered saline [PBS]; *n* = 5), Rat IgG2a, κ (isotype control; *n* = 12), Rat IgG2b, κ (isotype control; *n* = 12), anti‐CD4 Ab ([GK1.5] rat IgG2b, κ; *n* = 5), anti‐CD8a Ab ([53–6.7] rat IgG2a, κ; *n* = 5), and anti‐Ly‐6G/Ly‐6C Ab ([RB6‐8C5] rat IgG2b, κ; *n* = 5). Antibodies are described in Table S1. (B, D) The log‐rank test was performed to determine *P*‐values; *P < 0.05, **P < 0.01, ***P < 0.001, ****P < 0.0001.

Next, we established an IP dissemination PDAC mouse model by IP injection of PAN02 or PAN02‐CSTA‐GFP cells into wild‐type (WT) or nude mice (Fig. 1A), as previously described [23]. We confirmed that the PDAC WT mouse model established with PAN02‐CSTA‐GFP cells had a longer survival than the PDAC WT mouse model established with PAN02 cells and the PDAC nude mouse model established with PAN02‐CSTA‐GFP cells (Fig. 1B; Fig. S2A). By using the same IP models and survival conditions, we investigated the tumor location. Accordingly, images of mouse peritoneal cavities were taken on day 65 after establishment. No tumors were found in the peritoneal cavity or in the liver, pancreas, or spleen of the PAN02‐CSTA‐GFP IP PDAC model of WT mice (Fig. S2B). Instead, tumors were found along the intestinal tract and in the mesenteric area, but not in the pancreas, in the PAN02‐CSTA‐GFP IP PDAC model of nude mice and the PAN02 IP PDAC model of WT mice (Fig. S2C,D). We confirmed that, regardless of the IP PDAC model and condition tested, the PAN02 and PAN02‐CSTA‐GFP cell lines did not preferentially home to the pancreas following the IP injection.

This first survival test result implied that CSTA requires T cell involvement. To further confirm this hypothesis, we established an IP PDAC mouse model of PAN02‐CSTA‐GFP cells in WT mice, followed by selective cell‐depleting treatments: no treatment; Rat IgG2a, κ (isotype control); Rat IgG2b, κ (isotype control); anti‐CD4 Ab; anti‐CD8a Ab; and anti‐Ly‐6G/Ly‐6C Ab (Fig. 1C). We observed that mice that did not receive any treatment, or received the isotype controls, had the longest survival, followed by mice treated with anti‐Ly‐6G/Ly‐6C Ab (Fig. 1D). Meanwhile, mice treated with anti‐CD8a Ab and, in particular, anti‐CD4 Ab, had the shortest survival. These results confirm that the effect of CSTA is mediated by T cells and mainly by CD4+ T cells.

### 3.2 Features of SC PDAC tumors inducibly expressing *CSTA*

The continuous overexpression of *CSTA* in PAN02‐CSTA‐GFP PDAC models ultimately led to such a reduction in tumor size that we could not sample sufficient amounts of tissues for a detailed investigation of the CSTA mechanism. Moreover, GFP expression has also been reported to be highly immunogenic [39], and this property of GFP could have partly masked, overlapped, or interfered with the antitumor effect of CSTA. To circumvent these issues, we established Retro‐PAN02‐CSTA and Retro‐PAN02‐CSTA‐GFP cell lines, which are PAN02‐derived cell lines that only express *CSTA* or *CSTA‐GFP* protein upon doxycycline induction. Using qRT‐PCR, we confirmed the successful induction of *Csta1* and *Csta1‐GFP* (control) gene expression both *in vitro* (Fig. S3A,B) and *in vivo* (Fig. S3C,D).

**Fig. 2:**
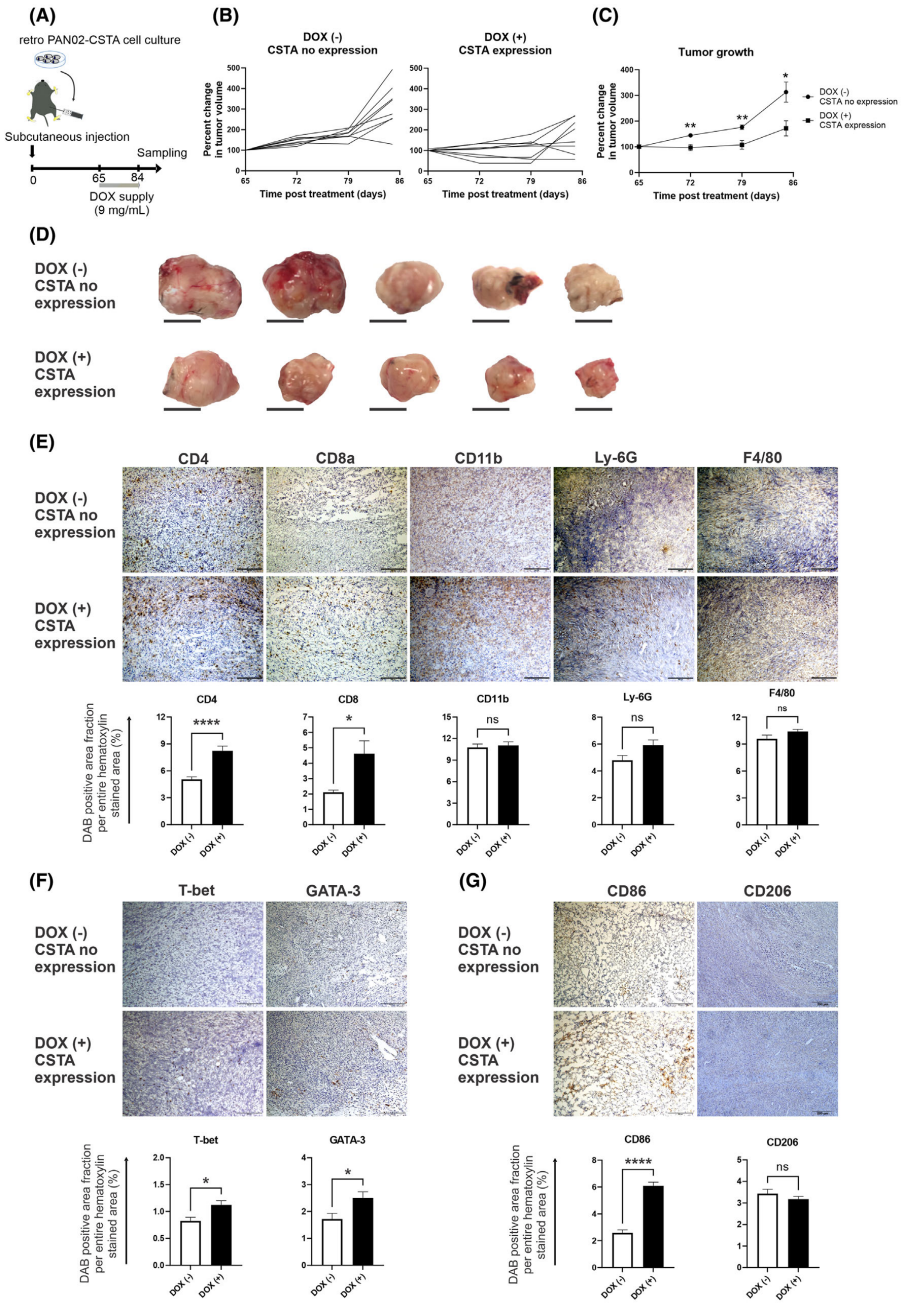
Response in subcutaneous (SC) pancreatic ductal adenocarcinoma (PDAC) model mice with tumors expressing high levels of *cystatin A* (*CSTA*). (A) Scheme of the *in vivo* establishment of the Retro‐PAN02‐CSTA SC PDAC model: mice were subcutaneously injected into the right leg with 5 × 10^6^ Retro‐PAN02‐CSTA cells suspended in 50 μL of phosphate‐buffered saline (PBS); doxycycline (DOX) was supplied in water from day 65 to day 84 for *CSTA* induction and tumors were sampled on day 84. (B) Over time, SC tumor growth of PDAC mice with either induced or not induced expression of *CSTA* in tumors; tumor size is expressed as the percentage change in volume, where 100% was set at the moment of doxycycline initiation (day 65; average tumor size: 806 ± 443 mm^3^). (C) Average values of the percent change in the tumor volumes presented in panel B; (B and C) *n* = 8. (D) Macroscopic images of tumors are shown; scale bar: 10 mm. (E) Immunohistochemical analysis of tumors sampled on day 84 for CD4+, CD8a+, CD11b+, Ly‐6G+, and F4/80+ inflammatory cells. (F) Analysis of lymphoid cell subsets: T‐bet+ for Th1 T cells and GATA‐3+ for Th2 T cells. (G) Analysis of myeloid lineage subsets: CD86+, mainly for M1‐type macrophages; and CD206, representative of M2‐type macrophages. (E–G) Antibody details are described in Table S1. Magnification: ×100; scale bar: 200 μm. ImageJ software was used to quantify inflammatory cell infiltration; DAB, diaminobenzidine. (C–G) Bars represent mean ± SEM; *n* = 8, Student's *t*‐test was performed for statistical analysis; *P < 0.05, **P < 0.01, ****P < 0.0001.

Next, we established a SC PDAC mouse model by subcutaneously injecting Retro‐PAN02‐CSTA cells into WT mice. In one group of mice, *CSTA* expression was induced in tumors by doxycycline supplied in water from day 65 to day 84 while, in the other group, *CSTA* expression was not induced (Fig. 2A). The tumor volume continually increased in mice with no induction of *CSTA*, whereas the tumors expressing *CSTA* grew minimally or stayed the same size (Fig. 2B–D). Histologically, the number of infiltrating CD4+ and CD8a+ cells increased in tumors of mice expressing *CSTA* compared with tumors without *CSTA* expression; meanwhile, no difference was observed in the infiltration of myeloid cells: CD11b+, Ly‐6G+, or F4/80+ cells (Fig. 2E). Furthermore, we confirmed increased numbers of T‐bet+ cells, as well as Gata3+ cells (Fig. 2F). Regarding myeloid subsets (Fig. 2G), we observed a steep increase in the infiltration of CD86+ cells, mainly M1‐type macrophages. Meanwhile, no change was noted in CD206+ M2‐type macrophages. The observed differences in phenotypes might seem relatively mild, but this is due to the short treatment window (day 64 to day 84; Fig. 2A). Considering that the *CSTA* induction starts when the tumors are already fully established, with a robust immunosuppressive microenvironment, we conclude that CSTA clearly exhibits overall proinflammatory effects.

### 3.3 Gene expression profile of tumor tissues altered by CSTA

Next, we used DNA microarray gene expression profile analysis to examine how tumor tissues were affected in the murine SC PDAC model of Retro‐PAN02‐CSTA by the induced expression of *CSTA*. We observed that 2455 genes were differentially regulated (parametric *P* < 0.05; Table S4) in tumors expressing *CSTA* when compared with tumors not expressing *CSTA*. Precisely, 1684 genes were upregulated in tumor tissue by CSTA (downregulated in tumor tissue without CSTA; gene set A) and 771 genes were downregulated in tumor tissue by CSTA (upregulated in tumor tissue without CSTA; gene set B). Among gene set A, the upregulation was confirmed of key proinflammatory chemokines: *Ccl3*, *Ccl4*, *Ccl5*, *Ccl8*, and *Cxcl16*. These chemokines induce the chemotaxis of T cells, DCs, and macrophages by interacting with Ccr5 and Cxcr6 receptors [33, 34, 40, 41, 42].

Then, we performed MetaCore enrichment analysis for these two gene sets: the identified pathway maps related to gene set A were largely associated with the immune response, mainly, “IFN‐gamma in macrophages activation”, “Induction of the antigen presentation machinery by IFN‐gamma”, and “Macrophage and dendritic cell phenotype shift in cancer”. These pathway maps are indicative of macrophage and dendritic cell anticancer activity mediated by IFN‐γ, as well as the other suggested pathway lymphoid activation (Table 1; Table S5). The identified pathway maps related to gene set B were associated with downregulated regulation of the cell cycle, checkpoint, and apoptosis in the presence of CSTA within the tumor tissues, confirming a diminished proliferation of cancer cells (Table 1; Table S6).

**Table 1:** Summary of MetaCore Enrichment Analyses (*P* < 0.01) for 2455 genes differentially expressed (parametric *P* < 0.05) in tumor tissues expressing *CSTA*.

MetaCore enrichment analysis by pathway maps of 1684 genes upregulated in tumor tissue by cystatin A (downregulated in tumor tissue in absence of cystatin A; gene set A)	MetaCore enrichment analysis by pathway maps of 771 genes downregulated in tumor tissue by cystatin A (upregulated in tumor tissue in absence of cystatin A; gene set B)
Within the top 50 pathways (Table S5): 43 are related to Immune response or Immune related pathways: IFN‐gamma in macrophages activation (*P* = 1.0E‐16) Classical complement pathway (*P* = 7.3E‐15) Induction of the antigen presentation machinery by IFN‐gamma (*P* = 7.3E‐15) Antigen presentation by MHC class I: cross‐presentation (*P* = 2.6E‐12) IFN‐alpha/beta signaling via JAK/STAT (*P* = 3.8E‐12) Macrophage and dendritic cell phenotype shift in cancer (*P* = 2.1E‐11) Common mechanisms of Th17 cell migration (*P* = 1.2E‐10) NK cells in allergic contact dermatitis (*P* = 2.8E‐10) Antigen presentation by MHC class II (*P* = 6.0E‐09) Role of tumor‐infiltrating B cells in antitumor immunity (*P* = 1.4E‐08) IFN‐gamma actions on blood cells (*P* = 3.0E‐07) IL‐10 signaling pathway (*P* = 5.3E‐07)	Within the top 50 pathways (Table S6): 17 are related to Cell cycle: The metaphase checkpoint (*P* = 3.2E‐21) Chromosome condensation in prometaphase (*P* = 3.1E‐19) Role of APC in cell cycle regulation (*P* = 1.0E‐15) Spindle assembly and chromosome separation (*P* = 5.8E‐14) 8 are related to DNA damage: ATM/ATR regulation of G2/M checkpoint: nuclear signaling (*P* = 4.6E‐16) ATM/ATR regulation of G2/M checkpoint: cytoplasmic signaling (*P* = 5.5E‐10) Intra S‐phase checkpoint (*P* = 1.3E‐15) 2 are related to Apoptosis and survival: DNA‐damage‐induced apoptosis (*P* = 2.3E‐04) p53‐dependent apoptosis (*P* = 3.3E‐04)

Furthermore, by using all 19 667 genes that passed the filtering criteria, we performed a Geneset Class Comparison Analysis using cell type‐related gene sets to estimate the variation in the type of cells within the tumor tissue under different conditions. Gene sets that were upregulated when *CSTA* expression was induced in tumors exhibited an increased presence of M1 macrophages, CD4+ T cells, both Th1 and Th2 type cells, CD8+ T cells, and DCs (Table S7, Fig. S4). In particular, the gene set identifying the DC5 subset was upregulated by *CSTA* expression. Specifically, DC5 cells were confirmed to potently activate T cells [43].

**Fig. 3:**
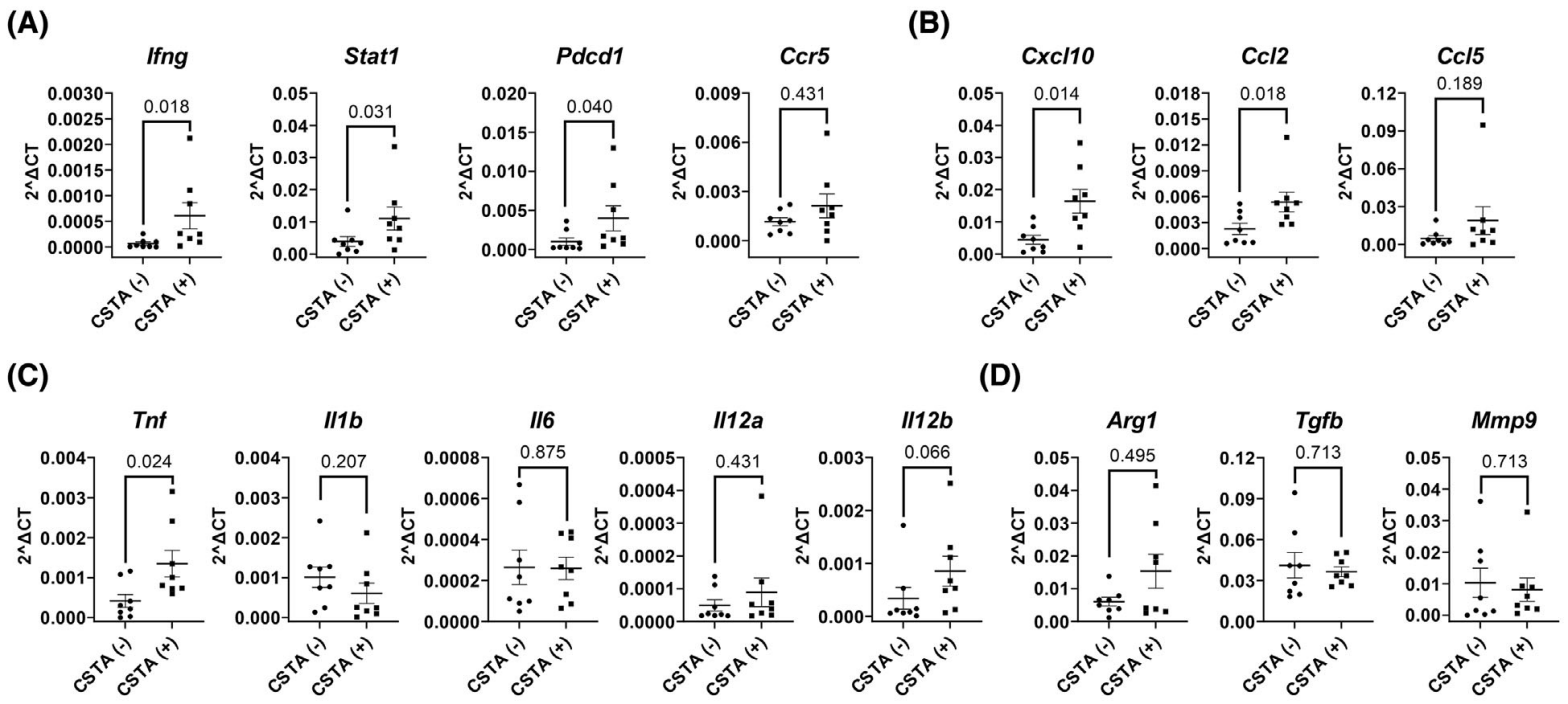
Gene expression analysis of tumor tissues from subcutaneous (SC) pancreatic ductal adenocarcinoma (PDAC) model mice by quantitative real‐time PCR (qRT‐PCR). Doxycycline was supplied in water from day 65 to day 84 to the Retro‐PAN02‐CSTA SC PDAC model mice for *cystatin A* (*CSTA*) induction; tumors were sampled on day 84, followed by RNA isolation. qRT‐PCR showing the expression of (A) T cell activation markers (*Ifng*, *Stat1*, *Pdcd1*, and *Ccr5*), (B) proinflammatory chemokines (*Cxcl10*, *Ccl2*, and *Ccl5*), (C) M1 macrophage‐related proinflammatory cytokines (*Tnf*, *Il1b*, *Il6*, *Il12a*, and *Il12b*), and (D) M2 macrophage‐related genes (*Arg1*, *Tgfb1*, and *Mmp9*); *n* = 8; (A–D) bars represent mean ± SEM; a two‐tailed Mann–Whitney test was used for statistical analysis.

Then, we examined the gene expression of cytokines related to anticancer immunity in tumor tissues from PDAC tumors that were expressing or not expressing *CSTA*. Using qRT‐PCR, we confirmed a CSTA‐induced increase in the T cell activation markers *Ifng*, *Stat1*, and *Pdcd1*, and only a very small increase in *Ccr5* expression (Fig. 3A). A strong increase was observed in the expression of *Cxcl10*, a chemokine responsible for the recruitment of activated T cells at sites of inflammation [44], and in the expression of *Ccl2*, which recruits monocytes, memory T cells, and DCs (Fig. 3B). In addition, a slight tendency toward an increase was observed in the expression of *Ccl5*. Among the M1 macrophage‐related cytokines, we observed a definitive increase in the expression of *Tnf* and a clear increasing tendency in the expression of *Il12b* (Fig. 3C). No difference was detected in the expression of *Il1b*, *Il6*, and *Il12a*. Among M2 macrophage‐related cytokines, we did not observe any change in the expression of *Arg1*, *Tgfb*, or *Mmp9* (Fig. 3D). Overall, we confirmed that the presence of CSTA within the tumor induced a strong proinflammatory and anticancer environment.

### 3.4 Features of lymphoid and myeloid lineage cells in TICs and SPCs in the presence of high levels of CSTA

**Fig. 4:**
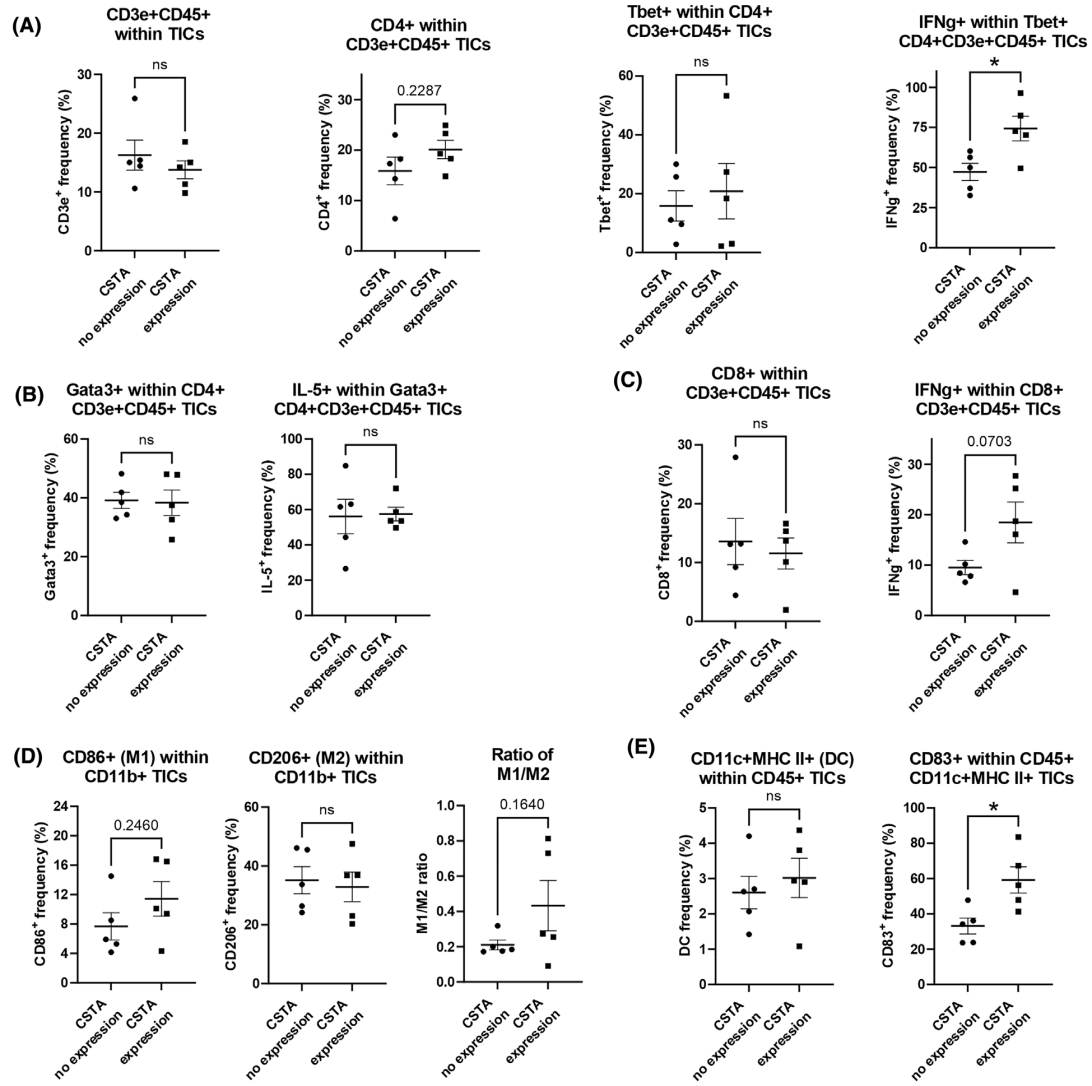
Flow cytometry (FCM) analysis of tumor‐infiltrating inflammatory cells (TICs) for lymphoid and myeloid lineage cells. Doxycycline was supplied in water from day 65 to day 84 to Retro‐PAN02‐CSTA SC PDAC model mice for *cystatin A* (*CSTA*) induction; tumors were sampled on day 84 for TIC isolation, followed by FCM analysis; *n* = 5. (A) CD3e+CD45+ TICs, CD4+ subpopulation in CD3+CD45+ TICs, T‐bet+ (Th1) subpopulation in CD4+CD3+CD45+ TICs, and IFN‐γ+ subpopulation in Th1 TICs. (B) Gata3+ (Th2) subpopulation in CD4+CD3+CD45+ TICs and IL‐5+ subpopulation in Th2 TICs. (C) CD8+ subpopulation in CD3+CD45+ TICs and IFN‐γ+ subpopulation in CD8+CD3+CD45+ TICs. (D) CD86+ subpopulation in CD11b+ TICs (M1 macrophages), CD206+ subpopulation in CD11b+ TICs (M2 macrophages), and the M1/M2 macrophage ratio in CD11b+ TICs. (E) CD11c+MHCII+ subpopulation in CD45+ TICs (dendritic cells; DCs) and activated CD83+ DCs subpopulation in TICs. (A–E) Antibodies are detailed in Table S1. Bars represent mean ± SEM; Student's *t*‐test was used for statistical analysis; *P < 0.05.

Antitumor immunity was significantly promoted in PDAC tumors in the presence of CSTA, confirmed by decreased tumor sizes and by histological and gene expression analyses. To further assess the features of immune‐mediating cells, we isolated tumor‐infiltrating lymphoid and myeloid lineage cells and assessed phenotypes using FCM analysis. No difference was observed in the frequency of CD45+CD3e+ TICs (Fig. 4A; Fig. S5A) but we confirmed a tendency toward an increase in the CD4+ subpopulation in CD3e+CD45+ TICs of tumors in the presence of CSTA (Fig. 4A; Fig. S5B). No difference was detected in T‐bet+ frequency in CD4+CD3e+CD45+ (Th1) TICs, but the IFN‐γ+ frequency in Th1 TICs was much higher in tumors expressing *CSTA* than in tumors without *CSTA*. This confirmed the strong change in the characteristics of the CD4+ TICs in tumors exposed to CSTA (Fig. 4A; Fig. S5C). No difference was observed in the frequency of Th2 T cells (Gata3+ in CD3e+CD4+CD45+ TICs) or in the expression of IL‐5 in Th2 TICs (Fig. 4B; Fig. S5D). In addition, we found no difference in the number of CD8+ cells in CD3e+CD45+ TICs of tumors expressing or not expressing *CSTA*, but a tendency toward an increase was clearly shown in the expression of IFN‐γ in CD8+CD3e+CD45+ TICs of tumors expressing *CSTA* (Fig. 4C; Fig. S5E). Overall, the lymphoid lineage cells acquired a clear proinflammatory phenotype, with Th1 T cells possibly one of the main cell subsets involved in the antitumor effect.

Next, we analyzed the CD86+ subpopulation in CD11b+ TICs (M1 macrophages) and the CD206+ subpopulation in CD11b+ TICs (M2 macrophages). We confirmed that, in tumors expressing *CSTA*, the M1/M2 macrophage ratio in CD11b+ TICs was generally higher than in tumors without induced expression of *CSTA* (Fig. 4D; Fig. S5F), mainly due to an increase in M1 macrophages. The CD11c+MHCII+ subpopulation in CD45+ TICs (DCs) did not show a change in frequency among conditions but, notably, the activated CD83+ subpopulation in DCs was almost twice as high in frequency in tumors expressing *CSTA* (Fig. 4E; Fig. S5G).

We further used qRT‐PCR to analyze the TICs in the context of lymphoid response activation and immune checkpoint expression. We confirmed that *Il2ra* expression was elevated following *CSTA* induction, which might be involved in T cell receptor activation of CD4+ and CD8+ T lymphocytes [45]. On the other hand, *Cd69*, a marker of early activation, only slightly changed (Fig. S6A). Specifically regarding immune checkpoint molecules (Fig. S6B), following CSTA treatment, we observed an overall decrease in the expression of *Pdcd1* (PD‐1) in TICs. This was in direct opposition to the enhanced expression of PD‐1 in the overall tumors (Fig. 3A). This difference might be due to the possible increased expression of PD‐1 in tumor cells, supporting the evidence that PD‐1 molecules are not exclusive to immune cells [46]. The other immune checkpoint molecules tested—*Havcr2* (TIM‐3), *Lag3*, and *Tigit*—all exhibited increased or slightly increased expression following CSTA exposure, suggesting that the inflammation caused by CSTA is sustained. All of these molecules could be possible targets for combination therapy.

The features of SPCs were also investigated to evaluate the host immune response for specific lymphoid cell populations for activation of and changes in immune checkpoint molecule expression with CSTA. FCM results showed no major changes in the expression of PD‐1+ and TIM‐3+ subpopulations in CD45+CD4+ SPCs but a mild increase in LAG‐3+ in CD45+CD4+ SPCs (Fig. S7). This mild increase might be indicative of antigen stimulation [47] and of a compensatory mechanism due to enhanced host inflammation and sustained activity of CD4+ cells. Furthermore, no major changes were observed in the expression of PD‐1+ and LAG‐3+ subpopulations in CD45+CD8+ SPCs, while we found a mild decrease in TIM‐3+ cells in CD45+CD8+ SPCs (Fig. S8). Regarding natural killer (NK) cells in SPCs, their frequency slightly increased when *CSTA* was expressed in the host (Fig. S9A,B). PD‐1 expression in CD335+CD45+ SPCs increased with CSTA (Fig. S9C,D). Interestingly, the presence of PD‐1 molecules on NK cells and the changes in their expression differed from that of PD‐1 molecules on T cells [48]. This difference provides possible research avenues related to combination therapy. Regarding NK cells, no change was seen in LAG‐3+ cells. Meanwhile, a slight decrease in the expression of TIM‐3+ molecules was confirmed in CD45+CD335+ SPCs. This slight decrease in TIM‐3 in both CD8+ T cells and NK cells suggested that the functionality and cytotoxicity of these cells was further improved with CSTA (Figs S8B and S9D).

**Fig. 5:**
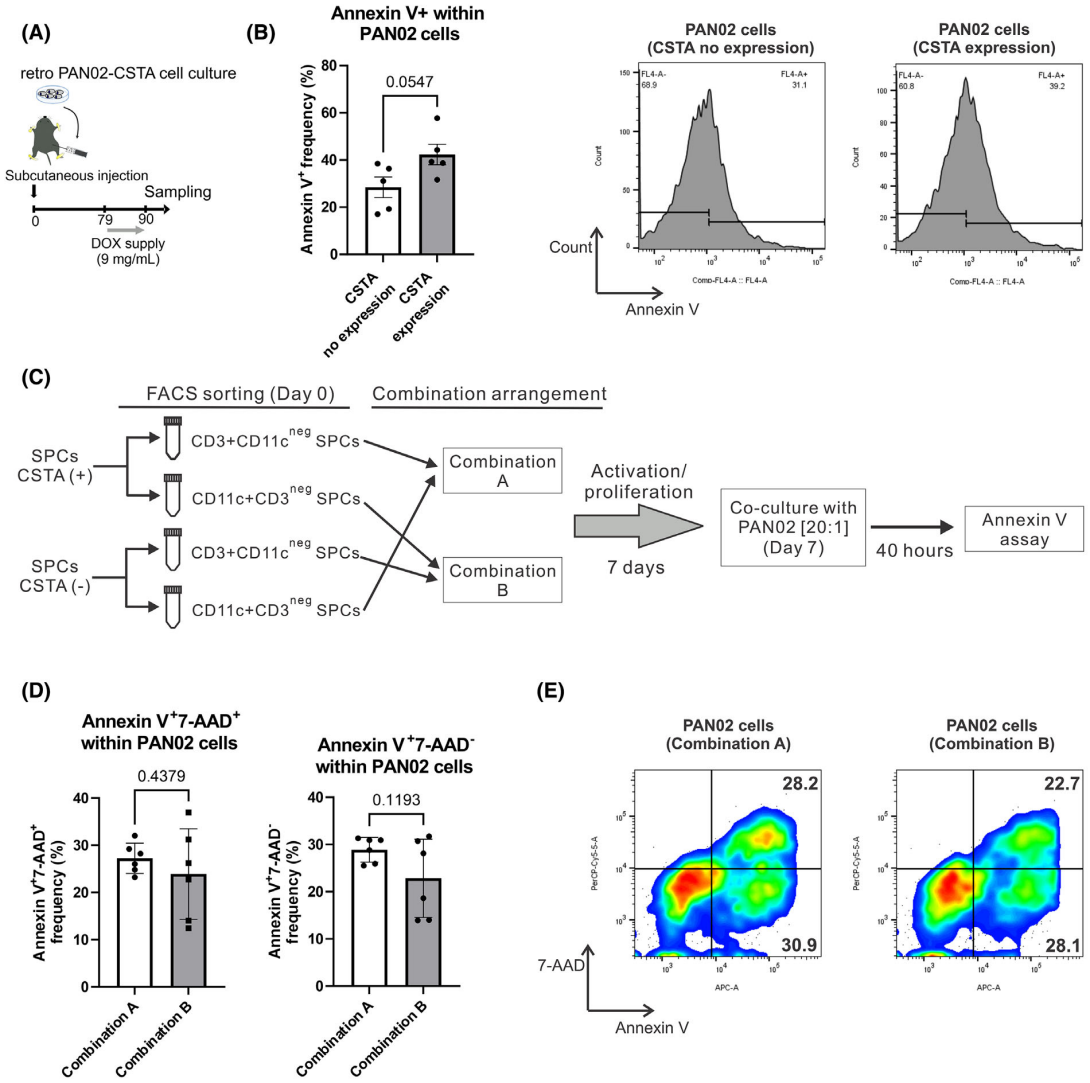
Cytotoxic effect of activated splenocytes (SPCs) induced by cystatin A (CSTA). (A) Doxycycline (DOX) was supplied in water from day 79 to day 90 to Retro‐PAN02‐CSTA SC PDAC model mice for *CSTA* induction; SPCs from treated and untreated mice were isolated on day 90. The SPCs from either the CSTA‐positive or CSTA‐negative group were cultured in medium with 100 units·mL^−1^ of murine IL‐2 and anti‐CD3/CD28 beads for 48 h. Then, the anti‐CD3/CD28 bead‐activated SPCs were further cocultured with PAN02 at a ratio of 20 : 1 for 20 h in polypropylene tubes. The cells were stained with phycoerythrin (PE) anti‐CD45 and allophycocyanin (APC) Annexin V for identifying apoptotic PAN02 cells by flow cytometry (FCM). (B) Annexin V+ cell frequency comparison and representative FCM histograms are depicted (*n* = 5; data is derived from one experiment). The same SPCs, isolated from Retro‐PAN02‐CSTA PDAC model mice that received or did not receive doxycycline for the induction of *CSTA* (panel A), were used for the selective fluorescence activated cell sorting (FACS) of CD3+CD11c^neg^ T cells and CD11c+CD3^neg^ dendritic cells (see scheme in panel C). (C) CD3+CD11c^neg^ SPCs‐CSTA‐primed were cocultured with CD11c+CD3^neg^ SPCs‐CSTA‐not‐primed (combination A), and vice versa (i.e., CD3+CD11c^neg^ SPCs‐CSTA‐not‐primed with CD11c+CD3^neg^ SPCs‐CSTA‐primed) (combination B). Each combination was activated for 7 days and then cocultured with PAN02 at a ratio of 20 : 1 for 40 h in polypropylene tubes. The cells were stained with PE anti‐CD45, 7‐aminoactinomycin D (7‐AAD), and APC Annexin V for the detection of dead and apoptotic PAN02 cells by FCM analysis. (D) Annexin V+7‐AAD+ (dead) and Annexin+7‐AAD^neg^ (apoptotic) PAN02 cell frequency comparison (*n* = 6) and (E) one representative scatter plot for each condition is depicted. (C–E) Data are derived from one experiment. (B, D) Bars represent mean ± SEM; Student's *t*‐test was used for statistical analysis. (B, D, E) Antibodies are described in Table S1.

Finally, we also analyzed the phenotypes of B cells in SPCs (Fig. S10). The overall frequencies of the CD138+CD45R+ subpopulation in CD19+CD45+ SPCs (plasma cells) and of the CD138^neg^CD45R+ subpopulation in CD19+CD45+ SPCs (non‐plasma cells) were not affected by CSTA. Moreover, CSTA induced higher PD‐1 expression in non‐plasma cells, whereas only a mild tendency toward increased PD‐1 expression was observed in activated plasma cells (Fig. S10B). Especially within the PD‐1+ non‐plasma cell subpopulation [49], where the presence of regulatory B cells would be higher, cells might have acquired immunosuppressive regulatory functions in response to the CSTA proinflammatory effect. Therefore, within this context, we can assume that CSTA treatment would further benefit from combination therapy by blocking the PD‐1 molecule. We further confirmed by apoptosis assay, that *ex vivo* anti‐CD3/CD28 bead‐activated SPCs, obtained from SC Retro‐PAN02‐CSTA PDAC model mice expressing *CSTA*, induced higher apoptosis in targeted PAN02 cells when compared to *ex vivo* anti‐CD3/CD28 bead‐activated SPCs isolated from mice without *CSTA* induction (Fig. 5A,B). Moreover, we wanted to identify the main active populations in SPCs that were primed by CSTA by performing a second apoptosis assay: we isolated SPCs from Retro‐PAN02‐CSTA PDAC model mice that received or did not receive doxycycline for *CSTA* induction and selectively sorted the CD3+CD11c^neg^ T cells and CD11c+CD3^neg^ DCs from the entire SPC population. We cocultured CD3+CD11c^neg^ SPCs‐CSTA‐primed with CD11c+CD3^neg^ SPCs‐CSTA‐not‐primed (combination A), and vice versa (i.e., CD3+CD11c^neg^ SPCs‐CSTA‐not‐primed with CD11c+CD3^neg^ SPCs‐CSTA‐primed) (combination B; Fig. 5C). Following activation/proliferation for 7 days and via an apoptosis assay, we confirmed a clear tendency for a higher cytotoxic effect with combination A compared with combination B (Fig. 5D,E), implying that T cells are the main population involved in the antitumor effect of CSTA.

**Fig. 6:**
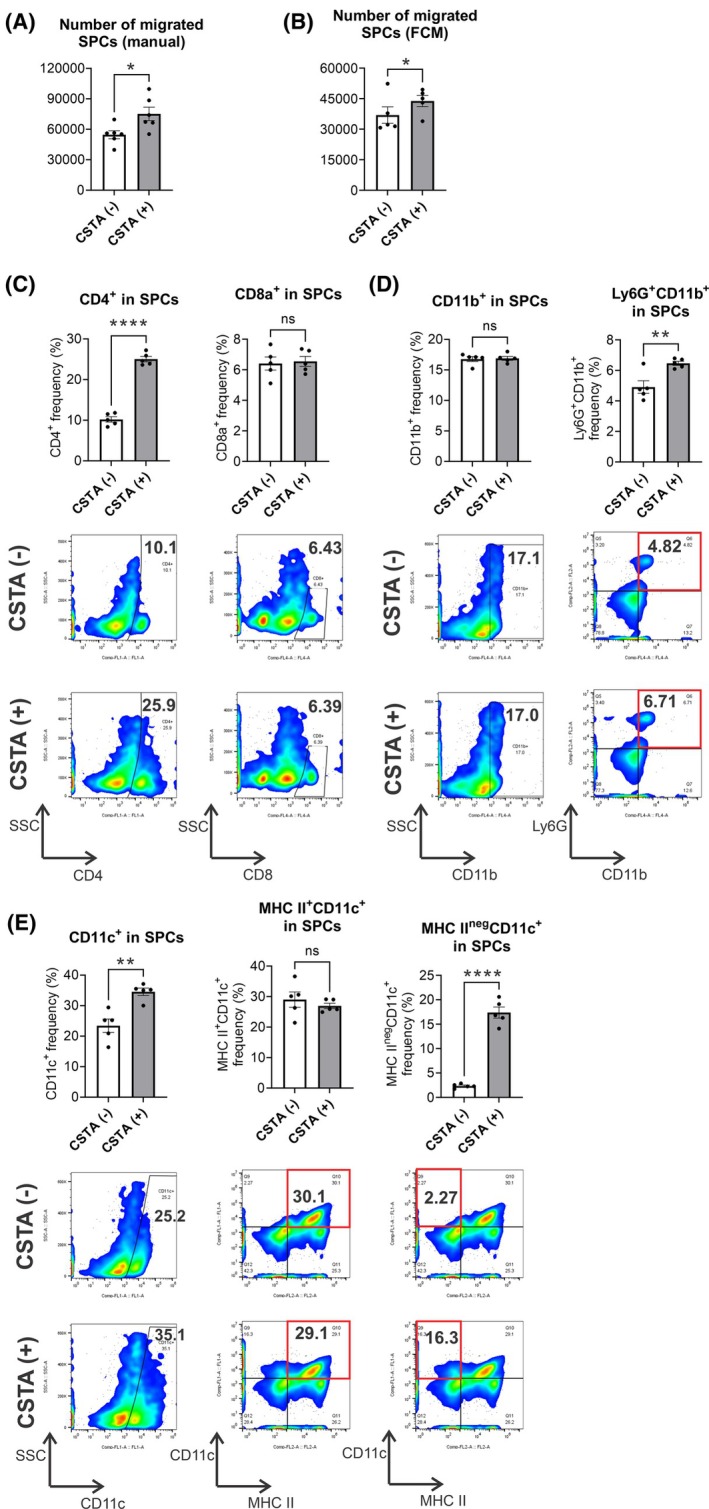
Immune cell recruitment induced by cystatin A (CSTA) *in vitro*. (A) Manual cell counts (*n* = 6) and (B) flow cytometry absolute cell counts of migrated splenocytes (SPCs) recovered from the supernatant of the lowermost compartment of wells following the removal of insert wells (*n* = 6). (C) CD4+ and CD8a+ cells in migrated SPCs. (D) CD11b+ and Ly6g+CD11b+ cells within migrated SPCs. (E) CD11c+, MHCII+CD11c+, and MHCII^neg^CD11b+ cells in migrated SPCs. (C–E) Antibodies are described in Table S1. (A–E) Bars represent mean ± SEM; Student's *t*‐test was used for statistical analysis; *P < 0.05, **P < 0.01, ****P < 0.0001. (B–E) *n* = 5.

Finally, by using permeable Boyden chambers, we confirmed *in vitro* that CSTA induced the migration of SPCs (Fig. 6A,B) and, precisely, the selective enhanced recruitment of CD4+ SPCs, but not CD8+ SPCs (Fig. 6C). There was no change in the frequency of CD11b+, although a slight increase in Ly6G+CD11b+ SPCs was detected (Fig. 6D). Moreover, we observed a distinct migration of CD11c+ SPCs (Fig. 6E). A specific subset of CD11c+ cells, MHCII^neg^CD11c+ SPCs, increased by about 7‐fold in frequency, while MHCII+CD11c+ SPCs did not. MHCII^neg^CD11c+ cells have been identified in mice as partial precursors of DCs [50], and they might migrate and differentiate when *CSTA* is expressed locally.

## 4 Discussion

PDAC is a disease with very poor prognosis [1]. Most cases are discovered in the final stages of the disease, metastases develop aggressively, and surgical treatment is not possible. Chemotherapeutics such as gemcitabine have limitations due to reduced efficacy and acquired resistance mechanisms [51]. FOLFIRINOX (a combination of oxaliplatin, irinotecan, 5‐fluorouracil, and leucovorin) shows improved outcomes over gemcitabine monotherapy in terms of the antitumor response and progression‐free survival but is associated with higher levels of toxicity compared with other therapies [52]. Accordingly, innovative treatments are urgently needed.

We previously confirmed that PDAC patients have increased expression of *CSTA* within CD4+ T cells of peripheral blood compared with healthy patients, as well as a role for CSTA as a biomarker in PDAC [6]. In this regard, *CSTA* expression has been observed in several tumor tissues [6, 8, 53, 54], and it has been recognized as having diagnostic significance [6, 10, 15]. Furthermore, depending on tumor type, CSTA appears to modulate both antitumor [16, 53] and protumor [15, 55] responses.

In the present study, we evaluated whether CSTA has an anticancer effect in mouse models of pancreatic cancer. We confirmed that *CSTA* expression in tumors exerted an overall antitumor effect, prolonging the survival and reducing the tumoral masses of PDAC mouse models. To our knowledge, this is the first report of the antitumor properties of CSTA in a PDAC murine model. The frequencies of CD4+ T cells, CD8+ T cells, and M1 macrophages were increased in tumor tissues, as confirmed by immunohistochemical analysis. When we analyzed the subsets of CD4+ T cells and myeloid cells, we confirmed an enhanced activity of Th1 T cells, activated CD83+ dendritic cells, and IFN‐γ‐activated macrophages. Moreover, CSTA altered the gene expression profile in DNA microarray of tumor tissues. The genes upregulated in tumor tissue expressing *CSTA* (downregulated in tumor tissue without *CSTA*) were related to pathways associated with the antitumor immune response: mainly IFN‐γ and IFN‐α/β regulation for activation of macrophages and DCs, but also enhanced antigen presentation activity. On the other hand, the genes downregulated in tumor tissue expressing *CSTA* (upregulated in tumor tissue without *CSTA*) were related to pathways associated with regulation of the cell cycle, checkpoint, and apoptosis, suggesting an overall reduced proliferation activity of cancer cells.

Following the Geneset Class Comparison Analysis for estimating the changes in cell types within tumor tissue in the presence of CSTA, we also confirmed the increased presence of M1 macrophages, CD4+ T cells, CD8+ T cells, and DCs. Moreover, by qRT‐PCR, tumors expressing *CSTA* showed an increase in the T cell activation markers *Ifng*, *Stat1*, and *Pdcd1*, higher expression of *Cxcl10*, a chemokine responsible for the recruitment of activated T cells at sites of inflammation [21], and higher expression of *Ccl2*, which recruits monocytes, memory T cells, and DCs. Among the M1 macrophage‐related cytokines, we also observed an increase in the expression of proinflammatory *Tnf*, which contributes to the proinflammatory environment. After analyzing the TICs with FCM, we found that the IFN‐γ+ frequency within Th1 TICs was higher in tumors expressing *CSTA* compared with control, as well as a clear increase in the expression of IFN‐γ in CD8+ TICs. This correlation between CSTA and enhanced IFN‐γ+ activity was also observed in our previous report in the clinical setting, where concentrations in blood sera were significantly higher in PDAC patients [6]. When we analyzed the CD86+ subpopulation in CD11b+ TICs (M1 macrophages) and the CD206+ subpopulation in CD11b+ TICs (M2 macrophages), we confirmed that, in tumors expressing *CSTA*, the M1/M2 macrophage ratio was higher than in tumors without *CSTA* expression. Moreover, activated CD83+ DCs were more prominent in tumors expressing *CSTA*. In addition, following qRT‐PCR analysis of TICs, we confirmed the enhanced expression of *Il2ra* and, thus, the superior CSTA‐induced activation of CD4+ and CD8+ T lymphocytes, as well as enhanced immune checkpoint expression of *Lag3*, *Havcr2* (TIM‐3), and *Tigit*, suggesting that the inflammation caused by CSTA is sustained.

To evaluate the host immune response in the context of lymphoid cell activation and changes in immune checkpoint molecule expression, we also isolated SPCs from SC Retro‐PAN02‐CSTA PDAC model mice expressing *CSTA*. Overall, only a limited change in the expression of immune checkpoint molecules was observed, except for some enhanced expression of PD‐1 in CD45+CD335+ SPCs (NK cells) and CD138^neg^CD45R+CD19+CD45+ SPCs (non‐plasma cells). In this context, we can hypothesize that CSTA treatment would act synergistically with PD‐1 blocking therapy by potentiating the effect of NK cells and by limiting the immunosuppressive effect of PD‐1+ non‐plasma cells.

Finally, we confirmed that the same isolated SPCs acquired a higher proapoptotic capability upon PAN02 cell targeting *in vitro*. More precisely, we were able to identify T cells as the main mediators of this effect within the SPCs. We also confirmed a further *in vitro* mechanism: CSTA induced the overall migration of immune cells, recruiting CD4+ as well as CD11c+ cells. MHCII^neg^CD11b+ cells, possible DCs precursors [50], were also greatly attracted by the expression of *CSTA*. In this regard, selective immune cell migration might be an indirect effect of CSTA, precisely by inducing PAN02 cells to produce specific chemokines. Indeed, when we analyzed gene expression using DNA microarray, we confirmed, in PAN02‐CSTA cells, the upregulation of *Ccl3*, *Ccl5*, and *Ccl9*, all chemokines that greatly induce the migration and interaction of macrophages, T cells, and DCs [33, 34, 35]. In addition, cumulative proinflammatory activity might also at least partly result from the selective inhibition of cathepsin B and L by CSTA [56], causing a shifting of the inflammatory response toward a Th1 phenotype [57] as well as inducing an alteration in the polarization from M2 to M1 macrophages [58]. Taken together, we confirmed that CSTA induced a very distinct anticancer immunity environment in PDAC tumor by enhancing activity and recruitment. We believe that this will be an innovative direction in terms of future anticancer options for PDAC.

## 5 Conclusion

In conclusion, to our knowledge, we are the first to report anticancer effects of CSTA in IP and SC murine models of pancreatic cancer and to confirm that the observed effects are not restricted to a single model. CSTA obtained beneficial results by acting as a proinflammatory agent and by indirectly promoting cell chemotaxis. In particular, CSTA augmented the activation and maturation of DCs and thereby increased the antigen presentation and recognition of malignant cells, reinforcing antitumoral responses by facilitating the polarization of macrophages to the M1 phenotype and, lastly, by promoting the activation of CD4+ Th1 cells and cytotoxicity against tumor cells. Although mouse models may not entirely predict clinical outcomes, we believe that this study provides a solid foundation for further investigation of CSTA as an immunomodulator of innate and adaptive immune responses in humans and that CSTA is thus a valid candidate for activation immunotherapy in patients with pancreatic cancer.

## Conflict of interest

The authors declare no conflict of interest.

## Author contributions

AN, SI, TTBH, AS, KY, YS, SK, TY: conceptualization, methodology, validation; AN, YS: data curation; AN, SI, TTBH, KY, KS, YS: investigation; AN, SI, TTBH, YS: formal analysis; AN, TTBH, YS, SK, TY: funding acquisition, resources; AN, YS, SK, TY: project administration, supervision; AN, SI, TTBH, AS, YS, SK, TY: writing – original draft. All authors agreed on the final manuscript and approved of its submission.

### Peer review

The peer review history for this article is available at https://www.webofscience.com/api/gateway/wos/peer‐review/10.1002/1878‐0261.13796.

## Supporting information


**Fig. S1.** Establishment of the PAN02‐CSTA‐GFP cell line.

**Fig. S2.** Intraperitoneal (IP) pancreatic ductal adenocarcinoma (PDAC) model mice established with PAN02‐CSTA‐GFP or PAN02 cell lines.

**Fig. S3.** Establishment of Retro‐PAN02‐CSTA and Retro‐PAN02‐CSTA‐GFP cell lines.

**Fig. S4.** DNA microarray analysis of PDAC tumor tissues in the presence or not of *cystatin A* (*CSTA*) expression.

**Fig. S5.** Flow cytometry (FCM) analysis of lymphoid and myeloid lineage cells in tumor‐infiltrating inflammatory cells.

**Fig. S6.** Gene expression analysis of tumor‐infiltrating inflammatory cells (TICs) from subcutaneous (SC) pancreatic ductal adenocarcinoma (PDAC) model mice by quantitative real‐time PCR (qRT‐PCR).

**Fig. S7.** Flow cytometry (FCM) analysis of splenocytes (SPCs) for CD4+ immune lineage cells and the expression of their immune checkpoint molecules.

**Fig. S8.** Flow cytometry (FCM) analysis of splenocytes (SPCs) for CD8+ immune lineage cells and the expression of their immune checkpoint molecules.

**Fig. S9.** Flow cytometry (FCM) analysis of splenocytes (SPCs) for CD335+ natural killer (NK) cells and the expression of their immune checkpoint molecules.

**Fig. S10.** Flow cytometry (FCM) analysis of splenocytes (SPCs) for B cells, activation status, and PD‐1 immune checkpoint expression.

**Table S1.** Details on the antibodies used in the experiments.
**Table S2.** DAVID Enrichment Analysis of 1204 genes upregulated in the PAN02‐CSTA cell line (genes downregulated in the PAN02 cell line).
**Table S3.** DAVID Enrichment Analysis of 1250 genes downregulated in the PAN02‐CSTA cell line (genes upregulated in the PAN02 cell line).
**Table S4.** *CSTA*, overexpressed in pancreatic ductal adenocarcinoma (PDAC) tumor tissues, induced *in vivo* the differential expression of 2455 genes.
**Table S5.** MetaCore Enrichment Analysis by Pathway Maps of 1684 genes upregulated in tumor tissue by CSTA (genes downregulated in tumor tissue without CSTA; gene set A).
**Table S6.** MetaCore Enrichment Analysis by Pathway Maps of 771 genes downregulated in tumor tissue by CSTA (genes upregulated in tumor tissue without CSTA; gene set B).
**Table S7.** Geneset Class Comparison Analysis by cell type‐related gene sets.

## Data Availability

All data relevant to the study are included in the article or uploaded as supplementary information.
