# Supplementary figures and images for: Cystatin A promotes the antitumor activity of T helper type 1 cells and dendritic cells in murine models of pancreatic cancer
Source: Mol Oncol. 2025 Jan 10;19(5):1452–70. doi: 10.1002/1878-0261.13796 (PMC12077287; doi:10.1002/1878-0261.13796)

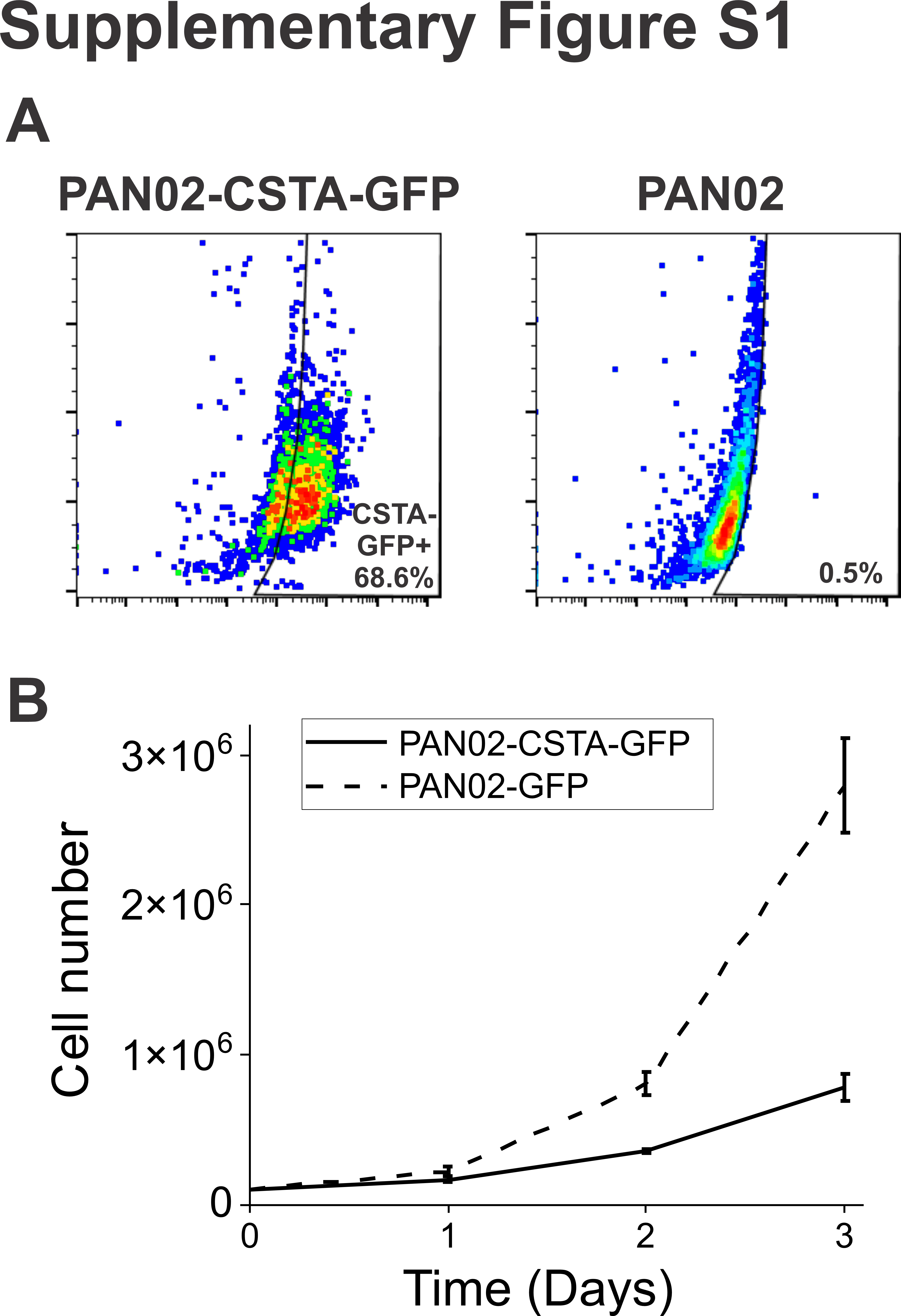

Supplement: Supplementary file 1 — Fig. S1. Establishment of the PAN02‐CSTA‐GFP cell line. [file MOL2-19-1452-s007.tif]

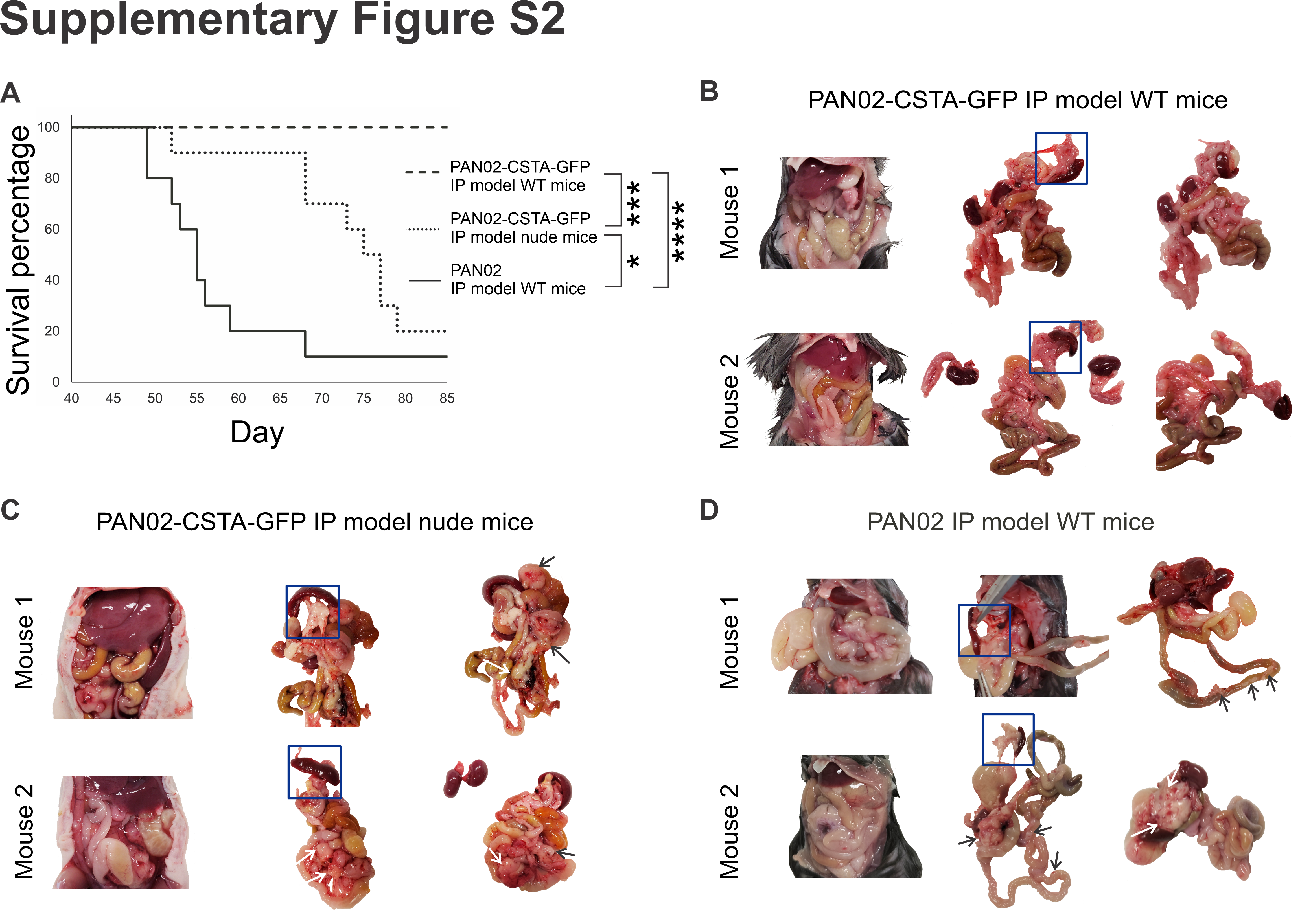

Supplement: Supplementary file 2 — Fig. S2. Intraperitoneal (IP) pancreatic ductal adenocarcinoma (PDAC) model mice established with PAN02‐CSTA‐GFP or PAN02 cell lines. [file MOL2-19-1452-s005.tif]

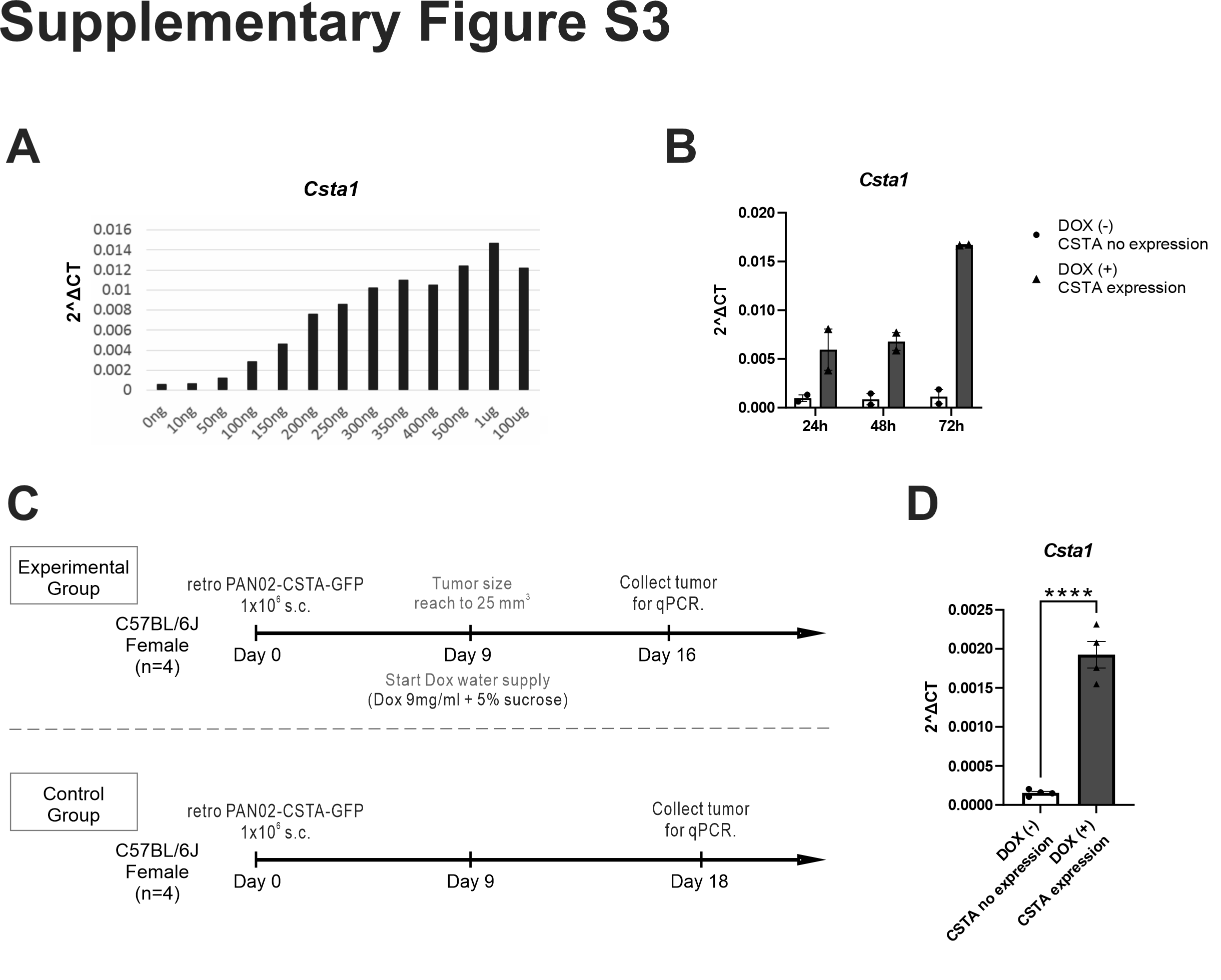

Supplement: Supplementary file 3 — Fig. S3. Establishment of Retro‐PAN02‐CSTA and Retro‐PAN02‐CSTA‐GFP cell lines. [file MOL2-19-1452-s001.tif]

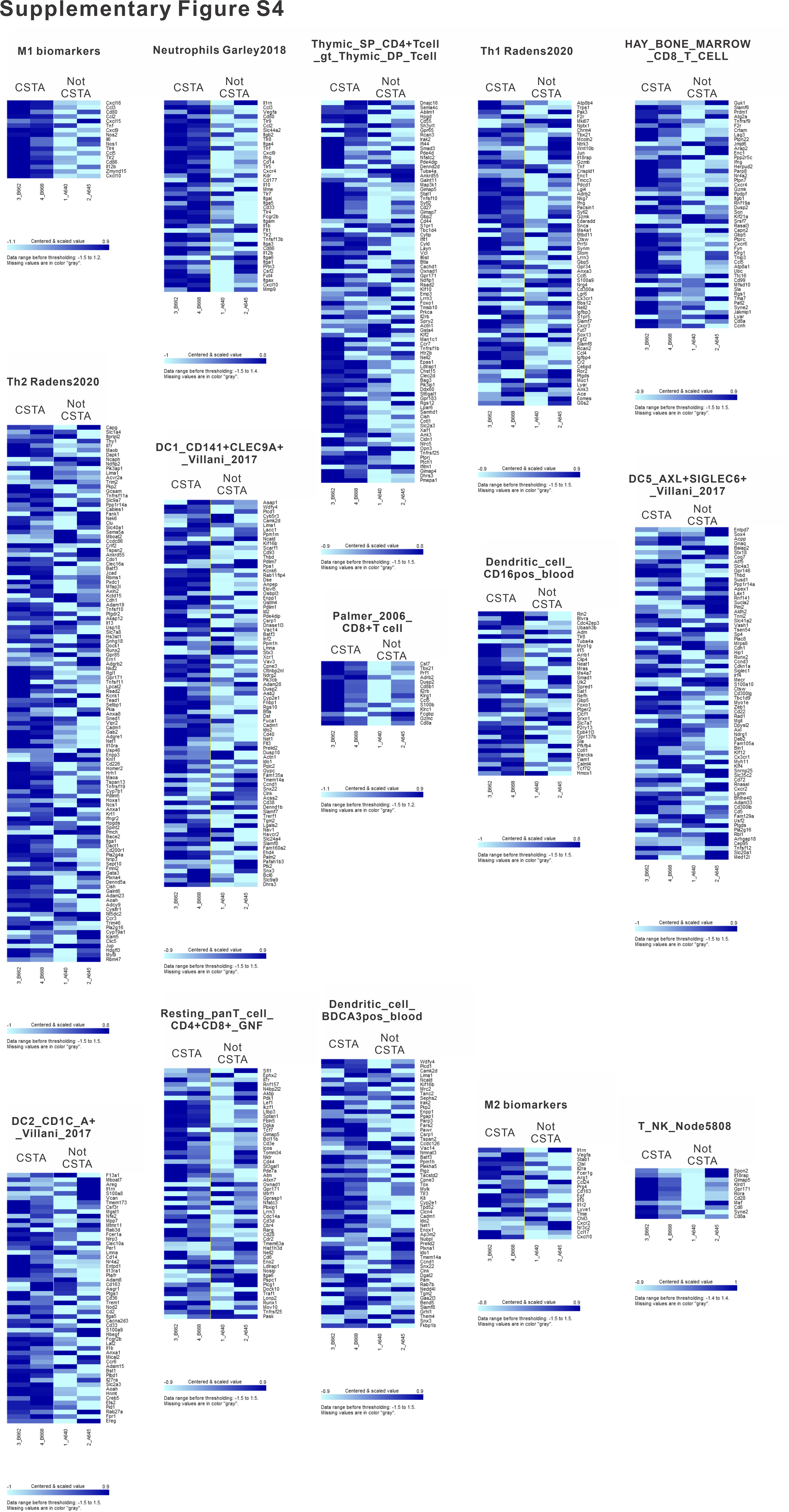

Supplement: Supplementary file 4 — Fig. S4. DNA microarray analysis of PDAC tumor tissues in the presence or not of cystatin A (CSTA) expression. [file MOL2-19-1452-s004.tif]

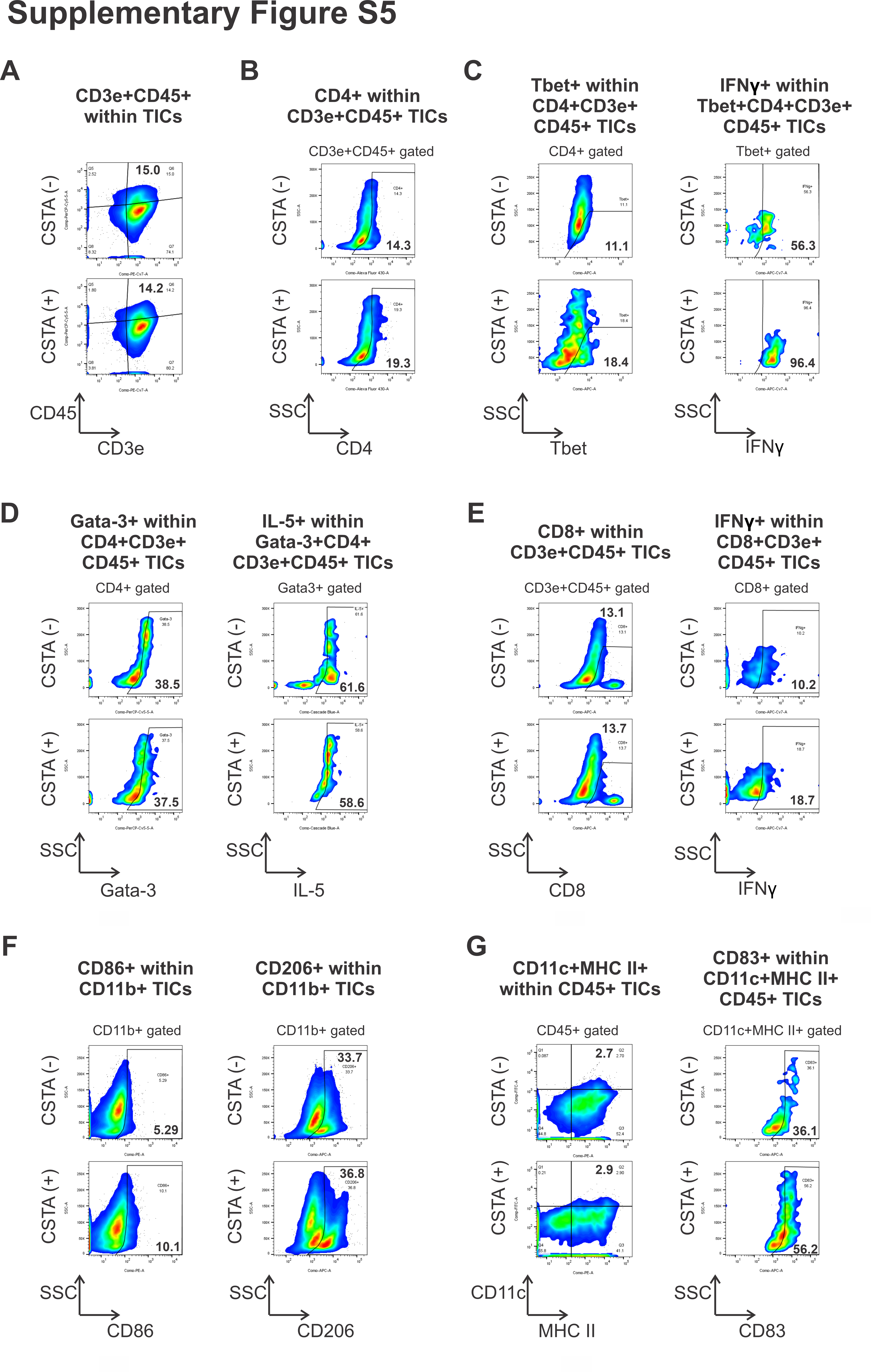

Supplement: Supplementary file 5 — Fig. S5. Flow cytometry (FCM) analysis of lymphoid and myeloid lineage cells in tumor‐infiltrating inflammatory cells. [file MOL2-19-1452-s009.tif]

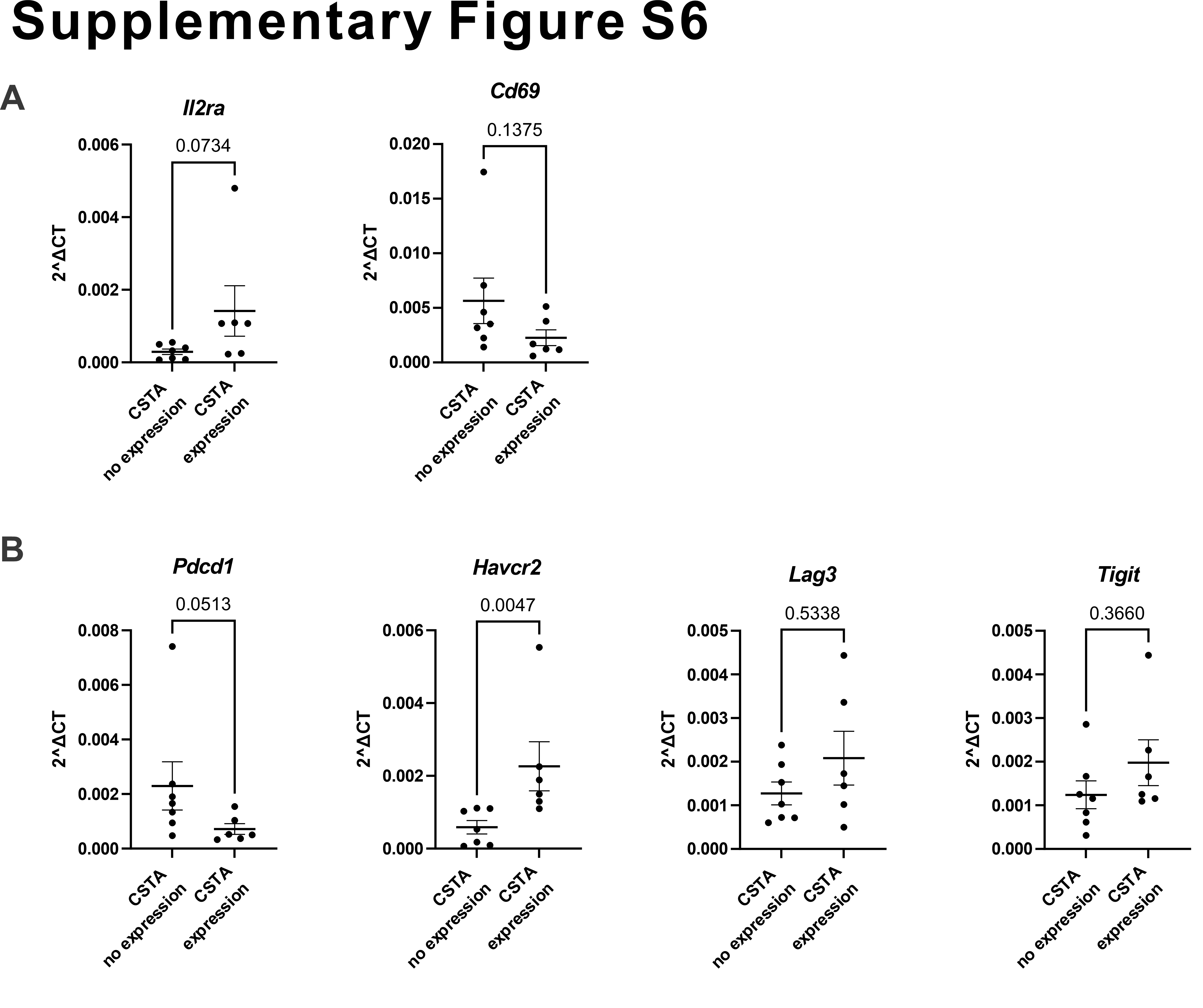

Supplement: Supplementary file 6 — Fig. S6. Gene expression analysis of tumor‐infiltrating inflammatory cells (TICs) from subcutaneous (SC) pancreatic ductal adenocarcinoma (PDAC) model mice by quantitative real‐time PCR (qRT‐PCR). [file MOL2-19-1452-s010.tif]

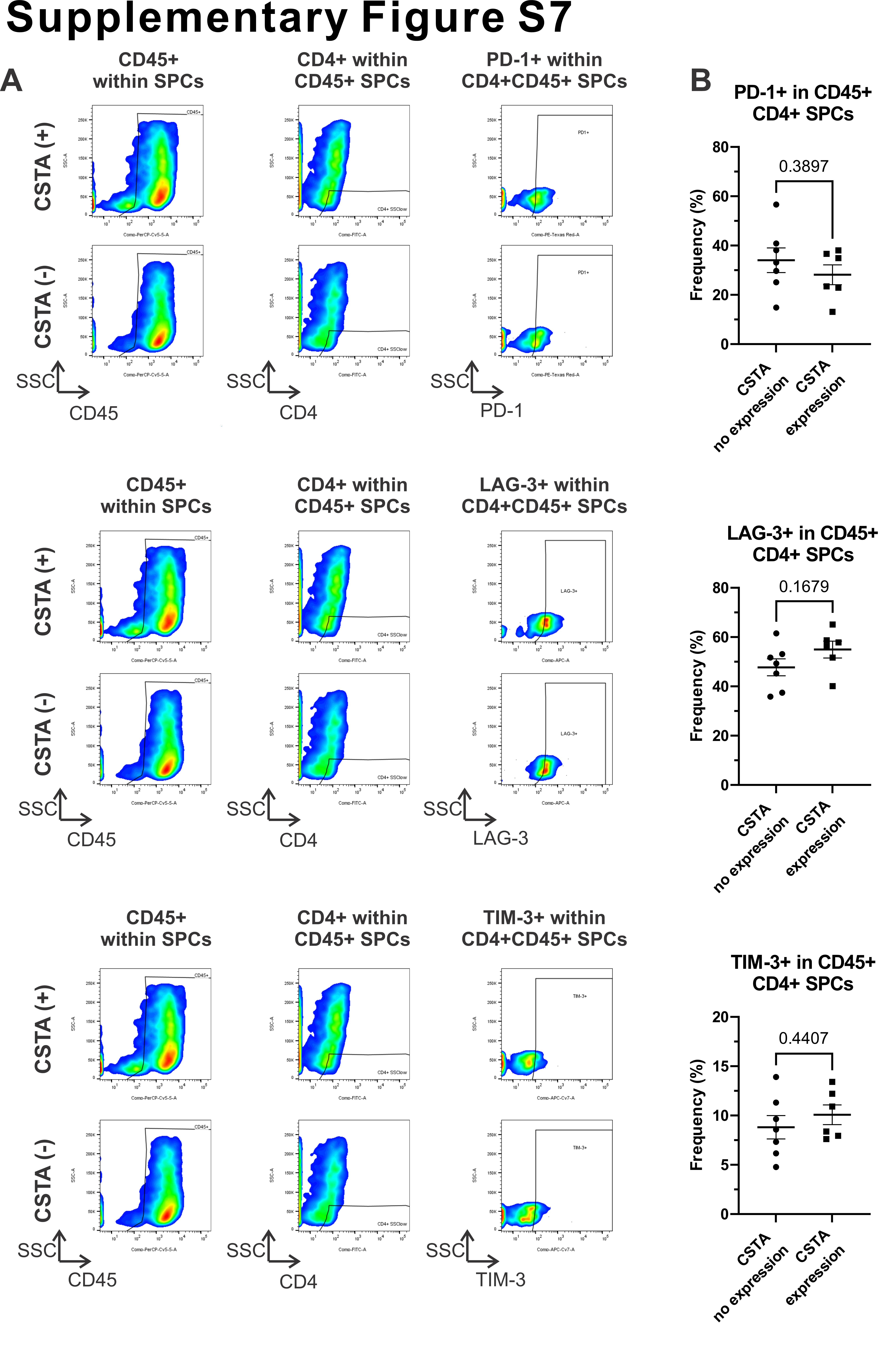

Supplement: Supplementary file 7 — Fig. S7. Flow cytometry (FCM) analysis of splenocytes (SPCs) for CD4+ immune lineage cells and the expression of their immune checkpoint molecules. [file MOL2-19-1452-s003.tif]

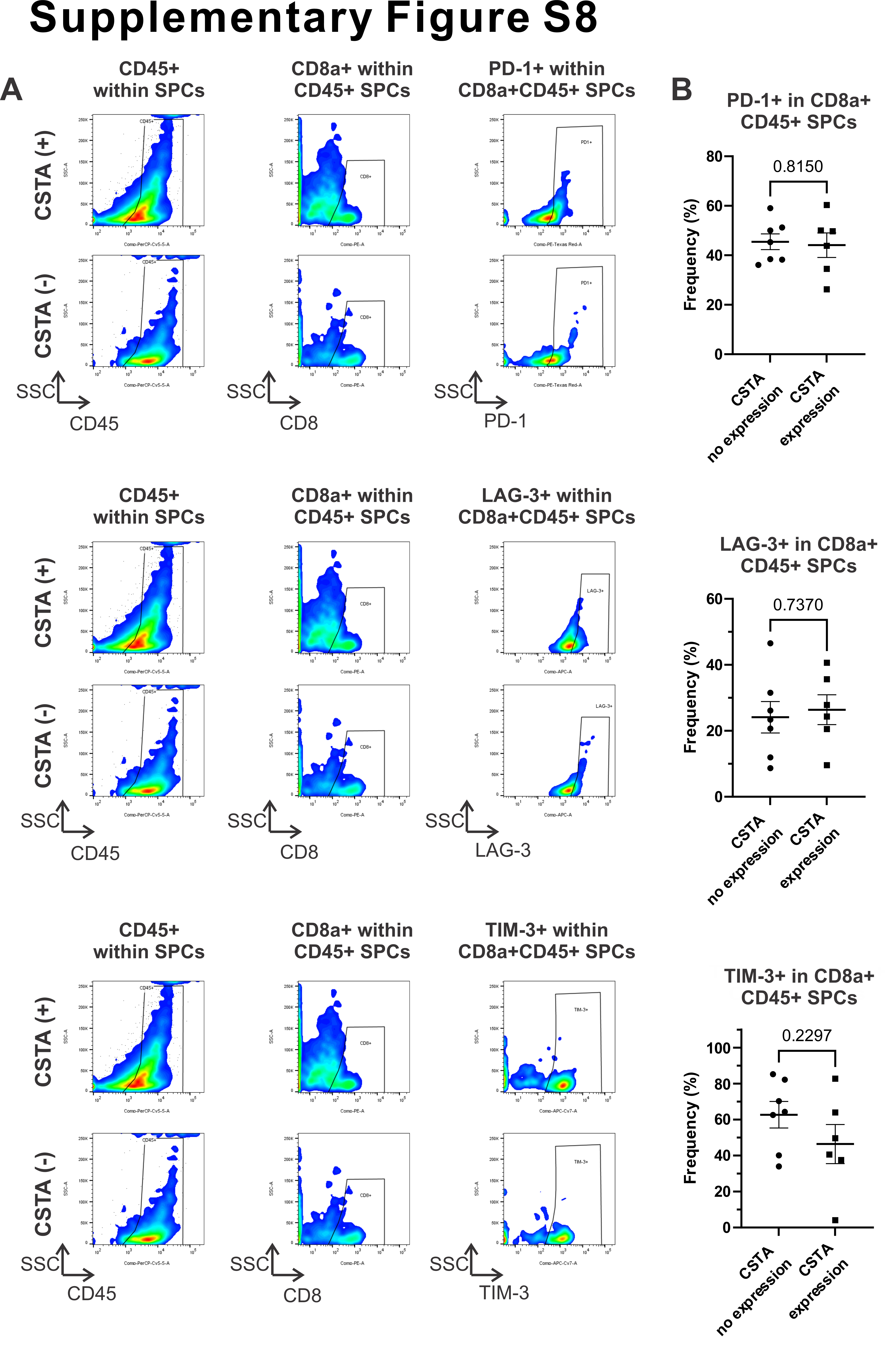

Supplement: Supplementary file 8 — Fig. S8. Flow cytometry (FCM) analysis of splenocytes (SPCs) for CD8+ immune lineage cells and the expression of their immune checkpoint molecules. [file MOL2-19-1452-s011.tif]

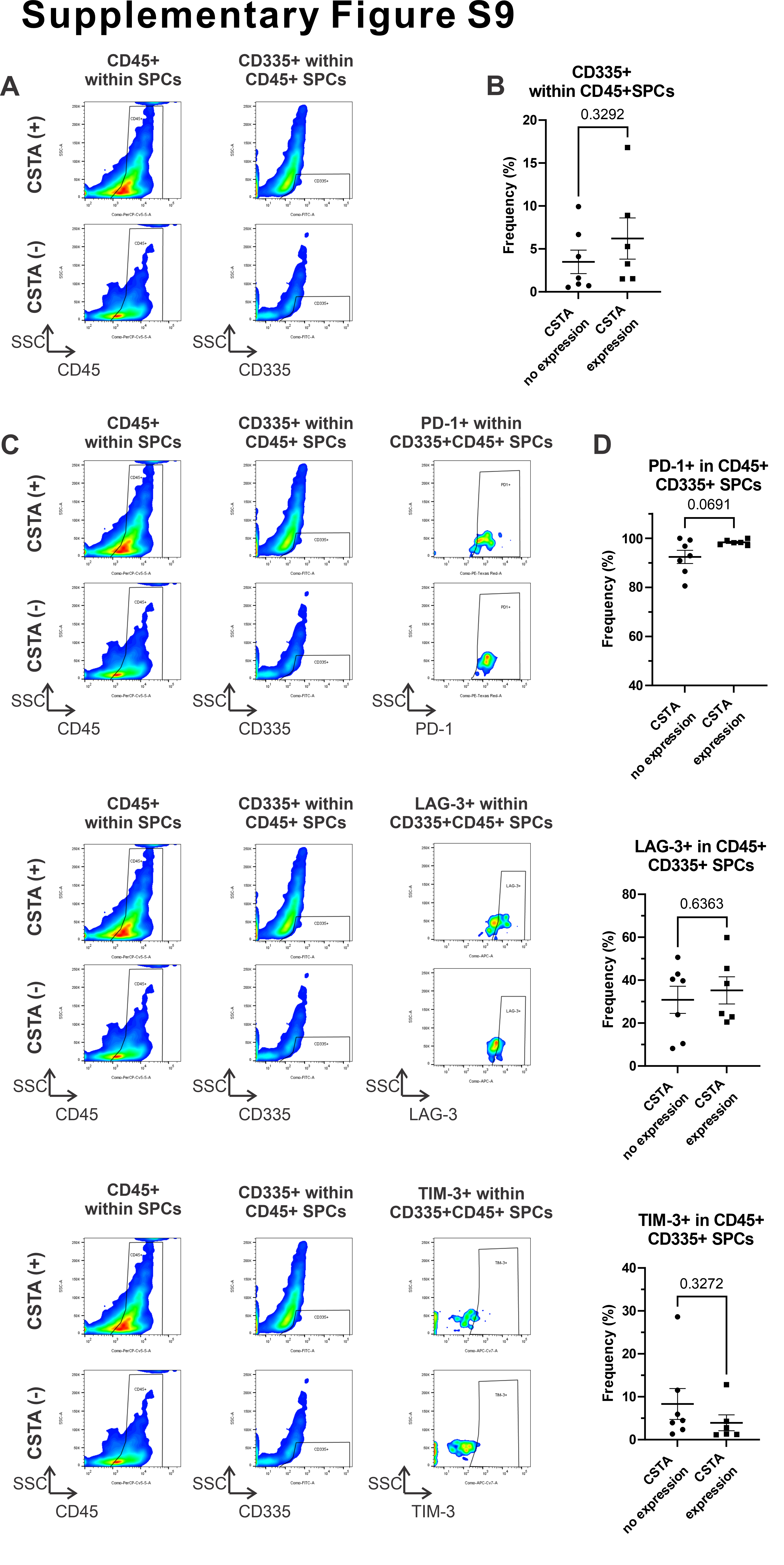

Supplement: Supplementary file 9 — Fig. S9. Flow cytometry (FCM) analysis of splenocytes (SPCs) for CD335+ natural killer (NK) cells and the expression of their immune checkpoint molecules. [file MOL2-19-1452-s006.tif]

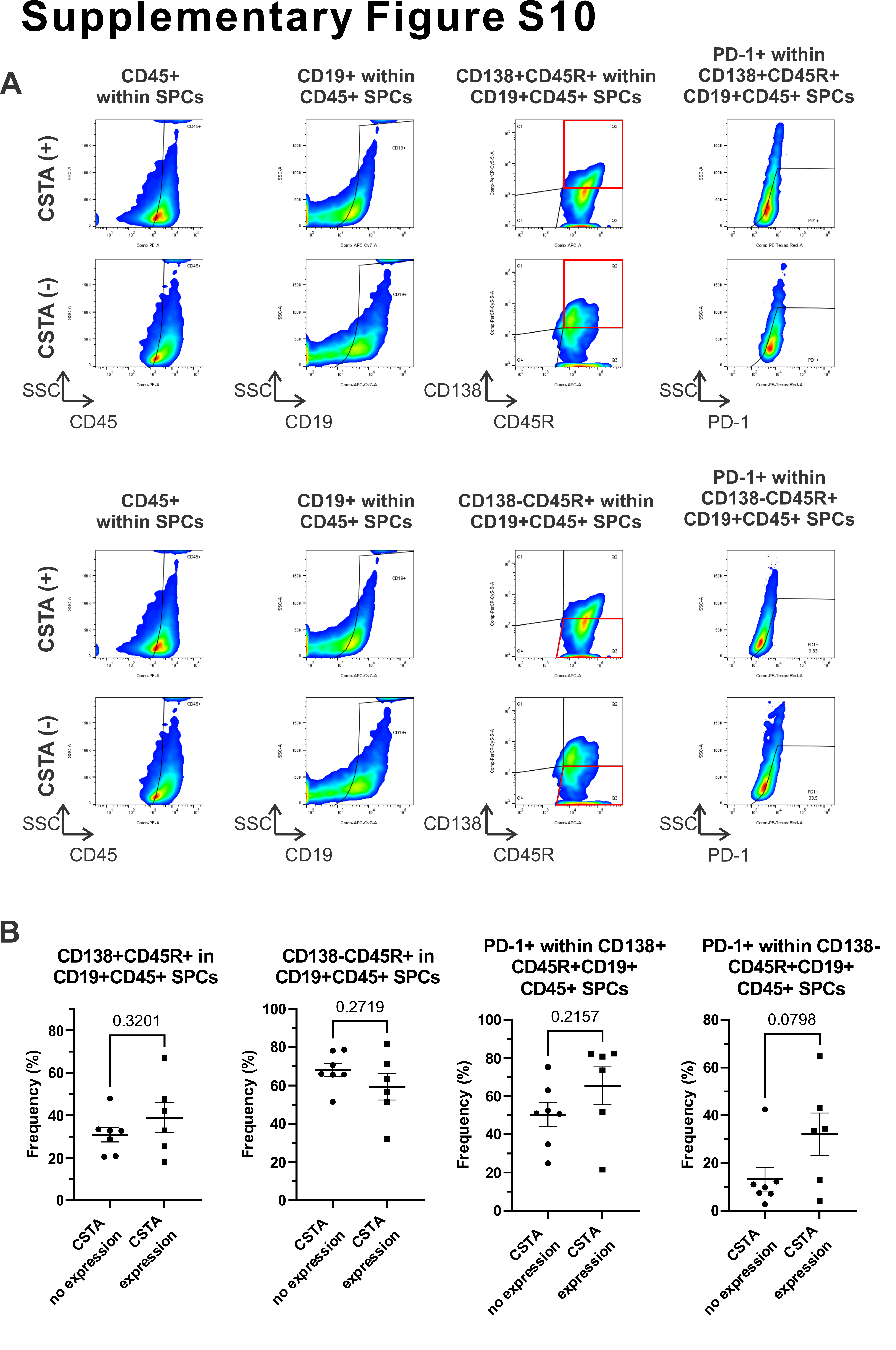

Supplement: Supplementary file 10 — Fig. S10. Flow cytometry (FCM) analysis of splenocytes (SPCs) for B cells, activation status, and PD‐1 immune checkpoint expression. [file MOL2-19-1452-s002.tif]
