# Supplementary material for: Cystatin A promotes the antitumor activity of T helper type 1 cells and dendritic cells in murine models of pancreatic cancer
Source: Mol Oncol. 2025 Jan 10;19(5):1452–70. doi: 10.1002/1878-0261.13796 (PMC12077287; doi:10.1002/1878-0261.13796)
Supplement: Supplementary file 11 — Table S1. Details on the antibodies used in the experiments. Table S2. DAVID Enrichment Analysis of 1204 genes upregulated in the PAN02‐CSTA cell line (genes downregulated in the PAN02 cell line). Table S3. DAVID Enrichment Analysis of 1250 genes downregulated in the PAN02‐CSTA cell line (genes upregulated in the PAN02 cell line). Table S4. CSTA, overexpressed in pancreatic ductal adenocarcinoma (PDAC) tumor tissues, induced in vivo the differential expression of 2455 genes. Table S5. MetaCore Enrichment Analysis by Pathway Maps of 1684 genes upregulated in tumor tissue by CSTA (genes downregulated in tumor tissue without CSTA; gene set A). Table S6. MetaCore Enrichment Analysis by Pathway Maps of 771 genes downregulated in tumor tissue by CSTA (genes upregulated in tumor tissue without CSTA; gene set B). Table S7. Geneset Class Comparison Analysis by cell type‐related gene sets. [file MOL2-19-1452-s008.docx]

**Cystatin A promotes the antitumor activity of T helper type 1 cells and dendritic cells in murine models of pancreatic cancer**

**SUPPLEMENTARY INFORMATION**

**TABLE OF CONTENTS**

[**1.** **Supplementary Tables** 3](#_Toc185439280)

[**Supplementary Table S1.** Details on the antibodies used in the experiments. 3](#_Toc185439281)

[**Supplementary Table S2.** DAVID Enrichment Analysis of 1204 genes upregulated in the PAN02-CSTA cell line (genes downregulated in the PAN02 cell line). 7](#_Toc185439282)

[**Supplementary Table S3.** DAVID Enrichment Analysis of 1250 genes downregulated in the PAN02-CSTA cell line (genes upregulated in the PAN02 cell line). 11](#_Toc185439283)

[**Supplementary Table S4.** *CSTA*, overexpressed in pancreatic ductal adenocarcinoma (PDAC) tumor tissues, induced *in vivo* the differential expression of 2455 genes 16](#_Toc185439284)

[**Supplementary Table S5.** MetaCore Enrichment Analysis by Pathway Maps of 1684 genes upregulated in tumor tissue by CSTA (genes downregulated in tumor tissue without CSTA; gene set A). 60](#_Toc185439285)

[**Supplementary Table S6.** MetaCore Enrichment Analysis by Pathway Maps of 771 genes downregulated in tumor tissue by CSTA (genes upregulated in tumor tissue without CSTA; gene set B). 67](#_Toc185439286)

[**Supplementary Table S7.** Geneset Class Comparison Analysis by cell type-related gene sets 71](#_Toc185439287)

[**2.** **Supplementary References** 72](#_Toc185439288)

[**3.** **Supplementary Figure Legends** 73](#_Toc185439289)

[**Supplementary Figure S1.** Establishment of the PAN02-CSTA-GFP cell line. 73](#_Toc185439290)

[**Supplementary Figure S2.** Intraperitoneal (IP) pancreatic ductal adenocarcinoma (PDAC) model mice established with PAN02-CSTA-GFP or PAN02 cell lines. 73](#_Toc185439291)

[**Supplementary Figure S3.** Establishment of Retro-PAN02-CSTA and Retro-PAN02-CSTA-GFP cell lines. 73](#_Toc185439292)

[**Supplementary Figure S4.** DNA microarray analysis of PDAC tumor tissues in the presence or not of *cystatin A* (*CSTA*) expression. 74](#_Toc185439293)

[**Supplementary Figure S5.** Flow cytometry (FCM) analysis of lymphoid and myeloid lineage cells in tumor-infiltrating inflammatory cells 74](#_Toc185439294)

[**Supplementary Figure S6.** Gene expression analysis of tumor-infiltrating inflammatory cells (TICs) from subcutaneous (SC) pancreatic ductal adenocarcinoma (PDAC) model mice by quantitative real-time PCR (qRT-PCR). 75](#_Toc185439295)

[**Supplementary Figure S7.** Flow cytometry (FCM) analysis of splenocytes (SPCs) for CD4+ immune lineage cells and the expression of their immune checkpoint molecules. 76](#_Toc185439296)

[**Supplementary Figure S8.** Flow cytometry (FCM) analysis of splenocytes (SPCs) for CD8+ immune lineage cells and the expression of their immune checkpoint molecules. 76](#_Toc185439297)

[**Supplementary Figure S9.** Flow cytometry (FCM) analysis of splenocytes (SPCs) for CD335+ natural killer (NK) cells and the expression of their immune checkpoint molecules. 77](#_Toc185439298)

[**Supplementary Figure S10.** Flow cytometry (FCM) analysis of splenocytes (SPCs) for B cells, activation status, and PD-1 immune checkpoint expression. 78](#_Toc185439299)

# **Supplementary Tables**

| **Supplementary Table S1.** Details on the antibodies used in the experiments. | | | | | | | |
| --- | --- | --- | --- | --- | --- | --- | --- |
| **Antibody name** | **RRID** | **Clone** | **Application** | **Vendor** | **Catalog number** | **Headquarter** | **Used in figure/panel** |
| Rat IgG2a isotype control | AB_3644245 | 2A3 | *In vivo* FuncS | Selleck Biotech | A2123 | Yokohama, Japan | 1D |
| Rat IgG2b isotype control | / | LTF-2 | *In vivo* FuncS | Selleck Biotech | A2116 | Yokohama, Japan | 1D |
| Purified NA/LE Rat Anti-Mouse CD4 | / | GK1.5 | *In vivo* FuncS (Blocking/Depletion) | BD Pharmingen | 624095 | San Diego, CA, USA | 1D |
| Purified NA/LE Rat Anti-Mouse CD8a | / | 53-6.7 | *In vivo* FuncS (Blocking/Depletion) | BD Pharmingen | 624095 | San Diego, CA, USA | 1D |
| Purified NA/LE Rat Anti-Mouse Ly-6G and Ly-6C | / | RB6-8C5 | *In vivo* FuncS (Inhibition/Depletion) | BD Pharmingen | 624095 | San Diego, CA, USA | 1D |
| Rat Anti-CD4 Monoclonal Antibody | AB_393574 | H129.19 | IHC-Fr | BD Biosciences | 550278 | Franklin Lakes, NJ, USA | 2E |
| Rat Anti-CD8a Monoclonal Antibody | AB_2275792 | 53-6.7 | IHC-Fr | BD Biosciences | 550281 | Franklin Lakes, NJ, USA | 2E |
| Rat Anti-CD11b Monoclonal Antibody | AB_393577 | M1/70 | IHC-Fr | BD Biosciences | 550282 | Franklin Lakes, NJ, USA | 2E |
| Rat Anti-Ly6g Monoclonal Antibody | AB_470492 | RB6-8C5 | IHC-P | Abcam | ab25377 | Cambridge, United Kingdom | 2E |
| F4/80 Monoclonal Antibody | AB_10376289 | BM8 | IHC-P | Thermo Fisher Scientific | MF48000 | Waltham, MA, USA | 2E |
| T-bet (H-210) antibody | AB_2200557 | H-210 | IHC-P | Santa Cruz Biotechnology | sc-21003 | Dallas, TX, USA | 2F |
| GATA-3 Antibody | / | BLR121H | IHC-P | Novus Biologicals | NBP3-14705 | Littleton, CO, USA | 2F |
| Rat Anti-CD86 Monoclonal Antibody | AB_10902800 | GL-1 | IHC-Fr | Abcam | ab119857 | Cambridge, United Kingdom | 2G |
| Anti-Mannose Receptor (CD206) Polyclonal Antibody | AB_1523910 | / | IHC-P | Abcam | ab64693 | Cambridge, United Kingdom | 2G |
| **Supplementary Table S1.** (Continued) | | | | | | | |
| **Antibody name** | **RRID** | **Clone** | **Application** | **Vendor** | **Catalog number** | **Headquarter** | **Used in figure/panel** |
| CD45 Antibody, anti-mouse, PerCP | AB_2889583 | 30-F11 | FC | Miltenyi Biotec | 130-123-879 | Bergisch Gladbach, Germany | 4A, E; S5A, B, G; S7A, B; S8A, B; S9A-D |
| CD3ε Antibody, anti-mouse, PE-Vio^®^ 770 | AB_2660400 | 145-2C11 | FC | Miltenyi Biotec | 130-102-359 | Bergisch Gladbach, Germany | 4A, B, C; S5A, B, C, D, E |
| CD4 Antibody, anti-mouse, VioGreen™ | AB_2819535 | GK1.5 | FC | Miltenyi Biotec | 130-123-899 | Bergisch Gladbach, Germany | 4A, B; S5B, C, D |
| T-bet Monoclonal Antibody (eBio4B10 (4B10)), APC, eBioscience | AB_2744712 | eBio4B10 (4B10) | ICFC | Thermo Fisher Scientific | 17-5825-82 | Waltham, MA, USA | 4A; S5C |
| IFN gamma Monoclonal Antibody (XMG1.2), Alexa Fluor™ 700, eBioscience | AB_2688063 | XMG1.2 | ICFC | Thermo Fisher Scientific | 56-7311-82 | Waltham, MA, USA | 4A, C; S5C, E |
| CD45 Monoclonal Antibody (30-F11), FITC, eBioscience | AB_465051 | 30-F11 | FC | Thermo Fisher Scientific | 11-0451-85 | Waltham, MA, USA | 4B, C; S5D, E |
| Gata-3 Monoclonal Antibody (TWAJ), PerCP-eFluor™ 710, eBioscience | AB_10805385 | TWAJ | ICFC | Thermo Fisher Scientific | 46-9966-41 | Waltham, MA, USA | 4B; S5D |
| Brilliant Violet 421(TM) anti-mouse/human IL-5 | AB_2563161 | TRFK5 | ICFC | BioLegend | 504311 | San Diego, CA, USA | 4B; S5D |
| APC Rat Anti-Mouse CD8a | AB_398527 | 53-6.7 | FC | BD Biosciences | 553035 | Franklin Lakes, NJ, USA | 4C; 6C; S5E |
| Rat Anti-CD11b Monoclonal Antibody, APC-Cy7 | AB_396772 | M1/70 | FC | BD Biosciences | 557657 | Franklin Lakes, NJ, USA | 4D; S5F |
| PE anti-mouse CD86 Antibody | AB_2819412 | REA1190 | FC | Miltenyi Biotec | 130-122-129 | Bergisch Gladbach, Germany | 4D; S5F |
| **Supplementary Table S1.** (Continued) | | | | | | | |
| **Antibody name** | **RRID** | **Clone** | **Application** | **Vendor** | **Catalog number** | **Headquarter** | **Used in figure/panel** |
| APC anti-mouse CD206 (MMR) Antibody | AB_10900231 | C068C2 | FC | BioLegend | 141708 | San Diego, CA, USA | 4D; S5F |
| Armenian Hamster Anti-CD11c Monoclonal Antibody, FITC Conjugated | AB_395060 | HL3 | FC | BD Biosciences | 553801 | Franklin Lakes, NJ, USA | 4E; 6E; S5G |
| MHC Class II Antibody, anti-mouse, PE | AB_2660056 | M5/114.15.2 | FC | Miltenyi Biotec | 130-102-186 | Bergisch Gladbach, Germany | 4E; 6E; S5G |
| CD83 Antibody, anti-mouse, APC, REAfinity™ | AB_2659311 | REA304 | FC | Miltenyi Biotec | 130-104-475 | Bergisch Gladbach, Germany | 4E; S5G |
| PE anti-mouse CD45 Antibody | AB_312971 | 30-F11 | FC | BioLegend | 103106 | San Diego, CA, USA | 5B, 5D, E; S10A, B |
| FITC Rat Anti-Mouse CD4 | AB_394582 | RM4.5 | FC | BD Biosciences | 553047 | Franklin Lakes, NJ, USA | 6C |
| APC Rat Anti-CD11b | AB_398535 | M1/70 | FC | BD Biosciences | 553312 | Franklin Lakes, NJ, USA | 6D |
| PE Rat Anti-Mouse Ly-6G | AB_394208 | 1A8 | FC | BD Biosciences | 551461 | Franklin Lakes, NJ, USA | 6D |
| Cell Lab FITC Rat anti-mouse CD4 | / | GK1.5 | FC | Beckman Coulter | 732000 | Indianapolis, IN, USA | S7A, B |
| PE/Dazzle™ 594 anti-mouse CD279 (PD-1) Antibody | AB_2566006 | 29F.1A12 | FC | BioLegend | 135228 | San Diego, CA, USA | S7A, B; S8A, B; S9C, D; S10A, B |
| (LAG-3) CD223 Antibody, anti-mouse, APC, REAfinity™ | AB_2656415 | REA776 | FC | Miltenyi Biotec | 130-111-328 | Bergisch Gladbach, Germany | S7A, B; S8A, B; S9C, D |
| TIM-3 Antibody, anti-mouse, APC-Vio^®^ 770, REAfinity™ | AB_2922208 | REA602 | FC | Miltenyi Biotec | 130-130-038 | Bergisch Gladbach, Germany | S7A, B; S8A, B; S9C, D |
| PE Rat Anti-Mouse CD8a | AB_394570 | 53-6.7 | FC | BD Biosciences | 553032 | Franklin Lakes, NJ, USA | S8A, B |
| **Supplementary Table S1.** (Continued) | | | | | | | |
| **Antibody name** | **RRID** | **Clone** |  | **Vendor** | **Catalog number** | **Headquarter** | **Used in figure/panel** |
| CD335 (NKp46) Antibody, anti-mouse, FITC, REAfinity™ | AB_2657601 | REA815 | FC | Miltenyi Biotec | 130-112-200 | Bergisch Gladbach, Germany | S9A-D |
| APC/Cyanine7 anti-mouse CD19 Antibody | AB_830707 | 6D5 | FC | BioLegend | 115530 | San Diego, CA, USA | S10A, B |
| Alexa Fluor^®^ 647 Rat Anti-Mouse CD45R | AB_396793 | RA3-6B2 | FC | BD Pharmingen | 557683 | San Diego, CA, USA | S10A, B |
| PerCP/Cyanine5.5 anti-mouse CD138 (Syndecan-1) Antibody | AB_2561601 | 281-2 | FC | BioLegend | 142510 | San Diego, CA, USA | S10A, B |
| RRID, Research Resource Identifier; FuncS, Functional Assay; IHC-P, immunohistochemistry (paraffin-embedded); IHC-Fr, immunohistochemistry (frozen sections); FC, flow cytometry; ICFC, intracellular staining for flow cytometry. | | | | | | | |

| **Supplementary Table S2.** DAVID Enrichment Analysis of 1204 genes upregulated in the PAN02-CSTA cell line (genes downregulated in the PAN02 cell line). Annotation clusters were identified based on shared identified genes among terms. | | | | | |
| --- | --- | --- | --- | --- | --- |
| **Category** | **Term** | **Count** | ***P* Value** | **Genes** | **Annotation Cluster related to:** |
| UP_SEQ_FEATURE | DOMAIN:C2 | 17 | 3.38E-04 | *CPNE9, UNC13C, CADPS2, SYT3, MCTP1, PRF1, DYSF, NEDD4L, SYT14, PIK3C2G, TC2N, SYT7, PLCB4, HECW2, PRKCQ, PLCH1, C2CD2L* | C2 domain |
| SUPFAM | SSF49562:C2 domain (Calcium/lipid-binding domain, CaLB) | 18 | 5.72E-04 | *CPNE9, UNC13C, CADPS2, SYT3, MCTP1, PRF1, DYSF, NEDD4L, PIK3CD, SYT14, PIK3C2G, TC2N, SYT7, PLCB4, HECW2, PRKCQ, PLCH1, C2CD2L* |  |
| INTERPRO | IPR000008:C2 calcium-dependent membrane targeting | 17 | 6.95E-04 | *CPNE9, UNC13C, CADPS2, SYT3, MCTP1, PRF1, DYSF, NEDD4L, SYT14, PIK3C2G, TC2N, SYT7, PLCB4, HECW2, PRKCQ, PLCH1, C2CD2L* |  |
| PROSITE | PS50004:PS50004 | 17 | 0.001207 | *CPNE9, UNC13C, CADPS2, SYT3, MCTP1, PRF1, DYSF, NEDD4L, SYT14, PIK3C2G, TC2N, SYT7, PLCB4, HECW2, PRKCQ, PLCH1, C2CD2L* |  |
| SMART | SM00239:C2 | 14 | 0.005087 | *CPNE9, UNC13C, SYT3, MCTP1, PRF1, DYSF, NEDD4L, SYT14, PIK3C2G, TC2N, SYT7, PLCB4, HECW2, PLCH1* |  |
| PFAM | PF00168:C2 domain | 14 | 0.00552 | *CPNE9, UNC13C, SYT3, MCTP1, PRF1, DYSF, NEDD4L, SYT14, PIK3C2G, TC2N, SYT7, PLCB4, HECW2, PLCH1* |  |
| GENE3D | 2.60.40.150:C2- domain Calcium/lipid binding domain | 16 | 0.007914 | *CPNE9, UNC13C, SYT3, MCTP1, PRF1, DYSF, NEDD4L, PIK3CD, SYT14, PIK3C2G, TC2N, SYT7, PLCB4, HECW2, PRKCQ, PLCH1* |  |
| GOTERM_CC_DIRECT | GO:0001533~cornified envelope | 13 | 4.14E-05 | *CSTA2, SERPINB12, CSTA1, SPRR2H, PPL, CST6, STFA3, CYSRT1, SPRR2A1, CSTDC4, SPRR1A, SPRR1B, CSTDC5* | Cystatin A, and negative regulation of peptidase regulation |
| GOTERM_BP_DIRECT | GO:0010466~negative regulation of peptidase activity | 16 | 6.99E-04 | *SERPINA11, CSTA2, SERPINB12, CSTA1, SERPINF2, SERPINA3N, NGF, CST7, STFA3, PBP2, SERPINA1D, COL7A1, CD109, CSTDC4, RECK, CSTDC5* |  |
| UP_SEQ_FEATURE | DOMAIN:Cystatin | 6 | 7.08E-04 | *CSTA2, CSTA1, CST7, CSTDC4, CST6, CSTDC5* |  |
| **Supplementary Table S2.** (Continued) | | | | | |
| **Category** | **Term** | **Count** | ***P* Value** | **Genes** | **Annotation Cluster related to:** |
| SMART | SM00043:CY | 7 | 9.08E-04 | *CSTA2, CSTA1, CST7, CSTDC4, CST6, CSTDC5, STFA3* | Cystatin A, and negative regulation of peptidase regulation |
| PFAM | PF00031:Cystatin domain | 7 | 0.001082 | *CSTA2, CSTA1, CST7, CSTDC4, CST6, CSTDC5, STFA3* |  |
| INTERPRO | IPR001713:Proteinase inhibitor I25A, stefin A | 5 | 0.001489 | *CSTA2, CSTA1, CSTDC4, CSTDC5, STFA3* |  |
| INTERPRO | IPR000010:Proteinase inhibitor I25, cystatin | 7 | 0.001566 | *CSTA2, CSTA1, CST7, CSTDC4, CST6, CSTDC5, STFA3* |  |
| GOTERM_CC_DIRECT | GO:1904090~peptidase inhibitor complex | 5 | 0.002899 | *CSTA2, CSTA1, CSTDC4, CSTDC5, STFA3* |  |
| KEGG_PATHWAY | mmu00830:Retinol metabolism | 13 | 0.001421 | *CYP4A12A, UGT2B34, UGT2B37, CYP3A44, CYP26B1, CYP2C23, CYP2A12, ALDH1A2, CYP3A41A, RDH10, ALDH1A1, RDH13, ALDH1A7* | Retinol metabolism |
| WIKIPATHWAYS | WP1259~Retinol metabolism | 8 | 0.004411 | *ABCG5, CYP26B1, NPC1L1, ALDH1A2, RDH10, ALDH1A1, LPL, BCO2* |  |
| GOTERM_BP_DIRECT | GO:0042573~retinoic acid metabolic process | 6 | 0.006239 | *CYP26B1, ALDH1A2, CYP3A41A, ALDH1A1, CYP3A44, BCO2* |  |
| GOTERM_CC_DIRECT | GO:0070161~anchoring junction | 31 | 0.002626 | *CRB1, ITGB3, SYNPO2, LIN7A, PPL, DPP4, BAIAP2L2, GJA1, CLMP, MARVELD2, EPCAM, XIRP1, GJA6, XIRP2, PGM5, WASF1, JAM2, HAVCR2, EPHA4, FYB1, FRMD4B, SLC8A3, ARHGAP31, OCLN, GJB4, CLDN8, GJB5, CLDN19, CLDN18, LCP1, AMOTL1* | Cell junctions |
| UP_KW_CELLULAR_COMPONENT | KW-0965~Cell junction | 32 | 0.005758 | *CRB1, ITGB3, SYNPO2, LIN7A, PPL, DPP4, BAIAP2L2, GJA1, CLMP, MARVELD2, EPCAM, CLDN34C1, XIRP1, GJA6, XIRP2, PGM5, WASF1, JAM2, HAVCR2, EPHA4, FYB1, FRMD4B, SLC8A3, ARHGAP31, OCLN, GJB4, CLDN8, GJB5, CLDN19, CLDN18, LCP1, AMOTL1* |  |
| **Supplementary Table S2.** (Continued) | | | | | |
| **Category** | **Term** | **Count** | ***P* Value** | **Genes** | **Annotation Cluster related to:** |
| UP_KW_CELLULAR_COMPONENT | KW-0796~Tight junction | 12 | 0.016477 | *OCLN, CLMP, MARVELD2, EPCAM, CLDN8, CLDN34C1, CLDN19, CLDN18, LIN7A, FRMD4B, AMOTL1, JAM2* | Cell junctions |
| GOTERM_CC_DIRECT | GO:0005923~bicellular tight junction | 14 | 0.017017 | *LIM2, LIN7A, FRMD4B, OCLN, CLMP, MARVELD2, EPCAM, CLDN8, CLDN34C1, WNK3, CLDN19, CLDN18, AMOTL1, JAM2* |  |
| GOTERM_BP_DIRECT | GO:0007154~cell communication | 8 | 0.001519 | *SLC8A3, GJA1, GJB4, GJB5, GJA6, ENPP5, GJE1, SLC8A1* |  |
| GOTERM_BP_DIRECT | GO:0007267~cell-cell signaling | 10 | 0.009956 | *GJA1, GJB4, GPNMB, PTH, CCL5, GJB5, CCL3, WNT7A, GJA6, GJE1* |  |
| INTERPRO | IPR013092:Connexin, N-terminal | 5 | 0.010955 | *GJA1, GJB4, GJB5, GJA6, GJE1* |  |
| INTERPRO | IPR019570:Gap junction protein, cysteine-rich domain | 5 | 0.010955 | *GJA1, GJB4, GJB5, GJA6, GJE1* |  |
| GOTERM_BP_DIRECT | GO:0006527~arginine catabolic process | 5 | 1.24E-04 | *DDAH1, NOS2, NOS3, NOS1, FAH* | Reactive oxygen species (ROS) and reactive nitrogen species (RNS) |
| REACTOME_PATHWAY | R-MMU-392154~Nitric oxide stimulates guanylate cyclase | 5 | 0.002719 | *NOS2, NOS3, PDE2A, NOS1, PDE9A* |  |
| SUPFAM | SSF56512:Nitric oxide (NO) synthase oxygenase domain | 3 | 0.011293 | *NOS2, NOS3, NOS1* |  |
| INTERPRO | IPR004030:Nitric oxide synthase, oxygenase domain | 3 | 0.011346 | *NOS2, NOS3, NOS1* |  |
| KEGG_PATHWAY | mmu00220:Arginine biosynthesis | 5 | 0.011412 | *NOS2, ARG1, NOS3, NAGS, NOS1* |  |
| REACTOME_PATHWAY | R-MMU-1222556~ROS and RNS production in phagocytes | 6 | 0.02827 | *NOS2, NOS3, RAC2, CYBB, ATP6V0A4, NOS1* |  |
| **Supplementary Table S2.** (Continued) | | | | | |
| **Category** | **Term** | **Count** | ***P* Value** | **Genes** | **Annotation Cluster related to:** |
| INTERPRO | IPR000827:CC chemokine, conserved site | 4 | 0.032519 | *CCL9, CCL5, CCL3, CCL1* | Chemokines |
| PROSITE | PS00472:PS00472 | 4 | 0.037322 | *CCL9, CCL5, CCL3, CCL1* |  |
| GOTERM_MF_DIRECT | GO:0031726~CCR1 chemokine receptor binding | 3 | 0.037911 | *CCL9, CCL5, CCL3* |  |
| GOTERM_BP_DIRECT | GO:0048247~lymphocyte chemotaxis | 5 | 0.047683 | *CCL9, CCL5, CCL3, SAA2, CCL1* |  |

| **Supplementary Table S3.** DAVID Enrichment Analysis of 1250 genes downregulated in the PAN02-CSTA cell line (genes upregulated in the PAN02 cell line). Annotation clusters were identified based on shared identified genes among terms. | | | | | |
| --- | --- | --- | --- | --- | --- |
| **Category** | **Term** | **Count** | ***P* Value** | **Genes** | **Annotation Cluster related to:** |
| GOTERM_BP_DIRECT | GO:0045893~positive regulation of transcription, DNA-templated | 60 | 5.62E-05 | *CALCOCO1, RORC, TRP63, SCX, SIX1, RORA, BMYC, CHD3, AHR, FGF2, GLI3, ETS2, HOXA11, CDH1, EOMES, KCNH2, MITF, PAX6, FOS, ARAP1, GREM1, TAL1, DDIT3, IL1B, IRF1, KAT6A, IRF7, IRF6, PPARA, ZFPM2, IRF9, DLX5, EPAS1, PBXIP1, PRDM11, FOXO1, LRP6, PDLIM1, RHOX5, T, NFIL3, ATOH8, TNNI2, HES1, ZFP711, JUN, EYA1, TGFB3, STAT3, GDF2, KLF4, TRERF1, AGT, COL1A1, NR4A2, NR4A1, IL6, ID2, BCL3, SNAI1* | Regulation of transcription |
| GOTERM_MF_DIRECT | GO:0043565~sequence-specific DNA binding | 46 | 6.78E-05 | *CALCOCO1, CEBPD, DLX4, EPAS1, RORC, SCX, FOXO6, TRP63, SIX1, RORA, AHR, ASCL1, MEOX1, HOXA13, ETS2, FOXO1, GLI3, T, HEY1, SIX3, HESX1, MAEL, HES1, ZFP711, EOMES, JUN, NKX6-2, ESX1, STAT3, ARID5A, PAX6, ZIC4, MITF, FOS, KLF4, POU6F1, NR4A2, NR4A1, MEIS1, DDIT3, IRF1, SNAI1, FOSB, RARB, IRF6, PPARA* |  |
| GOTERM_BP_DIRECT | GO:0045944~positive regulation of transcription from RNA polymerase II promoter | 88 | 2.00E-04 | *CALCOCO1, MAML2, ONECUT2, MYT1L, ONECUT1, SLC40A1, TRP63, SCX, SIX1, RORA, ARRB1, BMYC, AHR, MEOX1, HOXA13, FGF2, GLI3, ETS2, CX3CL1, DBP, HEY1, SIX3, JAK3, CDK5RAP3, EOMES, OLIG3, ARID5A, MITF, PAX6, FOS, RGMA, GREM1, TFR2, TAL1, DDIT3, IL1B, IRF1, KAT6A, IRF7, RARB, IRF6, PPARA, ZFPM2, ATF3, IRF9, TLR2, HDAC5, NOTCH3, AKNA, CEBPD, TNKS, DLX5, EPAS1, AGAP2, ASCL1, DLL1, FOXO1, LRP6, T, ATOH8, HES1, DRD2, ZFP711, TCF7L1, JUN, IFI204, EYA1, TGFB3, CAVIN4, PRRX2, STAT3, GDF2, RFXANK, KLF4, TRERF1, ELL3, NR4A2, NR4A1, PER1, IL6, MEIS1, RGCC, CCPG1, BCL3, CD28, FOSB, PF4, RIGI* |  |
| GOTERM_MF_DIRECT | GO:1990837~sequence-specific double-stranded DNA binding | 48 | 2.70E-04 | *CEBPD, DLX4, ONECUT2, DLX5, ZFP647, ONECUT1, RORC, SIX1, TFCP2L1, AHR, ASCL1, MEOX1, HOXA13, ETS2, GLI3, BACH2, HOXA11, DBP, T, HEY1, SIX3, HESX1, HES1, EOMES, JUN, TCF7L1, NKX6-2, OLIG3, ZFP1, ESX1, PRRX2, PAX6, ZIC4, FOS, KLF4, VAX1, POU6F1, NR4A2, NR4A1, SNAI1, IRF7, FOSB, RARB, IRF6, ZFP454, ATF3, IRF9, JDP2* |  |
| **Supplementary Table S3.** (Continued) | | | | |  |
| **Category** | **Term** | **Count** | ***P* Value** | **Genes** | **Annotation Cluster related to:** |
| GOTERM_MF_DIRECT | GO:0003700~transcription factor activity, sequence-specific DNA binding | 53 | 4.63E-04 | *ONECUT2, MYT1L, ONECUT1, RORC, TRP63, SIX1, RORA, BMYC, TFCP2L1, AHR, MEOX1, HOXA13, GLI3, ETS2, BACH2, DBP, HEY1, SOX5, EOMES, ZHX3, NKX6-2, MITF, PAX6, FOS, ARAP1, TAL1, DDIT3, IRF1, IRF7, RARB, IRF6, PPARA, ATF3, IRF9, JDP2, CEBPD, EPAS1, FOXO6, ASCL1, FOXO1, T, NFIL3, ATOH8, ZFP711, TCF7L1, JUN, STAT3, VAX1, KLF4, POU6F1, NR4A2, NR4A1, FOSB* | Regulation of transcription |
| GOTERM_MF_DIRECT | GO:0000978~RNA polymerase II core promoter proximal region sequence-specific DNA binding | 80 | 0.00316896 | *ZFP128, CALCOCO1, ONECUT2, MYT1L, ZFP647, ONECUT1, RORC, TRP63, SCX, SIX1, RORA, BMYC, TFCP2L1, MEOX1, HOXA13, GLI3, ETS2, BACH2, HOXA11, DBP, HEY1, SIX3, NKX1-2, SOX5, ZFP975, EOMES, NKX6-2, OLIG3, ZFP418, ZFP1, ZFP3, ZFP735, MITF, PAX6, FOS, TAL1, DDIT3, IRF1, IRF7, RARB, IRF6, ZFP454, PPARA, ATF3, IRF9, JDP2, ZFP941, HDAC5, CEBPD, DLX4, DLX5, EPAS1, FOXO6, ASCL1, FOXO1, T, NFIL3, ATOH8, HESX1, HES1, ZKSCAN14, ZFP711, TCF7L1, JUN, IFI204, PRRX2, STAT3, ZIC4, VAX1, KLF4, POU6F1, NR4A2, PER2, NR4A1, PER1, MEIS1, SNAI1, FOSB, MXD4, ZFP352* |  |
| PFAM | PF00048:Small cytokines (intecrine/chemokine), interleukin-8 like | 11 | 2.05E-05 | *CXCL12, CCL7, CCL6, CCL2, CXCL1, CXCL2, CCL17, CXCL5, CX3CL1, CCL21A, PF4* | Interleukin 8 |
| INTERPRO | IPR001811:Chemokine interleukin-8-like domain | 11 | 2.15E-05 | *CXCL12, CCL7, CCL6, CCL2, CXCL1, CXCL2, CCL17, CXCL5, CX3CL1, CCL21A, PF4* |  |
| **Supplementary Table S3.** (Continued) | | | | |  |
| **Category** | **Term** | **Count** | ***P* Value** | **Genes** | **Annotation Cluster related to:** |
| INTERPRO | IPR009030:Insulin-like growth factor binding protein, N-terminal | 19 | 6.36E-05 | *NOTCH3, EPHA7, MEGF6, EGFEM1, HTRA4, FBLN1, PCSK6, DLL1, KAZALD1, ELAPOR1, NELL1, STAB1, CCN5, HMCN2, MUC13, CCN2, SLIT2, CRELD1, SCARF2* | Epidermal growth factor (EGF) |
| INTERPRO | IPR001881:EGF-like calcium-binding | 18 | 1.05E-04 | *FBLN7, NOTCH3, EGFL7, MEGF6, C1RA, EGFEM1, FBLN1, THBS1, HSPG2, DLL1, BCAN, NELL1, C1S1, STAB1, HMCN2, SLIT2, CRELD1, EDIL3* |  |
| UP_KW_DOMAIN | KW-0245~EGF-like domain | 28 | 2.47E-04 | *FBLN7, NOTCH3, MEGF6, ITGB5, FBLN1, CLEC14A, THBS1, DLL1, LRP6, C1S1, CNTNAP5A, STAB1, HMCN2, ITGB8, MUC13, SLIT2, SCARF2, EDIL3, NTNG1, EGFL7, C1RA, EGFEM1, HSPG2, ADAM19, BCAN, NELL1, ADAM26A, CRELD1* |  |
| SMART | SM00179:EGF_CA | 18 | 6.97E-04 | *FBLN7, NOTCH3, EGFL7, MEGF6, C1RA, EGFEM1, FBLN1, THBS1, HSPG2, DLL1, BCAN, NELL1, C1S1, STAB1, HMCN2, SLIT2, CRELD1, EDIL3* |  |
| INTERPRO | IPR018097:EGF-like calcium-binding, conserved site | 14 | 7.03E-04 | *FBLN7, NOTCH3, EGFL7, MEGF6, C1RA, EGFEM1, FBLN1, DLL1, NELL1, C1S1, HMCN2, SLIT2, CRELD1, EDIL3* |  |
| REACTOME_PATHWAY | R-MMU-8948216~Collagen chain trimerization | 10 | 6.38E-05 | *COL1A1, COL3A1, COL16A1, COL14A1, COL25A1, COL6A2, COL12A1, COL6A1, COL8A2, COL19A1* | Extracellular matrix (ECM) organization |
| GOTERM_MF_DIRECT | GO:0030020~extracellular matrix structural constituent conferring tensile strength | 10 | 3.01E-04 | *COL1A1, COL3A1, COL16A1, COL14A1, COL25A1, COL6A2, COL12A1, COL6A1, COL8A2, COL19A1* |  |
| GOTERM_CC_DIRECT | GO:0005581~collagen trimer | 13 | 4.06E-04 | *COL16A1, COL14A1, COL25A1, COL12A1, COL19A1, COL1A1, COL3A1, C1QTNF1, COL6A2, C1QTNF6, COL6A1, COL8A2, EMILIN2* |  |
| **Supplementary Table S3.** (Continued) | | | | | |
| **Category** | **Term** | **Count** | ***P* Value** | **Genes** | **Annotation Cluster related to:** |
| REACTOME_PATHWAY | R-MMU-1474244~Extracellular matrix organization | 26 | 5.00E-04 | *SPARC, COL16A1, ITGB5, COL14A1, COL12A1, ICAM2, FGF2, COL19A1, KLK7, CDH1, SERPINH1, ITGB8, EMILIN2, COL25A1, ITGA2, MMP3, ADAM19, COL1A1, BCAN, MMP11, COL3A1, MMP16, COL6A2, COL6A1, COL8A2, MMP19* | Extracellular matrix (ECM) organization |
| PFAM | PF01391:Collagen triple helix repeat (20 copies) | 12 | 9.91E-04 | *COL1A1, COL3A1, C1QTNF1, COL16A1, COL14A1, COL25A1, COL6A2, C1QTNF6, COL12A1, COL6A1, COL8A2, COL19A1* |  |
| REACTOME_PATHWAY | R-MMU-1650814~Collagen biosynthesis and modifying enzymes | 11 | 0.00111578 | *COL1A1, COL3A1, COL16A1, COL14A1, COL25A1, COL6A2, COL12A1, COL6A1, COL8A2, SERPINH1, COL19A1* |  |
| REACTOME_PATHWAY | R-MMU-1474290~Collagen formation | 12 | 0.00151228 | *COL1A1, COL3A1, COL16A1, COL14A1, COL25A1, COL6A2, COL12A1, MMP3, COL6A1, COL8A2, SERPINH1, COL19A1* |  |
| UP_SEQ_FEATURE | DOMAIN:Laminin G-like | 7 | 0.00172737 | *NELL1, COL16A1, COL14A1, COL12A1, SLIT2, THBS1, COL19A1* |  |
| BIOGRID_INTERACTION | 12814:collagen, type XI, alpha 1(Col11a1) | 4 | 0.00220931 | *COL16A1, COL14A1, COL6A2, COL12A1* |  |
| REACTOME_PATHWAY | R-MMU-216083~Integrin cell surface interactions | 11 | 0.00234924 | *COL1A1, COL3A1, COL16A1, ITGB5, CDH1, COL6A2, ITGA2, COL6A1, COL8A2, ICAM2, ITGB8* |  |
| UP_SEQ_FEATURE | DOMAIN:Collagen-like 4 | 5 | 0.0045236 | *COL16A1, COL14A1, COL25A1, COL12A1, COL19A1* |  |
| INTERPRO | IPR001791:Laminin G domain | 9 | 0.00606799 | *NELL1, COL16A1, CNTNAP5A, COL14A1, COL12A1, SLIT2, THBS1, HSPG2, COL19A1* |  |
| **Supplementary Table S3.** (Continued) | | | | | |
| **Category** | **Term** | **Count** | ***P* Value** | **Genes** | **Annotation Cluster related to:** |
| UP_SEQ_FEATURE | DOMAIN:Collagen-like 3 | 5 | 0.00905432 | *COL16A1, COL14A1, COL25A1, COL12A1, COL19A1* | Extracellular matrix (ECM) organization |
| UP_SEQ_FEATURE | DOMAIN:Collagen-like 5 | 4 | 0.02133252 | *COL16A1, COL14A1, COL25A1, COL19A1* |  |
| GOTERM_MF_DIRECT | GO:0008083~growth factor activity | 22 | 1.92E-05 | *LEFTY1, PSPN, TGFB3, HGF, GMFG, GDF2, INHBB, CXCL1, VEGFD, PTN, FGF2, FGF5, GDF9, IL6, CXCL12, PDGFD, LEP, OGN, CCN5, CCN2, FGF12, FGF21* | Growth factors |
| UP_KW_MOLECULAR_FUNCTION | KW-0339~Growth factor | 19 | 7.70E-05 | *LEFTY1, PSPN, TGFB3, HGF, GMFG, GDF2, INHBB, CXCL1, VEGFD, PTN, FGF2, FGF5, GDF9, IL6, CXCL12, PDGFD, OGN, FGF12, FGF21* |  |
| PFAM | PF00019:Transforming growth factor beta like domain | 6 | 0.02957525 | *GDF9, LEFTY1, PSPN, TGFB3, GDF2, INHBB* |  |

| **Supplementary Table S4.** *CSTA*, overexpressed in pancreatic ductal adenocarcinoma (PDAC) tumor tissues, induced *in vivo* the differential expression of 2455 genes (p-value < 0.05). The listed genes are sorted by fold-change: 1684 genes were upregulated with CSTA (fold-change ≥ 1.5; gene set A) while 771 genes were downregulated (fold-change ≤ 0.67, gene set B). | | | | | |
| --- | --- | --- | --- | --- | --- |
| **Parametric p-value** | **Geom mean of intensities in tumors where *CSTA* was induced** | **Geom mean of intensities in tumors where *CSTA* was not induced** | **Fold-change [*CSTA* induced/*CSTA* not induced]** | **Agilent UniqueID** | **Gene Symbol** |
| 0.0021659 | 2510.47 | 8.13 | 308.74 | A_51_P339843 | *Mcpt2* |
| 0.002967 | 2478.37 | 48.78 | 50.8 | A_51_P409010 | *Comp* |
| 0.0421636 | 84.29 | 1.89 | 44.52 | A_52_P956261 | *Ndnf* |
| 4.61E-05 | 283.44 | 7.54 | 37.59 | A_51_P484526 | *Wif1* |
| 0.0131653 | 122.16 | 3.3 | 37.03 | A_52_P554703 | *Gprin3* |
| 0.0045248 | 259 | 7.74 | 33.45 | A_52_P419678 | *Serpina3f* |
| 0.0007482 | 12721.29 | 381.88 | 33.31 | A_51_P461665 | *Cxcl9* |
| 0.0473495 | 2823.14 | 96.32 | 29.31 | A_51_P162162 | *Inmt* |
| 0.001639 | 9005.29 | 314.82 | 28.6 | A_51_P207622 | *Fmod* |
| 0.0111256 | 104.28 | 3.66 | 28.47 | A_51_P323812 | *Slc6a12* |
| 0.0008567 | 7536.71 | 276.18 | 27.29 | A_51_P102987 | *Penk* |
| 0.0017263 | 310.86 | 12.42 | 25.03 | A_52_P68893 | *Ifng* |
| 0.0165381 | 923.83 | 36.97 | 24.99 | A_52_P414464 | *Rcan2* |
| 0.015645 | 683.53 | 30 | 22.78 | A_52_P87843 | *Aldh1a3* |
| 0.0007233 | 4315.5 | 190.85 | 22.61 | A_51_P423308 | *Igfbp2* |
| 0.0010783 | 263.26 | 12.47 | 21.12 | A_51_P258721 | *Tpsg1* |
| 0.0004099 | 3322.79 | 160.03 | 20.76 | A_52_P317393 | *Adgrg1* |
| 0.0001069 | 114.13 | 5.6 | 20.37 | A_51_P278653 | *Rprm* |
| 0.038383 | 278.95 | 14.05 | 19.86 | A_51_P333274 | *Gzmb* |
| 0.0061742 | 185.83 | 9.4 | 19.78 | A_51_P190961 | *Gzmf* |
| 4.38E-05 | 27.94 | 1.46 | 19.09 | A_51_P392005 | *Car8* |
| 0.0156292 | 64.31 | 3.49 | 18.43 | A_52_P480044 | *BC023105* |
| 7.15E-05 | 650.17 | 35.85 | 18.14 | A_51_P458130 | *Tph1* |
| 0.038163 | 2940.56 | 172.18 | 17.08 | A_52_P552036 | *Myh2* |
| 0.000287 | 289.37 | 17.18 | 16.85 | A_51_P100852 | *Fam26f* |
| 0.0118118 | 4174.69 | 264.96 | 15.76 | A_52_P362106 | *AK005018* |
| 0.000411 | 2150.67 | 139.42 | 15.43 | A_51_P459477 | *Col11a1* |
| 0.0048181 | 293.26 | 19.35 | 15.15 | A_52_P522640 | *Alx4* |
| 0.0055769 | 186.29 | 12.33 | 15.11 | A_52_P8459 | *Tpsab1* |
| 0.0395705 | 120.71 | 8 | 15.09 | A_52_P390127 | *Klrc1* |
| 0.0238172 | 18253.14 | 1229.96 | 14.84 | A_51_P393226 | *H2-Eb1* |
| 0.0110156 | 713.89 | 48.81 | 14.63 | A_51_P327405 | *Gbp8* |
| 0.007869 | 6694.26 | 463.53 | 14.44 | A_51_P326191 | *Serpina3g* |
| 0.0018754 | 302.01 | 20.92 | 14.43 | A_52_P335178 | *Prf1* |
| 0.0075978 | 831.17 | 57.67 | 14.41 | A_51_P463562 | *Gbp4* |
| 0.0016522 | 79.13 | 5.58 | 14.18 | A_51_P346243 | *Gp1ba* |
| 0.0254608 | 89 | 6.34 | 14.05 | A_51_P189451 | *Klrk1* |
| 0.0311487 | 74.27 | 5.32 | 13.95 | A_52_P276955 | *Epha3* |
| 0.0454378 | 1068.27 | 76.68 | 13.93 | A_52_P109941 | *Ciita* |
| 0.0046188 | 1484.95 | 111.56 | 13.31 | A_51_P444290 | *Slamf8* |
| 0.0016428 | 29.46 | 2.25 | 13.12 | A_52_P600417 | *Rgs8* |
| 0.0079721 | 44.99 | 3.45 | 13.03 | A_51_P283456 | *Cyp2e1* |
| 0.0091283 | 16541.12 | 1277.72 | 12.95 | A_52_P343306 | *H2-Aa* |
| 0.0001237 | 57.64 | 4.49 | 12.83 | A_51_P310676 | *Galr2* |
| 0.0013273 | 58.14 | 4.57 | 12.73 | A_51_P268697 | *Slc1a3* |
| 0.0015732 | 50.31 | 3.96 | 12.72 | A_51_P499599 | *Osr2* |
| 0.0395931 | 652.97 | 55.65 | 11.73 | A_52_P233411 | *Insc* |
| 0.0013408 | 26051.31 | 2228.73 | 11.69 | A_51_P387239 | *Iigp1* |
| 0.000429 | 2777.71 | 247.59 | 11.22 | A_52_P302544 | *Col8a2* |
| 0.00767 | 2885.25 | 258.84 | 11.15 | A_52_P189772 | *Gbp6* |
| 0.0469694 | 56.45 | 5.1 | 11.07 | A_51_P414879 | *ENSMUST00000103304* |
| 0.0137814 | 123.19 | 11.24 | 10.96 | A_52_P947847 | *Gm4951* |
| 0.0002174 | 53.5 | 4.91 | 10.9 | A_51_P221072 | *C1qtnf7* |
| 0.0028653 | 6938.81 | 636.76 | 10.9 | A_51_P390804 | *Wisp2* |
| 0.0017284 | 88.57 | 8.15 | 10.87 | A_51_P457481 | *Sectm1a* |
| 0.0271326 | 168.69 | 15.76 | 10.71 | A_51_P171832 | *Nrgn* |
| 0.0038392 | 28.32 | 2.65 | 10.68 | A_51_P467438 | *Olfr1193* |
| 0.0026991 | 1285.48 | 120.81 | 10.64 | A_52_P254155 | *Tpsb2* |
| 0.0452863 | 39113.65 | 3681.24 | 10.63 | A_51_P464703 | *Ccl8* |
| 0.0004976 | 8083.26 | 760.93 | 10.62 | A_51_P514035 | *Cma1* |
| 0.0008609 | 95.73 | 9.03 | 10.6 | A_52_P164821 | *Gm12250* |
| 0.022036 | 77766.93 | 7544.83 | 10.31 | A_52_P683598 | *H2-Ab1* |
| 0.0006336 | 22.07 | 2.17 | 10.18 | A_51_P373550 | *Nphs2* |
| 0.0292655 | 75718.65 | 7530.13 | 10.06 | A_51_P284608 | *Cd74* |
| 0.000405 | 13403.66 | 1348.43 | 9.94 | A_51_P311476 | *Rgma* |
| 0.0006146 | 98.39 | 10.04 | 9.8 | A_52_P611783 | *Fxyd2* |
| 0.0003161 | 8797.1 | 911.91 | 9.65 | A_52_P1020860 | *AW112010* |
| 0.000174 | 2075.2 | 215.38 | 9.64 | A_51_P327511 | *Fgl2* |
| 0.0024578 | 4394.02 | 457.48 | 9.6 | A_51_P214127 | *Cpa3* |
| 0.0018764 | 35293.54 | 3686.69 | 9.57 | A_51_P112355 | *Igtp* |
| 0.0054188 | 148.05 | 15.75 | 9.4 | A_51_P410387 | *Apol10b* |
| 0.005519 | 8355.59 | 892.17 | 9.37 | A_51_P145132 | *Mcpt4* |
| 0.0002087 | 28920.46 | 3108.47 | 9.3 | A_52_P486260 | *Prelp* |
| 0.0011166 | 2390.41 | 259.18 | 9.22 | A_51_P145130 | *Cma2* |
| 0.0298609 | 22406.17 | 2445.07 | 9.16 | A_52_P638459 | *Ccl5* |
| 0.0403218 | 369.86 | 40.49 | 9.14 | A_52_P165657 | *Dnase1l3* |
| 0.0192951 | 112.74 | 12.47 | 9.04 | A_51_P449995 | *C6* |
| 0.0006136 | 15133.23 | 1687.21 | 8.97 | A_51_P416295 | *Irgm2* |
| 0.0019886 | 3733.79 | 418.36 | 8.92 | A_51_P473734 | *H2-DMa* |
| 0.0007082 | 28757.56 | 3310.59 | 8.69 | A_51_P203955 | *Gbp2* |
| 0.0078244 | 4468.67 | 516.47 | 8.65 | A_51_P295085 | *Ogn* |
| 0.0002256 | 54.53 | 6.35 | 8.59 | A_52_P5942 | *Cd200r3* |
| 0.0018424 | 1956.02 | 229.86 | 8.51 | A_51_P248666 | *Cd274* |
| 0.0088384 | 2667.38 | 314.13 | 8.49 | A_52_P152133 | *H2-Q5* |
| 0.0002052 | 73.49 | 8.75 | 8.4 | A_52_P105537 | *Nov* |
| 0.0006621 | 79.97 | 9.53 | 8.39 | A_51_P309727 | *Cyp11a1* |
| 0.0395842 | 26.14 | 3.12 | 8.38 | A_51_P410859 | *9430085L16Rik* |
| 0.0076686 | 1625.37 | 196.08 | 8.29 | A_52_P327664 | *Gbp5* |
| 0.0016857 | 53.82 | 6.55 | 8.21 | A_52_P176865 | *Gm5431* |
| 0.0056983 | 29.78 | 3.64 | 8.18 | A_51_P322768 | *AK082639* |
| 0.0460872 | 76.85 | 9.55 | 8.04 | A_52_P646957 | *Grpr* |
| 0.0236754 | 55.82 | 6.95 | 8.03 | A_52_P288260 | *Mrgprb1* |
| 0.0023083 | 17.31 | 2.2 | 7.86 | A_51_P201873 | *Gpr88* |
| 0.0432419 | 77.46 | 9.87 | 7.85 | A_52_P125897 | *ENSMUST00000103317* |
| 0.0002491 | 4119.18 | 532.99 | 7.73 | A_51_P520849 | *Sfrp2* |
| 0.0160616 | 49.54 | 6.45 | 7.69 | A_51_P171107 | *Tmem35a* |
| 0.015541 | 1862.6 | 242.11 | 7.69 | A_51_P420276 | *Plxdc2* |
| 0.0104897 | 500.64 | 65.31 | 7.67 | A_51_P137419 | *Cst7* |
| 0.0021126 | 182.08 | 23.74 | 7.67 | A_52_P365660 | *Lrrc4c* |
| 0.0170277 | 71.27 | 9.43 | 7.56 | A_52_P451485 | *Cobl* |
| 0.0134518 | 577.57 | 76.54 | 7.55 | A_52_P499640 | *A530032D15Rik* |
| 0.0011952 | 13.97 | 1.85 | 7.55 | A_51_P380778 | *A730056A06Rik* |
| 0.0327321 | 87.79 | 11.69 | 7.51 | A_52_P103101 | *Ecm2* |
| 0.0006831 | 31.42 | 4.19 | 7.5 | A_52_P632921 | *NAP065340-1* |
| 0.0002076 | 43.56 | 5.81 | 7.49 | A_51_P501773 | *Slc35f3* |
| 0.0005795 | 409.19 | 55.01 | 7.44 | A_51_P105380 | *2010005H15Rik* |
| 0.0174841 | 44.53 | 6 | 7.42 | A_52_P25026 | *Klra17* |
| 0.0440739 | 61.96 | 8.37 | 7.4 | A_51_P218335 | *Tbx1* |
| 0.0018742 | 32536.14 | 4412.25 | 7.37 | A_51_P110301 | *C3* |
| 0.0398166 | 21.82 | 2.98 | 7.32 | A_51_P481958 | *Cldn1* |
| 0.0187415 | 5121.91 | 702.16 | 7.29 | A_51_P199135 | *Cd83* |
| 0.0005136 | 6837.27 | 958.87 | 7.13 | A_51_P129229 | *Ifi47* |
| 0.0064005 | 289.76 | 40.77 | 7.11 | A_51_P247184 | *Npr3* |
| 0.0037899 | 209.52 | 29.63 | 7.07 | A_52_P147466 | *Klra2* |
| 0.0165313 | 45.76 | 6.47 | 7.07 | A_51_P497152 | *Fgf9* |
| 0.0007639 | 8979.82 | 1277.2 | 7.03 | A_51_P345366 | *Psmb8* |
| 0.0020059 | 62.66 | 8.99 | 6.97 | A_52_P370031 | *AK043317* |
| 0.0179292 | 3216.12 | 462.5 | 6.95 | A_51_P246653 | *Clec7a* |
| 0.0022845 | 9780.56 | 1412.95 | 6.92 | A_51_P165244 | *Gbp3* |
| 0.0007707 | 3460.83 | 502.61 | 6.89 | A_51_P413866 | *Cfb* |
| 0.0158727 | 19.13 | 2.79 | 6.85 | A_52_P287456 | *Gca* |
| 0.0012715 | 2652.84 | 388.49 | 6.83 | A_52_P577384 | *Il18bp* |
| 0.0075556 | 1750.96 | 257.67 | 6.8 | A_52_P358360 | *ENSMUST00000177715* |
| 0.0004486 | 32.92 | 4.86 | 6.78 | A_52_P39505 | *C1s2* |
| 0.0060715 | 17.31 | 2.55 | 6.78 | A_52_P1044262 | *AK051949* |
| 0.0029879 | 413.73 | 62.16 | 6.66 | A_52_P261262 | *Slamf7* |
| 0.0472815 | 5946.9 | 892.9 | 6.66 | A_51_P359570 | *Ifit3* |
| 0.0070412 | 97.58 | 14.64 | 6.66 | A_51_P448741 | *Tnfsf10* |
| 0.0060026 | 14517.9 | 2192.16 | 6.62 | A_51_P278868 | *H2-DMb1* |
| 0.0037766 | 209.85 | 31.78 | 6.6 | A_51_P427132 | *Fbxl13* |
| 0.0260968 | 52.88 | 8.02 | 6.59 | A_51_P308844 | *Nrn1* |
| 0.0260754 | 56.93 | 8.7 | 6.54 | A_51_P119387 | *Tm4sf5* |
| 0.002101 | 965.42 | 147.93 | 6.53 | A_52_P586004 | *Hk3* |
| 0.0009924 | 7327.91 | 1133.23 | 6.47 | A_51_P369803 | *Psmb9* |
| 0.0408791 | 672.36 | 104 | 6.46 | A_51_P123625 | *Acod1* |
| 0.0485287 | 44.18 | 6.85 | 6.45 | A_51_P103364 | *ENSMUST00000103552* |
| 0.0007342 | 68.59 | 10.67 | 6.43 | A_51_P462271 | *Acan* |
| 0.0095718 | 25.85 | 4.04 | 6.4 | A_51_P423518 | *Amph* |
| 0.0010129 | 3564.03 | 556.54 | 6.4 | A_51_P241319 | *Cilp* |
| 0.001126 | 3724.89 | 582.71 | 6.39 | A_51_P208793 | *C1s1* |
| 0.0072531 | 11145.42 | 1746.62 | 6.38 | A_52_P676510 | *Tgtp1* |
| 0.0469857 | 5088.18 | 800.13 | 6.36 | A_52_P653054 | *NAP007796-001* |
| 0.0002274 | 27.66 | 4.37 | 6.33 | A_51_P476879 | *Olfml1* |
| 0.0138842 | 38.36 | 6.08 | 6.31 | A_52_P505038 | *Bend4* |
| 0.0030853 | 12.49 | 1.98 | 6.3 | A_51_P515120 | *Hs3st3a1* |
| 0.0263947 | 40.89 | 6.5 | 6.29 | A_52_P679247 | *AI504432* |
| 0.0134992 | 1648.03 | 262.62 | 6.28 | A_52_P615247 | *ENSMUST00000096862* |
| 0.0021971 | 4563.89 | 726.83 | 6.28 | A_51_P432641 | *Cxcl10* |
| 0.0003949 | 8181.86 | 1303.94 | 6.27 | A_51_P181517 | *Fcgr4* |
| 0.0270974 | 67.96 | 10.86 | 6.26 | A_52_P384264 | *Zfp185* |
| 0.0007428 | 140.53 | 22.45 | 6.26 | A_51_P226791 | *A_51_P226791* |
| 0.0472868 | 543.48 | 87 | 6.25 | A_51_P429770 | *Fcer1a* |
| 0.0261649 | 24.48 | 3.93 | 6.23 | A_51_P343598 | *1500009L16Rik* |
| 0.0006434 | 12.2 | 1.96 | 6.22 | A_51_P350069 | *Olfr1189* |
| 0.0020854 | 4225.82 | 682.6 | 6.19 | A_51_P165182 | *Batf2* |
| 0.0281156 | 113.38 | 18.45 | 6.14 | A_51_P280455 | *Prg4* |
| 0.000381 | 76.2 | 12.46 | 6.12 | A_51_P458778 | *Hpgd* |
| 0.0067842 | 924.57 | 151.4 | 6.11 | A_51_P112966 | *Ch25h* |
| 0.0007847 | 8.21 | 1.35 | 6.09 | A_51_P248865 | *Foxf2* |
| 0.0291298 | 11.47 | 1.88 | 6.09 | A_52_P162967 | *Cd28* |
| 0.044219 | 113.55 | 18.67 | 6.08 | A_51_P493117 | *Slc16a9* |
| 0.0002357 | 144.86 | 23.88 | 6.07 | A_52_P617638 | *Nox4* |
| 0.0004752 | 23.82 | 3.94 | 6.04 | A_52_P153019 | *Ptgfr* |
| 0.0112561 | 77.6 | 12.87 | 6.03 | A_51_P424532 | *Vnn1* |
| 0.0089 | 126.06 | 20.95 | 6.02 | A_51_P309920 | *Itga8* |
| 0.0007929 | 1242.67 | 206.35 | 6.02 | A_51_P366811 | *Apod* |
| 0.005124 | 29.3 | 4.88 | 6 | A_51_P110381 | *Cd207* |
| 0.0041581 | 49.03 | 8.18 | 5.99 | A_51_P224530 | *Nrip3* |
| 0.0120878 | 5443.62 | 910.35 | 5.98 | A_51_P404815 | *Apol6* |
| 0.0158982 | 30.21 | 5.06 | 5.97 | A_51_P281078 | *A_51_P281078* |
| 0.0120466 | 180.92 | 30.37 | 5.96 | A_51_P305508 | *Rerg* |
| 0.000593 | 749 | 125.66 | 5.96 | A_51_P316311 | *Ppfia4* |
| 0.008777 | 5603.98 | 943.69 | 5.94 | A_51_P184936 | *Zbp1* |
| 0.0046406 | 549.94 | 92.78 | 5.93 | A_51_P372456 | *Wdr86* |
| 0.0095039 | 398.58 | 67.95 | 5.87 | A_51_P456952 | *Il2rg* |
| 0.0046644 | 16.51 | 2.83 | 5.84 | A_52_P615630 | *Adgrf2* |
| 0.001412 | 918.06 | 157.87 | 5.82 | A_51_P378789 | *Cxcl13* |
| 0.0067366 | 1593.75 | 274.44 | 5.81 | A_51_P181297 | *Serpinb1a* |
| 0.0028755 | 55.18 | 9.49 | 5.81 | A_51_P142421 | *Rspo1* |
| 0.0028425 | 1400.1 | 242.55 | 5.77 | A_51_P436652 | *Ccl7* |
| 0.0020714 | 103.12 | 17.9 | 5.76 | A_52_P470150 | *Ddc* |
| 0.0469358 | 85.17 | 14.84 | 5.74 | A_52_P150273 | *2610027K06Rik* |
| 0.0010204 | 1148.78 | 200.58 | 5.73 | A_51_P331831 | *Hvcn1* |
| 0.048162 | 11.07 | 1.94 | 5.72 | A_51_P415395 | *C2cd4b* |
| 0.0098049 | 2051.92 | 359.62 | 5.71 | A_51_P115441 | *Dpysl3* |
| 0.0091205 | 12.96 | 2.27 | 5.71 | A_52_P607683 | *Ppm1e* |
| 0.0042991 | 9.05 | 1.59 | 5.69 | A_51_P170476 | *Kcns1* |
| 0.038201 | 12.05 | 2.15 | 5.62 | A_52_P207303 | *Bmp3* |
| 0.0007946 | 4821.16 | 861.92 | 5.59 | A_51_P466229 | *Pdgfrl* |
| 0.0103969 | 293.11 | 52.5 | 5.58 | A_51_P397437 | *Prss46* |
| 0.018916 | 19.28 | 3.45 | 5.58 | A_51_P463003 | *Tslp* |
| 0.0434414 | 128.04 | 23.02 | 5.56 | A_51_P487791 | *Adrb2* |
| 0.0008464 | 612.27 | 110.04 | 5.56 | A_51_P100327 | *Tap1* |
| 0.0297613 | 37599.32 | 6769.46 | 5.55 | A_51_P515883 | *Plac8* |
| 0.0004437 | 368.51 | 66.65 | 5.53 | A_51_P355906 | *Kit* |
| 0.005422 | 402.38 | 72.84 | 5.52 | A_51_P214985 | *Zfp521* |
| 0.027983 | 17.37 | 3.14 | 5.52 | A_52_P987766 | *AK037330* |
| 0.0008961 | 430.95 | 78.31 | 5.5 | A_51_P107591 | *Klrb1b* |
| 0.0067411 | 747.54 | 136.27 | 5.49 | A_51_P463846 | *Gbp7* |
| 0.0059843 | 503.56 | 91.76 | 5.49 | A_51_P257934 | *Tnfsf13b* |
| 0.0116228 | 333.55 | 61.01 | 5.47 | A_52_P382149 | *Cyp26a1* |
| 0.0018081 | 1048.63 | 192.09 | 5.46 | A_52_P467726 | *Nsg1* |
| 0.0235054 | 88.59 | 16.33 | 5.43 | A_52_P1173559 | *A_52_P1173559* |
| 0.0008896 | 252.15 | 46.43 | 5.43 | A_52_P114260 | *C1rb* |
| 0.0004769 | 12753.79 | 2362.27 | 5.4 | A_52_P505218 | *Stat1* |
| 0.0058066 | 8.22 | 1.54 | 5.35 | A_51_P382544 | *Majin* |
| 0.0360178 | 162.19 | 30.34 | 5.35 | A_51_P333460 | *5430427O19Rik* |
| 0.0090532 | 170.79 | 31.99 | 5.34 | A_51_P488739 | *Hcar2* |
| 0.0003412 | 28 | 5.28 | 5.31 | A_51_P446179 | *Tmprss4* |
| 0.0019151 | 5117.71 | 965.96 | 5.3 | A_51_P384318 | *C1ra* |
| 0.030618 | 69.83 | 13.26 | 5.27 | A_52_P70856 | *Frmpd1* |
| 0.0069195 | 18.04 | 3.42 | 5.27 | A_52_P683836 | *TC1655871* |
| 0.0191841 | 945.13 | 179.67 | 5.26 | A_51_P473498 | *Gpr171* |
| 0.001753 | 1462.29 | 277.85 | 5.26 | A_51_P428134 | *Lrig3* |
| 0.0117349 | 18.85 | 3.6 | 5.24 | A_51_P421724 | *St6galnac5* |
| 0.0019858 | 1611.42 | 307.24 | 5.24 | A_51_P279606 | *Socs1* |
| 0.0003966 | 625.86 | 119.93 | 5.22 | A_52_P177599 | *Zfp773* |
| 0.0168687 | 66.79 | 12.83 | 5.21 | A_51_P139716 | *Fam26e* |
| 0.0135337 | 2486.2 | 477.85 | 5.2 | A_51_P329291 | *Cygb* |
| 0.0218269 | 388.63 | 74.88 | 5.19 | A_51_P205008 | *Lst1* |
| 0.0112338 | 29.07 | 5.6 | 5.19 | A_52_P127813 | *Ifi213* |
| 0.00375 | 3514.01 | 678.14 | 5.18 | A_52_P588483 | *Fbln1* |
| 0.0021134 | 64.96 | 12.6 | 5.16 | A_52_P931133 | *AK053561* |
| 0.0027799 | 1179.36 | 228.56 | 5.16 | A_52_P257502 | *Igfbp4* |
| 0.0006465 | 22.55 | 4.37 | 5.16 | A_51_P477736 | *4932415M13Rik* |
| 0.0090356 | 51.99 | 10.09 | 5.15 | A_51_P330044 | *Cyp2j9* |
| 0.0039599 | 22.43 | 4.36 | 5.14 | A_52_P61697 | *9930111J21Rik2* |
| 0.0117947 | 131.79 | 25.71 | 5.13 | A_52_P326187 | *ENSMUST00000192876* |
| 0.0082433 | 14.85 | 2.9 | 5.12 | A_51_P279284 | *BC051142* |
| 0.0350754 | 96.54 | 18.85 | 5.12 | A_51_P302566 | *Maob* |
| 0.019653 | 180.66 | 35.38 | 5.11 | A_51_P465331 | *Fgf7* |
| 0.0003456 | 11104.62 | 2183.85 | 5.08 | A_51_P257457 | *Ltbp2* |
| 0.0031569 | 816.56 | 161.32 | 5.06 | A_52_P578732 | *Ccr5* |
| 0.0489837 | 13.62 | 2.69 | 5.06 | A_51_P514985 | *Irf4* |
| 0.0050679 | 526.27 | 103.92 | 5.06 | A_51_P140710 | *Ccl3* |
| 0.0021474 | 1965.35 | 391.04 | 5.03 | A_52_P14600 | *Cd72* |
| 0.0373705 | 23.38 | 4.65 | 5.03 | A_52_P77764 | *Slc7a2* |
| 0.0090451 | 2693.42 | 538.78 | 5 | A_51_P452629 | *Tlr2* |
| 0.0022072 | 550.43 | 110.51 | 4.98 | A_52_P424970 | *Itgal* |
| 0.0042929 | 361.91 | 73.01 | 4.96 | A_51_P520718 | *Ptger3* |
| 0.034948 | 7381.27 | 1490.34 | 4.95 | A_51_P463765 | *Timp3* |
| 0.0140522 | 120.55 | 24.48 | 4.92 | A_52_P619911 | *Dact2* |
| 0.0030138 | 84.56 | 17.24 | 4.9 | A_51_P364185 | *Lilra5* |
| 0.0010813 | 38.29 | 7.83 | 4.89 | A_52_P675039 | *Fhad1* |
| 0.001669 | 36.09 | 7.38 | 4.89 | A_52_P425890 | *Slfn1* |
| 0.0368787 | 543.99 | 111.2 | 4.89 | A_51_P466910 | *Cacna1g* |
| 0.0101454 | 188.46 | 38.64 | 4.88 | A_51_P271887 | *Reck* |
| 0.0078266 | 65.86 | 13.49 | 4.88 | A_51_P453909 | *Cyp2f2* |
| 0.004907 | 127.53 | 26.19 | 4.87 | A_51_P104608 | *Rnase6* |
| 0.0185561 | 1175.7 | 241.22 | 4.87 | A_51_P194415 | *Slco3a1* |
| 0.0012622 | 21.51 | 4.43 | 4.86 | A_51_P234975 | *Ms4a2* |
| 0.0026693 | 227.57 | 46.94 | 4.85 | A_51_P174723 | *Cd86* |
| 0.0053803 | 8711.32 | 1796.83 | 4.85 | A_51_P195244 | *Mfap4* |
| 0.0024739 | 429.33 | 88.91 | 4.83 | A_51_P365854 | *Fam43a* |
| 0.0151977 | 90.16 | 18.82 | 4.79 | A_52_P599964 | *LOC102639543* |
| 0.0011552 | 3145.88 | 656.27 | 4.79 | A_51_P291417 | *Thbd* |
| 0.0050231 | 37.63 | 7.85 | 4.79 | A_51_P132013 | *Cysltr2* |
| 0.0251148 | 1995.41 | 417.4 | 4.78 | A_51_P286737 | *Ccl2* |
| 0.0037035 | 849.88 | 179.33 | 4.74 | A_51_P127297 | *Hsd11b1* |
| 0.0087327 | 7.02 | 1.48 | 4.74 | A_52_P145433 | *Snhg14* |
| 0.0221983 | 20.85 | 4.41 | 4.73 | A_52_P640694 | *Rims3* |
| 0.0280334 | 106.75 | 22.57 | 4.73 | A_52_P109270 | *P3h2* |
| 0.0391641 | 15.78 | 3.35 | 4.72 | A_51_P342556 | *Zfhx4* |
| 0.0018143 | 287.93 | 61.03 | 4.72 | A_52_P16873 | *Rasal3* |
| 0.0051669 | 1881.48 | 398.32 | 4.72 | A_51_P186703 | *Fbln5* |
| 0.0005349 | 1920.1 | 407.88 | 4.71 | A_51_P202633 | *Ebi3* |
| 0.0420546 | 39.16 | 8.45 | 4.64 | A_51_P388412 | *Cd55* |
| 0.0005953 | 39.88 | 8.6 | 4.64 | A_52_P216613 | *Gpr18* |
| 0.0010913 | 1915.6 | 413 | 4.64 | A_51_P428708 | *C4b* |
| 0.044033 | 39449.93 | 8504.88 | 4.64 | A_51_P426270 | *Mgp* |
| 0.0117513 | 7461.77 | 1606.62 | 4.64 | A_51_P417720 | *Itga11* |
| 0.0009374 | 10584.35 | 2293.2 | 4.62 | A_51_P251768 | *Adgre1* |
| 0.0056678 | 195.83 | 42.52 | 4.61 | A_51_P288138 | *Fpr2* |
| 0.0013223 | 25.82 | 5.6 | 4.61 | A_51_P306287 | *Slc30a2* |
| 0.0309486 | 1197.69 | 259.68 | 4.61 | A_51_P352296 | *Sfrp1* |
| 0.0272967 | 39.8 | 8.65 | 4.6 | A_52_P97699 | *D430019H16Rik* |
| 0.0171512 | 594.39 | 129.1 | 4.6 | A_52_P764477 | *Gm8995* |
| 0.0349783 | 22.17 | 4.83 | 4.59 | A_51_P190249 | *Ctnnd2* |
| 0.0068638 | 171.02 | 37.42 | 4.57 | A_52_P111031 | *Pcdh17* |
| 0.0121587 | 133.2 | 29.13 | 4.57 | A_51_P212057 | *Serpinb1c* |
| 0.0040186 | 113.69 | 24.98 | 4.55 | A_52_P45563 | *Slamf6* |
| 0.0110014 | 21.33 | 4.7 | 4.54 | A_51_P273449 | *Gnaz* |
| 0.0113566 | 27063.5 | 5959.77 | 4.54 | A_52_P582059 | *Lyz1* |
| 0.001445 | 367.02 | 81.02 | 4.53 | A_52_P211956 | *Ms4a4d* |
| 0.0009973 | 7217.01 | 1597.12 | 4.52 | A_52_P512201 | *Sp110* |
| 0.00739 | 66.11 | 14.63 | 4.52 | A_51_P181286 | *Cd69* |
| 0.0082886 | 1435.83 | 317.34 | 4.52 | A_51_P195066 | *Sema4a* |
| 0.0012926 | 3678.72 | 815.73 | 4.51 | A_51_P494597 | *Themis2* |
| 0.0226858 | 42.44 | 9.4 | 4.51 | A_51_P200262 | *Gfi1* |
| 0.021391 | 44.71 | 9.94 | 4.5 | A_52_P95759 | *Sh3rf3* |
| 0.008784 | 64.2 | 14.34 | 4.48 | A_51_P346815 | *Arxes1* |
| 0.0005638 | 105.13 | 23.47 | 4.48 | A_51_P265444 | *Slc28a2* |
| 0.0027429 | 462.77 | 103.24 | 4.48 | A_51_P221223 | *Scg5* |
| 0.0159245 | 11.82 | 2.65 | 4.46 | A_52_P156314 | *Esr2* |
| 0.0056894 | 15966.5 | 3577.92 | 4.46 | A_51_P444447 | *Cebpd* |
| 0.0010256 | 239.35 | 53.73 | 4.45 | A_52_P319495 | *Boc* |
| 0.0050511 | 76.31 | 17.24 | 4.43 | A_52_P681557 | *Naaladl2* |
| 0.0230845 | 1036.56 | 233.76 | 4.43 | A_52_P444628 | *Cybb* |
| 0.0023771 | 1183.29 | 267.87 | 4.42 | A_52_P86176 | *Tap2* |
| 0.0369149 | 2529.12 | 572.12 | 4.42 | A_51_P394814 | *Svep1* |
| 0.0079383 | 115.76 | 26.17 | 4.42 | A_51_P268496 | *Slc24a3* |
| 0.0190028 | 87.81 | 19.85 | 4.42 | A_52_P417945 | *9330175E14Rik* |
| 0.0007027 | 158.38 | 36.03 | 4.4 | A_52_P451614 | *Tmod2* |
| 0.0009213 | 13.97 | 3.17 | 4.4 | A_52_P461105 | *Gpr31b* |
| 0.0164897 | 145.68 | 33.16 | 4.39 | A_51_P414126 | *Rab19* |
| 0.0008862 | 12379.34 | 2817.45 | 4.39 | A_51_P465350 | *Ly86* |
| 0.002805 | 12999.13 | 2968.53 | 4.38 | A_51_P262171 | *Irgm1* |
| 0.0157969 | 2895.79 | 660.98 | 4.38 | A_51_P186476 | *Slc11a1* |
| 0.0198878 | 1228.77 | 280.42 | 4.38 | A_52_P431615 | *Gm1966* |
| 0.0016729 | 454.35 | 103.95 | 4.37 | A_51_P496031 | *Ar* |
| 0.028861 | 178.95 | 40.94 | 4.37 | A_51_P495242 | *Lat* |
| 0.0022542 | 9.99 | 2.28 | 4.37 | A_52_P527438 | *Adgb* |
| 0.002986 | 902.26 | 207.15 | 4.36 | A_51_P517105 | *Aoah* |
| 0.0034665 | 34.51 | 7.92 | 4.36 | A_52_P357829 | *Gli2* |
| 0.0263953 | 7986.75 | 1832.88 | 4.36 | A_52_P251690 | *Gvin1* |
| 0.0068215 | 376.97 | 86.48 | 4.36 | A_51_P345393 | *Fas* |
| 0.0075649 | 82.45 | 19.02 | 4.34 | A_52_P568895 | *Kcnab1* |
| 0.004662 | 278.61 | 64.25 | 4.34 | A_51_P490867 | *Enpp4* |
| 0.0045735 | 392.38 | 90.48 | 4.34 | A_52_P393488 | *Ms4a4c* |
| 0.0177617 | 228.86 | 52.87 | 4.33 | A_52_P288251 | *Tmem204* |
| 0.0254889 | 1110.33 | 256.76 | 4.32 | A_51_P509573 | *Ccl4* |
| 0.0057552 | 7.02 | 1.62 | 4.32 | A_51_P163942 | *Nos2* |
| 0.0198983 | 28.08 | 6.52 | 4.31 | A_52_P151393 | *Lrrc75b* |
| 0.0022566 | 1141.55 | 264.95 | 4.31 | A_51_P326529 | *Col14a1* |
| 0.003183 | 128.96 | 30.07 | 4.29 | A_52_P365466 | *Plcb2* |
| 0.0024602 | 2046.81 | 477.89 | 4.28 | A_51_P235984 | *Il10ra* |
| 0.0218053 | 2197.54 | 514.42 | 4.27 | A_52_P591153 | *Pld4* |
| 0.0051943 | 46.45 | 10.9 | 4.26 | A_51_P377171 | *Tespa1* |
| 0.0054313 | 4499.84 | 1059.39 | 4.25 | A_52_P354823 | *Irf8* |
| 0.0220568 | 42.7 | 10.05 | 4.25 | A_52_P609738 | *Asic3* |
| 0.0103069 | 39.58 | 9.31 | 4.25 | A_52_P322927 | *ENSMUST00000117952* |
| 0.0025794 | 7460.03 | 1753.27 | 4.25 | A_51_P376238 | *Serping1* |
| 0.0037615 | 87.81 | 20.77 | 4.23 | A_51_P337210 | *Lrrc4* |
| 0.0006001 | 57.33 | 13.54 | 4.23 | A_52_P449871 | *Id4* |
| 0.0405282 | 12.45 | 2.95 | 4.22 | A_52_P867926 | *AK080210* |
| 0.0052895 | 12.55 | 2.97 | 4.22 | A_51_P427229 | *AK040292* |
| 0.0019376 | 26.71 | 6.35 | 4.21 | A_51_P112817 | *Cyp27a1* |
| 0.0115983 | 334.49 | 79.4 | 4.21 | A_52_P260659 | *Kcnj10* |
| 0.0022699 | 89.11 | 21.21 | 4.2 | A_51_P194609 | *Prss34* |
| 0.0062217 | 200.35 | 47.67 | 4.2 | A_51_P285779 | *Asphd2* |
| 0.0074201 | 488.28 | 116.4 | 4.19 | A_52_P199633 | *Trim30d* |
| 0.0376865 | 5.79 | 1.39 | 4.18 | A_52_P95096 | *1700025L06Rik* |
| 0.0187146 | 65.82 | 15.78 | 4.17 | A_52_P64383 | *Klf15* |
| 0.0073081 | 578.68 | 138.97 | 4.16 | A_51_P323180 | *Gbp9* |
| 0.0308737 | 20.2 | 4.88 | 4.14 | A_52_P28960 | *Gdf6* |
| 0.0009019 | 196.98 | 47.7 | 4.13 | A_52_P409601 | *Cd40* |
| 0.0022339 | 39.32 | 9.52 | 4.13 | A_52_P638457 | *Scimp* |
| 0.0055752 | 1104.77 | 267.45 | 4.13 | A_52_P467389 | *Slc15a3* |
| 0.0019928 | 4396.49 | 1067.52 | 4.12 | A_52_P337086 | *Thbs1* |
| 0.0006753 | 205.1 | 49.96 | 4.11 | A_51_P452533 | *Ildr2* |
| 0.0046006 | 95.93 | 23.33 | 4.11 | A_51_P208240 | *Tnfsf14* |
| 0.0128209 | 72.74 | 17.68 | 4.11 | A_51_P410715 | *Hs3st3b1* |
| 0.0013069 | 5420.21 | 1323.48 | 4.1 | A_52_P575178 | *Cdon* |
| 0.0039678 | 23.8 | 5.81 | 4.1 | A_52_P532957 | *Zfp667* |
| 0.0385352 | 1305.27 | 319.5 | 4.09 | A_52_P398989 | *Cytip* |
| 0.0136831 | 191.65 | 46.86 | 4.09 | A_51_P188054 | *Ebf1* |
| 0.0390422 | 2691.58 | 657.47 | 4.09 | A_52_P498241 | *Cdkn2b* |
| 0.0054842 | 8051.22 | 1972.07 | 4.08 | A_51_P365885 | *Csf1r* |
| 0.0005943 | 14589.32 | 3587.72 | 4.07 | A_51_P400543 | *Aif1* |
| 0.037732 | 729.46 | 179.2 | 4.07 | A_51_P345549 | *Nkd2* |
| 0.0430026 | 493.84 | 121.26 | 4.07 | A_51_P487690 | *Ifi44* |
| 0.0015743 | 6.81 | 1.68 | 4.06 | A_51_P349213 | *Fcrl1* |
| 0.0037574 | 69.41 | 17.1 | 4.06 | A_51_P401184 | *Rarres1* |
| 0.0258901 | 7.51 | 1.85 | 4.06 | A_52_P201730 | *A130071D04Rik* |
| 0.021801 | 78.62 | 19.35 | 4.06 | A_51_P323081 | *Bhlhb9* |
| 0.0078546 | 692.41 | 170.89 | 4.05 | A_51_P114693 | *Parm1* |
| 0.0017092 | 8730.71 | 2153.58 | 4.05 | A_51_P368171 | *LOC108167440* |
| 0.0007736 | 145.45 | 36.04 | 4.04 | A_51_P293401 | *AK089315* |
| 0.00613 | 35.72 | 8.92 | 4.01 | A_52_P621588 | *Ifnlr1* |
| 0.0048334 | 83.78 | 20.89 | 4.01 | A_51_P371750 | *Marco* |
| 0.0030902 | 20.82 | 5.21 | 4 | A_52_P763635 | *AK087135* |
| 0.0248622 | 3275.62 | 820.59 | 3.99 | A_51_P156108 | *Crlf1* |
| 0.0169357 | 105.94 | 26.61 | 3.98 | A_51_P367263 | *Lman1l* |
| 0.0115001 | 1367.5 | 345.02 | 3.96 | A_51_P447976 | *Fam46c* |
| 0.0099823 | 354.1 | 89.31 | 3.96 | A_52_P671794 | *Plscr4* |
| 0.0049347 | 851.63 | 215.87 | 3.95 | A_52_P203691 | *Arl5c* |
| 0.0253299 | 93.39 | 23.65 | 3.95 | A_51_P276479 | *4930486L24Rik* |
| 0.0296179 | 2095.51 | 530.19 | 3.95 | A_52_P332788 | *ENSMUST00000162421* |
| 0.028457 | 994.64 | 252.25 | 3.94 | A_52_P452689 | *Atf3* |
| 0.0031076 | 61.92 | 15.75 | 3.93 | A_52_P1149693 | *A930006K02Rik* |
| 0.0008635 | 4228.89 | 1078.74 | 3.92 | A_51_P497985 | *C2* |
| 0.0081863 | 92.22 | 23.61 | 3.91 | A_52_P430348 | *Serpinb1b* |
| 0.0098072 | 1599.22 | 409.34 | 3.91 | A_52_P219233 | *Loxl3* |
| 0.0338154 | 14553.18 | 3719.66 | 3.91 | A_52_P472302 | *Fxyd6* |
| 0.0035778 | 168.84 | 43.15 | 3.91 | A_51_P490678 | *Trim68* |
| 0.028802 | 1182.4 | 303.49 | 3.9 | A_51_P219109 | *Il12rb1* |
| 0.0276956 | 292.14 | 74.98 | 3.9 | A_51_P161021 | *Ifit2* |
| 0.0350125 | 908.87 | 232.97 | 3.9 | A_52_P367520 | *Nexn* |
| 0.0026036 | 1578.43 | 404.27 | 3.9 | A_51_P215627 | *Plac9a* |
| 0.0059051 | 17.23 | 4.44 | 3.88 | A_51_P207301 | *AK037047* |
| 0.0461573 | 290 | 75 | 3.87 | A_52_P1131540 | *AK046587* |
| 0.0312199 | 4459.92 | 1151.54 | 3.87 | A_51_P205000 | *AF000427* |
| 0.0017494 | 73.25 | 18.98 | 3.86 | A_51_P192397 | *Scube2* |
| 0.0024645 | 275.46 | 71.34 | 3.86 | A_51_P489289 | *Rhoh* |
| 0.0129075 | 294.53 | 76.46 | 3.85 | A_51_P270339 | *Pik3ap1* |
| 0.0079807 | 501.12 | 130.05 | 3.85 | A_51_P419286 | *Batf3* |
| 0.0017825 | 2112.82 | 548.24 | 3.85 | A_51_P108459 | *Gpr65* |
| 0.020365 | 21377.4 | 5570.23 | 3.84 | A_51_P146103 | *Irf1* |
| 0.0221103 | 205.16 | 53.38 | 3.84 | A_51_P440210 | *Prkg1* |
| 0.0055543 | 1549.7 | 403.14 | 3.84 | A_51_P408506 | *Icam1* |
| 0.0470128 | 81.22 | 21.22 | 3.83 | A_52_P285024 | *Sertad4* |
| 0.0094191 | 255.43 | 66.78 | 3.82 | A_51_P383524 | *Art3* |
| 0.0013248 | 113.81 | 29.98 | 3.8 | A_51_P338413 | *Stap1* |
| 0.0188838 | 9.12 | 2.4 | 3.8 | A_52_P335768 | *AK035943* |
| 0.0066005 | 36.65 | 9.63 | 3.8 | A_51_P179831 | *Arhgap44* |
| 0.0097991 | 65.01 | 17.15 | 3.79 | A_52_P541161 | *Rgs18* |
| 0.0207584 | 5.7 | 1.51 | 3.78 | A_52_P867755 | *AK030821* |
| 0.0008896 | 106.85 | 28.3 | 3.78 | A_52_P150683 | *Grap2* |
| 0.0429252 | 10.03 | 2.65 | 3.78 | A_52_P320394 | *Mrgprb3* |
| 0.0022416 | 30.59 | 8.12 | 3.77 | A_52_P117408 | *Tg* |
| 0.0124814 | 96.45 | 25.56 | 3.77 | A_52_P810953 | *AK041753* |
| 0.0012453 | 74.88 | 19.94 | 3.76 | A_51_P265374 | *Vnn3* |
| 0.0025153 | 1840.58 | 489.72 | 3.76 | A_51_P169061 | *Lpcat2* |
| 0.0107467 | 7166.58 | 1905.8 | 3.76 | A_51_P517388 | *Plbd1* |
| 0.0014263 | 4707.78 | 1251.74 | 3.76 | A_52_P99888 | *Cxcl16* |
| 0.0019986 | 44.91 | 11.94 | 3.76 | A_51_P416108 | *TC1596474* |
| 0.0035861 | 6.96 | 1.85 | 3.76 | A_52_P98287 | *Ddx43* |
| 0.0017225 | 234.85 | 62.84 | 3.74 | A_52_P287338 | *Eif4e3* |
| 0.0015846 | 6335.3 | 1700.31 | 3.73 | A_51_P514712 | *Parp14* |
| 0.0026896 | 970.03 | 260.04 | 3.73 | A_51_P103222 | *Slc39a4* |
| 0.0200852 | 935.16 | 250.5 | 3.73 | A_51_P225793 | *Prr5l* |
| 0.0412387 | 10.01 | 2.68 | 3.73 | A_52_P715616 | *D230034L24Rik* |
| 0.0102588 | 15588 | 4190.78 | 3.72 | A_51_P172054 | *Gas6* |
| 0.0101887 | 61.17 | 16.44 | 3.72 | A_51_P147123 | *Ntn1* |
| 0.0369853 | 61.86 | 16.66 | 3.71 | A_52_P404535 | *Pcdhac2* |
| 0.0126958 | 7.35 | 1.99 | 3.7 | A_52_P56818 | *Foxp2* |
| 0.042854 | 248.18 | 67.12 | 3.7 | A_51_P507509 | *Gimap1* |
| 0.0066297 | 186.46 | 50.56 | 3.69 | A_51_P454696 | *44256* |
| 0.0080774 | 822.98 | 223.02 | 3.69 | A_51_P287001 | *Fxyd1* |
| 0.0062091 | 70.1 | 18.98 | 3.69 | A_52_P333567 | *Adam33* |
| 0.0064048 | 346.53 | 93.79 | 3.69 | A_51_P384515 | *Ido2* |
| 0.0255855 | 1017.47 | 276.6 | 3.68 | A_52_P180130 | *Itgbl1* |
| 0.0023018 | 2798.21 | 759.89 | 3.68 | A_51_P212491 | *Pfkfb3* |
| 0.0054451 | 14.92 | 4.07 | 3.67 | A_52_P316841 | *A430057L12Rik* |
| 0.0029541 | 13.55 | 3.69 | 3.67 | A_51_P394281 | *Gbgt1* |
| 0.0050339 | 23.41 | 6.37 | 3.67 | A_52_P1187949 | *Trim5* |
| 0.0205994 | 26.64 | 7.28 | 3.66 | A_52_P368310 | *Nkain3* |
| 0.001668 | 279.23 | 76.2 | 3.66 | A_51_P411264 | *Txndc16* |
| 0.0076147 | 2721.09 | 742.55 | 3.66 | A_52_P650855 | *Myo1d* |
| 0.0016449 | 4.81 | 1.31 | 3.66 | A_51_P314547 | *Serpina3i* |
| 0.0260259 | 202.17 | 55.44 | 3.65 | A_51_P359891 | *Siglec1* |
| 0.0346364 | 19.85 | 5.43 | 3.65 | A_51_P469942 | *Myocd* |
| 0.0299062 | 638.43 | 175.61 | 3.64 | A_51_P249957 | *Fgf18* |
| 0.0015585 | 22.93 | 6.3 | 3.64 | A_51_P261204 | *Foxi2* |
| 0.0016147 | 26.46 | 7.3 | 3.63 | A_51_P383950 | *A_51_P383950* |
| 0.044472 | 159.11 | 43.8 | 3.63 | A_51_P431888 | *Radil* |
| 0.005104 | 63.67 | 17.52 | 3.63 | A_51_P509418 | *5430440P10Rik* |
| 0.0134907 | 161.65 | 44.7 | 3.62 | A_51_P181691 | *Nod2* |
| 0.0065473 | 196.17 | 54.24 | 3.62 | A_51_P214747 | *Parp12* |
| 0.0182666 | 7.2 | 1.99 | 3.62 | A_52_P155124 | *Zfp229* |
| 0.0429562 | 74.51 | 20.57 | 3.62 | A_51_P453818 | *Lzts1* |
| 0.0236142 | 132.66 | 36.8 | 3.61 | A_51_P517695 | *Ly6f* |
| 0.0309546 | 6.57 | 1.82 | 3.61 | A_52_P990 | *AK079832* |
| 0.0038587 | 363.67 | 101 | 3.6 | A_51_P241262 | *Arhgap15* |
| 0.0206434 | 251.16 | 70.01 | 3.59 | A_52_P1068292 | *AK050741* |
| 0.0228378 | 1544.78 | 429.97 | 3.59 | A_51_P238357 | *Plek* |
| 0.0324753 | 115.94 | 32.41 | 3.58 | A_52_P565396 | *NP062207* |
| 0.0013325 | 117.56 | 32.85 | 3.58 | A_51_P334785 | *Ifi209* |
| 0.0158275 | 271.98 | 76.26 | 3.57 | A_51_P264825 | *Lag3* |
| 0.0063416 | 1254.21 | 351.99 | 3.56 | A_51_P461319 | *Gatm* |
| 0.0032524 | 49.58 | 13.98 | 3.55 | A_52_P715416 | *AK053597* |
| 0.0226305 | 55.8 | 15.72 | 3.55 | A_51_P234359 | *Sct* |
| 0.0172207 | 72.52 | 20.41 | 3.55 | A_51_P260265 | *Hoxd4* |
| 0.040552 | 597.96 | 168.26 | 3.55 | A_52_P666442 | *ENSMUST00000184842* |
| 0.0057755 | 4.91 | 1.38 | 3.55 | A_51_P221294 | *A_51_P221294* |
| 0.0277594 | 21.63 | 6.12 | 3.54 | A_52_P1195763 | *AK030647* |
| 0.0106857 | 184.23 | 52.25 | 3.53 | A_52_P279294 | *Arxes2* |
| 0.0167012 | 99.46 | 28.16 | 3.53 | A_52_P543489 | *Slfn8* |
| 0.0175302 | 420.04 | 118.91 | 3.53 | A_51_P499838 | *Bst1* |
| 0.0392295 | 57.09 | 16.15 | 3.53 | A_51_P421538 | *Slc9b2* |
| 0.0262357 | 165.53 | 47.16 | 3.51 | A_51_P230583 | *Tnfrsf14* |
| 0.0043858 | 17449.78 | 4969.24 | 3.51 | A_51_P124748 | *Tgfb3* |
| 0.0402648 | 574.02 | 164.14 | 3.5 | A_51_P324303 | *Mylip* |
| 0.0334627 | 33.12 | 9.47 | 3.5 | A_51_P379318 | *Olfr127* |
| 0.0039879 | 8695.87 | 2486.33 | 3.5 | A_51_P296755 | *Fcgr1* |
| 0.0360907 | 166.97 | 47.88 | 3.49 | A_52_P607255 | *Slc2a13* |
| 0.0090645 | 7283.05 | 2088.47 | 3.49 | A_51_P108020 | *Zmynd15* |
| 0.0026716 | 1024.19 | 293.44 | 3.49 | A_51_P125842 | *Tmem51* |
| 0.0204543 | 231.96 | 66.41 | 3.49 | A_51_P138378 | *Fosb* |
| 0.005929 | 5.4 | 1.55 | 3.48 | A_52_P353551 | *4932407I05* |
| 0.0091508 | 10850.04 | 3129.7 | 3.47 | A_51_P277216 | *Ms4a7* |
| 0.0028338 | 324.43 | 93.52 | 3.47 | A_51_P513032 | *Trps1* |
| 0.0151539 | 454.33 | 130.9 | 3.47 | A_52_P565549 | *Npr2* |
| 0.0332873 | 18.58 | 5.35 | 3.47 | A_51_P305628 | *Atp8a2* |
| 0.0058203 | 62.88 | 18.1 | 3.47 | A_52_P738953 | *AK035454* |
| 0.0094492 | 89436.67 | 25864.97 | 3.46 | A_51_P423981 | *Ctss* |
| 0.0010233 | 136.27 | 39.5 | 3.45 | A_52_P55902 | *Il15ra* |
| 0.0069458 | 634.51 | 183.9 | 3.45 | A_52_P214740 | *Ube2l6* |
| 0.0024167 | 780.04 | 225.94 | 3.45 | A_51_P358037 | *Abi3bp* |
| 0.0111893 | 278.16 | 80.55 | 3.45 | A_51_P246924 | *Tppp3* |
| 0.0090171 | 29.5 | 8.54 | 3.45 | A_52_P359071 | *4930599N23Rik* |
| 0.0082756 | 172.75 | 50.41 | 3.43 | A_52_P536947 | *Cyfip2* |
| 0.0203931 | 310.29 | 90.38 | 3.43 | A_52_P574668 | *Nt5e* |
| 0.0061461 | 14.94 | 4.35 | 3.43 | A_51_P237865 | *Il4* |
| 0.005768 | 773.52 | 226.46 | 3.42 | A_51_P371912 | *Kctd12* |
| 0.0251492 | 38.57 | 11.29 | 3.42 | A_52_P258617 | *Cd180* |
| 0.0248011 | 16209.03 | 4734.1 | 3.42 | A_51_P181451 | *C1qa* |
| 0.0079477 | 5.04 | 1.48 | 3.41 | A_52_P1067648 | *AK031178* |
| 0.0063261 | 288.51 | 84.63 | 3.41 | A_52_P134762 | *ENSMUST00000138143* |
| 0.0083692 | 249.58 | 73.21 | 3.41 | A_52_P114905 | *Ifitm10* |
| 0.001968 | 1237.93 | 362.62 | 3.41 | A_51_P520966 | *Icosl* |
| 0.0194961 | 597.57 | 175.76 | 3.4 | A_52_P650028 | *Dock10* |
| 0.0028856 | 7447.69 | 2188.43 | 3.4 | A_52_P507382 | *Unc93b1* |
| 0.0044276 | 3112.38 | 919.39 | 3.39 | A_51_P469968 | *H2-M3* |
| 0.0035242 | 508.99 | 150.32 | 3.39 | A_51_P433870 | *Vegfc* |
| 0.023642 | 903.56 | 266.79 | 3.39 | A_51_P499918 | *Hck* |
| 0.0324058 | 14.47 | 4.27 | 3.39 | A_52_P30803 | *Gm973* |
| 0.0066237 | 27.83 | 8.23 | 3.38 | A_51_P479696 | *Cds1* |
| 0.0020893 | 1732.41 | 512.31 | 3.38 | A_52_P393314 | *P2rx7* |
| 0.0010382 | 99.93 | 29.68 | 3.37 | A_51_P261131 | *AK084367* |
| 0.0044805 | 26.71 | 7.93 | 3.37 | A_51_P109295 | *Pcdhb3* |
| 0.0019126 | 3703.49 | 1099.46 | 3.37 | A_51_P279841 | *Blnk* |
| 0.0019993 | 324.95 | 96.36 | 3.37 | A_51_P495581 | *Tlr1* |
| 0.0248317 | 259.1 | 76.82 | 3.37 | A_51_P108581 | *Grk3* |
| 0.0019235 | 15.82 | 4.69 | 3.37 | A_51_P448091 | *Zfp78* |
| 0.0019893 | 1422.12 | 421.47 | 3.37 | A_51_P372992 | *Vsir* |
| 0.0230525 | 107.39 | 32 | 3.36 | A_51_P448140 | *Cd22* |
| 0.0013018 | 26.49 | 7.89 | 3.36 | A_51_P193510 | *A430068E04Rik* |
| 0.0253505 | 30036.96 | 8942.61 | 3.36 | A_52_P582814 | *H2-Q4* |
| 0.0192599 | 19.98 | 5.94 | 3.36 | A_52_P31697 | *Adora3* |
| 0.0125729 | 113.37 | 33.89 | 3.35 | A_51_P497171 | *Ly9* |
| 0.0034834 | 2956.22 | 882.06 | 3.35 | A_52_P466090 | *Samhd1* |
| 0.0030953 | 2816.77 | 840.41 | 3.35 | A_52_P52618 | *Csf2rb* |
| 0.0012723 | 214.11 | 63.83 | 3.35 | A_52_P278853 | *Trim34a* |
| 0.0014467 | 860.9 | 256.63 | 3.35 | A_51_P147274 | *Clec4a3* |
| 0.0077404 | 276.83 | 82.99 | 3.34 | A_52_P507736 | *Itgb8* |
| 0.0140938 | 40.96 | 12.27 | 3.34 | A_51_P497741 | *Wdr95* |
| 0.0049933 | 2378.85 | 711.75 | 3.34 | A_51_P146753 | *Csf2rb2* |
| 0.0117506 | 975.42 | 291.71 | 3.34 | A_52_P99810 | *Cx3cr1* |
| 0.0075368 | 272.4 | 81.92 | 3.33 | A_51_P213205 | *Fam131a* |
| 0.0359196 | 150.37 | 45.16 | 3.33 | A_51_P217039 | *Gdf3* |
| 0.0494541 | 35.93 | 10.79 | 3.33 | A_52_P917015 | *Csprs* |
| 0.0127699 | 6.48 | 1.94 | 3.33 | A_52_P287692 | *Stk32c* |
| 0.0083954 | 728.46 | 219.68 | 3.32 | A_52_P173726 | *Vav1* |
| 0.0040633 | 205.01 | 61.82 | 3.32 | A_52_P279329 | *Acvrl1* |
| 0.039629 | 1212.34 | 365.52 | 3.32 | A_52_P350519 | *H2-Bl* |
| 0.0086451 | 11.28 | 3.4 | 3.32 | A_51_P326631 | *Dnah8* |
| 0.037068 | 200.75 | 60.4 | 3.32 | A_52_P452199 | *NAP050063-1* |
| 0.0031918 | 16.82 | 5.09 | 3.31 | A_52_P252070 | *Tmem150b* |
| 0.005116 | 4399.86 | 1331.26 | 3.31 | A_51_P462428 | *Galnt15* |
| 0.0086402 | 2986 | 903.08 | 3.31 | A_51_P152990 | *Grem2* |
| 0.0390943 | 671.48 | 203.01 | 3.31 | A_51_P124285 | *Nkd1* |
| 0.0411599 | 222.84 | 67.27 | 3.31 | A_52_P42245 | *Klrb1a* |
| 0.0236193 | 17578.52 | 5326.47 | 3.3 | A_51_P390538 | *Mpeg1* |
| 0.0029852 | 873.46 | 264.44 | 3.3 | A_52_P442567 | *Trim30a* |
| 0.0024379 | 2431.39 | 740.01 | 3.29 | A_52_P131466 | *Rac2* |
| 0.0377736 | 15015.26 | 4569.92 | 3.29 | A_51_P175018 | *Apcdd1* |
| 0.0023539 | 966.81 | 294.06 | 3.29 | A_51_P487244 | *Adamts7* |
| 0.0249043 | 23.57 | 7.16 | 3.29 | A_51_P209697 | *Atcay* |
| 0.0243026 | 1174.17 | 356.61 | 3.29 | A_52_P115787 | *Grb10* |
| 0.0403268 | 5.47 | 1.67 | 3.28 | A_52_P592305 | *Kcnc1* |
| 0.0182646 | 864.18 | 263.51 | 3.28 | A_52_P259126 | *Bub1* |
| 0.0298492 | 47.16 | 14.37 | 3.28 | A_51_P219266 | *Tmprss6* |
| 0.0283315 | 5.96 | 1.81 | 3.28 | A_52_P1180232 | *AK038475* |
| 0.0071322 | 29.28 | 8.97 | 3.27 | A_52_P101990 | *Hils1* |
| 0.0222344 | 62.16 | 19.04 | 3.27 | A_52_P440885 | *9630023C09Rik* |
| 0.0088619 | 8.13 | 2.49 | 3.27 | A_52_P80423 | *Dnajb7* |
| 0.0031795 | 296.38 | 90.76 | 3.27 | A_51_P372702 | *Il16* |
| 0.004219 | 1204.47 | 368.58 | 3.27 | A_51_P144143 | *A_51_P144143* |
| 0.0225516 | 27.2 | 8.32 | 3.27 | A_52_P29987 | *Prickle2* |
| 0.0231178 | 16.22 | 4.96 | 3.27 | A_51_P430014 | *Ptafr* |
| 0.0239356 | 255 | 77.97 | 3.27 | A_51_P451052 | *Pgm5* |
| 0.0301206 | 6992.41 | 2137.18 | 3.27 | A_51_P417612 | *H2-D4* |
| 0.0359308 | 9.73 | 2.97 | 3.27 | A_52_P604303 | *Ispd* |
| 0.0019137 | 242.88 | 74.57 | 3.26 | A_52_P386627 | *Irak3* |
| 0.0367478 | 59.91 | 18.36 | 3.26 | A_51_P389885 | *Spic* |
| 0.0150074 | 28581.99 | 8805.88 | 3.25 | A_51_P405476 | *Fcer1g* |
| 0.0033378 | 509.61 | 156.86 | 3.25 | A_52_P644210 | *Cul9* |
| 0.0485985 | 24.45 | 7.52 | 3.25 | A_52_P271572 | *Ece2* |
| 0.0279176 | 18036.54 | 5546.73 | 3.25 | A_51_P502456 | *H2-K2* |
| 0.0016977 | 55.44 | 17.04 | 3.25 | A_52_P664404 | *Zfp286* |
| 0.0362889 | 918.04 | 283.57 | 3.24 | A_52_P188827 | *Rasgef1b* |
| 0.0192541 | 919.49 | 283.82 | 3.24 | A_51_P505795 | *Tapbpl* |
| 0.002304 | 130.4 | 40.24 | 3.24 | A_52_P618569 | *Ikzf1* |
| 0.0243496 | 3790.24 | 1168.99 | 3.24 | A_51_P325904 | *Inhbb* |
| 0.0019134 | 1511.81 | 466.09 | 3.24 | A_51_P131358 | *Selplg* |
| 0.0084049 | 224.77 | 69.69 | 3.23 | A_51_P247694 | *Adgrg3* |
| 0.0054127 | 4349.07 | 1350.16 | 3.22 | A_52_P322141 | *Ccdc88b* |
| 0.0446885 | 599.5 | 186.1 | 3.22 | A_52_P361165 | *Ass1* |
| 0.0290314 | 10.81 | 3.37 | 3.21 | A_52_P609648 | *E130120C16Rik* |
| 0.0356921 | 3135.82 | 976.39 | 3.21 | A_51_P178411 | *NAP000023-001* |
| 0.0060649 | 146.03 | 45.7 | 3.2 | A_52_P64514 | *Herc6* |
| 0.0171412 | 107.42 | 33.59 | 3.2 | A_51_P190254 | *Scrn1* |
| 0.0027845 | 30644.99 | 9581.49 | 3.2 | A_51_P151126 | *Cd52* |
| 0.0163376 | 615.14 | 192.26 | 3.2 | A_51_P425047 | *Srpx2* |
| 0.019352 | 2448.01 | 764.33 | 3.2 | A_51_P479618 | *Rdh5* |
| 0.002945 | 118.14 | 37.08 | 3.19 | A_52_P295933 | *Eya4* |
| 0.0441438 | 27.41 | 8.59 | 3.19 | A_51_P328439 | *Zfp423* |
| 0.0021661 | 96.28 | 30.16 | 3.19 | A_52_P1021909 | *A_52_P1021909* |
| 0.0127302 | 1075.27 | 336.76 | 3.19 | A_51_P346668 | *Irf5* |
| 0.0028888 | 47739.2 | 14950.51 | 3.19 | A_51_P327796 | *Itgb5* |
| 0.0170524 | 17.42 | 5.49 | 3.18 | A_51_P249073 | *Speer4f1* |
| 0.0012526 | 1035.15 | 325.54 | 3.18 | A_51_P364694 | *Slfn5* |
| 0.0140948 | 100.35 | 31.53 | 3.18 | A_51_P218975 | *Kcns3* |
| 0.012164 | 2119.12 | 669.18 | 3.17 | A_52_P488092 | *Etl4* |
| 0.0091685 | 34.24 | 10.81 | 3.17 | A_51_P373142 | *AI854703* |
| 0.001562 | 5891.88 | 1859.88 | 3.17 | A_52_P195839 | *Ctsc* |
| 0.0210043 | 408.74 | 128.84 | 3.17 | A_51_P514085 | *Mx2* |
| 0.0089512 | 150.35 | 47.38 | 3.17 | A_52_P137193 | *AI662270* |
| 0.0037471 | 386.76 | 122.54 | 3.16 | A_52_P534844 | *Atp8a1* |
| 0.0180711 | 273.31 | 86.55 | 3.16 | A_51_P109258 | *Cys1* |
| 0.0039482 | 1609.06 | 509.38 | 3.16 | A_51_P496432 | *Acsl1* |
| 0.0078568 | 74.66 | 23.61 | 3.16 | A_52_P1139792 | *AK042676* |
| 0.0128378 | 258.96 | 81.87 | 3.16 | A_52_P672960 | *Camk4* |
| 0.0297178 | 134.74 | 42.57 | 3.16 | A_51_P444264 | *Rtn1* |
| 0.0355031 | 383.97 | 121.99 | 3.15 | A_51_P225933 | *Inpp4b* |
| 0.0413472 | 109.37 | 34.71 | 3.15 | A_51_P384136 | *Arsi* |
| 0.0022597 | 8329.57 | 2643.31 | 3.15 | A_51_P297679 | *Hcls1* |
| 0.0191862 | 148.55 | 47.28 | 3.14 | A_52_P680827 | *Abat* |
| 0.0079712 | 873.15 | 277.75 | 3.14 | A_51_P263471 | *Clec4n* |
| 0.0095764 | 834.95 | 265.58 | 3.14 | A_52_P336958 | *Jcad* |
| 0.0401128 | 40720.75 | 12949.86 | 3.14 | A_51_P444565 | *H2-T-ps* |
| 0.0030331 | 202.53 | 64.78 | 3.13 | A_51_P398191 | *Auts2* |
| 0.0097311 | 439.45 | 140.52 | 3.13 | A_51_P264227 | *Gpr160* |
| 0.0094782 | 17.83 | 5.7 | 3.13 | A_51_P167922 | *Tifab* |
| 0.0101193 | 159.84 | 51.07 | 3.13 | A_52_P287550 | *Krba1* |
| 0.0132152 | 93494.28 | 29863.83 | 3.13 | A_51_P198434 | *H2-K1* |
| 0.0154523 | 4042.74 | 1290.67 | 3.13 | A_51_P333253 | *Myo1g* |
| 0.0290957 | 1338.11 | 426.84 | 3.13 | A_51_P109508 | *Havcr2* |
| 0.0016047 | 1382.52 | 443.72 | 3.12 | A_51_P504337 | *Entpd1* |
| 0.019965 | 2539.87 | 814.79 | 3.12 | A_51_P511787 | *Casp4* |
| 0.0020377 | 673.7 | 215.84 | 3.12 | A_51_P339822 | *Arhgap4* |
| 0.0132385 | 49.15 | 15.74 | 3.12 | A_52_P497188 | *Prrg1* |
| 0.0113917 | 7520.84 | 2420.4 | 3.11 | A_51_P196925 | *Cx3cl1* |
| 0.0240679 | 292.28 | 94.01 | 3.11 | A_52_P132055 | *Cd33* |
| 0.016678 | 10.2 | 3.28 | 3.11 | A_52_P258796 | *ENSMUST00000191829* |
| 0.0039671 | 90.05 | 29.07 | 3.1 | A_51_P181751 | *Tmsb15l* |
| 0.0038657 | 43.09 | 13.9 | 3.1 | A_51_P451574 | *Acot1* |
| 0.0392614 | 13498.95 | 4353.65 | 3.1 | A_51_P351860 | *C1qb* |
| 0.0391065 | 880.36 | 283.93 | 3.1 | A_51_P135802 | *Mef2c* |
| 0.0026029 | 7164 | 2310.42 | 3.1 | A_51_P387123 | *Oasl2* |
| 0.014696 | 243.55 | 78.53 | 3.1 | A_51_P245631 | *Rftn2* |
| 0.0147854 | 145.27 | 46.81 | 3.1 | A_52_P54176 | *Axin2* |
| 0.045615 | 8176.53 | 2647.9 | 3.09 | A_51_P191463 | *H2-M2* |
| 0.0059653 | 850 | 275.01 | 3.09 | A_51_P362029 | *Cd48* |
| 0.0032653 | 2927.71 | 946.41 | 3.09 | A_51_P419768 | *Rgs2* |
| 0.0035634 | 3811.5 | 1231.77 | 3.09 | A_52_P249514 | *Ccl12* |
| 0.0296742 | 29196.48 | 9489.99 | 3.08 | A_52_P57582 | *Gm11127* |
| 0.0435442 | 7.97 | 2.59 | 3.08 | A_51_P223569 | *Ddx4* |
| 0.0165566 | 39.9 | 12.94 | 3.08 | A_52_P176634 | *Taf9b* |
| 0.0183702 | 27088.51 | 8781.56 | 3.08 | A_51_P517075 | *Serpinf1* |
| 0.0058938 | 12.24 | 3.99 | 3.07 | A_51_P234397 | *4930447K03Rik* |
| 0.0017975 | 6726.2 | 2189.71 | 3.07 | A_51_P341736 | *Mmp2* |
| 0.0015497 | 85.95 | 27.98 | 3.07 | A_51_P120738 | *P2ry14* |
| 0.0234381 | 225.21 | 73.7 | 3.06 | A_52_P1027961 | *Gm5086* |
| 0.0247751 | 697.58 | 228.14 | 3.06 | A_51_P242930 | *Lat2* |
| 0.0116704 | 1265.64 | 413.34 | 3.06 | A_51_P489887 | *Susd3* |
| 0.0092011 | 27.45 | 8.96 | 3.06 | A_52_P975 | *Mmp16* |
| 0.0029706 | 17.53 | 5.74 | 3.05 | A_51_P245865 | *C130098B18Rik* |
| 0.041789 | 40815.29 | 13364.26 | 3.05 | A_51_P262079 | *H2-Q7* |
| 0.023008 | 6.26 | 2.06 | 3.04 | A_52_P287576 | *AK036308* |
| 0.0049848 | 225.82 | 74.3 | 3.04 | A_52_P290020 | *Arhgef6* |
| 0.0247469 | 68.29 | 22.45 | 3.04 | A_51_P223686 | *Lmo2* |
| 0.0096848 | 97.77 | 32.13 | 3.04 | A_51_P215530 | *Rnf180* |
| 0.0228529 | 38.8 | 12.75 | 3.04 | A_51_P322954 | *Nfkbid* |
| 0.0116202 | 6.97 | 2.29 | 3.04 | A_51_P473259 | *Dpyd* |
| 0.0083643 | 6.03 | 1.98 | 3.04 | A_52_P482274 | *Zfp583* |
| 0.0489429 | 7.8 | 2.56 | 3.04 | A_51_P498602 | *NAP101353-1* |
| 0.0078038 | 98.33 | 32.5 | 3.03 | A_52_P141136 | *Fggy* |
| 0.0299937 | 141.41 | 46.72 | 3.03 | A_52_P382107 | *Nlrc5* |
| 0.0296263 | 24.11 | 7.96 | 3.03 | A_52_P309022 | *Dach1* |
| 0.0057586 | 5732.34 | 1890.87 | 3.03 | A_51_P316816 | *Uba7* |
| 0.0095427 | 157.37 | 51.88 | 3.03 | A_52_P95544 | *Scarf1* |
| 0.0128581 | 5 | 1.66 | 3.02 | A_52_P1123661 | *AK041851* |
| 0.007799 | 228.83 | 75.84 | 3.02 | A_52_P354744 | *Slc2a3* |
| 0.0249789 | 31.17 | 10.33 | 3.02 | A_52_P356343 | *Fam13a* |
| 0.0042241 | 97.72 | 32.37 | 3.02 | A_51_P510418 | *Aldh1b1* |
| 0.0117229 | 290.16 | 95.92 | 3.02 | A_52_P1093529 | *Pik3r5* |
| 0.0030611 | 6.28 | 2.09 | 3.01 | A_51_P418488 | *Robo2* |
| 0.0470862 | 7.58 | 2.52 | 3.01 | A_52_P923713 | *AK037394* |
| 0.0252721 | 579.25 | 193.35 | 3 | A_51_P358683 | *Gpr183* |
| 0.0053618 | 6197.68 | 2067.79 | 3 | A_52_P56751 | *Lcp1* |
| 0.0231495 | 26.32 | 8.77 | 3 | A_51_P302831 | *Brinp1* |
| 0.0070802 | 390.06 | 129.97 | 3 | A_51_P390285 | *Larp6* |
| 0.0164261 | 1140.52 | 379.85 | 3 | A_51_P459661 | *Lipa* |
| 0.0136989 | 1603.94 | 534 | 3 | A_52_P389595 | *Erap1* |
| 0.0024664 | 43.66 | 14.62 | 2.99 | A_52_P966930 | *LF204648* |
| 0.0219352 | 34.09 | 11.41 | 2.99 | A_52_P338550 | *Ank2* |
| 0.0309909 | 89.94 | 30.09 | 2.99 | A_52_P1172063 | *AK048142* |
| 0.0393408 | 45230.6 | 15131.98 | 2.99 | A_51_P219789 | *H2-Q2* |
| 0.0273226 | 1903.16 | 636.38 | 2.99 | A_52_P668543 | *Lair1* |
| 0.0431642 | 9.84 | 3.29 | 2.99 | A_51_P390954 | *Gm9961* |
| 0.015075 | 788.22 | 263.52 | 2.99 | A_52_P223495 | *Itga4* |
| 0.0376087 | 33491.98 | 11196.94 | 2.99 | A_51_P496997 | *H2-Q10* |
| 0.0388261 | 427.61 | 142.81 | 2.99 | A_52_P646832 | *Samsn1* |
| 0.0240605 | 783.14 | 263.16 | 2.98 | A_52_P89335 | *Tmie* |
| 0.0437858 | 24844.84 | 8345.22 | 2.98 | A_51_P400752 | *HQ258995* |
| 0.0112524 | 1431.39 | 480.5 | 2.98 | A_51_P382849 | *Emb* |
| 0.0033887 | 953.4 | 319.95 | 2.98 | A_51_P448664 | *Tbxas1* |
| 0.0270732 | 51.58 | 17.29 | 2.98 | A_52_P426768 | *Cited4* |
| 0.0282364 | 1938.83 | 649.83 | 2.98 | A_51_P153053 | *Smpdl3a* |
| 0.0030273 | 615.63 | 206.27 | 2.98 | A_51_P348372 | *Prex1* |
| 0.0412064 | 40.12 | 13.44 | 2.98 | A_51_P415369 | *Zbtb32* |
| 0.0046605 | 673.12 | 226.83 | 2.97 | A_52_P51355 | *Prkcb* |
| 0.0444653 | 156.33 | 52.65 | 2.97 | A_51_P499854 | *Ghr* |
| 0.0213708 | 136.84 | 46.08 | 2.97 | A_52_P613688 | *Elmo1* |
| 0.0039483 | 1985.44 | 668.53 | 2.97 | A_52_P114722 | *Ptpn6* |
| 0.0019992 | 8.41 | 2.83 | 2.97 | A_52_P1196662 | *AK086825* |
| 0.0021172 | 75.6 | 25.58 | 2.96 | A_52_P345946 | *Map3k9* |
| 0.0404222 | 2759.88 | 933.44 | 2.96 | A_51_P143190 | *Lyl1* |
| 0.0221122 | 475.95 | 160.87 | 2.96 | A_51_P492707 | *Ptpro* |
| 0.0432139 | 46.41 | 15.67 | 2.96 | A_52_P20727 | *Nhlrc1* |
| 0.0357019 | 65.66 | 22.16 | 2.96 | A_52_P130727 | *Ldoc1l* |
| 0.0158695 | 509.21 | 171.82 | 2.96 | A_51_P475156 | *Rnf19b* |
| 0.0055324 | 87.3 | 29.6 | 2.95 | A_51_P123731 | *Zfr2* |
| 0.0190288 | 2081.32 | 705.67 | 2.95 | A_51_P222077 | *3110062M04Rik* |
| 0.0094997 | 423.53 | 143.58 | 2.95 | A_51_P387810 | *Ifih1* |
| 0.0141717 | 383.55 | 129.94 | 2.95 | A_51_P130057 | *Ppm1k* |
| 0.0223553 | 61.56 | 20.85 | 2.95 | A_51_P150302 | *Crtam* |
| 0.0204961 | 88700.41 | 30033.74 | 2.95 | A_51_P129012 | *B2m* |
| 0.0019339 | 118.82 | 40.23 | 2.95 | A_52_P245806 | *Tsix* |
| 0.0318107 | 672.05 | 228.65 | 2.94 | A_52_P492411 | *Serpinb9* |
| 0.0045918 | 997.5 | 339.24 | 2.94 | A_52_P113190 | *Myo1f* |
| 0.0045831 | 727.64 | 247.41 | 2.94 | A_52_P471812 | *NAP061485-1* |
| 0.0201468 | 115.06 | 39.12 | 2.94 | A_52_P200458 | *Fut4* |
| 0.0023356 | 12.53 | 4.26 | 2.94 | A_52_P296338 | *Galnt18* |
| 0.0385963 | 35688.75 | 12130.12 | 2.94 | A_51_P102789 | *C1qc* |
| 0.0120586 | 19.07 | 6.48 | 2.94 | A_51_P143200 | *Tcstv3* |
| 0.0217437 | 16.14 | 5.52 | 2.93 | A_51_P450191 | *Art4* |
| 0.0134427 | 274.68 | 93.8 | 2.93 | A_51_P476529 | *Lhb* |
| 0.0036788 | 2489.48 | 849.77 | 2.93 | A_52_P677117 | *Ptprc* |
| 0.0035684 | 2738.25 | 934.48 | 2.93 | A_51_P119429 | *Nckap1l* |
| 0.0023137 | 27.69 | 9.44 | 2.93 | A_52_P654703 | *Trim69* |
| 0.0050437 | 334.91 | 114.17 | 2.93 | A_51_P285669 | *Pigz* |
| 0.0028923 | 29.36 | 10.05 | 2.92 | A_52_P762808 | *AK032340* |
| 0.0028009 | 2723.46 | 931.81 | 2.92 | A_51_P345422 | *Lyn* |
| 0.0085086 | 14.44 | 4.94 | 2.92 | A_52_P348250 | *Klhl29* |
| 0.0088394 | 779.86 | 266.75 | 2.92 | A_51_P449171 | *Birc3* |
| 0.007858 | 1120.56 | 385.58 | 2.91 | A_51_P237383 | *Rnase4* |
| 0.0349118 | 19.72 | 6.78 | 2.91 | A_51_P493816 | *Rab44* |
| 0.0217625 | 33.6 | 11.54 | 2.91 | A_51_P339724 | *2310079G19Rik* |
| 0.0326325 | 54836.81 | 18939.55 | 2.9 | A_52_P313279 | *H2-L* |
| 0.0172818 | 41.09 | 14.19 | 2.9 | A_52_P526565 | *Pde1a* |
| 0.0129738 | 606.16 | 209.32 | 2.9 | A_52_P299771 | *Bcl2a1c* |
| 0.0077082 | 1488.66 | 513.68 | 2.9 | A_51_P237856 | *Ifi203* |
| 0.0440933 | 27.59 | 9.52 | 2.9 | A_51_P506328 | *Cyp2j6* |
| 0.019974 | 3393.77 | 1170.35 | 2.9 | A_51_P337125 | *Inpp5d* |
| 0.0042778 | 52.65 | 18.15 | 2.9 | A_52_P803414 | *AK048115* |
| 0.0126197 | 144.15 | 49.69 | 2.9 | A_51_P362877 | *Il21r* |
| 0.0067757 | 2696.72 | 929.13 | 2.9 | A_51_P309158 | *Snx20* |
| 0.0309035 | 419.05 | 144.36 | 2.9 | A_51_P282557 | *C3ar1* |
| 0.005004 | 395.95 | 136.35 | 2.9 | A_51_P401683 | *Tm6sf1* |
| 0.0030825 | 1522.7 | 527.32 | 2.89 | A_51_P485260 | *Rgl1* |
| 0.0223554 | 11.79 | 4.08 | 2.89 | A_52_P1131922 | *AK047335* |
| 0.0311401 | 258.77 | 89.54 | 2.89 | A_51_P328489 | *1700025G04Rik* |
| 0.0345617 | 70.47 | 24.38 | 2.89 | A_51_P410900 | *Fut10* |
| 0.0097093 | 5656.34 | 1956.84 | 2.89 | A_51_P425772 | *Ckb* |
| 0.0368397 | 49.47 | 17.11 | 2.89 | A_51_P200447 | *Pkib* |
| 0.0049613 | 208.54 | 72.11 | 2.89 | A_52_P496924 | *Mgat4a* |
| 0.0038536 | 523.79 | 181.1 | 2.89 | A_52_P302345 | *Cyp4v3* |
| 0.0080091 | 704.87 | 244.98 | 2.88 | A_51_P383991 | *44443* |
| 0.0392593 | 2750.17 | 955.6 | 2.88 | A_52_P282741 | *Sdc3* |
| 0.0484205 | 43.76 | 15.19 | 2.88 | A_52_P763295 | *A630081D01Rik* |
| 0.0242339 | 3068.61 | 1064.14 | 2.88 | A_51_P375453 | *Wnt9a* |
| 0.0022429 | 241.22 | 83.63 | 2.88 | A_51_P199634 | *Rgag4* |
| 0.0370447 | 714 | 247.49 | 2.88 | A_51_P393426 | *Pros1* |
| 0.0420702 | 13.87 | 4.84 | 2.87 | A_52_P503614 | *Kcnj2* |
| 0.0360025 | 119.68 | 41.7 | 2.87 | A_52_P487599 | *Thnsl2* |
| 0.0023168 | 58.05 | 20.22 | 2.87 | A_52_P655136 | *Nlrc4* |
| 0.0138337 | 4162.19 | 1448.95 | 2.87 | A_51_P231184 | *Anpep* |
| 0.0052204 | 78.71 | 27.38 | 2.87 | A_52_P37826 | *Dapk1* |
| 0.0147217 | 33.8 | 11.8 | 2.86 | A_52_P446431 | *Mx1* |
| 0.0274802 | 17.14 | 5.98 | 2.86 | A_51_P217123 | *Rnf208* |
| 0.0031373 | 45.23 | 15.9 | 2.85 | A_52_P1187915 | *AK036708* |
| 0.0121707 | 5.72 | 2.01 | 2.85 | A_52_P762901 | *AK038254* |
| 0.038352 | 82.78 | 29.04 | 2.85 | A_52_P291736 | *Prkar1b* |
| 0.003956 | 44.84 | 15.73 | 2.85 | A_52_P485542 | *Hoxd8* |
| 0.0242946 | 31.36 | 11 | 2.85 | A_51_P251213 | *Cacna1d* |
| 0.0095525 | 127.12 | 44.54 | 2.85 | A_51_P175069 | *Adamts10* |
| 0.0137778 | 379.48 | 133.84 | 2.84 | A_51_P267494 | *Cdc42ep3* |
| 0.0037209 | 3468.06 | 1222.84 | 2.84 | A_51_P449777 | *Pmepa1* |
| 0.0196408 | 20.08 | 7.08 | 2.84 | A_51_P466270 | *Heph* |
| 0.0474516 | 50071.79 | 17639.45 | 2.84 | A_51_P237754 | *H2-T23* |
| 0.0105276 | 20.47 | 7.21 | 2.84 | A_52_P119792 | *Zfp709* |
| 0.002032 | 1222.48 | 430.54 | 2.84 | A_51_P108108 | *Lrrc25* |
| 0.0289046 | 39.61 | 13.95 | 2.84 | A_51_P101460 | *Dsp* |
| 0.01997 | 2623.75 | 923.5 | 2.84 | A_51_P137236 | *Olfm1* |
| 0.014326 | 27.84 | 9.79 | 2.84 | A_51_P479132 | *Zcchc3* |
| 0.0017864 | 92.8 | 32.62 | 2.84 | A_51_P277373 | *Slc8a1* |
| 0.0137847 | 409.16 | 144.56 | 2.83 | A_51_P301117 | *ENSMUST00000160674* |
| 0.0316832 | 1087.96 | 384.32 | 2.83 | A_51_P317181 | *Pcdhb21* |
| 0.0139287 | 172.1 | 60.75 | 2.83 | A_51_P114917 | *Dock2* |
| 0.019413 | 7004.07 | 2485.84 | 2.82 | A_51_P324690 | *Clec2d* |
| 0.0090584 | 286.65 | 101.69 | 2.82 | A_51_P317321 | *Iffo1* |
| 0.0292282 | 2645.07 | 937.78 | 2.82 | A_52_P199614 | *Wdfy4* |
| 0.0167966 | 505.99 | 179.39 | 2.82 | A_52_P676635 | *Trpm2* |
| 0.0293394 | 503.75 | 178.56 | 2.82 | A_52_P195260 | *Xxylt1* |
| 0.0354074 | 1493.38 | 529.13 | 2.82 | A_51_P311137 | *Sgce* |
| 0.0247725 | 6192.2 | 2205.29 | 2.81 | A_51_P484020 | *Cd300c2* |
| 0.0124128 | 3899.68 | 1387.26 | 2.81 | A_51_P130095 | *Fcgr2b* |
| 0.0432234 | 93.19 | 33.15 | 2.81 | A_52_P368306 | *Tmem100* |
| 0.0331601 | 2043.08 | 725.86 | 2.81 | A_52_P220783 | *Tmem229b* |
| 0.0122085 | 3.32 | 1.19 | 2.8 | A_51_P342031 | *Mkrn3* |
| 0.0255209 | 93.98 | 33.59 | 2.8 | A_52_P363110 | *Fgfr2* |
| 0.0161172 | 81.68 | 29.19 | 2.8 | A_52_P487686 | *BC100530* |
| 0.0178112 | 156.94 | 56.06 | 2.8 | A_51_P464300 | *Gdf1* |
| 0.0457405 | 1597.46 | 570.61 | 2.8 | A_51_P149699 | *P3h3* |
| 0.0083167 | 133745.16 | 47754.59 | 2.8 | A_52_P238027 | *Lyz2* |
| 0.006622 | 38.17 | 13.62 | 2.8 | A_52_P818794 | *Gm42027* |
| 0.0136554 | 9.62 | 3.43 | 2.8 | A_52_P707683 | *AK087612* |
| 0.0172784 | 159.34 | 57.15 | 2.79 | A_51_P465128 | *Chst15* |
| 0.0089297 | 412.87 | 148.03 | 2.79 | A_51_P391159 | *Ang* |
| 0.005619 | 9.01 | 3.23 | 2.79 | A_51_P217695 | *Cfap157* |
| 0.0184665 | 4729.71 | 1694.98 | 2.79 | A_51_P494241 | *Bcl2a1b* |
| 0.0124024 | 34.17 | 12.24 | 2.79 | A_51_P438657 | *Ipcef1* |
| 0.0249891 | 1573.75 | 563.63 | 2.79 | A_51_P303424 | *Itgax* |
| 0.0118566 | 446.01 | 159.67 | 2.79 | A_52_P2659 | *A_52_P2659* |
| 0.0307748 | 1370.8 | 493.97 | 2.78 | A_51_P487518 | *Casp12* |
| 0.0124007 | 12.97 | 4.66 | 2.78 | A_52_P78787 | *ENSMUST00000162126* |
| 0.0061034 | 167.72 | 60.26 | 2.78 | A_51_P383822 | *Evi2* |
| 0.0270009 | 32.82 | 11.86 | 2.77 | A_52_P194867 | *AK034320* |
| 0.0033014 | 585 | 211.26 | 2.77 | A_52_P244463 | *D16Ertd472e* |
| 0.014483 | 117.91 | 42.55 | 2.77 | A_51_P456699 | *Olfr812* |
| 0.0372735 | 7.87 | 2.84 | 2.77 | A_52_P396271 | *Reps2* |
| 0.0366577 | 11.81 | 4.26 | 2.77 | A_52_P58503 | *Armc2* |
| 0.0423937 | 37.49 | 13.6 | 2.76 | A_51_P182311 | *Clec2i* |
| 0.0181966 | 64.59 | 23.41 | 2.76 | A_51_P301435 | *B3galt5* |
| 0.006395 | 148.64 | 53.86 | 2.76 | A_52_P389484 | *Pag1* |
| 0.0040205 | 1376.56 | 498.75 | 2.76 | A_51_P411225 | *Wipf1* |
| 0.0035254 | 190.4 | 68.96 | 2.76 | A_52_P73475 | *Fam78a* |
| 0.0088806 | 2734.51 | 989.26 | 2.76 | A_51_P145453 | *Xist* |
| 0.0258052 | 98.1 | 35.69 | 2.75 | A_51_P234274 | *Ddx60* |
| 0.0112996 | 70236.89 | 25535.72 | 2.75 | A_52_P282058 | *Col8a1* |
| 0.022784 | 19.37 | 7.04 | 2.75 | A_52_P220912 | *P4ha3* |
| 0.0055467 | 168.13 | 61.46 | 2.74 | A_51_P240136 | *Gng8* |
| 0.0281512 | 75.42 | 27.54 | 2.74 | A_52_P498086 | *Spi1* |
| 0.0117053 | 1002.73 | 367.88 | 2.73 | A_52_P260994 | *Fgd2* |
| 0.0052427 | 34.31 | 12.58 | 2.73 | A_52_P184855 | *AK051864* |
| 0.0116676 | 2067.78 | 757.51 | 2.73 | A_51_P494403 | *Xaf1* |
| 0.0125934 | 114.34 | 41.88 | 2.73 | A_51_P507832 | *Pik3cg* |
| 0.0155875 | 536.63 | 196.29 | 2.73 | A_52_P585124 | *Cxcr4* |
| 0.0055656 | 42.35 | 15.6 | 2.72 | A_51_P315344 | *Zfp677* |
| 0.0128392 | 4322.94 | 1592.18 | 2.72 | A_51_P380178 | *Id3* |
| 0.0103799 | 241.05 | 88.7 | 2.72 | A_52_P1013232 | *A_52_P1013232* |
| 0.0048535 | 34.79 | 12.8 | 2.72 | A_52_P45616 | *Emilin3* |
| 0.0096486 | 427.12 | 156.96 | 2.72 | A_51_P352875 | *Lcp2* |
| 0.0080082 | 135.63 | 49.82 | 2.72 | A_52_P158282 | *St6galnac4* |
| 0.0065098 | 2872.44 | 1054.49 | 2.72 | A_52_P498355 | *Lyrm4* |
| 0.0194205 | 6988.47 | 2565.25 | 2.72 | A_51_P341465 | *Csf2ra* |
| 0.0033782 | 876.26 | 323.65 | 2.71 | A_51_P345975 | *Lpin3* |
| 0.0116173 | 1041.99 | 384.69 | 2.71 | A_51_P357533 | *Tmem44* |
| 0.0231552 | 223.16 | 82.34 | 2.71 | A_52_P487615 | *Fam105a* |
| 0.0045571 | 450.78 | 166.25 | 2.71 | A_51_P130079 | *Lrmp* |
| 0.0310387 | 1959.81 | 722.68 | 2.71 | A_51_P414079 | *Trp53i11* |
| 0.0109975 | 5156.03 | 1899.93 | 2.71 | A_51_P418116 | *Tmem119* |
| 0.0028126 | 52.02 | 19.27 | 2.7 | A_52_P1197192 | *Layn* |
| 0.0299536 | 760.95 | 281.7 | 2.7 | A_51_P227345 | *Dpep1* |
| 0.0154549 | 197.55 | 73.05 | 2.7 | A_51_P249313 | *Herc3* |
| 0.0034076 | 382.81 | 142.49 | 2.69 | A_51_P447866 | *Sash3* |
| 0.0326332 | 130.54 | 48.55 | 2.69 | A_52_P399536 | *NAP123700-1* |
| 0.0045557 | 9588.78 | 3563.71 | 2.69 | A_51_P236118 | *Ptpn18* |
| 0.0066897 | 615.47 | 228.7 | 2.69 | A_52_P51429 | *Dennd1c* |
| 0.0145015 | 2967.53 | 1109.32 | 2.68 | A_51_P288687 | *Serpinb6b* |
| 0.0062678 | 3875.05 | 1448.01 | 2.68 | A_51_P437426 | *Nrros* |
| 0.0175091 | 704.93 | 263.4 | 2.68 | A_51_P150678 | *Tnfaip8l2* |
| 0.0244282 | 287.7 | 107.36 | 2.68 | A_51_P199425 | *Glcci1* |
| 0.0312036 | 11732.34 | 4378.1 | 2.68 | A_51_P357606 | *Phyhd1* |
| 0.0090121 | 391.53 | 146.02 | 2.68 | A_51_P444934 | *Syk* |
| 0.0229534 | 11.55 | 4.3 | 2.68 | A_51_P233583 | *Clec1b* |
| 0.0036187 | 373.45 | 140 | 2.67 | A_51_P158073 | *A230050P20Rik* |
| 0.0191131 | 11.29 | 4.23 | 2.67 | A_51_P173858 | *Apba2* |
| 0.0052497 | 1775.69 | 665.22 | 2.67 | A_52_P263518 | *Gng2* |
| 0.0098244 | 472.44 | 176.9 | 2.67 | A_51_P207031 | *Ncf1* |
| 0.0260088 | 17448.13 | 6527.85 | 2.67 | A_51_P261517 | *Tyrobp* |
| 0.0142436 | 439.6 | 164.36 | 2.67 | A_51_P145883 | *Btk* |
| 0.0133895 | 5049.83 | 1901.51 | 2.66 | A_52_P387237 | *Dhrs3* |
| 0.0122055 | 1400.73 | 527.36 | 2.66 | A_51_P501858 | *Plekha4* |
| 0.00406 | 654.09 | 246.25 | 2.66 | A_51_P302749 | *Trpv2* |
| 0.0295892 | 23.58 | 8.87 | 2.66 | A_51_P351015 | *Lta* |
| 0.0210278 | 1785.05 | 671.47 | 2.66 | A_51_P222467 | *Abcg1* |
| 0.0141994 | 174.75 | 65.69 | 2.66 | A_52_P50063 | *Trim12c* |
| 0.0394488 | 811.84 | 304.7 | 2.66 | A_52_P441974 | *Evc2* |
| 0.0193515 | 11.46 | 4.33 | 2.65 | A_52_P71695 | *BC043934* |
| 0.0115341 | 357 | 134.79 | 2.65 | A_52_P144297 | *Tspyl3* |
| 0.0093756 | 29582.02 | 11156.99 | 2.65 | A_51_P519251 | *Nupr1* |
| 0.0201502 | 26.78 | 10.1 | 2.65 | A_52_P84487 | *Ankrd6* |
| 0.0095042 | 151.82 | 57.25 | 2.65 | A_51_P384894 | *Csta1* |
| 0.0025109 | 327.55 | 123.51 | 2.65 | A_52_P35534 | *E230029C05Rik* |
| 0.0436174 | 700.21 | 264 | 2.65 | A_52_P223809 | *Dhx58* |
| 0.008084 | 352.73 | 132.98 | 2.65 | A_52_P374556 | *Lgi4* |
| 0.0099791 | 5963.55 | 2247.86 | 2.65 | A_51_P377452 | *Ncf4* |
| 0.0140924 | 162.24 | 61.51 | 2.64 | A_51_P255853 | *Tal1* |
| 0.0276557 | 420.38 | 159.3 | 2.64 | A_51_P445166 | *C1qtnf1* |
| 0.0316395 | 24.57 | 9.31 | 2.64 | A_51_P227445 | *Gm32819* |
| 0.0037166 | 120.61 | 45.7 | 2.64 | A_51_P167551 | *Fam214a* |
| 0.0059465 | 220.59 | 83.57 | 2.64 | A_52_P60353 | *Greb1l* |
| 0.0256025 | 199.62 | 75.53 | 2.64 | A_52_P462296 | *Cpeb1* |
| 0.0212387 | 20.78 | 7.86 | 2.64 | A_51_P179881 | *Lca5l* |
| 0.005945 | 1593.78 | 606.36 | 2.63 | A_51_P248629 | *Arhgap25* |
| 0.0147663 | 135.52 | 51.53 | 2.63 | A_51_P502906 | *ENSMUST00000162314* |
| 0.0035449 | 9036.98 | 3432.64 | 2.63 | A_51_P488340 | *Crlf2* |
| 0.0032927 | 6.17 | 2.34 | 2.63 | A_52_P1172401 | *AK038162* |
| 0.0477145 | 4525.85 | 1730.29 | 2.62 | A_51_P195506 | *Csf1* |
| 0.0290728 | 57.77 | 22.08 | 2.62 | A_52_P979803 | *AK051975* |
| 0.0303823 | 909.41 | 347.43 | 2.62 | A_52_P513177 | *Sla* |
| 0.0041863 | 527.51 | 201.52 | 2.62 | A_51_P463120 | *Hacd4* |
| 0.0167961 | 2732.64 | 1043.24 | 2.62 | A_51_P233160 | *Lysmd2* |
| 0.011922 | 1142.3 | 435.87 | 2.62 | A_51_P340027 | *Pappa* |
| 0.0176677 | 42730.61 | 16297.31 | 2.62 | A_52_P570266 | *Psmb10* |
| 0.0214739 | 879.72 | 335.44 | 2.62 | A_51_P121031 | *44256* |
| 0.0166343 | 28.87 | 11 | 2.62 | A_52_P1004880 | *A_52_P1004880* |
| 0.0147341 | 428.63 | 164.46 | 2.61 | A_52_P618427 | *Pdgfb* |
| 0.0086163 | 252.15 | 96.5 | 2.61 | A_51_P356871 | *ENSMUST00000120523* |
| 0.0268872 | 8.78 | 3.36 | 2.61 | A_51_P345867 | *Hsf4* |
| 0.0480017 | 89.63 | 34.3 | 2.61 | A_51_P301636 | *Kazn* |
| 0.0045957 | 157.01 | 60.5 | 2.6 | A_51_P269166 | *Mmp19* |
| 0.0468629 | 37.83 | 14.57 | 2.6 | A_51_P465147 | *3632451O06Rik* |
| 0.0173365 | 5.74 | 2.21 | 2.6 | A_52_P811275 | *AK040293* |
| 0.0092387 | 25.43 | 9.79 | 2.6 | A_52_P148389 | *AK040902* |
| 0.0165301 | 61.94 | 23.83 | 2.6 | A_51_P171772 | *Bcl11b* |
| 0.012788 | 512.58 | 197.06 | 2.6 | A_52_P527625 | *Colec12* |
| 0.0086342 | 5086.91 | 1953.58 | 2.6 | A_52_P154800 | *Pla2g16* |
| 0.0211814 | 1321.11 | 507.26 | 2.6 | A_51_P147942 | *Igf1* |
| 0.0248926 | 3159.76 | 1221.64 | 2.59 | A_51_P225808 | *Stat2* |
| 0.0248827 | 26.02 | 10.05 | 2.59 | A_52_P295893 | *C130083B21Rik* |
| 0.0180566 | 188.83 | 72.92 | 2.59 | A_52_P683580 | *Tbc1d9* |
| 0.0060067 | 437.52 | 168.88 | 2.59 | A_52_P296074 | *Zfp945* |
| 0.0046054 | 3.42 | 1.32 | 2.59 | A_52_P322941 | *Gm11992* |
| 0.0086053 | 1973.06 | 761.08 | 2.59 | A_52_P599264 | *Mdfic* |
| 0.0044486 | 55.8 | 21.52 | 2.59 | A_51_P466633 | *Slc16a7* |
| 0.019387 | 17.46 | 6.73 | 2.59 | A_52_P672903 | *ENSMUST00000103746* |
| 0.0484919 | 387.55 | 149.38 | 2.59 | A_51_P191199 | *Gypc* |
| 0.0232544 | 3.4 | 1.32 | 2.58 | A_51_P379698 | *Fxyd7* |
| 0.0071236 | 45.75 | 17.76 | 2.58 | A_51_P471458 | *Sult5a1* |
| 0.0453189 | 670.24 | 260.07 | 2.58 | A_51_P252725 | *Gpc4* |
| 0.0055634 | 2286.74 | 886.92 | 2.58 | A_51_P401668 | *Laptm5* |
| 0.0072982 | 40.76 | 15.8 | 2.58 | A_51_P391716 | *Ermap* |
| 0.0027166 | 50.29 | 19.47 | 2.58 | A_51_P253883 | *Fam49a* |
| 0.0266205 | 7.83 | 3.03 | 2.58 | A_52_P602559 | *TC1637468* |
| 0.0131233 | 52.03 | 20.28 | 2.57 | A_51_P269385 | *AK081699* |
| 0.0030138 | 3.62 | 1.41 | 2.57 | A_52_P1142105 | *ENSMUST00000103464* |
| 0.0107061 | 280.9 | 109.39 | 2.57 | A_52_P51503 | *Abca9* |
| 0.0163902 | 7.48 | 2.91 | 2.57 | A_51_P290526 | *Abcd2* |
| 0.0123897 | 2118.16 | 824.02 | 2.57 | A_51_P113144 | *Ap1s2* |
| 0.009826 | 7.43 | 2.89 | 2.57 | A_52_P407049 | *Hoxd10* |
| 0.0099514 | 12.19 | 4.74 | 2.57 | A_52_P549754 | *A_52_P549754* |
| 0.0059369 | 12.63 | 4.91 | 2.57 | A_51_P358714 | *AK031433* |
| 0.0185526 | 603.4 | 234.4 | 2.57 | A_51_P407351 | *Ly75* |
| 0.0056138 | 311.12 | 121.51 | 2.56 | A_52_P827934 | *AK084349* |
| 0.0171939 | 19671.5 | 7673.67 | 2.56 | A_52_P220879 | *Tgm2* |
| 0.0186839 | 55.91 | 21.8 | 2.56 | A_52_P171197 | *Gfra4* |
| 0.0051131 | 287.71 | 113.05 | 2.55 | A_52_P555262 | *Asrgl1* |
| 0.0161303 | 153.76 | 60.33 | 2.55 | A_52_P158431 | *Tshz2* |
| 0.011633 | 1515.69 | 594.25 | 2.55 | A_51_P520879 | *Sh3d19* |
| 0.0167217 | 115.24 | 45.14 | 2.55 | A_51_P399896 | *Matk* |
| 0.0065809 | 1145.61 | 448.43 | 2.55 | A_51_P479321 | *Acss1* |
| 0.0190586 | 50.58 | 19.95 | 2.54 | A_51_P110323 | *Gna14* |
| 0.0195932 | 16.79 | 6.62 | 2.54 | A_52_P1155656 | *AK039972* |
| 0.0472588 | 185.3 | 73.04 | 2.54 | A_51_P201709 | *Ggt5* |
| 0.0208764 | 4.29 | 1.69 | 2.54 | A_51_P301394 | *Gspt2* |
| 0.029399 | 2250.7 | 886.15 | 2.54 | A_51_P438821 | *Pycard* |
| 0.0412005 | 953.66 | 375.32 | 2.54 | A_51_P202911 | *Spats2l* |
| 0.0240222 | 794.26 | 312.3 | 2.54 | A_51_P507053 | *Slc38a1* |
| 0.0234305 | 21.11 | 8.3 | 2.54 | A_51_P333712 | *Pde7b* |
| 0.0195919 | 13.43 | 5.28 | 2.54 | A_52_P963811 | *AK037186* |
| 0.0042664 | 2898.75 | 1147.95 | 2.53 | A_51_P305583 | *Sp100* |
| 0.0039911 | 1337.56 | 529.61 | 2.53 | A_51_P272876 | *Fam46a* |
| 0.0395057 | 3744.89 | 1481.65 | 2.53 | A_51_P364600 | *Fyb* |
| 0.0034289 | 28357.02 | 11216.3 | 2.53 | A_51_P304500 | *Tapbp* |
| 0.0089726 | 426.3 | 168.57 | 2.53 | A_51_P407227 | *Gimap6* |
| 0.0290676 | 29.46 | 11.64 | 2.53 | A_51_P144303 | *Ptger1* |
| 0.025862 | 42.65 | 16.85 | 2.53 | A_52_P301486 | *B930068K11Rik* |
| 0.0056468 | 11191.97 | 4420.34 | 2.53 | A_52_P244682 | *Rab7b* |
| 0.0047702 | 4.06 | 1.6 | 2.53 | A_51_P301236 | *Olfr686* |
| 0.0413919 | 194.06 | 77.15 | 2.52 | A_51_P248819 | *Bambi* |
| 0.003822 | 1330.99 | 527.95 | 2.52 | A_51_P371091 | *Rcsd1* |
| 0.0195387 | 45.14 | 17.9 | 2.52 | A_52_P354752 | *Rnf144b* |
| 0.0124386 | 677.84 | 268.74 | 2.52 | A_51_P342652 | *Cd79b* |
| 0.0262767 | 112.59 | 44.63 | 2.52 | A_51_P435922 | *Rsph9* |
| 0.010225 | 1459.53 | 578.54 | 2.52 | A_51_P297105 | *Ucp2* |
| 0.0385064 | 2133.91 | 845.53 | 2.52 | A_51_P381558 | *Rasa4* |
| 0.0171281 | 1434.7 | 572.66 | 2.51 | A_52_P665675 | *Abca1* |
| 0.0114382 | 673.19 | 268.49 | 2.51 | A_51_P420918 | *Ly6i* |
| 0.0486412 | 416.14 | 165.83 | 2.51 | A_51_P254855 | *Ptgs2* |
| 0.0034218 | 598.54 | 238.43 | 2.51 | A_52_P640484 | *Trim21* |
| 0.0450129 | 56.93 | 22.67 | 2.51 | A_51_P243808 | *Traf3ip3* |
| 0.0267418 | 22.42 | 8.92 | 2.51 | A_51_P498483 | *Frmd3* |
| 0.0040776 | 9739.49 | 3902.22 | 2.5 | A_51_P298570 | *Eif4a2* |
| 0.0112065 | 287.97 | 115.27 | 2.5 | A_51_P110672 | *Mst1r* |
| 0.0366699 | 23.81 | 9.52 | 2.5 | A_52_P39614 | *Vmn2r30* |
| 0.0038487 | 1750.88 | 699.38 | 2.5 | A_51_P502661 | *Vwa5a* |
| 0.0385578 | 2498.46 | 1003.95 | 2.49 | A_51_P419147 | *Lpxn* |
| 0.0037761 | 27.41 | 11.01 | 2.49 | A_51_P268469 | *Pcdhb19* |
| 0.0278691 | 747.89 | 300.29 | 2.49 | A_51_P345649 | *Pdgfra* |
| 0.005045 | 2368.07 | 949.25 | 2.49 | A_52_P162486 | *Bgn* |
| 0.0043184 | 2862.44 | 1147.42 | 2.49 | A_52_P495869 | *Mafb* |
| 0.0035002 | 19.07 | 7.71 | 2.48 | A_51_P118046 | *Cyp2r1* |
| 0.017768 | 869.45 | 351.07 | 2.48 | A_51_P409173 | *Ldhb* |
| 0.0251603 | 209.25 | 84.49 | 2.48 | A_52_P102931 | *Gimap5* |
| 0.0060602 | 4311.41 | 1740.24 | 2.48 | A_52_P170635 | *Sirpa* |
| 0.0071687 | 145.44 | 58.62 | 2.48 | A_51_P395864 | *Srgap1* |
| 0.0258871 | 678.61 | 273.42 | 2.48 | A_51_P367240 | *Tspan33* |
| 0.0041224 | 708.3 | 285.04 | 2.48 | A_51_P446886 | *5730409E04Rik* |
| 0.0238804 | 12.55 | 5.05 | 2.48 | A_51_P323518 | *Cend1* |
| 0.0036684 | 366.19 | 148.51 | 2.47 | A_51_P459240 | *Gstk1* |
| 0.0115797 | 884.78 | 358.03 | 2.47 | A_51_P402909 | *Ncf2* |
| 0.0033294 | 292.51 | 118.35 | 2.47 | A_51_P337771 | *Pcdhb17* |
| 0.0281766 | 6.83 | 2.76 | 2.47 | A_52_P561088 | *AK046555* |
| 0.0275759 | 42.02 | 16.98 | 2.47 | A_51_P355852 | *Prkch* |
| 0.0257798 | 705.91 | 287.43 | 2.46 | A_52_P157150 | *Rassf4* |
| 0.0357394 | 72215.38 | 29384.25 | 2.46 | A_51_P472292 | *Igfbp7* |
| 0.0051107 | 1123.78 | 456.95 | 2.46 | A_51_P327121 | *Was* |
| 0.0048182 | 82.75 | 33.64 | 2.46 | A_51_P274124 | *Mansc1* |
| 0.0042754 | 3756.03 | 1524.34 | 2.46 | A_52_P46447 | *Crim1* |
| 0.039162 | 88.38 | 35.86 | 2.46 | A_51_P386539 | *Rnf125* |
| 0.043733 | 37.91 | 15.38 | 2.46 | A_52_P1133481 | *ENSMUST00000163885* |
| 0.0103176 | 2802.98 | 1143.23 | 2.45 | A_51_P266546 | *Zc2hc1a* |
| 0.0248207 | 250.53 | 102.14 | 2.45 | A_52_P627677 | *NAP123578-1* |
| 0.0117356 | 10.25 | 4.21 | 2.44 | A_52_P989397 | *TC1685454* |
| 0.0049481 | 3349.61 | 1374.6 | 2.44 | A_52_P635338 | *Fes* |
| 0.045446 | 593.52 | 243.36 | 2.44 | A_51_P394558 | *Dchs1* |
| 0.0384782 | 8.16 | 3.34 | 2.44 | A_51_P374737 | *Ovch2* |
| 0.0153383 | 474.63 | 194.22 | 2.44 | A_51_P439966 | *Ggh* |
| 0.0496539 | 1444.3 | 595.53 | 2.43 | A_52_P337357 | *Oas1a* |
| 0.0122379 | 3229.06 | 1331.12 | 2.43 | A_51_P501757 | *Rgmb* |
| 0.0300354 | 505.79 | 208.29 | 2.43 | A_52_P274960 | *Arhgap30* |
| 0.0117364 | 80.59 | 33.18 | 2.43 | A_52_P648688 | *Zc3h12d* |
| 0.0456096 | 197.59 | 81.35 | 2.43 | A_51_P116651 | *Dpt* |
| 0.006986 | 110.36 | 45.43 | 2.43 | A_52_P27117 | *Ago4* |
| 0.0497507 | 327.79 | 134.91 | 2.43 | A_51_P171288 | *Gli3* |
| 0.0056987 | 19.61 | 8.07 | 2.43 | A_51_P362104 | *Enpp5* |
| 0.0454687 | 602.03 | 247.69 | 2.43 | A_52_P108845 | *Clip3* |
| 0.0245522 | 254.65 | 104.67 | 2.43 | A_51_P393078 | *Tigd2* |
| 0.0214456 | 365.57 | 150.14 | 2.43 | A_52_P670263 | *Tfpi* |
| 0.0040189 | 1646.7 | 676.29 | 2.43 | A_51_P505538 | *Hfe* |
| 0.0486572 | 136.51 | 56.48 | 2.42 | A_51_P517430 | *Cd1d1* |
| 0.0186464 | 207.59 | 85.76 | 2.42 | A_51_P275407 | *Phactr2* |
| 0.0048812 | 263.18 | 108.67 | 2.42 | A_51_P326994 | *Calhm2* |
| 0.0099504 | 6652.79 | 2744.65 | 2.42 | A_51_P300277 | *Coro1a* |
| 0.0055265 | 260.67 | 108.25 | 2.41 | A_51_P378550 | *Oplah* |
| 0.0045169 | 86.16 | 35.77 | 2.41 | A_51_P191865 | *Lama2* |
| 0.0061168 | 219.42 | 91.08 | 2.41 | A_52_P308413 | *1810011H11Rik* |
| 0.0040186 | 2708.36 | 1124.06 | 2.41 | A_51_P514623 | *Cd302* |
| 0.0212187 | 44.72 | 18.56 | 2.41 | A_51_P133992 | *Smim24* |
| 0.0404865 | 21.26 | 8.82 | 2.41 | A_51_P308279 | *St18* |
| 0.0278864 | 324.18 | 134.49 | 2.41 | A_52_P643832 | *Pcp2* |
| 0.0315442 | 48.98 | 20.29 | 2.41 | A_52_P891260 | *Gm9905* |
| 0.0060848 | 2813.38 | 1174.67 | 2.4 | A_51_P182471 | *Celf2* |
| 0.0057764 | 331.74 | 138.35 | 2.4 | A_52_P40954 | *Dubr* |
| 0.0281798 | 14927.23 | 6221.9 | 2.4 | A_52_P463977 | *Tmem140* |
| 0.0108184 | 207.06 | 86.27 | 2.4 | A_52_P98778 | *Ang4* |
| 0.008504 | 459.79 | 191.43 | 2.4 | A_52_P626329 | *NAP026388-1* |
| 0.0051664 | 22.11 | 9.2 | 2.4 | A_51_P315411 | *Epb41l3* |
| 0.0182167 | 10.88 | 4.52 | 2.4 | A_52_P205810 | *Gcsam* |
| 0.0084241 | 111.26 | 46.59 | 2.39 | A_51_P179504 | *Ang3* |
| 0.0242091 | 3.8 | 1.59 | 2.39 | A_52_P653448 | *CA559477* |
| 0.0180361 | 5129.16 | 2144.74 | 2.39 | A_51_P146168 | *Col12a1* |
| 0.0274196 | 28.15 | 11.77 | 2.39 | A_52_P267843 | *Clec4a1* |
| 0.0049057 | 776.02 | 324.42 | 2.39 | A_51_P316616 | *Smagp* |
| 0.043331 | 202.54 | 84.66 | 2.39 | A_51_P338746 | *Prdm1* |
| 0.0274488 | 1171.17 | 489.45 | 2.39 | A_51_P157083 | *Gas1* |
| 0.0245888 | 373.96 | 156.18 | 2.39 | A_51_P114616 | *Batf* |
| 0.0099576 | 408.58 | 170.62 | 2.39 | A_52_P633597 | *Rftn1* |
| 0.0183003 | 15731.98 | 6620.72 | 2.38 | A_51_P180492 | *Dbp* |
| 0.00704 | 221.91 | 93.34 | 2.38 | A_52_P507498 | *Plxnc1* |
| 0.0135011 | 84.14 | 35.39 | 2.38 | A_51_P350048 | *Gstt2* |
| 0.0371879 | 8.35 | 3.51 | 2.38 | A_52_P480309 | *4930470G03Rik* |
| 0.0090806 | 25.96 | 10.9 | 2.38 | A_52_P474294 | *Iqgap2* |
| 0.0138187 | 536.33 | 225.01 | 2.38 | A_51_P402908 | *A_51_P402908* |
| 0.0498512 | 3.89 | 1.65 | 2.37 | A_51_P162023 | *Vmn1r76* |
| 0.0059191 | 31.32 | 13.24 | 2.37 | A_51_P176893 | *Tlr8* |
| 0.0051976 | 45.27 | 19.13 | 2.37 | A_51_P474334 | *Pfpl* |
| 0.0054841 | 312.19 | 131.82 | 2.37 | A_52_P254095 | *Cd200* |
| 0.0183423 | 212.19 | 89.58 | 2.37 | A_51_P434483 | *Hpn* |
| 0.0038706 | 709.68 | 299.49 | 2.37 | A_51_P483220 | *Impact* |
| 0.0057606 | 128.36 | 54.16 | 2.37 | A_52_P971828 | *AK050801* |
| 0.0057775 | 2646.98 | 1114.72 | 2.37 | A_51_P473888 | *Il6st* |
| 0.0140324 | 701.57 | 297.89 | 2.36 | A_52_P618417 | *Bin2* |
| 0.0125883 | 20881.61 | 8859.51 | 2.36 | A_51_P265495 | *Ly6a* |
| 0.0091196 | 52.3 | 22.18 | 2.36 | A_52_P127069 | *Sgk3* |
| 0.0208299 | 65.91 | 27.95 | 2.36 | A_52_P1147740 | *AK034071* |
| 0.0069725 | 520.64 | 220.76 | 2.36 | A_51_P360918 | *Ehd3* |
| 0.0042617 | 17.49 | 7.41 | 2.36 | A_51_P351217 | *Rab39* |
| 0.0292994 | 449.53 | 190.3 | 2.36 | A_51_P464710 | *Adap2* |
| 0.0038411 | 1069.1 | 452.29 | 2.36 | A_51_P161724 | *Ophn1* |
| 0.0453222 | 811.1 | 345.39 | 2.35 | A_51_P315890 | *Kcnk6* |
| 0.0138729 | 2.68 | 1.14 | 2.35 | A_52_P505697 | *TC1650694* |
| 0.0099961 | 94.08 | 40.01 | 2.35 | A_52_P417846 | *Tbkbp1* |
| 0.0057821 | 481.22 | 204.62 | 2.35 | A_52_P118521 | *Pilra* |
| 0.0039185 | 17409.28 | 7401.19 | 2.35 | A_51_P183746 | *Prrx2* |
| 0.0353353 | 1371.65 | 583.07 | 2.35 | A_51_P364391 | *Mvb12b* |
| 0.0056531 | 30.26 | 12.86 | 2.35 | A_51_P183882 | *Tnfsf12* |
| 0.0056914 | 8972.95 | 3811.85 | 2.35 | A_52_P597634 | *Fzd1* |
| 0.0043881 | 573.41 | 243.54 | 2.35 | A_51_P483617 | *0610040J01Rik* |
| 0.0066489 | 143.61 | 61.5 | 2.34 | A_51_P240421 | *Pcdhb14* |
| 0.0091125 | 383.85 | 164.31 | 2.34 | A_52_P731686 | *AK085106* |
| 0.0040638 | 817.32 | 349.53 | 2.34 | A_51_P339023 | *Rassf2* |
| 0.0078056 | 12104.8 | 5171.25 | 2.34 | A_52_P209184 | *Csrp1* |
| 0.0267793 | 147.47 | 62.98 | 2.34 | A_52_P652442 | *Rora* |
| 0.0057628 | 539.92 | 230.56 | 2.34 | A_51_P299805 | *Slc46a3* |
| 0.0270342 | 7.26 | 3.1 | 2.34 | A_51_P357622 | *Olfr638* |
| 0.0278795 | 2668.93 | 1139.39 | 2.34 | A_51_P209771 | *Prrx1* |
| 0.034948 | 13.17 | 5.62 | 2.34 | A_51_P464892 | *4930426L09Rik* |
| 0.0169895 | 1490 | 635.55 | 2.34 | A_51_P431433 | *Tmem2* |
| 0.023944 | 146.58 | 62.99 | 2.33 | A_51_P464911 | *Msrb2* |
| 0.0051366 | 17.06 | 7.33 | 2.33 | A_51_P485731 | *Sytl1* |
| 0.0197144 | 19.83 | 8.52 | 2.33 | A_52_P413646 | *Bmp6* |
| 0.0217589 | 1185.48 | 509.22 | 2.33 | A_51_P255456 | *Cyp1b1* |
| 0.0200391 | 1508.03 | 647.45 | 2.33 | A_51_P218984 | *Parp9* |
| 0.0111713 | 7.04 | 3.02 | 2.33 | A_52_P770910 | *AK050314* |
| 0.0223499 | 872.54 | 373.78 | 2.33 | A_51_P275496 | *Gm39749* |
| 0.0222254 | 198.49 | 85.73 | 2.32 | A_51_P403413 | *Pcdhb6* |
| 0.0041438 | 159.64 | 68.91 | 2.32 | A_51_P302453 | *Lhfpl2* |
| 0.0296027 | 3.88 | 1.67 | 2.32 | A_51_P328537 | *Eda* |
| 0.0087374 | 596.1 | 256.56 | 2.32 | A_51_P468240 | *Dennd4a* |
| 0.0097389 | 68.37 | 29.66 | 2.31 | A_52_P189246 | *Mtm1* |
| 0.0154784 | 90.14 | 39.1 | 2.31 | A_51_P237307 | *Plekhg1* |
| 0.0206847 | 4701.05 | 2038.55 | 2.31 | A_51_P220343 | *Wisp1* |
| 0.0061169 | 6.02 | 2.61 | 2.31 | A_52_P221588 | *H2al1n* |
| 0.0062361 | 79.69 | 34.54 | 2.31 | A_52_P113950 | *NAP030542-1* |
| 0.0142404 | 54.52 | 23.62 | 2.31 | A_51_P313581 | *Fabp2* |
| 0.008489 | 15.99 | 6.92 | 2.31 | A_51_P254425 | *Ahrr* |
| 0.0270262 | 8.26 | 3.57 | 2.31 | A_51_P183197 | *Cysrt1* |
| 0.0145742 | 202.09 | 88.05 | 2.3 | A_51_P439326 | *Arhgap9* |
| 0.023379 | 4544.31 | 1979.69 | 2.3 | A_51_P142153 | *Filip1l* |
| 0.0288675 | 21.2 | 9.22 | 2.3 | A_51_P486121 | *Aff3* |
| 0.0414047 | 7.66 | 3.33 | 2.3 | A_52_P645675 | *NAP108488-1* |
| 0.0251653 | 4.12 | 1.79 | 2.3 | A_52_P159365 | *Sall3* |
| 0.00612 | 45.02 | 19.55 | 2.3 | A_52_P763117 | *AK049729* |
| 0.0121613 | 2290.81 | 1002.53 | 2.29 | A_51_P267587 | *Gdap10* |
| 0.0417233 | 347.17 | 151.93 | 2.29 | A_51_P487918 | *Rinl* |
| 0.0093165 | 3349.97 | 1463.01 | 2.29 | A_51_P210956 | *Vcam1* |
| 0.025191 | 1884.69 | 822.89 | 2.29 | A_51_P424878 | *Fam213b* |
| 0.0491879 | 2866.92 | 1251.55 | 2.29 | A_52_P440284 | *Mxra7* |
| 0.0146086 | 723.93 | 315.99 | 2.29 | A_51_P260169 | *Gstm5* |
| 0.006555 | 40.69 | 17.74 | 2.29 | A_52_P1140098 | *Gm10914* |
| 0.0072963 | 154.19 | 67.22 | 2.29 | A_52_P594756 | *Asb4* |
| 0.0065151 | 6.01 | 2.62 | 2.29 | A_51_P136181 | *AK047573* |
| 0.0103966 | 2607.75 | 1136.77 | 2.29 | A_51_P440790 | *Dpp7* |
| 0.00471 | 37.6 | 16.51 | 2.28 | A_51_P446085 | *Map10* |
| 0.0336401 | 4.01 | 1.76 | 2.28 | A_51_P520699 | *Shisa8* |
| 0.0289173 | 40.15 | 17.62 | 2.28 | A_52_P380805 | *Pdpn* |
| 0.027711 | 249.29 | 109.18 | 2.28 | A_51_P307979 | *Etv1* |
| 0.0248716 | 182.44 | 79.9 | 2.28 | A_52_P631307 | *Ypel4* |
| 0.0297389 | 3.21 | 1.42 | 2.27 | A_52_P852771 | *A_52_P852771* |
| 0.0078684 | 7517.81 | 3318.6 | 2.27 | A_51_P202033 | *Wls* |
| 0.0072041 | 517.7 | 228.46 | 2.27 | A_51_P286946 | *Lhpp* |
| 0.0403 | 119.67 | 52.7 | 2.27 | A_51_P173678 | *Slc10a6* |
| 0.016875 | 36.7 | 16.16 | 2.27 | A_51_P179697 | *Fam57b* |
| 0.0077943 | 45.45 | 20.01 | 2.27 | A_51_P232748 | *Plxnb3* |
| 0.0093067 | 433.12 | 190.56 | 2.27 | A_51_P505868 | *Lhfp* |
| 0.0272935 | 112.86 | 49.65 | 2.27 | A_52_P343455 | *Tnfrsf13b* |
| 0.041952 | 285.46 | 125.53 | 2.27 | A_51_P200339 | *Cachd1* |
| 0.020744 | 385.01 | 170.4 | 2.26 | A_51_P144438 | *Znfx1* |
| 0.0068509 | 83.36 | 36.89 | 2.26 | A_51_P144813 | *Cdkn2a* |
| 0.0133379 | 24.41 | 10.8 | 2.26 | A_52_P665393 | *2610030P05Rik* |
| 0.0092975 | 7.54 | 3.33 | 2.26 | A_52_P22421 | *AK078692* |
| 0.0212203 | 1275.9 | 563.33 | 2.26 | A_52_P516091 | *Pla2g15* |
| 0.0379949 | 286.22 | 126.37 | 2.26 | A_52_P1083556 | *AK032841* |
| 0.0126513 | 25.69 | 11.44 | 2.25 | A_52_P221756 | *Spock2* |
| 0.0485812 | 105.18 | 46.79 | 2.25 | A_52_P451644 | *Ndn* |
| 0.0138619 | 343.56 | 152.82 | 2.25 | A_51_P231308 | *Mfng* |
| 0.0102309 | 2085.94 | 927.79 | 2.25 | A_52_P380895 | *Evi2a* |
| 0.0324825 | 7175.7 | 3187 | 2.25 | A_51_P163188 | *Rin2* |
| 0.0184225 | 10.27 | 4.59 | 2.24 | A_52_P1084809 | *Gm39334* |
| 0.0068678 | 2322.91 | 1038.16 | 2.24 | A_51_P157994 | *Dab2* |
| 0.0275383 | 1782.5 | 796.58 | 2.24 | A_51_P362638 | *Trf* |
| 0.0335362 | 27.15 | 12.13 | 2.24 | A_51_P462918 | *Ehhadh* |
| 0.0245229 | 18.63 | 8.32 | 2.24 | A_51_P340653 | *Slc16a4* |
| 0.0102263 | 44960.69 | 20074.17 | 2.24 | A_51_P197528 | *Ly6c2* |
| 0.0261552 | 82.19 | 36.69 | 2.24 | A_52_P949284 | *A_52_P949284* |
| 0.0140525 | 4.37 | 1.95 | 2.24 | A_52_P614320 | *Gm128* |
| 0.0106319 | 138.09 | 61.54 | 2.24 | A_51_P407515 | *6330407A03Rik* |
| 0.0182161 | 2.85 | 1.27 | 2.24 | A_52_P557395 | *Gm5084* |
| 0.0218633 | 42.19 | 18.95 | 2.23 | A_51_P184223 | *Pcdhb7* |
| 0.012104 | 4.81 | 2.16 | 2.23 | A_51_P435617 | *Best3* |
| 0.0095896 | 1366.2 | 613.26 | 2.23 | A_52_P472324 | *Slpi* |
| 0.0154299 | 3436.78 | 1541.02 | 2.23 | A_52_P12023 | *NAP033385-1* |
| 0.0141274 | 931.15 | 417.44 | 2.23 | A_51_P221998 | *Man1c1* |
| 0.0227585 | 139.06 | 62.24 | 2.23 | A_51_P257292 | *Pcdhb5* |
| 0.0184133 | 662.95 | 298.79 | 2.22 | A_52_P324767 | *Tmed8* |
| 0.0357378 | 230.08 | 103.63 | 2.22 | A_52_P460703 | *Rassf3* |
| 0.0171916 | 717.11 | 322.78 | 2.22 | A_52_P99848 | *Pik3cd* |
| 0.0467215 | 143.49 | 64.58 | 2.22 | A_52_P319361 | *Rab30* |
| 0.0147252 | 175777.67 | 79077.62 | 2.22 | A_51_P171999 | *Apoe* |
| 0.016285 | 16048.98 | 7215.46 | 2.22 | A_52_P113611 | *Scn5a* |
| 0.0447684 | 181.73 | 82.38 | 2.21 | A_51_P297671 | *Gk* |
| 0.0067094 | 83.17 | 37.68 | 2.21 | A_52_P708792 | *Gm5547* |
| 0.0087954 | 234.19 | 105.99 | 2.21 | A_52_P206762 | *Zfp422* |
| 0.0266582 | 442.89 | 200.3 | 2.21 | A_51_P387379 | *Tshz3* |
| 0.0054894 | 340.9 | 154.12 | 2.21 | A_52_P112676 | *Ccdc28b* |
| 0.0421451 | 101.62 | 45.92 | 2.21 | A_51_P385974 | *Rapsn* |
| 0.0086323 | 712.34 | 321.66 | 2.21 | A_51_P463187 | *Tbc1d2b* |
| 0.0254504 | 3927.2 | 1785.51 | 2.2 | A_51_P234113 | *Nod1* |
| 0.0314469 | 96.84 | 44.02 | 2.2 | A_52_P207654 | *TC1655445* |
| 0.0135018 | 55.84 | 25.37 | 2.2 | A_52_P136709 | *Pctp* |
| 0.0324172 | 1613.63 | 733.05 | 2.2 | A_51_P127367 | *Irf9* |
| 0.0216587 | 63.43 | 28.81 | 2.2 | A_51_P451335 | *Pla2r1* |
| 0.0349791 | 5.89 | 2.67 | 2.2 | A_51_P450169 | *Dnajb13* |
| 0.0154355 | 9517.86 | 4355.32 | 2.19 | A_51_P227222 | *Adamts2* |
| 0.033947 | 5.53 | 2.53 | 2.19 | A_51_P134913 | *Gpr15* |
| 0.0219111 | 188.25 | 86.12 | 2.19 | A_51_P125355 | *Maoa* |
| 0.0343443 | 154.66 | 70.72 | 2.19 | A_51_P262841 | *Stambpl1* |
| 0.0241195 | 377.5 | 172.44 | 2.19 | A_51_P337675 | *Cd53* |
| 0.0117433 | 101.98 | 46.55 | 2.19 | A_51_P244586 | *Zfp618* |
| 0.0430408 | 1192.36 | 543.9 | 2.19 | A_52_P342159 | *Nfatc4* |
| 0.0283245 | 25.76 | 11.75 | 2.19 | A_51_P250049 | *AK087715* |
| 0.0332389 | 4.1 | 1.87 | 2.19 | A_52_P513811 | *TC1705553* |
| 0.0058263 | 173.62 | 79.18 | 2.19 | A_52_P537907 | *Tsga10* |
| 0.0085417 | 4.67 | 2.15 | 2.18 | A_52_P44259 | *LF194582* |
| 0.0158834 | 13858.66 | 6368.43 | 2.18 | A_51_P453111 | *Hexb* |
| 0.0265097 | 4.92 | 2.26 | 2.18 | A_51_P271761 | *Gm568* |
| 0.019395 | 17024.15 | 7818.8 | 2.18 | A_51_P157042 | *Ctgf* |
| 0.0380487 | 282.15 | 129.47 | 2.18 | A_51_P482121 | *Edn2* |
| 0.0364391 | 16.74 | 7.68 | 2.18 | A_52_P1196325 | *AK051621* |
| 0.014985 | 4761.5 | 2183.26 | 2.18 | A_51_P470432 | *Pdlim4* |
| 0.0247583 | 17752.98 | 8134.86 | 2.18 | A_51_P403536 | *Ltbp4* |
| 0.0449123 | 178.42 | 81.72 | 2.18 | A_51_P504546 | *Aldh1l1* |
| 0.017403 | 80.19 | 36.72 | 2.18 | A_51_P260504 | *Arhgef4* |
| 0.0089268 | 256.2 | 118.31 | 2.17 | A_52_P193440 | *E130102H24Rik* |
| 0.0228684 | 563.32 | 260.08 | 2.17 | A_51_P236267 | *St8sia4* |
| 0.0318418 | 41.98 | 19.38 | 2.17 | A_51_P462870 | *Mkl2* |
| 0.0344834 | 2474.38 | 1141.7 | 2.17 | A_51_P408343 | *Ifi204* |
| 0.0366787 | 19.58 | 9.03 | 2.17 | A_52_P452599 | *Bco2* |
| 0.0483594 | 1259.73 | 580.83 | 2.17 | A_51_P444669 | *Tmem106a* |
| 0.0085124 | 678.34 | 312.68 | 2.17 | A_51_P185465 | *Slc29a3* |
| 0.0461921 | 5.6 | 2.58 | 2.17 | A_52_P715292 | *AK034384* |
| 0.0143967 | 275.25 | 126.58 | 2.17 | A_51_P105991 | *Tbx6* |
| 0.0251688 | 198.9 | 92.29 | 2.16 | A_51_P322265 | *Grap* |
| 0.0395996 | 94.18 | 43.65 | 2.16 | A_51_P515137 | *2610027H17Rik* |
| 0.0367114 | 163.56 | 75.77 | 2.16 | A_51_P502203 | *Asap3* |
| 0.0333111 | 170.67 | 79.05 | 2.16 | A_51_P148290 | *Azin2* |
| 0.0058284 | 50.6 | 23.43 | 2.16 | A_51_P327585 | *Gstm4* |
| 0.0182697 | 1414.22 | 654.22 | 2.16 | A_51_P404463 | *1500015O10Rik* |
| 0.0073978 | 37.55 | 17.5 | 2.15 | A_51_P506128 | *Zfp28* |
| 0.0261622 | 16.48 | 7.68 | 2.15 | A_52_P1116185 | *AK051921* |
| 0.0155115 | 104 | 48.42 | 2.15 | A_51_P314388 | *Cntln* |
| 0.0324307 | 1766.55 | 821.82 | 2.15 | A_52_P231075 | *Fcrls* |
| 0.0121057 | 214.31 | 99.53 | 2.15 | A_51_P167360 | *Ptpn7* |
| 0.0189379 | 9715.96 | 4548.94 | 2.14 | A_52_P585751 | *Gns* |
| 0.0077542 | 713.19 | 333.77 | 2.14 | A_51_P308275 | *P2rx4* |
| 0.0106225 | 18934.01 | 8848.25 | 2.14 | A_51_P470328 | *Selenop* |
| 0.0362903 | 554.38 | 258.77 | 2.14 | A_52_P497908 | *Lhx6* |
| 0.0175888 | 426.17 | 198.88 | 2.14 | A_51_P184886 | *Abcg2* |
| 0.0303084 | 9.23 | 4.34 | 2.13 | A_51_P361858 | *AK003099* |
| 0.0221673 | 122.47 | 57.57 | 2.13 | A_51_P415225 | *Zfp105* |
| 0.0284999 | 547.41 | 257.18 | 2.13 | A_51_P159711 | *Car7* |
| 0.0412102 | 31.29 | 14.69 | 2.13 | A_52_P639048 | *Adra1b* |
| 0.0113373 | 839.06 | 393.85 | 2.13 | A_51_P370600 | *Fli1* |
| 0.0067548 | 104.3 | 48.95 | 2.13 | A_52_P302433 | *Plau* |
| 0.0368495 | 125.05 | 58.63 | 2.13 | A_52_P44824 | *Gngt2* |
| 0.00632 | 5.74 | 2.71 | 2.12 | A_51_P514584 | *Tbata* |
| 0.0302976 | 20.04 | 9.46 | 2.12 | A_52_P351905 | *9530001P21Rik* |
| 0.0064958 | 17618.82 | 8312.14 | 2.12 | A_52_P93910 | *Nrp2* |
| 0.0136633 | 26.11 | 12.31 | 2.12 | A_52_P121502 | *Pllp* |
| 0.0252553 | 2854.23 | 1345.6 | 2.12 | A_52_P132165 | *Hsd17b11* |
| 0.0461923 | 13.58 | 6.4 | 2.12 | A_51_P122217 | *Ripor2* |
| 0.0410539 | 66.33 | 31.26 | 2.12 | A_52_P691933 | *AK087708* |
| 0.0133792 | 47.18 | 22.23 | 2.12 | A_51_P465292 | *Hnmt* |
| 0.0372936 | 1051.46 | 495.08 | 2.12 | A_51_P476767 | *Nnmt* |
| 0.0415365 | 656.09 | 311.65 | 2.11 | A_51_P134627 | *Asb13* |
| 0.0328654 | 33.66 | 15.97 | 2.11 | A_51_P511918 | *Pcdhb18* |
| 0.0078238 | 11.11 | 5.27 | 2.11 | A_51_P261560 | *Tex33* |
| 0.0410128 | 987.77 | 467.88 | 2.11 | A_52_P1012802 | *Ptprj* |
| 0.0238165 | 210.66 | 99.73 | 2.11 | A_51_P297993 | *A_51_P297993* |
| 0.0478232 | 17216.01 | 8142.83 | 2.11 | A_52_P470663 | *Rdh10* |
| 0.007114 | 13.24 | 6.3 | 2.1 | A_51_P161248 | *Scg3* |
| 0.0248057 | 12.73 | 6.05 | 2.1 | A_51_P126258 | *Pcdhga1* |
| 0.0175693 | 496.23 | 235.78 | 2.1 | A_51_P249989 | *Tifa* |
| 0.010209 | 20.69 | 9.92 | 2.09 | A_51_P490148 | *Nphp4* |
| 0.0430482 | 133.58 | 64.03 | 2.09 | A_51_P372112 | *Gpc6* |
| 0.0467146 | 764.61 | 366.09 | 2.09 | A_51_P365189 | *Il13ra1* |
| 0.0137877 | 7.25 | 3.47 | 2.09 | A_51_P403378 | *Cnr1* |
| 0.0420559 | 4638.41 | 2219.53 | 2.09 | A_51_P250058 | *Epas1* |
| 0.0202208 | 59.25 | 28.32 | 2.09 | A_51_P123546 | *Armc3* |
| 0.0257459 | 17.81 | 8.51 | 2.09 | A_52_P293120 | *Prrg3* |
| 0.0457282 | 5.17 | 2.47 | 2.09 | A_51_P509137 | *Hrk* |
| 0.0444286 | 996.97 | 476.13 | 2.09 | A_51_P185906 | *Abi3* |
| 0.01636 | 117.37 | 56.56 | 2.08 | A_51_P256884 | *Mtss1l* |
| 0.0179066 | 64.48 | 31.04 | 2.08 | A_51_P393563 | *A_51_P393563* |
| 0.0073559 | 1471.93 | 707.73 | 2.08 | A_51_P425824 | *C1qtnf12* |
| 0.037734 | 111.16 | 53.41 | 2.08 | A_51_P144134 | *Slc22a16* |
| 0.0329645 | 365.02 | 175.26 | 2.08 | A_51_P284730 | *Fam217b* |
| 0.0388403 | 81.9 | 39.32 | 2.08 | A_52_P570690 | *Atp1b2* |
| 0.0277019 | 54.81 | 26.31 | 2.08 | A_51_P376934 | *Bace2* |
| 0.0105761 | 130.1 | 62.4 | 2.08 | A_51_P395405 | *Klhl5* |
| 0.0143585 | 252.37 | 121.04 | 2.08 | A_52_P530291 | *Pim1* |
| 0.0135852 | 498.44 | 240.6 | 2.07 | A_51_P451758 | *Rab3il1* |
| 0.0073774 | 8442.5 | 4072.88 | 2.07 | A_51_P505493 | *Elovl5* |
| 0.0448094 | 2.94 | 1.43 | 2.06 | A_52_P972052 | *AK045847* |
| 0.0477791 | 6.01 | 2.92 | 2.06 | A_52_P875265 | *AK035370* |
| 0.0076068 | 140.06 | 68.04 | 2.06 | A_51_P267278 | *Slc15a2* |
| 0.0072307 | 12.98 | 6.3 | 2.06 | A_52_P1180839 | *AK087344* |
| 0.0090954 | 2568.87 | 1246.58 | 2.06 | A_52_P372901 | *Plscr2* |
| 0.0111887 | 1066.63 | 517.15 | 2.06 | A_51_P106322 | *Gpsm3* |
| 0.0150701 | 3807.35 | 1845.69 | 2.06 | A_51_P172231 | *Gsdmd* |
| 0.0299047 | 2463.96 | 1194.02 | 2.06 | A_51_P278994 | *Swap70* |
| 0.0231197 | 177.92 | 86.2 | 2.06 | A_52_P94521 | *2510009E07Rik* |
| 0.0114334 | 4566.11 | 2230.77 | 2.05 | A_51_P238722 | *Cd93* |
| 0.0230122 | 431.25 | 210.68 | 2.05 | A_51_P483215 | *Phxr4* |
| 0.0166403 | 212.71 | 103.91 | 2.05 | A_51_P362698 | *AK054351* |
| 0.0468368 | 7337.18 | 3584.15 | 2.05 | A_51_P445728 | *Pirb* |
| 0.0186091 | 8.07 | 3.94 | 2.05 | A_51_P367780 | *Adamtsl2* |
| 0.009024 | 3.55 | 1.73 | 2.05 | A_51_P410882 | *AK040362* |
| 0.0158394 | 783.51 | 381.62 | 2.05 | A_52_P117892 | *Pde7a* |
| 0.0459 | 92.28 | 44.93 | 2.05 | A_52_P334259 | *NAP103523-1* |
| 0.0080205 | 8.61 | 4.23 | 2.04 | A_52_P527268 | *D14Ertd426e* |
| 0.0175015 | 226.48 | 111.26 | 2.04 | A_51_P415809 | *Tusc1* |
| 0.0447477 | 14.13 | 6.94 | 2.04 | A_52_P436122 | *NAP056910-1* |
| 0.0087337 | 7159.97 | 3516.4 | 2.04 | A_51_P159503 | *Rnf213* |
| 0.012883 | 57.88 | 28.42 | 2.04 | A_51_P461123 | *Tlr5* |
| 0.0421373 | 580.62 | 284.95 | 2.04 | A_52_P356313 | *Stard9* |
| 0.0096419 | 235.56 | 115.54 | 2.04 | A_52_P118310 | *Chn1* |
| 0.0317603 | 59.71 | 29.2 | 2.04 | A_51_P243978 | *NAP026550-1* |
| 0.0164637 | 3.06 | 1.49 | 2.04 | A_52_P948295 | *AK089760* |
| 0.0291536 | 63.5 | 31.5 | 2.02 | A_52_P1019965 | *AK048091* |
| 0.0418732 | 5.95 | 2.95 | 2.02 | A_52_P274749 | *ENSMUST00000174341* |
| 0.0103414 | 75.2 | 37.28 | 2.02 | A_51_P372186 | *Zfp467* |
| 0.0434832 | 447.04 | 221.53 | 2.02 | A_52_P58594 | *Lipo3* |
| 0.0154151 | 328.12 | 162.58 | 2.02 | A_52_P424462 | *Ero1lb* |
| 0.0207799 | 9.93 | 4.92 | 2.02 | A_51_P242967 | *Piwil2* |
| 0.0131405 | 676.78 | 335.1 | 2.02 | A_51_P276235 | *Pnpla7* |
| 0.0124982 | 10.1 | 5 | 2.02 | A_52_P802796 | *AK047643* |
| 0.015566 | 113.31 | 55.99 | 2.02 | A_51_P444795 | *Pou2f2* |
| 0.0135192 | 824.65 | 407.33 | 2.02 | A_51_P232901 | *Cnp* |
| 0.039873 | 62.54 | 31.14 | 2.01 | A_51_P328622 | *Tlcd2* |
| 0.0087028 | 33.93 | 16.88 | 2.01 | A_52_P131778 | *Ldlrad3* |
| 0.0198129 | 692.95 | 344.57 | 2.01 | A_51_P260730 | *Syt11* |
| 0.039876 | 1736.34 | 863 | 2.01 | A_52_P662562 | *LF205063* |
| 0.0402113 | 7.88 | 3.91 | 2.01 | A_52_P1003937 | *AK037893* |
| 0.0133584 | 10.55 | 5.29 | 2 | A_51_P343717 | *ENSMUST00000121437* |
| 0.0360343 | 1120.1 | 561.18 | 2 | A_51_P504991 | *Slfn9* |
| 0.0124603 | 1170.34 | 586.11 | 2 | A_51_P376347 | *Hebp1* |
| 0.0084996 | 4916.38 | 2458.54 | 2 | A_51_P105124 | *P2ry6* |
| 0.04478 | 6.09 | 3.04 | 2 | A_52_P980283 | *AK080232* |
| 0.0277436 | 423.95 | 211.59 | 2 | A_52_P542645 | *Itga9* |
| 0.035674 | 52.66 | 26.27 | 2 | A_52_P78650 | *Cass4* |
| 0.029554 | 8.2 | 4.13 | 1.99 | A_51_P191791 | *Pcdhgb4* |
| 0.0227861 | 2.78 | 1.4 | 1.99 | A_52_P352958 | *NAP055006-1* |
| 0.0381962 | 136.98 | 68.92 | 1.99 | A_51_P405227 | *Nradd* |
| 0.041611 | 214.04 | 107.69 | 1.99 | A_51_P296528 | *Cers4* |
| 0.0266398 | 5.25 | 2.64 | 1.99 | A_52_P972094 | *AK051074* |
| 0.038659 | 27.62 | 13.86 | 1.99 | A_52_P263800 | *AK080088* |
| 0.0097956 | 4132.62 | 2072.78 | 1.99 | A_51_P156868 | *Gmfg* |
| 0.0334184 | 94.39 | 47.34 | 1.99 | A_52_P99082 | *NAP065150-1* |
| 0.0149992 | 33.07 | 16.73 | 1.98 | A_51_P320980 | *Sfxn4* |
| 0.0229528 | 848.55 | 429.24 | 1.98 | A_51_P377526 | *Sts* |
| 0.0175549 | 6018.4 | 3042.16 | 1.98 | A_51_P249127 | *Pkd1* |
| 0.0298712 | 7.54 | 3.81 | 1.98 | A_51_P451275 | *Zfp385b* |
| 0.0234702 | 8.67 | 4.38 | 1.98 | A_51_P348433 | *Rasal1* |
| 0.02163 | 32.67 | 16.5 | 1.98 | A_51_P514139 | *Prdm16* |
| 0.0169229 | 50.07 | 25.28 | 1.98 | A_51_P488211 | *Atp10d* |
| 0.0150142 | 1525.54 | 770.18 | 1.98 | A_51_P318192 | *Gem* |
| 0.0400098 | 49.75 | 25.11 | 1.98 | A_51_P409101 | *Cdhr4* |
| 0.0357935 | 24.32 | 12.27 | 1.98 | A_51_P332264 | *Dyx1c1* |
| 0.0114962 | 16630.59 | 8386.64 | 1.98 | A_51_P107326 | *Oxct1* |
| 0.0182696 | 470.78 | 237.4 | 1.98 | A_52_P504268 | *B3galnt1* |
| 0.04562 | 234.19 | 119.1 | 1.97 | A_51_P251129 | *Stard8* |
| 0.0311154 | 406.74 | 206.78 | 1.97 | A_52_P400999 | *Arhgap31* |
| 0.0172677 | 25.19 | 12.8 | 1.97 | A_52_P266320 | *Akap17b* |
| 0.008898 | 1257.12 | 636.66 | 1.97 | A_52_P215106 | *Pik3r6* |
| 0.0411348 | 4.66 | 2.36 | 1.97 | A_52_P939928 | *Gm8369* |
| 0.0095089 | 615.4 | 311.66 | 1.97 | A_51_P316379 | *Gpr146* |
| 0.0107624 | 1287.05 | 657.6 | 1.96 | A_52_P79923 | *Zfp691* |
| 0.015309 | 1706.72 | 871.96 | 1.96 | A_52_P118591 | *Tnfsfm13* |
| 0.0199295 | 720.79 | 367.72 | 1.96 | A_52_P33221 | *Apbb1ip* |
| 0.0357379 | 3.08 | 1.57 | 1.96 | A_51_P438039 | *Fam24a* |
| 0.0096305 | 12096.5 | 6163.86 | 1.96 | A_51_P218774 | *Rgs10* |
| 0.0177134 | 72.13 | 36.74 | 1.96 | A_52_P263990 | *Agk* |
| 0.0208029 | 66.97 | 34.39 | 1.95 | A_51_P343996 | *AK076545* |
| 0.0400993 | 6.74 | 3.46 | 1.95 | A_51_P284617 | *AK086271* |
| 0.0201567 | 37.87 | 19.43 | 1.95 | A_52_P467291 | *AK043151* |
| 0.0214399 | 4175.63 | 2141.05 | 1.95 | A_52_P190405 | *Ifnar2* |
| 0.0175427 | 2302.63 | 1179.71 | 1.95 | A_52_P350111 | *Cyth1* |
| 0.0310453 | 54.23 | 27.78 | 1.95 | A_51_P143431 | *Pde3b* |
| 0.0135986 | 181.38 | 92.91 | 1.95 | A_51_P263419 | *Zfp30* |
| 0.0142656 | 14.48 | 7.48 | 1.94 | A_52_P883463 | *AK045690* |
| 0.0348568 | 5.77 | 2.98 | 1.94 | A_51_P442118 | *Gm6583* |
| 0.0101156 | 137.36 | 70.89 | 1.94 | A_51_P416910 | *Slc36a4* |
| 0.0102718 | 23832.4 | 12296.96 | 1.94 | A_51_P494675 | *Cotl1* |
| 0.0243678 | 203.7 | 105 | 1.94 | A_52_P371135 | *C130050O18Rik* |
| 0.0147236 | 9.84 | 5.07 | 1.94 | A_51_P500082 | *Ifit1bl1* |
| 0.0164343 | 5419.93 | 2790.93 | 1.94 | A_52_P473866 | *Ppt1* |
| 0.026702 | 3.29 | 1.69 | 1.94 | A_51_P403477 | *Dio1* |
| 0.0298536 | 6.23 | 3.23 | 1.93 | A_52_P576499 | *NAP061425-1* |
| 0.0495443 | 571.76 | 296.02 | 1.93 | A_51_P464308 | *Gnb4* |
| 0.0169084 | 19.99 | 10.34 | 1.93 | A_52_P656389 | *NAP106668-1* |
| 0.0221185 | 19.13 | 9.99 | 1.92 | A_52_P383217 | *NAP030950-1* |
| 0.0187542 | 404.4 | 211.04 | 1.92 | A_51_P392350 | *Frmd4b* |
| 0.0484854 | 721.47 | 376.41 | 1.92 | A_52_P353417 | *Gm2a* |
| 0.0403216 | 3.01 | 1.57 | 1.92 | A_51_P256533 | *Nxf2* |
| 0.0219256 | 79.07 | 41.24 | 1.92 | A_51_P479645 | *Cdnf* |
| 0.020638 | 813.25 | 424.11 | 1.92 | A_52_P474719 | *Carmil1* |
| 0.0441356 | 3310.78 | 1725.49 | 1.92 | A_51_P204740 | *Cd34* |
| 0.040471 | 5219.69 | 2714.8 | 1.92 | A_52_P658122 | *Ets2* |
| 0.034071 | 75.7 | 39.37 | 1.92 | A_51_P239177 | *Apol7c* |
| 0.0113416 | 35.66 | 18.72 | 1.91 | A_51_P213666 | *Psma8* |
| 0.0225989 | 74.63 | 39.16 | 1.91 | A_52_P559557 | *BC026585* |
| 0.0466743 | 158.43 | 83.13 | 1.91 | A_52_P249672 | *4833417J20Rik* |
| 0.0213913 | 882.33 | 462.88 | 1.91 | A_51_P389864 | *B3gnt9* |
| 0.0153719 | 3656.1 | 1917.86 | 1.91 | A_51_P144090 | *Slc16a3* |
| 0.0177046 | 7.57 | 3.97 | 1.91 | A_51_P399277 | *AF103797* |
| 0.0339022 | 1768.17 | 927.09 | 1.91 | A_51_P336770 | *Aebp1* |
| 0.0124898 | 4976.63 | 2602.89 | 1.91 | A_52_P586141 | *Adcy7* |
| 0.0251837 | 14.99 | 7.84 | 1.91 | A_52_P579519 | *Tmem144* |
| 0.0200547 | 29.65 | 15.5 | 1.91 | A_51_P163173 | *Rbm12b1* |
| 0.036638 | 2727.6 | 1425.13 | 1.91 | A_52_P306065 | *Nuak1* |
| 0.0405585 | 9546.48 | 5034.25 | 1.9 | A_52_P161495 | *Bcl6* |
| 0.0285007 | 28.51 | 15.03 | 1.9 | A_51_P290542 | *Spo11* |
| 0.0224067 | 764.21 | 402.62 | 1.9 | A_52_P1107697 | *AK031731* |
| 0.0364773 | 249.31 | 131.31 | 1.9 | A_51_P140017 | *Cysltr1* |
| 0.0433955 | 1176.64 | 619.56 | 1.9 | A_51_P435348 | *Tmem159* |
| 0.0190482 | 469.88 | 247.33 | 1.9 | A_52_P105197 | *Galc* |
| 0.0169595 | 8.54 | 4.49 | 1.9 | A_52_P1059923 | *AK042985* |
| 0.0454908 | 633.48 | 332.93 | 1.9 | A_51_P159171 | *Irak2* |
| 0.0408497 | 56.82 | 29.84 | 1.9 | A_52_P1116521 | *Gm39290* |
| 0.0151464 | 10.79 | 5.72 | 1.89 | A_52_P456714 | *Dnah1* |
| 0.0201826 | 61.78 | 32.74 | 1.89 | A_52_P995993 | *AK050867* |
| 0.0145585 | 4853.66 | 2571.05 | 1.89 | A_52_P355084 | *Metrnl* |
| 0.0415749 | 3.55 | 1.88 | 1.89 | A_52_P1100061 | *AK038083* |
| 0.0151629 | 4028.41 | 2131.11 | 1.89 | A_52_P101279 | *Glul* |
| 0.0141382 | 526.03 | 278.2 | 1.89 | A_51_P206134 | *Akna* |
| 0.0298716 | 100.49 | 53.11 | 1.89 | A_51_P304561 | *AK013640* |
| 0.0115513 | 370.94 | 195.92 | 1.89 | A_52_P198019 | *Fuz* |
| 0.0411814 | 79.55 | 42.42 | 1.88 | A_52_P619388 | *Kcnq1ot1* |
| 0.0340027 | 764.75 | 407.19 | 1.88 | A_51_P174407 | *BC028528* |
| 0.025544 | 5162.35 | 2746.3 | 1.88 | A_51_P267370 | *Itm2c* |
| 0.047743 | 29.91 | 15.9 | 1.88 | A_51_P423666 | *Fbxo17* |
| 0.0273013 | 230.6 | 122.51 | 1.88 | A_51_P170178 | *B3gnt8* |
| 0.0193334 | 8.21 | 4.36 | 1.88 | A_52_P263763 | *Map7d3* |
| 0.0464614 | 24.26 | 12.88 | 1.88 | A_52_P646552 | *Olfr128* |
| 0.0136504 | 201.88 | 108.25 | 1.87 | A_51_P225186 | *Calcrl* |
| 0.0161547 | 242.21 | 129.83 | 1.87 | A_51_P497993 | *Gpr155* |
| 0.015272 | 3.49 | 1.87 | 1.87 | A_51_P295131 | *Pax7* |
| 0.0265338 | 18.82 | 10.08 | 1.87 | A_52_P795929 | *AK081614* |
| 0.0289105 | 2763.88 | 1480.15 | 1.87 | A_51_P144285 | *Rps6kc1* |
| 0.0135425 | 23.19 | 12.41 | 1.87 | A_52_P1058797 | *Gm26688* |
| 0.0425618 | 6.13 | 3.28 | 1.87 | A_52_P62617 | *Gm9895* |
| 0.0146326 | 974.01 | 520.95 | 1.87 | A_51_P448563 | *Myo7a* |
| 0.0372797 | 1616.14 | 864.01 | 1.87 | A_51_P149623 | *Gabrd* |
| 0.0277601 | 18.35 | 9.81 | 1.87 | A_52_P327146 | *AK031552* |
| 0.0225153 | 90.44 | 48.34 | 1.87 | A_51_P144014 | *Gdap5* |
| 0.0129789 | 3.48 | 1.86 | 1.87 | A_52_P584704 | *NAP121186-002* |
| 0.0346176 | 283.3 | 151.39 | 1.87 | A_51_P302503 | *Ppp1r15a* |
| 0.037471 | 846.9 | 452.06 | 1.87 | A_52_P653303 | *TC1640144* |
| 0.0188379 | 47.99 | 25.61 | 1.87 | A_51_P319141 | *Stard5* |
| 0.0168305 | 3420.39 | 1824.78 | 1.87 | A_51_P234728 | *Lpar6* |
| 0.0486687 | 77.02 | 41.09 | 1.87 | A_51_P133252 | *Slc27a3* |
| 0.0278063 | 3129.36 | 1669.29 | 1.87 | A_52_P63044 | *Lsp1* |
| 0.0225801 | 3.27 | 1.74 | 1.87 | A_52_P1115773 | *LOC102642641* |
| 0.0200255 | 471.82 | 253.88 | 1.86 | A_52_P275700 | *Nr2f1* |
| 0.0357668 | 944.59 | 508.19 | 1.86 | A_51_P417839 | *Hdac4* |
| 0.0121298 | 3.83 | 2.06 | 1.86 | A_52_P200286 | *Dnah17* |
| 0.0130108 | 4.11 | 2.21 | 1.86 | A_51_P232558 | *4933414I06Rik* |
| 0.0217636 | 8402.32 | 4515.94 | 1.86 | A_51_P338443 | *Angptl4* |
| 0.0154491 | 150.8 | 81.73 | 1.85 | A_51_P275679 | *Rassf5* |
| 0.0126149 | 4.45 | 2.41 | 1.85 | A_51_P419270 | *Dnajc5b* |
| 0.0476552 | 5.32 | 2.88 | 1.85 | A_52_P1140678 | *AK077575* |
| 0.0139942 | 2653.47 | 1435.26 | 1.85 | A_51_P356760 | *Mical1* |
| 0.0123494 | 204.83 | 110.7 | 1.85 | A_51_P386670 | *Dse* |
| 0.0349872 | 6.84 | 3.73 | 1.84 | A_52_P648759 | *A_52_P648759* |
| 0.019238 | 181.05 | 98.63 | 1.84 | A_52_P41674 | *TC1699558* |
| 0.0238853 | 184.13 | 100.27 | 1.84 | A_51_P284442 | *9530077C05Rik* |
| 0.0424914 | 123.47 | 67.17 | 1.84 | A_52_P371922 | *Dgkh* |
| 0.0144407 | 159.2 | 86.52 | 1.84 | A_51_P177667 | *Fyn* |
| 0.0430344 | 1403.69 | 761.76 | 1.84 | A_52_P36191 | *TC1707343* |
| 0.0405196 | 105.64 | 57.27 | 1.84 | A_52_P378211 | *Dusp22* |
| 0.0252001 | 1200.6 | 657.79 | 1.83 | A_51_P334199 | *Ephb3* |
| 0.0333643 | 1401.49 | 767.24 | 1.83 | A_51_P452779 | *Pygl* |
| 0.0175875 | 7851.17 | 4296.22 | 1.83 | A_52_P571707 | *Extl3* |
| 0.0265796 | 7.15 | 3.91 | 1.83 | A_52_P883537 | *AK044879* |
| 0.0251632 | 213.09 | 116.41 | 1.83 | A_51_P452659 | *Ssc5d* |
| 0.0182253 | 59.73 | 32.62 | 1.83 | A_52_P189358 | *Rnf150* |
| 0.0297151 | 6344.25 | 3459.14 | 1.83 | A_52_P635278 | *Ece1* |
| 0.0150847 | 1244.09 | 684.59 | 1.82 | A_52_P155778 | *Snap23* |
| 0.0434395 | 21.7 | 11.93 | 1.82 | A_52_P112110 | *Tmem82* |
| 0.0159745 | 316.94 | 174.24 | 1.82 | A_52_P1367 | *AK162987* |
| 0.0375888 | 21.32 | 11.72 | 1.82 | A_51_P107039 | *4930555G01Rik* |
| 0.0175307 | 18898.4 | 10388.17 | 1.82 | A_51_P100856 | *Fn1* |
| 0.0442915 | 1082.27 | 594.85 | 1.82 | A_52_P682745 | *Dock4* |
| 0.0135879 | 1627.95 | 893.91 | 1.82 | A_51_P438149 | *Mapre2* |
| 0.0447498 | 21.78 | 11.95 | 1.82 | A_51_P239166 | *Adam21* |
| 0.0339649 | 4.94 | 2.71 | 1.82 | A_52_P295676 | *Cacnb2* |
| 0.0170696 | 753.66 | 413.36 | 1.82 | A_52_P413947 | *Mthfr* |
| 0.0322283 | 103.72 | 56.85 | 1.82 | A_51_P327831 | *AK029200* |
| 0.0478593 | 3681.32 | 2033.95 | 1.81 | A_52_P1197913 | *Gadd45b* |
| 0.0359805 | 341.96 | 188.93 | 1.81 | A_52_P660945 | *Ctsf* |
| 0.0295784 | 689.9 | 381.15 | 1.81 | A_52_P487156 | *Zfp984* |
| 0.0170825 | 53.16 | 29.36 | 1.81 | A_52_P436447 | *Slc25a35* |
| 0.0321626 | 54.71 | 30.21 | 1.81 | A_51_P304417 | *Ttc41* |
| 0.0333527 | 114.82 | 63.38 | 1.81 | A_52_P214241 | *Gt(pU21)140Imeg* |
| 0.0207201 | 25.93 | 14.31 | 1.81 | A_51_P148093 | *Ptprm* |
| 0.0362224 | 4.88 | 2.72 | 1.8 | A_51_P240287 | *Car5a* |
| 0.0310026 | 4050.86 | 2254.45 | 1.8 | A_51_P256665 | *Pon3* |
| 0.0290536 | 21.91 | 12.18 | 1.8 | A_52_P196458 | *Dzip1* |
| 0.014382 | 4.23 | 2.35 | 1.8 | A_51_P122481 | *1700010L04Rik* |
| 0.0243497 | 18.65 | 10.35 | 1.8 | A_52_P811068 | *AK030462* |
| 0.0265903 | 960.98 | 533.19 | 1.8 | A_51_P423578 | *Slfn2* |
| 0.0273634 | 278.32 | 155.7 | 1.79 | A_52_P36261 | *TC1645771* |
| 0.0206097 | 8156.58 | 4562.23 | 1.79 | A_51_P246001 | *Cpe* |
| 0.0169323 | 241.36 | 134.91 | 1.79 | A_52_P424692 | *AK048914* |
| 0.0158789 | 14968.89 | 8363.4 | 1.79 | A_51_P256202 | *Ctsz* |
| 0.016618 | 4446.35 | 2483.64 | 1.79 | A_51_P510849 | *Tmem50b* |
| 0.0303763 | 647.86 | 361.78 | 1.79 | A_52_P105840 | *Fnbp1* |
| 0.0433503 | 2527.95 | 1409.3 | 1.79 | A_51_P416974 | *Pbx3* |
| 0.0328587 | 26.25 | 14.63 | 1.79 | A_52_P360076 | *E330013P08Rik* |
| 0.0280062 | 1258.52 | 701.17 | 1.79 | A_51_P439248 | *Mrc1* |
| 0.0251016 | 13.74 | 7.65 | 1.79 | A_52_P114875 | *Ebf4* |
| 0.0226311 | 10.62 | 5.97 | 1.78 | A_51_P299485 | *AK085216* |
| 0.0215861 | 5509.93 | 3094.74 | 1.78 | A_51_P406454 | *Il10rb* |
| 0.0291042 | 63.07 | 35.4 | 1.78 | A_52_P859798 | *AK045817* |
| 0.0158989 | 25683.09 | 14407.01 | 1.78 | A_51_P356493 | *Bicc1* |
| 0.0272209 | 169.63 | 96.1 | 1.77 | A_52_P496202 | *Pcsk1n* |
| 0.0447059 | 33.98 | 19.24 | 1.77 | A_51_P491667 | *Derl3* |
| 0.0288149 | 9.9 | 5.6 | 1.77 | A_51_P430766 | *Il10* |
| 0.0200633 | 4431.16 | 2503.92 | 1.77 | A_52_P158527 | *Bcl2l11* |
| 0.0340676 | 10.91 | 6.16 | 1.77 | A_52_P690675 | *AK087943* |
| 0.0399631 | 4320.77 | 2438.56 | 1.77 | A_51_P463428 | *Pik3ip1* |
| 0.0203134 | 506.6 | 285.77 | 1.77 | A_51_P268953 | *Tmem64* |
| 0.0301846 | 2360.47 | 1331.47 | 1.77 | A_51_P206585 | *Runx1* |
| 0.0355677 | 280.61 | 158.27 | 1.77 | A_51_P407999 | *1500011B03Rik* |
| 0.0445301 | 3827.49 | 2158.31 | 1.77 | A_51_P161890 | *Fcgrt* |
| 0.0323277 | 7.91 | 4.46 | 1.77 | A_51_P108923 | *AK035046* |
| 0.0215136 | 129.32 | 72.9 | 1.77 | A_52_P350012 | *Megf9* |
| 0.0180133 | 632.2 | 356.31 | 1.77 | A_51_P128491 | *Tcn2* |
| 0.0464626 | 22476.93 | 12802.61 | 1.76 | A_52_P51078 | *Ctsh* |
| 0.046119 | 13.73 | 7.82 | 1.76 | A_52_P441305 | *TC1652492* |
| 0.0318239 | 2.34 | 1.33 | 1.76 | A_51_P410804 | *Olfr482* |
| 0.0220068 | 3253.53 | 1846.47 | 1.76 | A_52_P546635 | *Zeb1* |
| 0.0311922 | 1627.21 | 923.41 | 1.76 | A_52_P401640 | *Bpgm* |
| 0.0377671 | 56.37 | 31.98 | 1.76 | A_51_P363338 | *Prkd1* |
| 0.0167867 | 592.26 | 335.98 | 1.76 | A_52_P461378 | *Tmbim1* |
| 0.0468817 | 5.65 | 3.24 | 1.75 | A_51_P248403 | *Naip6* |
| 0.0352191 | 12265.12 | 7027.31 | 1.75 | A_51_P267239 | *Litaf* |
| 0.0389612 | 153.54 | 87.96 | 1.75 | A_52_P5263 | *NAP115274-1* |
| 0.0186067 | 3278.75 | 1877.73 | 1.75 | A_51_P129199 | *Parp10* |
| 0.0245373 | 2651.79 | 1514.4 | 1.75 | A_51_P256323 | *Lpin2* |
| 0.0338078 | 327.44 | 186.98 | 1.75 | A_52_P74368 | *Slc43a2* |
| 0.020568 | 10.99 | 6.27 | 1.75 | A_51_P333929 | *Col25a1* |
| 0.034729 | 236.35 | 134.71 | 1.75 | A_52_P233441 | *Gata2* |
| 0.0334646 | 4.72 | 2.69 | 1.75 | A_51_P305230 | *Elavl3* |
| 0.0211911 | 13.8 | 7.95 | 1.74 | A_51_P136337 | *Galm* |
| 0.0332691 | 2425.44 | 1397.26 | 1.74 | A_52_P481423 | *Cttnbp2nl* |
| 0.0343149 | 5.4 | 3.11 | 1.74 | A_51_P393544 | *A_51_P393544* |
| 0.0413639 | 726.82 | 418.41 | 1.74 | A_51_P473154 | *Traf5* |
| 0.0208286 | 1806.37 | 1036.93 | 1.74 | A_52_P636894 | *Sppl2a* |
| 0.0281024 | 253.08 | 146.69 | 1.73 | A_51_P373696 | *Maml2* |
| 0.0362587 | 804.43 | 466.26 | 1.73 | A_52_P168567 | *Cebpa* |
| 0.0413153 | 15.68 | 9.08 | 1.73 | A_51_P181865 | *Tmem38b* |
| 0.0446127 | 5493.74 | 3177.62 | 1.73 | A_51_P428345 | *Mbnl1* |
| 0.0471642 | 474.32 | 274.17 | 1.73 | A_52_P788961 | *A_52_P788961* |
| 0.0308335 | 11.35 | 6.56 | 1.73 | A_52_P851490 | *AK037474* |
| 0.0184198 | 2491.55 | 1439.68 | 1.73 | A_52_P326808 | *Ypel3* |
| 0.0323456 | 26.87 | 15.52 | 1.73 | A_51_P484671 | *Adcy3* |
| 0.0230151 | 691.41 | 399.11 | 1.73 | A_51_P107686 | *Foxc1* |
| 0.0181944 | 6.95 | 4.01 | 1.73 | A_52_P298953 | *NAP028353-1* |
| 0.0191664 | 608.03 | 350.76 | 1.73 | A_51_P157537 | *Mapkapk3* |
| 0.0427611 | 64.72 | 37.31 | 1.73 | A_52_P565015 | *NAP103072-1* |
| 0.0443617 | 8.94 | 5.15 | 1.73 | A_52_P1115594 | *AK047731* |
| 0.0303544 | 7.42 | 4.33 | 1.72 | A_52_P996260 | *AK052024* |
| 0.0219541 | 703.4 | 409.21 | 1.72 | A_51_P286397 | *2610524H06Rik* |
| 0.0323996 | 710.18 | 413.14 | 1.72 | A_51_P246224 | *Tram2* |
| 0.0208166 | 7.18 | 4.17 | 1.72 | A_51_P485237 | *BC052537* |
| 0.0497742 | 4283.91 | 2487.88 | 1.72 | A_51_P386585 | *Gpsm1* |
| 0.0426293 | 3.41 | 1.98 | 1.72 | A_52_P477359 | *A_52_P477359* |
| 0.0318054 | 9087.89 | 5274.17 | 1.72 | A_51_P167971 | *Bace1* |
| 0.0211847 | 1789.56 | 1038.02 | 1.72 | A_52_P673391 | *Stoml1* |
| 0.0265131 | 1062.4 | 616.01 | 1.72 | A_51_P286865 | *Wdr33* |
| 0.0257353 | 35.07 | 20.55 | 1.71 | A_51_P294402 | *Scarf2* |
| 0.0212032 | 63285.62 | 37043.54 | 1.71 | A_51_P225224 | *Htra1* |
| 0.0297608 | 466.89 | 273.24 | 1.71 | A_51_P300806 | *Tlr4* |
| 0.0449329 | 26.34 | 15.4 | 1.71 | A_52_P347176 | *Nat8l* |
| 0.0246027 | 20.47 | 12.07 | 1.7 | A_52_P802856 | *A1300008O04Rik* |
| 0.0269253 | 12.18 | 7.18 | 1.7 | A_52_P108223 | *D430004P15Rik* |
| 0.0232866 | 4201.84 | 2476.93 | 1.7 | A_51_P414412 | *Trafd1* |
| 0.0404126 | 3.8 | 2.24 | 1.7 | A_51_P329243 | *Tmem56* |
| 0.0228699 | 830.46 | 488.89 | 1.7 | A_52_P665979 | *Gls* |
| 0.0402429 | 32224.25 | 18969.38 | 1.7 | A_52_P609200 | *Serinc3* |
| 0.0219378 | 264.6 | 155.56 | 1.7 | A_51_P472531 | *Arsk* |
| 0.0280615 | 2375.39 | 1395.12 | 1.7 | A_51_P319572 | *Rusc2* |
| 0.0475931 | 6.81 | 4.04 | 1.69 | A_52_P253757 | *Nlrp1a* |
| 0.0349193 | 5.56 | 3.29 | 1.69 | A_52_P1108272 | *A130074J08Rik* |
| 0.0453882 | 20.05 | 11.86 | 1.69 | A_52_P819979 | *AK078658* |
| 0.0365973 | 267.38 | 158.04 | 1.69 | A_52_P6621 | *NAP028872-1* |
| 0.0366142 | 194.49 | 114.92 | 1.69 | A_51_P427926 | *Smad6* |
| 0.0234153 | 8.7 | 5.14 | 1.69 | A_52_P632423 | *Palm2* |
| 0.0451355 | 938.27 | 553.89 | 1.69 | A_51_P280437 | *Slc12a6* |
| 0.0373703 | 2447.74 | 1461.26 | 1.68 | A_52_P619007 | *Mllt3* |
| 0.0386308 | 20.7 | 12.34 | 1.68 | A_52_P546175 | *Gm5589* |
| 0.031623 | 81.68 | 48.68 | 1.68 | A_52_P16249 | *Dppa2* |
| 0.0265457 | 1208.64 | 720.27 | 1.68 | A_51_P448236 | *Ctsk* |
| 0.0357938 | 1608.01 | 958.08 | 1.68 | A_51_P146443 | *Dennd1a* |
| 0.0355143 | 5.8 | 3.45 | 1.68 | A_51_P348922 | *Olfr1* |
| 0.0497952 | 3027.22 | 1800.14 | 1.68 | A_51_P332602 | *Surf1* |
| 0.0293429 | 104.67 | 62.24 | 1.68 | A_52_P341320 | *AK046811* |
| 0.0465016 | 1161.86 | 690.81 | 1.68 | A_52_P215876 | *Kdm7a* |
| 0.0369964 | 12793.62 | 7601.57 | 1.68 | A_51_P474518 | *Pcdhga9* |
| 0.0353929 | 959.25 | 569.65 | 1.68 | A_51_P300493 | *Lox* |
| 0.0429894 | 71.04 | 42.66 | 1.67 | A_51_P510764 | *Agmo* |
| 0.0361661 | 223.2 | 133.96 | 1.67 | A_51_P189405 | *Podxl2* |
| 0.0475018 | 171.24 | 102.58 | 1.67 | A_52_P515572 | *Slc18a2* |
| 0.0266717 | 27.08 | 16.22 | 1.67 | A_52_P915781 | *AK038901* |
| 0.0252754 | 1687.88 | 1010.66 | 1.67 | A_52_P229770 | *Akap13* |
| 0.0306984 | 112.56 | 67.34 | 1.67 | A_51_P179789 | *Cd300ld* |
| 0.0366638 | 345.44 | 206.23 | 1.67 | A_51_P454797 | *Ccdc82* |
| 0.0252775 | 5.29 | 3.19 | 1.66 | A_52_P214516 | *A_52_P214516* |
| 0.0472275 | 22.8 | 13.73 | 1.66 | A_52_P95150 | *NAP028620-1* |
| 0.0473318 | 1181.98 | 710.9 | 1.66 | A_52_P65524 | *Ppp1r12b* |
| 0.0290755 | 273.63 | 164.56 | 1.66 | A_52_P300786 | *Naip5* |
| 0.0305005 | 4215.56 | 2534.77 | 1.66 | A_52_P700056 | *Wfdc17* |
| 0.0389609 | 28736.44 | 17267.27 | 1.66 | A_52_P86693 | *Ifi27* |
| 0.0304375 | 39.39 | 23.93 | 1.65 | A_52_P622941 | *ENSMUST00000179116* |
| 0.0466371 | 1098.31 | 666.9 | 1.65 | A_52_P142531 | *Phc3* |
| 0.0473101 | 301.37 | 182.92 | 1.65 | A_52_P348560 | *NAP102495-1* |
| 0.0499929 | 340.27 | 205.91 | 1.65 | A_51_P389957 | *Rgs14* |
| 0.0278017 | 502.76 | 304.14 | 1.65 | A_52_P81533 | *AK089292* |
| 0.0310618 | 3836.12 | 2319.87 | 1.65 | A_51_P427674 | *Cpt1a* |
| 0.0424205 | 28.16 | 17.01 | 1.65 | A_52_P754929 | *AK042092* |
| 0.0355714 | 4.02 | 2.46 | 1.64 | A_51_P342916 | *B3galt1* |
| 0.0303172 | 1470.7 | 899.02 | 1.64 | A_51_P312497 | *Atp7a* |
| 0.0443292 | 1526.47 | 932.67 | 1.64 | A_52_P646643 | *Dzip3* |
| 0.0475917 | 9.07 | 5.53 | 1.64 | A_51_P468707 | *AK078992* |
| 0.0434282 | 502.57 | 306.41 | 1.64 | A_51_P370050 | *Nagpa* |
| 0.0470124 | 1323.2 | 806.56 | 1.64 | A_52_P276748 | *Daam1* |
| 0.0479448 | 6.99 | 4.26 | 1.64 | A_52_P326468 | *Asb16* |
| 0.035489 | 2.2 | 1.34 | 1.64 | A_52_P439263 | *Ugt8a* |
| 0.0323017 | 39.84 | 24.25 | 1.64 | A_51_P476492 | *Plcxd2* |
| 0.0371844 | 287.4 | 176.79 | 1.63 | A_52_P851862 | *AK080781* |
| 0.0496686 | 2178.44 | 1339.89 | 1.63 | A_51_P465409 | *Kmt2a* |
| 0.0307696 | 8.46 | 5.2 | 1.63 | A_51_P334809 | *AK040811* |
| 0.0341801 | 3621.88 | 2222.68 | 1.63 | A_51_P416689 | *Ext1* |
| 0.0433741 | 4.94 | 3.03 | 1.63 | A_52_P434255 | *Trpm1* |
| 0.0354639 | 6404.31 | 3927.84 | 1.63 | A_51_P487298 | *Vasn* |
| 0.0426503 | 43.76 | 26.81 | 1.63 | A_52_P329207 | *Wfdc18* |
| 0.0356545 | 2665.97 | 1631.83 | 1.63 | A_51_P284946 | *Rnd3* |
| 0.0363104 | 10.95 | 6.7 | 1.63 | A_52_P1116342 | *AK084033* |
| 0.0305404 | 1606.28 | 982.78 | 1.63 | A_51_P266861 | *Me1* |
| 0.0458193 | 991.6 | 612.35 | 1.62 | A_51_P223776 | *Nr1d1* |
| 0.0485747 | 1559.16 | 962.76 | 1.62 | A_51_P140721 | *Chst14* |
| 0.0470868 | 25.07 | 15.46 | 1.62 | A_52_P633489 | *C1qtnf4* |
| 0.0423891 | 2.4 | 1.48 | 1.62 | A_52_P459143 | *Celf6* |
| 0.044321 | 8895.29 | 5484.61 | 1.62 | A_51_P370874 | *Pkig* |
| 0.0309232 | 1410.81 | 869.27 | 1.62 | A_51_P426246 | *Anks3* |
| 0.0361871 | 15.17 | 9.45 | 1.61 | A_52_P312368 | *NAP029356-1* |
| 0.0306457 | 73.3 | 45.64 | 1.61 | A_52_P456561 | *Abcd1* |
| 0.031797 | 884.93 | 550.61 | 1.61 | A_52_P344389 | *Zer1* |
| 0.0299111 | 38.05 | 23.67 | 1.61 | A_52_P818642 | *5830469G19Rik* |
| 0.0306622 | 64.29 | 39.94 | 1.61 | A_51_P124485 | *AK087204* |
| 0.0312527 | 589.46 | 369.13 | 1.6 | A_52_P397204 | *Csk* |
| 0.0463122 | 3032.06 | 1898.46 | 1.6 | A_52_P601385 | *Cd99l2* |
| 0.0392103 | 4275.03 | 2675.82 | 1.6 | A_51_P384033 | *Psen2* |
| 0.0307211 | 7606.2 | 4759.12 | 1.6 | A_51_P427663 | *Cnn2* |
| 0.0341235 | 2.05 | 1.28 | 1.6 | A_51_P441585 | *Vmn1r54* |
| 0.0449907 | 1.81 | 1.13 | 1.6 | A_51_P143185 | *AK047920* |
| 0.0417191 | 8.06 | 5.03 | 1.6 | A_52_P224485 | *Pcdhb12* |
| 0.0416237 | 47.96 | 30.25 | 1.59 | A_52_P235861 | *Peg3* |
| 0.0398141 | 4425.59 | 2789.86 | 1.59 | A_52_P234729 | *Pkd2* |
| 0.0490205 | 1142 | 719.5 | 1.59 | A_51_P435968 | *Tnfaip8* |
| 0.0334619 | 990.86 | 623.6 | 1.59 | A_51_P289912 | *Rapgef2* |
| 0.0454399 | 340.17 | 213.71 | 1.59 | A_51_P387591 | *Nfkbiz* |
| 0.0338151 | 530.83 | 333.39 | 1.59 | A_52_P394561 | *Txnrd2* |
| 0.0331693 | 2.63 | 1.65 | 1.59 | A_52_P30220 | *Gm31679* |
| 0.0427128 | 7348.52 | 4610.18 | 1.59 | A_51_P377620 | *Ifi211* |
| 0.0461434 | 7.54 | 4.73 | 1.59 | A_52_P955716 | *AK053446* |
| 0.035019 | 1354.64 | 860.09 | 1.58 | A_51_P225948 | *Snx33* |
| 0.0484201 | 562.57 | 357.04 | 1.58 | A_52_P32683 | *Hcfc2* |
| 0.0394908 | 2526.28 | 1601.88 | 1.58 | A_52_P183752 | *Cpd* |
| 0.0360242 | 1034.27 | 655.8 | 1.58 | A_51_P248444 | *Poglut1* |
| 0.0334194 | 13.81 | 8.73 | 1.58 | A_52_P723436 | *D130062J10Rik* |
| 0.033012 | 294.39 | 185.95 | 1.58 | A_52_P630774 | *5930436O19Rik* |
| 0.0431863 | 82.37 | 52.02 | 1.58 | A_52_P1124299 | *AK084543* |
| 0.0345932 | 6328.62 | 4037.89 | 1.57 | A_51_P507942 | *Atp13a2* |
| 0.0344124 | 465.62 | 296.52 | 1.57 | A_52_P546660 | *1700029I15Rik* |
| 0.0416632 | 249.56 | 158.72 | 1.57 | A_52_P151278 | *Lrch4* |
| 0.0431119 | 893.09 | 573.36 | 1.56 | A_51_P432651 | *Il3ra* |
| 0.0404863 | 9525.49 | 6111.94 | 1.56 | A_51_P259378 | *Rnf114* |
| 0.0411566 | 9468.73 | 6070.54 | 1.56 | A_51_P408410 | *Ctsa* |
| 0.0410614 | 2431.66 | 1555.81 | 1.56 | A_51_P340601 | *Btg3* |
| 0.0400498 | 2832.58 | 1828.38 | 1.55 | A_51_P494686 | *Sumf1* |
| 0.0465262 | 625.22 | 403.07 | 1.55 | A_52_P674386 | *Tmcc1* |
| 0.0398698 | 5458.83 | 3517.61 | 1.55 | A_51_P232371 | *Stab1* |
| 0.0428708 | 220.56 | 143.68 | 1.54 | A_51_P371241 | *Usf3* |
| 0.0441921 | 1322.62 | 861.1 | 1.54 | A_52_P20977 | *Ldlrad4* |
| 0.0490985 | 87.06 | 56.56 | 1.54 | A_52_P674608 | *NAP027879-1* |
| 0.0393923 | 695 | 450.79 | 1.54 | A_51_P166695 | *Inpp5b* |
| 0.0415446 | 4.65 | 3.04 | 1.53 | A_51_P443958 | *A_51_P443958* |
| 0.0408972 | 2510.92 | 1640.76 | 1.53 | A_51_P131800 | *Cyba* |
| 0.0440786 | 1921.67 | 1263.95 | 1.52 | A_51_P349367 | *Ccpg1* |
| 0.0499543 | 1230.36 | 809.1 | 1.52 | A_51_P375698 | *Fam13b* |
| 0.0467449 | 35.93 | 23.6 | 1.52 | A_51_P137991 | *Wnt5b* |
| 0.0419744 | 847.75 | 556.17 | 1.52 | A_51_P403760 | *Braf* |
| 0.0444128 | 3215.8 | 2125.38 | 1.51 | A_51_P254646 | *Jdp2* |
| 0.0472408 | 13980.37 | 9311.26 | 1.5 | A_51_P450573 | *Tgfbr2* |
| 0.0492043 | 22.17 | 14.75 | 1.5 | A_52_P836776 | *A_52_P836776* |
| 0.0467558 | 8719.75 | 13108.89 | 0.67 | A_51_P201532 | *Nup85* |
| 0.0495435 | 942.15 | 1413.11 | 0.67 | A_51_P131942 | *Dph5* |
| 0.0469623 | 814.96 | 1222.07 | 0.67 | A_51_P440892 | *Dph2* |
| 0.0496793 | 498.17 | 740.79 | 0.67 | A_51_P390078 | *Styx* |
| 0.048599 | 510.64 | 779.07 | 0.66 | A_52_P491849 | *Trp53* |
| 0.0459422 | 144.28 | 219.96 | 0.66 | A_52_P9347 | *Ddx23* |
| 0.0486354 | 4993.99 | 7596.57 | 0.66 | A_52_P498743 | *ENSMUST00000119956* |
| 0.0457556 | 319.53 | 485.94 | 0.66 | A_51_P108935 | *Iars2* |
| 0.0499812 | 45.79 | 69.46 | 0.66 | A_51_P418662 | *Snora3* |
| 0.0472008 | 35.07 | 53.13 | 0.66 | A_51_P493682 | *Zfp518a* |
| 0.0473413 | 10.64 | 16.09 | 0.66 | A_51_P360655 | *Slc22a6* |
| 0.0480929 | 16.59 | 25.07 | 0.66 | A_52_P422885 | *TC1669390* |
| 0.0495887 | 125.42 | 194.28 | 0.65 | A_51_P428529 | *Oas1c* |
| 0.0466775 | 5.73 | 8.86 | 0.65 | A_52_P1179609 | *Kcnh3* |
| 0.0393699 | 734.15 | 1134.36 | 0.65 | A_52_P430247 | *Nacc1* |
| 0.0397539 | 5.22 | 8.06 | 0.65 | A_52_P586062 | *ENSMUST00000206638* |
| 0.0454009 | 301.62 | 465.43 | 0.65 | A_52_P542204 | *Mtfmt* |
| 0.0444361 | 181.39 | 279.4 | 0.65 | A_52_P398211 | *ENSMUST00000191574* |
| 0.0499582 | 1006.85 | 1543.66 | 0.65 | A_52_P570288 | *Syncrip* |
| 0.047364 | 46590.22 | 71367.38 | 0.65 | A_51_P136097 | *Hist1h4d* |
| 0.0465634 | 143.48 | 219.6 | 0.65 | A_52_P296311 | *ENSMUST00000119442* |
| 0.0429137 | 23.83 | 36.45 | 0.65 | A_52_P624107 | *Gm5039* |
| 0.0417838 | 94.47 | 144.28 | 0.65 | A_52_P119128 | *Gm5065* |
| 0.0487599 | 545.34 | 858.67 | 0.64 | A_51_P253204 | *Katnb1* |
| 0.0402778 | 230.91 | 363.31 | 0.64 | A_51_P411355 | *Taf1c* |
| 0.0485536 | 10140.87 | 15946.81 | 0.64 | A_52_P658974 | *ENSMUST00000121222* |
| 0.0457448 | 5.65 | 8.88 | 0.64 | A_51_P183985 | *1500009C09Rik* |
| 0.0435809 | 153.79 | 241.48 | 0.64 | A_52_P206925 | *Parp16* |
| 0.0430995 | 210.26 | 330.08 | 0.64 | A_51_P297579 | *Gch1* |
| 0.0488108 | 7.9 | 12.4 | 0.64 | A_51_P157506 | *AK051759* |
| 0.0452358 | 69.36 | 108.82 | 0.64 | A_52_P426062 | *Gm40864* |
| 0.0445937 | 1161.73 | 1822.22 | 0.64 | A_52_P206613 | *Adss* |
| 0.0369758 | 1384.96 | 2169.84 | 0.64 | A_51_P162624 | *Sac3d1* |
| 0.0362067 | 63.73 | 99.74 | 0.64 | A_51_P501236 | *4930503H13Rik* |
| 0.0466492 | 8780.24 | 13734.82 | 0.64 | A_52_P602787 | *Hmgb1-rs17* |
| 0.0450542 | 59.58 | 93.2 | 0.64 | A_52_P422185 | *Gm15411* |
| 0.036913 | 19.96 | 31.21 | 0.64 | A_52_P358913 | *D830024N08Rik* |
| 0.0358192 | 991 | 1549.08 | 0.64 | A_51_P155073 | *Nudt14* |
| 0.0477705 | 5073.9 | 7931.22 | 0.64 | A_51_P447714 | *Rbmxl1* |
| 0.0453949 | 423.47 | 660.25 | 0.64 | A_51_P418339 | *Nudcd2* |
| 0.0499379 | 2087.73 | 3253.73 | 0.64 | A_51_P222453 | *Tmem254a* |
| 0.0380998 | 363.57 | 565.91 | 0.64 | A_51_P270891 | *1700056E22Rik* |
| 0.0380908 | 49.22 | 76.58 | 0.64 | A_52_P279068 | *Gpatch2* |
| 0.0438596 | 606.67 | 942.99 | 0.64 | A_52_P159885 | *Ddx49* |
| 0.0378435 | 10675.74 | 16593.41 | 0.64 | A_52_P528707 | *Mrpl57* |
| 0.0384077 | 224.98 | 349.65 | 0.64 | A_51_P393862 | *Spryd4* |
| 0.0438895 | 3121.29 | 4848.55 | 0.64 | A_51_P169576 | *ENSMUST00000185927* |
| 0.0443742 | 318.59 | 494.31 | 0.64 | A_52_P232314 | *NAP102462-1* |
| 0.0405416 | 288.33 | 447.1 | 0.64 | A_51_P282883 | *Rab4a* |
| 0.047978 | 578.64 | 897.19 | 0.64 | A_52_P201232 | *Xpo5* |
| 0.0424616 | 5413.79 | 8658.55 | 0.63 | A_51_P406077 | *Fmc1* |
| 0.0323881 | 48.36 | 77.27 | 0.63 | A_52_P571607 | *Myo1a* |
| 0.0443531 | 3.37 | 5.38 | 0.63 | A_52_P673122 | *NAP057972-1* |
| 0.0321306 | 8207.52 | 13074.8 | 0.63 | A_51_P322420 | *Srp9* |
| 0.0410596 | 2648.18 | 4218.61 | 0.63 | A_51_P392244 | *Tbcd* |
| 0.039267 | 2059.02 | 3278.66 | 0.63 | A_51_P195215 | *Fam149a* |
| 0.0321994 | 157.09 | 249.97 | 0.63 | A_52_P657817 | *Mapk8* |
| 0.0473862 | 881.48 | 1402.51 | 0.63 | A_51_P274163 | *Clybl* |
| 0.0392833 | 68.35 | 108.58 | 0.63 | A_52_P391000 | *Pde8a* |
| 0.0484214 | 499.37 | 792.68 | 0.63 | A_52_P386302 | *Borcs7* |
| 0.0349637 | 504.29 | 800.12 | 0.63 | A_51_P317443 | *Cd3eap* |
| 0.0359464 | 605.55 | 960.24 | 0.63 | A_52_P5549 | *Fam133b* |
| 0.0318523 | 2180.63 | 3456.04 | 0.63 | A_51_P172752 | *Gpatch4* |
| 0.0358923 | 38.35 | 60.76 | 0.63 | A_52_P957260 | *BB199080* |
| 0.0361035 | 85.97 | 135.73 | 0.63 | A_52_P323621 | *ENSMUST00000199146* |
| 0.0382014 | 1263.64 | 1994.76 | 0.63 | A_52_P518715 | *Nsf* |
| 0.0356449 | 15.55 | 25.28 | 0.62 | A_52_P1075882 | *LOC108167915* |
| 0.0370401 | 156 | 253.57 | 0.62 | A_51_P428742 | *Pus7l* |
| 0.0288412 | 6299.08 | 10236.19 | 0.62 | A_51_P227004 | *Cks1b* |
| 0.0360535 | 973.95 | 1582.48 | 0.62 | A_51_P193011 | *Klc1* |
| 0.0293239 | 92.77 | 150.73 | 0.62 | A_51_P161131 | *5730507C01Rik* |
| 0.0324259 | 269.47 | 437.72 | 0.62 | A_51_P186552 | *Hid1* |
| 0.0479599 | 28.96 | 46.9 | 0.62 | A_52_P477804 | *Rfx3* |
| 0.0385615 | 248.71 | 402.68 | 0.62 | A_52_P73559 | *ENSMUST00000159853* |
| 0.047011 | 61078.05 | 98868.65 | 0.62 | A_51_P405773 | *Hist1h2aa* |
| 0.0337854 | 627.91 | 1015.78 | 0.62 | A_51_P291139 | *Upf3b* |
| 0.0321831 | 1635.67 | 2644.42 | 0.62 | A_52_P252737 | *Rangap1* |
| 0.0352783 | 545.19 | 880.77 | 0.62 | A_52_P187855 | *Trim37* |
| 0.0440293 | 5.33 | 8.59 | 0.62 | A_52_P66809 | *TC1654250* |
| 0.0499649 | 1.21 | 1.95 | 0.62 | A_51_P449334 | *AK007945* |
| 0.0299843 | 152.21 | 244.8 | 0.62 | A_52_P495372 | *Twnk* |
| 0.0305766 | 403.16 | 648.37 | 0.62 | A_52_P657123 | *NAP101526-1* |
| 0.0333748 | 56.7 | 91.18 | 0.62 | A_52_P284814 | *Nup107* |
| 0.0408639 | 6.66 | 10.69 | 0.62 | A_51_P412895 | *Olfr1101* |
| 0.0456738 | 426.67 | 684.67 | 0.62 | A_51_P200622 | *Bloc1s2* |
| 0.0414675 | 1127.12 | 1805.72 | 0.62 | A_52_P682990 | *Timm50* |
| 0.0395712 | 1.48 | 2.45 | 0.61 | A_52_P1124356 | *AK086255* |
| 0.0320826 | 1477.77 | 2441.56 | 0.61 | A_51_P193980 | *ENSMUST00000119920* |
| 0.036208 | 4072.12 | 6725.41 | 0.61 | A_52_P649683 | *Srsf7* |
| 0.0382878 | 698.03 | 1152.19 | 0.61 | A_52_P5420 | *Mrps23* |
| 0.027656 | 569.45 | 939.85 | 0.61 | A_52_P1149594 | *A_52_P1149594* |
| 0.0246059 | 1474.69 | 2433.78 | 0.61 | A_51_P389636 | *Kcnn4* |
| 0.0424821 | 3.28 | 5.41 | 0.61 | A_52_P374754 | *Olfr1305* |
| 0.048684 | 260.47 | 428.92 | 0.61 | A_52_P26892 | *C1galt1* |
| 0.0278054 | 208.83 | 343.87 | 0.61 | A_52_P49406 | *Pde12* |
| 0.0336132 | 166.3 | 273.53 | 0.61 | A_52_P496497 | *Abhd6* |
| 0.0344706 | 40.25 | 66.11 | 0.61 | A_52_P196956 | *ENSMUST00000210047* |
| 0.0370894 | 7254.42 | 11900.6 | 0.61 | A_52_P473344 | *Srebf2* |
| 0.0336293 | 1734.81 | 2844.34 | 0.61 | A_51_P504490 | *Rbm34* |
| 0.0493432 | 119.77 | 196.25 | 0.61 | A_51_P415247 | *ENSMUST00000046721* |
| 0.0274424 | 4.22 | 6.91 | 0.61 | A_52_P254088 | *Ifnl3* |
| 0.0436341 | 2563.78 | 4196.6 | 0.61 | A_51_P314019 | *Banf1* |
| 0.0447724 | 1724.55 | 2821.94 | 0.61 | A_52_P534620 | *Oxnad1* |
| 0.0480843 | 2223.3 | 3634.35 | 0.61 | A_51_P165683 | *Hirip3* |
| 0.031631 | 10392.9 | 16988.12 | 0.61 | A_52_P481770 | *ENSMUST00000196164* |
| 0.0328986 | 11844.01 | 19350.82 | 0.61 | A_52_P320711 | *Dynlt1b* |
| 0.038958 | 308.96 | 504.71 | 0.61 | A_52_P344116 | *Rnf6* |
| 0.0349231 | 78.08 | 127.5 | 0.61 | A_52_P89717 | *ENSMUST00000076359* |
| 0.0485386 | 2380.22 | 3886.44 | 0.61 | A_51_P166155 | *Gmnn* |
| 0.030807 | 22.27 | 36.33 | 0.61 | A_52_P266643 | *Tigar* |
| 0.0283776 | 4958.95 | 8089.23 | 0.61 | A_52_P552832 | *Ndufa4* |
| 0.0322625 | 5.31 | 8.65 | 0.61 | A_51_P120823 | *Olfr868* |
| 0.0404453 | 6.22 | 10.13 | 0.61 | A_51_P368620 | *Adam3* |
| 0.0378901 | 381.11 | 620.04 | 0.61 | A_52_P147816 | *Morc4* |
| 0.0332342 | 17.58 | 28.6 | 0.61 | A_52_P195358 | *ENSMUST00000218244* |
| 0.0461977 | 79558.63 | 129416.39 | 0.61 | A_52_P165633 | *Hist1h2af* |
| 0.03235 | 1553.78 | 2611.23 | 0.6 | A_52_P265877 | *Aldh9a1* |
| 0.0286561 | 989.94 | 1661.04 | 0.6 | A_51_P257743 | *Dnajc22* |
| 0.0354624 | 120.93 | 202.87 | 0.6 | A_52_P337821 | *Gm8300* |
| 0.0353928 | 296.41 | 497.15 | 0.6 | A_51_P248067 | *Ezh2* |
| 0.0450558 | 104.36 | 174.87 | 0.6 | A_51_P379409 | *Cuedc1* |
| 0.0366787 | 78201.34 | 130960.62 | 0.6 | A_52_P130490 | *Hist1h2ao* |
| 0.0457131 | 13.33 | 22.32 | 0.6 | A_52_P291562 | *AK015959* |
| 0.0453064 | 257.24 | 429.8 | 0.6 | A_52_P238846 | *Bpnt1* |
| 0.0327145 | 2179.86 | 3639.13 | 0.6 | A_52_P333178 | *Pold1* |
| 0.0239996 | 4.38 | 7.3 | 0.6 | A_52_P98000 | *TC1650164* |
| 0.0258308 | 2529.21 | 4208.61 | 0.6 | A_52_P268549 | *Rhno1* |
| 0.0375619 | 720.03 | 1197.43 | 0.6 | A_51_P267080 | *Tymp* |
| 0.0280277 | 3.69 | 6.13 | 0.6 | A_51_P511521 | *Morc2b* |
| 0.0454232 | 303.26 | 502.94 | 0.6 | A_51_P327322 | *Cenpk* |
| 0.0362736 | 1329.22 | 2204.2 | 0.6 | A_52_P503208 | *ENSMUST00000119126* |
| 0.0277392 | 404.81 | 670.94 | 0.6 | A_52_P407751 | *Pom121* |
| 0.0293193 | 2.64 | 4.37 | 0.6 | A_51_P392738 | *Spata18* |
| 0.0389958 | 5345.3 | 8839.88 | 0.6 | A_52_P30877 | *Gm5176* |
| 0.0267687 | 1.76 | 2.91 | 0.6 | A_52_P490071 | *Olfr810* |
| 0.0212079 | 144.83 | 247.53 | 0.59 | A_51_P163261 | *ENSMUST00000121255* |
| 0.0201854 | 726.1 | 1240.97 | 0.59 | A_52_P380649 | *Thap12* |
| 0.0197707 | 429.16 | 733.2 | 0.59 | A_52_P590740 | *Tle3* |
| 0.0330855 | 2991.95 | 5101.75 | 0.59 | A_52_P507310 | *Mrps24* |
| 0.0329377 | 433.11 | 738.5 | 0.59 | A_52_P469512 | *Psmd9* |
| 0.0328249 | 46.18 | 78.73 | 0.59 | A_52_P493806 | *2310034G01Rik* |
| 0.0273339 | 625.16 | 1064.75 | 0.59 | A_51_P118763 | *Ahctf1* |
| 0.0411962 | 22.64 | 38.53 | 0.59 | A_51_P385653 | *Fam122b* |
| 0.027334 | 121.91 | 207.35 | 0.59 | A_51_P359137 | *Doc2g* |
| 0.0285359 | 162.56 | 276.43 | 0.59 | A_51_P193093 | *Pemt* |
| 0.0288779 | 13210.97 | 22447.38 | 0.59 | A_51_P495641 | *Stmn1* |
| 0.0231897 | 112.85 | 191.73 | 0.59 | A_51_P442838 | *Mettl18* |
| 0.0306848 | 2103.55 | 3572.95 | 0.59 | A_52_P404005 | *ENSMUST00000093326* |
| 0.028137 | 162.63 | 276.2 | 0.59 | A_51_P123345 | *Arf2* |
| 0.0227966 | 1341.64 | 2277.55 | 0.59 | A_51_P460958 | *Cep89* |
| 0.0305378 | 2238.21 | 3797.63 | 0.59 | A_51_P281255 | *Nasp* |
| 0.0295684 | 3.56 | 6.04 | 0.59 | A_52_P223626 | *Olig2* |
| 0.0380353 | 312.42 | 529.89 | 0.59 | A_51_P212012 | *8430419K02Rik* |
| 0.0334865 | 56.89 | 96.39 | 0.59 | A_52_P163541 | *NAP072161-1* |
| 0.0345805 | 3814.12 | 6461.2 | 0.59 | A_51_P252784 | *Fam216a* |
| 0.0237954 | 675.78 | 1144.76 | 0.59 | A_51_P420655 | *Reep4* |
| 0.0386167 | 151.95 | 257.22 | 0.59 | A_51_P265999 | *Gemin8* |
| 0.038444 | 68.58 | 116.08 | 0.59 | A_52_P365948 | *5033406O09Rik* |
| 0.0486126 | 78.75 | 132.92 | 0.59 | A_52_P674886 | *CF750939* |
| 0.0396562 | 1740.53 | 2936.68 | 0.59 | A_51_P296416 | *Orc6* |
| 0.0303279 | 229.1 | 386.47 | 0.59 | A_52_P464629 | *Nme7* |
| 0.0291386 | 20129.47 | 33941.93 | 0.59 | A_51_P426975 | *Hist2h4* |
| 0.026859 | 2438.07 | 4105.28 | 0.59 | A_52_P292528 | *Sfpq* |
| 0.0349921 | 2342.46 | 3943.23 | 0.59 | A_52_P624757 | *NAP000004-017* |
| 0.0315492 | 1097.32 | 1846.06 | 0.59 | A_51_P210928 | *Tsta3* |
| 0.0292778 | 201.37 | 338.66 | 0.59 | A_52_P551318 | *NAP046244-1* |
| 0.0219763 | 25.03 | 42.08 | 0.59 | A_52_P202991 | *LF197252* |
| 0.0244067 | 1.88 | 3.16 | 0.59 | A_52_P227833 | *Slc6a11* |
| 0.0413675 | 3612.5 | 6276.74 | 0.58 | A_52_P252007 | *ENSMUST00000210437* |
| 0.0392098 | 644.19 | 1119.19 | 0.58 | A_51_P425696 | *Nt5c2* |
| 0.0363895 | 12.55 | 21.77 | 0.58 | A_52_P891345 | *AK053488* |
| 0.018808 | 1.8 | 3.12 | 0.58 | A_51_P272681 | *Gm26560* |
| 0.0390788 | 2146.96 | 3718.21 | 0.58 | A_51_P288162 | *Ddx39* |
| 0.0235262 | 3.99 | 6.91 | 0.58 | A_52_P724703 | *A_52_P724703* |
| 0.0499663 | 4618.64 | 7991.42 | 0.58 | A_51_P464822 | *Hist1h1e* |
| 0.0416625 | 4.84 | 8.37 | 0.58 | A_52_P38825 | *ENSMUST00000118665* |
| 0.0306319 | 11.29 | 19.52 | 0.58 | A_51_P442592 | *9330153N18Rik* |
| 0.0415142 | 34.38 | 59.41 | 0.58 | A_52_P443676 | *9330119M13Rik* |
| 0.033105 | 3715.92 | 6404.13 | 0.58 | A_51_P249051 | *Pycr2* |
| 0.0497849 | 3.05 | 5.25 | 0.58 | A_52_P605001 | *LF197380* |
| 0.0339495 | 8.93 | 15.35 | 0.58 | A_52_P802748 | *AK047802* |
| 0.0448217 | 807.77 | 1387.56 | 0.58 | A_52_P612993 | *Tbrg4* |
| 0.0343947 | 52757.39 | 90606.75 | 0.58 | A_52_P257377 | *Hist2h2ac* |
| 0.0442635 | 199.53 | 342.3 | 0.58 | A_51_P132978 | *Idh1* |
| 0.0217299 | 13.81 | 23.69 | 0.58 | A_51_P134030 | *Oas1e* |
| 0.0389437 | 7.41 | 12.71 | 0.58 | A_52_P231762 | *ENSMUST00000212774* |
| 0.0472335 | 619.5 | 1062.47 | 0.58 | A_52_P708886 | *A_52_P708886* |
| 0.039191 | 3042.11 | 5216.63 | 0.58 | A_51_P170696 | *Haus1* |
| 0.0411491 | 36.64 | 62.81 | 0.58 | A_51_P346272 | *Arhgap33* |
| 0.0193019 | 679.32 | 1164.46 | 0.58 | A_52_P29646 | *Dis3* |
| 0.0205564 | 244.72 | 418.9 | 0.58 | A_52_P423859 | *Nvl* |
| 0.029155 | 134.44 | 230.01 | 0.58 | A_52_P58024 | *Gm21293* |
| 0.0210164 | 11.56 | 19.77 | 0.58 | A_52_P118264 | *Hyls1* |
| 0.0384726 | 7.06 | 12.07 | 0.58 | A_51_P288643 | *BC006028* |
| 0.0493844 | 469.4 | 802.5 | 0.58 | A_51_P406008 | *Hgh1* |
| 0.0229864 | 30.78 | 54.47 | 0.57 | A_52_P794346 | *NAP112302-1* |
| 0.0226829 | 13050.81 | 23092.28 | 0.57 | A_51_P412050 | *Hist1h4a* |
| 0.0209889 | 59.56 | 105.32 | 0.57 | A_52_P45724 | *Mterf1b* |
| 0.0344379 | 190.04 | 336.03 | 0.57 | A_52_P434039 | *Lsm2* |
| 0.0453945 | 34.85 | 61.51 | 0.57 | A_51_P213334 | *Hdac11* |
| 0.0254578 | 191.12 | 336.98 | 0.57 | A_52_P448805 | *1700096K18Rik* |
| 0.0495938 | 125.18 | 220.21 | 0.57 | A_52_P325797 | *TC1603713* |
| 0.0291162 | 469.59 | 825.58 | 0.57 | A_52_P704340 | *ENSMUST00000120803* |
| 0.0214085 | 925.03 | 1625.51 | 0.57 | A_51_P488626 | *Cnih4* |
| 0.0388986 | 1422.03 | 2498.57 | 0.57 | A_51_P285997 | *Dhx32* |
| 0.0488996 | 3.04 | 5.34 | 0.57 | A_51_P116725 | *Olfr1240* |
| 0.0227506 | 164.84 | 289.54 | 0.57 | A_52_P623775 | *Eri2* |
| 0.01705 | 6.76 | 11.86 | 0.57 | A_52_P506250 | *Olfr441* |
| 0.0336048 | 790.83 | 1387.46 | 0.57 | A_51_P128229 | *Hn1l* |
| 0.0180744 | 3892.73 | 6829.34 | 0.57 | A_52_P135392 | *Uck2* |
| 0.026316 | 560.49 | 982.77 | 0.57 | A_51_P302651 | *Topbp1* |
| 0.0303438 | 17.06 | 29.91 | 0.57 | A_52_P296026 | *AK080429* |
| 0.0189338 | 2409.36 | 4219.72 | 0.57 | A_51_P174681 | *Bzw2* |
| 0.0173741 | 183.75 | 321.2 | 0.57 | A_52_P401386 | *Jup* |
| 0.021783 | 9.28 | 16.22 | 0.57 | A_51_P197292 | *AK082620* |
| 0.0299324 | 1391.6 | 2432.22 | 0.57 | A_51_P436719 | *Eftud2* |
| 0.0328208 | 225.05 | 393.13 | 0.57 | A_52_P659258 | *Ppat* |
| 0.0204235 | 227.97 | 397.74 | 0.57 | A_51_P355943 | *Mvd* |
| 0.0334736 | 9.32 | 16.25 | 0.57 | A_52_P67270 | *4930515G01Rik* |
| 0.0380193 | 1451.48 | 2530.42 | 0.57 | A_51_P473533 | *Gm4737* |
| 0.030136 | 1661.81 | 2896 | 0.57 | A_52_P250664 | *NAP026936-1* |
| 0.0435377 | 129.98 | 226.42 | 0.57 | A_52_P371108 | *Cdc6* |
| 0.0356772 | 24.37 | 42.43 | 0.57 | A_52_P400637 | *Appbp2os* |
| 0.0397448 | 64.42 | 112.1 | 0.57 | A_51_P242591 | *Shq1* |
| 0.0356714 | 2.69 | 4.85 | 0.56 | A_52_P748350 | *TC1679528* |
| 0.0438832 | 51.6 | 92.94 | 0.56 | A_51_P474752 | *Ucn2* |
| 0.0231317 | 396.37 | 713.6 | 0.56 | A_52_P478339 | *Chac2* |
| 0.0356356 | 2.9 | 5.22 | 0.56 | A_52_P672636 | *Olfr974* |
| 0.0231102 | 1411.08 | 2538.31 | 0.56 | A_51_P308948 | *Tamm41* |
| 0.0230271 | 85.78 | 154.28 | 0.56 | A_51_P217706 | *Ribc1* |
| 0.0212093 | 12122.72 | 21795.72 | 0.56 | A_52_P571537 | *3110039M20Rik* |
| 0.0278596 | 15.8 | 28.36 | 0.56 | A_52_P648246 | *ENSMUST00000178167* |
| 0.0174598 | 103.36 | 185.52 | 0.56 | A_51_P326854 | *Ube2cbp* |
| 0.0377746 | 16.98 | 30.45 | 0.56 | A_51_P427603 | *Plppr2* |
| 0.0195948 | 20.84 | 37.37 | 0.56 | A_52_P227974 | *Nkain4* |
| 0.0212905 | 6675.11 | 11962.5 | 0.56 | A_51_P264064 | *Incenp* |
| 0.0181087 | 28.39 | 50.87 | 0.56 | A_52_P142327 | *1700031C06Rik* |
| 0.0194563 | 267.73 | 479.47 | 0.56 | A_51_P195808 | *Cenpl* |
| 0.0245304 | 1.91 | 3.42 | 0.56 | A_51_P108432 | *M31654* |
| 0.0254866 | 159.74 | 285.99 | 0.56 | A_52_P364871 | *Ube2d-ps* |
| 0.0241975 | 82.26 | 147.23 | 0.56 | A_52_P322962 | *NAP018810-001* |
| 0.0383089 | 884.63 | 1583.23 | 0.56 | A_51_P273979 | *Cenpa* |
| 0.0365007 | 12.37 | 22.13 | 0.56 | A_51_P364996 | *AK045584* |
| 0.0347116 | 2734.61 | 4891.03 | 0.56 | A_52_P72434 | *Khk* |
| 0.0287528 | 338.07 | 604.41 | 0.56 | A_51_P354706 | *Lefty1* |
| 0.0228915 | 532.01 | 950.37 | 0.56 | A_51_P329332 | *Slc19a2* |
| 0.0471189 | 144.6 | 257.9 | 0.56 | A_52_P139936 | *Phf21b* |
| 0.0200937 | 5.62 | 10.02 | 0.56 | A_52_P52521 | *NAP053249-1* |
| 0.046099 | 122.94 | 219.03 | 0.56 | A_52_P484118 | *Urb2* |
| 0.0383676 | 1485.55 | 2645.64 | 0.56 | A_51_P384673 | *Lrrc59* |
| 0.0384798 | 71.47 | 127.26 | 0.56 | A_51_P166288 | *Acy1* |
| 0.0221506 | 875.1 | 1556.98 | 0.56 | A_51_P158210 | *Mcm2* |
| 0.0376982 | 51.93 | 92.37 | 0.56 | A_52_P152412 | *Wdr89* |
| 0.0241665 | 2481.55 | 4412.69 | 0.56 | A_51_P482711 | *Dhcr24* |
| 0.0315429 | 1783.58 | 3167.84 | 0.56 | A_51_P359625 | *Gsr* |
| 0.0348292 | 610.19 | 1080.69 | 0.56 | A_52_P234189 | *Ints7* |
| 0.0436127 | 83.6 | 148.06 | 0.56 | A_51_P158598 | *Scrn2* |
| 0.0375629 | 32.32 | 59.29 | 0.55 | A_51_P495730 | *1700049L16Rik* |
| 0.0201906 | 33.31 | 60.85 | 0.55 | A_52_P371666 | *Cep68* |
| 0.0208417 | 1022.87 | 1868.52 | 0.55 | A_52_P329256 | *Fxn* |
| 0.015449 | 238.08 | 434.88 | 0.55 | A_51_P345747 | *E130309D02Rik* |
| 0.0226661 | 51.09 | 93.28 | 0.55 | A_52_P426863 | *Faap24* |
| 0.018565 | 714.58 | 1304.49 | 0.55 | A_51_P145232 | *ENSMUST00000117940* |
| 0.0332436 | 38.84 | 70.9 | 0.55 | A_52_P242631 | *Gm8363* |
| 0.0430634 | 50.75 | 92.43 | 0.55 | A_51_P326499 | *Chek1* |
| 0.0436966 | 1610.76 | 2932.73 | 0.55 | A_51_P312360 | *Pkmyt1* |
| 0.0338887 | 569.43 | 1036.2 | 0.55 | A_51_P357561 | *Fbxw9* |
| 0.0404374 | 113.36 | 206.2 | 0.55 | A_52_P22617 | *ENSMUST00000170405* |
| 0.0417385 | 556.81 | 1012.62 | 0.55 | A_51_P261379 | *Spats2* |
| 0.0157708 | 1513.59 | 2751.94 | 0.55 | A_51_P272147 | *Emc9* |
| 0.0183468 | 3459.78 | 6283.29 | 0.55 | A_51_P285446 | *Lig1* |
| 0.0282655 | 500.91 | 909.59 | 0.55 | A_51_P382618 | *Casp3* |
| 0.0494987 | 118.39 | 214.67 | 0.55 | A_51_P236588 | *9430038I01Rik* |
| 0.0225521 | 1287.22 | 2333.38 | 0.55 | A_51_P521125 | *Cfap36* |
| 0.0322804 | 1596.91 | 2894.07 | 0.55 | A_51_P473528 | *Ahcy* |
| 0.0163242 | 482.21 | 873.44 | 0.55 | A_52_P2670 | *Rmrp* |
| 0.0156562 | 559.53 | 1012.52 | 0.55 | A_51_P386080 | *Mecr* |
| 0.0203921 | 381.42 | 689.45 | 0.55 | A_51_P317512 | *5730508B09Rik* |
| 0.0165887 | 2499.19 | 4512.7 | 0.55 | A_51_P486190 | *Pabpc2* |
| 0.0226913 | 2317.17 | 4178.26 | 0.55 | A_51_P138548 | *Hmgb3* |
| 0.0260677 | 38.71 | 69.8 | 0.55 | A_52_P418807 | *9430081H08Rik* |
| 0.0497488 | 10 | 18.69 | 0.54 | A_51_P170725 | *Stra6l* |
| 0.0270865 | 1.67 | 3.12 | 0.54 | A_51_P257892 | *Adam25* |
| 0.0150017 | 149.18 | 278.25 | 0.54 | A_51_P151586 | *Haspin* |
| 0.0283725 | 68.84 | 128.06 | 0.54 | A_52_P148212 | *Mis18bp1* |
| 0.0295555 | 3.26 | 6.06 | 0.54 | A_52_P500202 | *Taar2* |
| 0.0231546 | 137.06 | 254.66 | 0.54 | A_51_P337655 | *Dsn1* |
| 0.0282619 | 8.13 | 15.09 | 0.54 | A_51_P230195 | *A730008H23Rik* |
| 0.0477109 | 54.18 | 100.53 | 0.54 | A_51_P256798 | *A_51_P256798* |
| 0.0145613 | 89.11 | 165.12 | 0.54 | A_52_P527874 | *Rrp12* |
| 0.0493672 | 1695.16 | 3137.83 | 0.54 | A_51_P505172 | *Tcf19* |
| 0.0160391 | 101.46 | 187.77 | 0.54 | A_51_P215438 | *Prodh* |
| 0.0149862 | 81.94 | 151.64 | 0.54 | A_52_P421005 | *Taf2* |
| 0.0394504 | 280.18 | 518.47 | 0.54 | A_51_P169087 | *Gls2* |
| 0.0394779 | 12.64 | 23.37 | 0.54 | A_52_P744437 | *D930028M14Rik* |
| 0.0158901 | 4307.91 | 7947.86 | 0.54 | A_52_P299703 | *Hist4h4* |
| 0.0182149 | 654.15 | 1205.78 | 0.54 | A_51_P201137 | *Lbr* |
| 0.0223281 | 17.98 | 33.07 | 0.54 | A_51_P403799 | *Sptbn2* |
| 0.035169 | 322.84 | 593.68 | 0.54 | A_52_P479539 | *Cit* |
| 0.0155692 | 49.31 | 90.61 | 0.54 | A_52_P620497 | *9630019E01Rik* |
| 0.0293521 | 196.67 | 360.98 | 0.54 | A_52_P31510 | *Pdx1* |
| 0.0301477 | 523.16 | 995.59 | 0.53 | A_51_P345792 | *Mfsd13a* |
| 0.0356581 | 510.29 | 970.08 | 0.53 | A_51_P345159 | *Ube2t* |
| 0.0109325 | 79.24 | 150.61 | 0.53 | A_52_P349467 | *Tmlhe* |
| 0.0191173 | 1.8 | 3.42 | 0.53 | A_51_P402175 | *Vsig8* |
| 0.0477719 | 6653.79 | 12632.4 | 0.53 | A_51_P262766 | *Ccnd1* |
| 0.023511 | 424.64 | 802.11 | 0.53 | A_52_P157880 | *ENSMUST00000122451* |
| 0.0192589 | 115.18 | 217.53 | 0.53 | A_52_P535084 | *NAP030857-1* |
| 0.0282177 | 830.16 | 1567.75 | 0.53 | A_51_P105709 | *Trip13* |
| 0.015082 | 2.73 | 5.15 | 0.53 | A_51_P105480 | *Nanos3* |
| 0.041155 | 327.09 | 616.48 | 0.53 | A_51_P480855 | *Rad18* |
| 0.0208363 | 36.75 | 69.24 | 0.53 | A_52_P215176 | *Opn3* |
| 0.0129856 | 580.03 | 1092.41 | 0.53 | A_51_P490388 | *Nudt8* |
| 0.044878 | 344.68 | 648.83 | 0.53 | A_52_P381303 | *Gins2* |
| 0.0219732 | 50.27 | 94.6 | 0.53 | A_52_P230609 | *0610039H22Rik* |
| 0.0301668 | 575.23 | 1081.59 | 0.53 | A_52_P284658 | *Cdkn2aipnl* |
| 0.0163811 | 69.24 | 130.15 | 0.53 | A_52_P432904 | *Ddx11* |
| 0.0185921 | 2665.67 | 5008.02 | 0.53 | A_52_P681787 | *Cyb561* |
| 0.0211996 | 1012.52 | 1901.55 | 0.53 | A_51_P212107 | *Ctnnbl1* |
| 0.0417476 | 221.27 | 415.06 | 0.53 | A_51_P409496 | *Arhgap11a* |
| 0.0409906 | 70.23 | 131.64 | 0.53 | A_51_P220062 | *Mmp15* |
| 0.0303815 | 179.91 | 337.21 | 0.53 | A_51_P313896 | *Anln* |
| 0.0429144 | 195.95 | 367.02 | 0.53 | A_51_P295206 | *Brip1* |
| 0.0480115 | 113.24 | 211.96 | 0.53 | A_52_P27878 | *Arhgef39* |
| 0.0292521 | 8.59 | 16.05 | 0.53 | A_51_P431999 | *B9d1os* |
| 0.0360551 | 24.81 | 48.18 | 0.52 | A_52_P554436 | *Rwdd2a* |
| 0.0238818 | 294.85 | 571.67 | 0.52 | A_52_P81677 | *ENSMUST00000117218* |
| 0.0151358 | 8.66 | 16.79 | 0.52 | A_52_P154564 | *Scrt1* |
| 0.0226117 | 2.71 | 5.25 | 0.52 | A_51_P239448 | *AK081574* |
| 0.0129725 | 1.87 | 3.62 | 0.52 | A_52_P285861 | *LF201862* |
| 0.0470412 | 1.24 | 2.4 | 0.52 | A_52_P245577 | *Sncb* |
| 0.040911 | 2267.95 | 4379.01 | 0.52 | A_51_P489779 | *Cdk6* |
| 0.0336898 | 38.91 | 75.1 | 0.52 | A_52_P183826 | *Dctd* |
| 0.0407252 | 584.28 | 1127.54 | 0.52 | A_51_P450033 | *Cdk1* |
| 0.0373974 | 62.37 | 120.18 | 0.52 | A_51_P121236 | *Pidd1* |
| 0.0339643 | 563.63 | 1085.73 | 0.52 | A_51_P485946 | *Fdft1* |
| 0.0369657 | 3004.31 | 5787.25 | 0.52 | A_51_P512541 | *Sdr39u1* |
| 0.0109564 | 3.39 | 6.53 | 0.52 | A_51_P123385 | *Olfr503* |
| 0.0304664 | 517.54 | 995.89 | 0.52 | A_51_P449935 | *Ftsj3* |
| 0.0208861 | 66.85 | 128.5 | 0.52 | A_52_P470586 | *NAP027971-1* |
| 0.0331845 | 17.22 | 33 | 0.52 | A_51_P331841 | *Ccdc122* |
| 0.0281903 | 134.94 | 258.52 | 0.52 | A_51_P187210 | *Nol11* |
| 0.0291252 | 62.64 | 119.97 | 0.52 | A_52_P231232 | *Nanos1* |
| 0.0143103 | 9.04 | 17.29 | 0.52 | A_51_P497768 | *Gpat2* |
| 0.0470228 | 369.06 | 705.02 | 0.52 | A_51_P254045 | *Traip* |
| 0.0422141 | 106.12 | 202.59 | 0.52 | A_51_P499233 | *C330027C09Rik* |
| 0.0426438 | 22.59 | 43.12 | 0.52 | A_51_P354018 | *Kcnk7* |
| 0.0146718 | 46.06 | 87.9 | 0.52 | A_51_P182503 | *Heatr3* |
| 0.037284 | 45.15 | 86.16 | 0.52 | A_51_P156222 | *Elfn1* |
| 0.0301449 | 14.82 | 28.26 | 0.52 | A_52_P512365 | *Cideb* |
| 0.0356216 | 225.02 | 428.98 | 0.52 | A_52_P595663 | *Knstrn* |
| 0.031856 | 2993.03 | 5705.68 | 0.52 | A_51_P155142 | *Cdca8* |
| 0.031523 | 193.66 | 368.88 | 0.52 | A_51_P159612 | *Hebp2* |
| 0.0183999 | 1.7 | 3.37 | 0.51 | A_51_P503722 | *Elavl4* |
| 0.0164759 | 2932.54 | 5806.66 | 0.51 | A_51_P202074 | *Ncapd2* |
| 0.0104871 | 272.89 | 539.93 | 0.51 | A_52_P23413 | *Prag1* |
| 0.0216402 | 13006.61 | 25732.75 | 0.51 | A_52_P420466 | *Hist1h2ab* |
| 0.0183252 | 913.38 | 1802.08 | 0.51 | A_52_P10683 | *Hist1h1d* |
| 0.036434 | 5.83 | 11.49 | 0.51 | A_51_P247542 | *Ttll6* |
| 0.0184524 | 338.8 | 666.48 | 0.51 | A_52_P116704 | *BY754910* |
| 0.0215324 | 1756.42 | 3452.55 | 0.51 | A_52_P13448 | *Pclaf* |
| 0.0157624 | 20.23 | 39.69 | 0.51 | A_51_P288978 | *9430002A10Rik* |
| 0.0340787 | 2.14 | 4.19 | 0.51 | A_52_P851299 | *AK042814* |
| 0.0157022 | 4.25 | 8.32 | 0.51 | A_52_P670599 | *NAP122837-1* |
| 0.0193566 | 11869.29 | 23214.44 | 0.51 | A_52_P594768 | *Aprt* |
| 0.0370144 | 87.37 | 170.64 | 0.51 | A_51_P285323 | *Bora* |
| 0.0133881 | 464.12 | 906.35 | 0.51 | A_51_P484537 | *Smc2* |
| 0.0155407 | 218.01 | 425.39 | 0.51 | A_51_P269687 | *Pole2* |
| 0.0448144 | 630.02 | 1228.6 | 0.51 | A_51_P369200 | *Tpx2* |
| 0.0442052 | 154.08 | 300.32 | 0.51 | A_51_P364168 | *Lrp5* |
| 0.0194511 | 902.66 | 1756.16 | 0.51 | A_51_P505521 | *Hist1h4i* |
| 0.0108059 | 1461.8 | 2840.81 | 0.51 | A_52_P348986 | *Atp2b1* |
| 0.0152159 | 536.41 | 1041.98 | 0.51 | A_51_P497402 | *Tonsl* |
| 0.0383778 | 1564.36 | 3156.4 | 0.5 | A_51_P190111 | *Mcm5* |
| 0.0212409 | 1139.78 | 2297.29 | 0.5 | A_51_P489268 | *Ncapg* |
| 0.0208241 | 708.53 | 1426.36 | 0.5 | A_52_P625111 | *Cisd3* |
| 0.0308073 | 10.48 | 21.08 | 0.5 | A_51_P499698 | *Asprv1* |
| 0.0298052 | 204.23 | 410.53 | 0.5 | A_52_P658437 | *Espl1* |
| 0.0206638 | 6 | 12.06 | 0.5 | A_51_P172155 | *Hal* |
| 0.0104939 | 1.08 | 2.17 | 0.5 | A_52_P851529 | *AK045794* |
| 0.010687 | 692.9 | 1387.82 | 0.5 | A_52_P104824 | *Diaph3* |
| 0.0474977 | 3879.97 | 7767.03 | 0.5 | A_51_P165704 | *Mcm7* |
| 0.0329894 | 2544.29 | 5089.59 | 0.5 | A_52_P222230 | *Gm40525* |
| 0.0181187 | 371.63 | 742.25 | 0.5 | A_51_P492830 | *Cenph* |
| 0.0416353 | 58.82 | 117.34 | 0.5 | A_51_P414927 | *Pigw* |
| 0.0201351 | 2.76 | 5.49 | 0.5 | A_51_P391934 | *Cpb1* |
| 0.0231073 | 35.64 | 70.89 | 0.5 | A_51_P412846 | *Efhd1* |
| 0.0272829 | 5.85 | 11.62 | 0.5 | A_52_P190763 | *Flicr* |
| 0.0194627 | 679.56 | 1348.12 | 0.5 | A_52_P39546 | *Tead2* |
| 0.0402815 | 8117.16 | 16077.03 | 0.5 | A_51_P131408 | *Tnfrsf12a* |
| 0.0120716 | 5.97 | 11.82 | 0.5 | A_52_P116811 | *NAP049688-1* |
| 0.0295981 | 22.2 | 45.76 | 0.49 | A_52_P645122 | *NAP029013-1* |
| 0.0076256 | 3.51 | 7.23 | 0.49 | A_52_P135000 | *AK081948* |
| 0.0442276 | 106.17 | 218.62 | 0.49 | A_51_P401263 | *Eme1* |
| 0.0218784 | 31.09 | 63.85 | 0.49 | A_52_P75348 | *Spdl1* |
| 0.0441119 | 3.25 | 6.67 | 0.49 | A_51_P376478 | *A_51_P376478* |
| 0.042577 | 48.21 | 98.91 | 0.49 | A_52_P151116 | *Kcnab3* |
| 0.0322528 | 5672.64 | 11634.37 | 0.49 | A_51_P273433 | *Prkg2* |
| 0.0247449 | 111.13 | 227.87 | 0.49 | A_51_P351970 | *Hells* |
| 0.0275244 | 519.32 | 1064.8 | 0.49 | A_51_P148105 | *Rad51* |
| 0.010397 | 2939.73 | 6023.17 | 0.49 | A_51_P209372 | *Msmo1* |
| 0.0179733 | 8281.02 | 16965.11 | 0.49 | A_51_P413348 | *Ezr* |
| 0.0454249 | 1338.38 | 2740.89 | 0.49 | A_51_P234044 | *1190005I06Rik* |
| 0.0108921 | 1826.3 | 3737.07 | 0.49 | A_52_P354373 | *Lockd* |
| 0.0128412 | 36.34 | 74.3 | 0.49 | A_52_P662054 | *Klra16* |
| 0.0088519 | 2.71 | 5.53 | 0.49 | A_51_P263749 | *Fbxo48* |
| 0.0094231 | 1958.71 | 3990.97 | 0.49 | A_52_P195922 | *Exosc6* |
| 0.0085605 | 490.39 | 998.37 | 0.49 | A_51_P130015 | *Ect2* |
| 0.0142941 | 1.98 | 4.03 | 0.49 | A_52_P722719 | *AK078177* |
| 0.0266684 | 4.95 | 10.07 | 0.49 | A_51_P521090 | *2210406O10Rik* |
| 0.0218826 | 10.63 | 21.62 | 0.49 | A_51_P154473 | *Mc1r* |
| 0.0386403 | 2193.06 | 4458.88 | 0.49 | A_51_P224013 | *Tk1* |
| 0.0094637 | 461.84 | 938.87 | 0.49 | A_52_P682382 | *Scd1* |
| 0.0371617 | 100.77 | 204.84 | 0.49 | A_52_P399584 | *Ckap2l* |
| 0.0195763 | 71.01 | 143.99 | 0.49 | A_52_P227391 | *Kif15* |
| 0.0084703 | 31.3 | 63.34 | 0.49 | A_52_P642947 | *Snora52* |
| 0.0092311 | 6.78 | 13.72 | 0.49 | A_52_P227481 | *Gnal* |
| 0.0102075 | 92.2 | 186.52 | 0.49 | A_51_P275016 | *Slc7a3* |
| 0.0189676 | 43.86 | 88.69 | 0.49 | A_51_P437883 | *Mzf1* |
| 0.0240806 | 3021.81 | 6109.62 | 0.49 | A_52_P313813 | *Nhp2* |
| 0.0222551 | 1615.33 | 3264.74 | 0.49 | A_51_P301207 | *Knop1* |
| 0.0154454 | 482.9 | 1013.91 | 0.48 | A_51_P165082 | *Tmem191c* |
| 0.0278725 | 85.83 | 180.16 | 0.48 | A_51_P347240 | *Lrr1* |
| 0.007955 | 190.5 | 399.76 | 0.48 | A_52_P113672 | *Chml* |
| 0.0264725 | 455 | 954.77 | 0.48 | A_51_P411917 | *Gata6* |
| 0.018284 | 298.18 | 624.39 | 0.48 | A_51_P509651 | *Cep55* |
| 0.0243167 | 119.46 | 250.01 | 0.48 | A_52_P636752 | *Cyp51* |
| 0.0474849 | 1.51 | 3.16 | 0.48 | A_51_P205524 | *Otor* |
| 0.012231 | 33.6 | 70.3 | 0.48 | A_51_P340336 | *Pcbp3* |
| 0.0277577 | 166.63 | 348.38 | 0.48 | A_51_P138909 | *H60c* |
| 0.0113805 | 650.48 | 1359.77 | 0.48 | A_52_P330214 | *Fanca* |
| 0.016601 | 188.14 | 393.27 | 0.48 | A_51_P232393 | *Myo5c* |
| 0.006722 | 3208.65 | 6706.67 | 0.48 | A_51_P265327 | *Mpp6* |
| 0.0240809 | 668.13 | 1392.01 | 0.48 | A_51_P379798 | *Fdps* |
| 0.0186513 | 78.97 | 164.35 | 0.48 | A_52_P255034 | *Parpbp* |
| 0.0093623 | 7.37 | 15.33 | 0.48 | A_51_P418147 | *Fn3krp* |
| 0.0178995 | 9.32 | 19.37 | 0.48 | A_51_P100625 | *Apon* |
| 0.0249132 | 282.59 | 587.12 | 0.48 | A_52_P226489 | *ENSMUST00000221823* |
| 0.014013 | 330.93 | 687.37 | 0.48 | A_52_P196388 | *Tmem8* |
| 0.0123402 | 33.42 | 69.39 | 0.48 | A_52_P502279 | *4732460I02Rik* |
| 0.0112542 | 192.26 | 398.94 | 0.48 | A_51_P201035 | *Cenpi* |
| 0.011759 | 9.75 | 20.22 | 0.48 | A_52_P287772 | *AK052728* |
| 0.0249065 | 101.6 | 210.7 | 0.48 | A_51_P270519 | *Apitd1* |
| 0.0363406 | 168.58 | 349.2 | 0.48 | A_51_P489285 | *Knl1* |
| 0.0416831 | 682.9 | 1413.15 | 0.48 | A_51_P483301 | *Prdx6b* |
| 0.0340624 | 15.4 | 31.82 | 0.48 | A_51_P450373 | *2310068J16Rik* |
| 0.0101987 | 3.89 | 8.37 | 0.47 | A_52_P308373 | *TC1603457* |
| 0.0378039 | 35.7 | 76.76 | 0.47 | A_52_P476075 | *Clspn* |
| 0.0222627 | 785.59 | 1687.36 | 0.47 | A_51_P198775 | *BC055324* |
| 0.0310909 | 34.84 | 74.73 | 0.47 | A_52_P290325 | *Depdc1a* |
| 0.0193738 | 425.22 | 911.54 | 0.47 | A_51_P454447 | *Pradc1* |
| 0.039772 | 771.87 | 1653.73 | 0.47 | A_52_P36591 | *Hmmr* |
| 0.0126279 | 266.95 | 571.83 | 0.47 | A_51_P195153 | *Gtse1* |
| 0.0100985 | 8517.82 | 18235.21 | 0.47 | A_52_P184352 | *Hist3h2a* |
| 0.0369136 | 179.21 | 383.39 | 0.47 | A_52_P670275 | *Kif14* |
| 0.0257502 | 5095.07 | 10894.22 | 0.47 | A_52_P479262 | *Col6a3* |
| 0.0071334 | 5.7 | 12.17 | 0.47 | A_52_P460800 | *Ascl3* |
| 0.0152743 | 495.83 | 1058.5 | 0.47 | A_51_P513530 | *Spag5* |
| 0.0179905 | 148.73 | 317.24 | 0.47 | A_52_P66205 | *Fancd2* |
| 0.0310772 | 116 | 246.69 | 0.47 | A_52_P27694 | *2810408A11Rik* |
| 0.0125293 | 5337.08 | 11331.55 | 0.47 | A_51_P253803 | *Mki67* |
| 0.0178473 | 1.22 | 2.59 | 0.47 | A_51_P342307 | *Olfr732* |
| 0.0288852 | 270.07 | 571.63 | 0.47 | A_51_P441843 | *Fanci* |
| 0.0120819 | 1449.15 | 3066.39 | 0.47 | A_52_P236088 | *Gdpd1* |
| 0.0194886 | 96.07 | 203.08 | 0.47 | A_52_P502771 | *Rad54b* |
| 0.0083474 | 830.36 | 1752.4 | 0.47 | A_52_P463109 | *Nolc1* |
| 0.0459676 | 4.77 | 10.06 | 0.47 | A_52_P1164457 | *AK048928* |
| 0.0122561 | 3499.39 | 7370.26 | 0.47 | A_51_P366931 | *Prc1* |
| 0.0128052 | 96.53 | 203.27 | 0.47 | A_51_P131442 | *Dpf1* |
| 0.0348683 | 7718.66 | 16921.67 | 0.46 | A_51_P502872 | *2200002D01Rik* |
| 0.0156759 | 2.6 | 5.69 | 0.46 | A_52_P495829 | *AF454394* |
| 0.0204595 | 3.4 | 7.44 | 0.46 | A_51_P371867 | *3830417A13Rik* |
| 0.0306083 | 114.49 | 250.4 | 0.46 | A_51_P206616 | *Cdc7* |
| 0.0241039 | 11.42 | 24.96 | 0.46 | A_51_P405129 | *Ccdc96* |
| 0.0060006 | 103.74 | 226.51 | 0.46 | A_52_P460537 | *Lpar2* |
| 0.0139973 | 141.18 | 308.24 | 0.46 | A_51_P111380 | *AF223568* |
| 0.0224352 | 623.87 | 1361.17 | 0.46 | A_51_P204402 | *Shcbp1* |
| 0.009558 | 91.1 | 198.68 | 0.46 | A_52_P364299 | *Mms22l* |
| 0.0187521 | 12.2 | 26.58 | 0.46 | A_51_P204160 | *Palb2* |
| 0.0202811 | 870.32 | 1895.75 | 0.46 | A_51_P324450 | *Pbp2* |
| 0.0064313 | 4.94 | 10.75 | 0.46 | A_51_P382214 | *Rab17* |
| 0.0486979 | 14.01 | 30.44 | 0.46 | A_52_P521607 | *LF197799* |
| 0.021857 | 2.61 | 5.67 | 0.46 | A_51_P497270 | *Mycs* |
| 0.0096131 | 42.75 | 92.83 | 0.46 | A_52_P421918 | *Helq* |
| 0.0355252 | 111.52 | 241.96 | 0.46 | A_51_P404377 | *Rnd2* |
| 0.0071055 | 1840.25 | 3991.28 | 0.46 | A_51_P164014 | *Cenpe* |
| 0.0201952 | 1.8 | 3.9 | 0.46 | A_51_P476798 | *Olfr2* |
| 0.0061427 | 8.39 | 18.14 | 0.46 | A_52_P770765 | *AK033165* |
| 0.0484549 | 6.11 | 13.21 | 0.46 | A_52_P143129 | *Eno4* |
| 0.0327041 | 121.09 | 260.51 | 0.46 | A_51_P109144 | *Grtp1* |
| 0.0281326 | 700.29 | 1572.45 | 0.45 | A_51_P155482 | *Pole* |
| 0.0348649 | 37.04 | 83.17 | 0.45 | A_52_P446677 | *Rhbdl2* |
| 0.0051929 | 5.54 | 12.43 | 0.45 | A_52_P586104 | *ENSMUST00000118669* |
| 0.0245161 | 410.31 | 917.99 | 0.45 | A_51_P254811 | *Kif4* |
| 0.0128376 | 65.09 | 145.47 | 0.45 | A_51_P135092 | *Kif2c* |
| 0.0303257 | 9.67 | 21.56 | 0.45 | A_51_P503297 | *Zfp786* |
| 0.0134765 | 42.94 | 95.69 | 0.45 | A_52_P734742 | *Lrrc73* |
| 0.0125545 | 217.68 | 485.07 | 0.45 | A_51_P394802 | *Fam111a* |
| 0.0109395 | 66.67 | 148.27 | 0.45 | A_52_P257026 | *AF039839* |
| 0.039685 | 1.8 | 4 | 0.45 | A_52_P675857 | *NAP062699-1* |
| 0.0455468 | 61.04 | 135.51 | 0.45 | A_51_P337708 | *Ovgp1* |
| 0.020661 | 93.36 | 207.17 | 0.45 | A_51_P309589 | *2700099C18Rik* |
| 0.0355452 | 270.31 | 599.51 | 0.45 | A_52_P379531 | *Timeless* |
| 0.0245717 | 479.14 | 1060.05 | 0.45 | A_51_P358633 | *Melk* |
| 0.0067298 | 118.55 | 262.14 | 0.45 | A_52_P208613 | *Fancb* |
| 0.017243 | 269.71 | 595.63 | 0.45 | A_51_P287093 | *Ccnb1* |
| 0.0461456 | 95.48 | 210.83 | 0.45 | A_51_P155582 | *Rad51ap1* |
| 0.0107511 | 185.66 | 409.9 | 0.45 | A_51_P231958 | *Zwilch* |
| 0.0181593 | 125.03 | 275.99 | 0.45 | A_52_P205951 | *Gen1* |
| 0.0350798 | 89.3 | 196.84 | 0.45 | A_51_P447248 | *Aqp11* |
| 0.0141723 | 21.24 | 46.81 | 0.45 | A_51_P166706 | *AK028934* |
| 0.0219052 | 1193.86 | 2627.11 | 0.45 | A_51_P398723 | *Flt1* |
| 0.0085264 | 118.8 | 272.8 | 0.44 | A_52_P639997 | *Nt5c3b* |
| 0.028738 | 327.88 | 751.77 | 0.44 | A_51_P415059 | *Aurkb* |
| 0.0053799 | 80.41 | 184.05 | 0.44 | A_51_P473438 | *D730045B01Rik* |
| 0.0238386 | 176.17 | 402.95 | 0.44 | A_52_P655890 | *Wdhd1* |
| 0.0120734 | 2082.65 | 4757.92 | 0.44 | A_51_P344566 | *Plk1* |
| 0.0048722 | 4.44 | 10.14 | 0.44 | A_52_P484956 | *Nnat* |
| 0.0362854 | 698.3 | 1594.71 | 0.44 | A_51_P493522 | *Kazald1* |
| 0.0152729 | 1774.76 | 4050.58 | 0.44 | A_51_P481920 | *Ccna2* |
| 0.0207163 | 223.93 | 510.51 | 0.44 | A_52_P497553 | *Dhfr* |
| 0.0152893 | 986.66 | 2248.93 | 0.44 | A_51_P239203 | *Mapk13* |
| 0.0424543 | 49.33 | 112.39 | 0.44 | A_52_P319181 | *Efcab11* |
| 0.0187164 | 1437.27 | 3271.11 | 0.44 | A_51_P150912 | *Aurka* |
| 0.0121823 | 6226.8 | 14160.43 | 0.44 | A_52_P498208 | *Hist1h2ak* |
| 0.0182808 | 41.57 | 94.31 | 0.44 | A_52_P320279 | *Inca1* |
| 0.0453055 | 4212.65 | 9552.11 | 0.44 | A_51_P308362 | *Ugt1a6b* |
| 0.0455026 | 111.67 | 252.94 | 0.44 | A_52_P251366 | *Neil3* |
| 0.010364 | 12.38 | 28.03 | 0.44 | A_52_P957031 | *Mroh2a* |
| 0.0056132 | 304.47 | 689.29 | 0.44 | A_51_P340355 | *Tarsl2* |
| 0.014715 | 30.78 | 69.57 | 0.44 | A_52_P184544 | *Ticrr* |
| 0.016497 | 309.81 | 699.26 | 0.44 | A_52_P529570 | *Nsl1* |
| 0.0353423 | 2.54 | 5.73 | 0.44 | A_51_P134184 | *Gm16982* |
| 0.0401003 | 61.78 | 139.26 | 0.44 | A_51_P229875 | *E330009J07Rik* |
| 0.0256557 | 1.3 | 2.93 | 0.44 | A_52_P166735 | *Zdbf2* |
| 0.0137915 | 2357.27 | 5312 | 0.44 | A_51_P493467 | *Kif22* |
| 0.0092381 | 4033.33 | 9486.44 | 0.43 | A_52_P651235 | *Hist1h2ai* |
| 0.0065733 | 91.68 | 215.48 | 0.43 | A_52_P101487 | *Ddn* |
| 0.0274906 | 1620.58 | 3808.45 | 0.43 | A_52_P314705 | *Uhrf1* |
| 0.0090472 | 480.82 | 1128.49 | 0.43 | A_51_P220222 | *Tacc3* |
| 0.0161705 | 8.52 | 19.99 | 0.43 | A_52_P668903 | *AK087716* |
| 0.0085968 | 131.57 | 308.61 | 0.43 | A_52_P190647 | *Mxd3* |
| 0.0337264 | 373.9 | 876.8 | 0.43 | A_52_P596755 | *Dnph1* |
| 0.0215526 | 7.07 | 16.55 | 0.43 | A_52_P459900 | *ENSMUST00000145158* |
| 0.0105154 | 268.02 | 627.18 | 0.43 | A_52_P668812 | *Sgo2a* |
| 0.0123041 | 3369.3 | 7876.96 | 0.43 | A_51_P361022 | *Cdc20* |
| 0.0041385 | 7.14 | 16.69 | 0.43 | A_51_P171728 | *Ceacam2* |
| 0.0158497 | 141.07 | 328.76 | 0.43 | A_51_P423608 | *Slc35g1* |
| 0.0348554 | 3.14 | 7.31 | 0.43 | A_52_P430411 | *1700029F12Rik* |
| 0.0411929 | 4.46 | 10.38 | 0.43 | A_52_P1076565 | *1110002J07Rik* |
| 0.0353157 | 2.65 | 6.16 | 0.43 | A_52_P540365 | *Mapk15* |
| 0.0349833 | 1.52 | 3.53 | 0.43 | A_51_P326259 | *A_51_P326259* |
| 0.0269744 | 50.73 | 117.64 | 0.43 | A_52_P391743 | *Vsig2* |
| 0.006609 | 1.86 | 4.31 | 0.43 | A_52_P525574 | *Klk1b21* |
| 0.020066 | 107.17 | 248.18 | 0.43 | A_51_P191649 | *Ndc80* |
| 0.033352 | 261.4 | 604.92 | 0.43 | A_52_P139650 | *Ska1* |
| 0.0396334 | 202.9 | 468.41 | 0.43 | A_51_P307964 | *Krt13* |
| 0.005867 | 11.05 | 25.5 | 0.43 | A_51_P466364 | *Arhgap19* |
| 0.0189625 | 2761.07 | 6368.76 | 0.43 | A_51_P457528 | *Ccnb2* |
| 0.0125916 | 1.35 | 3.25 | 0.42 | A_51_P123373 | *Fshr* |
| 0.0066556 | 28.37 | 68.24 | 0.42 | A_52_P293391 | *Tcam1* |
| 0.034804 | 224.04 | 536.3 | 0.42 | A_51_P122425 | *Ctf1* |
| 0.011689 | 3.27 | 7.82 | 0.42 | A_51_P436401 | *Ifna5* |
| 0.0357585 | 460.61 | 1100.92 | 0.42 | A_51_P273538 | *Syce2* |
| 0.0067614 | 224.1 | 534.73 | 0.42 | A_51_P303749 | *Depdc1b* |
| 0.0141319 | 1797.96 | 4286.99 | 0.42 | A_51_P133138 | *Kif20a* |
| 0.01849 | 246.15 | 586.56 | 0.42 | A_51_P469449 | *Ung* |
| 0.0121199 | 14.61 | 34.69 | 0.42 | A_52_P923720 | *AK040434* |
| 0.0415081 | 92.44 | 219.23 | 0.42 | A_52_P304947 | *Cenpn* |
| 0.0234418 | 77.82 | 184.51 | 0.42 | A_52_P161454 | *Ldhd* |
| 0.0043149 | 1.06 | 2.51 | 0.42 | A_52_P432245 | *Cbln4* |
| 0.0138288 | 1565.97 | 3707.59 | 0.42 | A_51_P125135 | *Cdca5* |
| 0.0075704 | 153.37 | 362.85 | 0.42 | A_51_P481398 | *Kif11* |
| 0.0301328 | 781.13 | 1845.71 | 0.42 | A_51_P506915 | *Pkp3* |
| 0.0315914 | 1581.63 | 3734.91 | 0.42 | A_51_P137433 | *Kifc1* |
| 0.0287456 | 727.44 | 1717.08 | 0.42 | A_52_P550734 | *Kifc5b* |
| 0.0371095 | 19.51 | 45.94 | 0.42 | A_51_P445765 | *Lrp8* |
| 0.0122404 | 167.03 | 412.04 | 0.41 | A_52_P318073 | *ENSMUST00000198525* |
| 0.0473501 | 9.04 | 22.3 | 0.41 | A_52_P213722 | *Rnf207* |
| 0.0062005 | 5.55 | 13.69 | 0.41 | A_52_P876207 | *Gm32273* |
| 0.0155549 | 8.31 | 20.49 | 0.41 | A_51_P359411 | *Gnat2* |
| 0.0110884 | 301.33 | 742.86 | 0.41 | A_52_P384574 | *Stard4* |
| 0.0043355 | 20.7 | 51.01 | 0.41 | A_52_P538117 | *Chadl* |
| 0.0155757 | 20.09 | 49.44 | 0.41 | A_52_P110534 | *Ttk* |
| 0.043512 | 18.18 | 44.64 | 0.41 | A_51_P101975 | *Fgf8* |
| 0.022596 | 38.75 | 94.95 | 0.41 | A_51_P493709 | *2810474C18Rik* |
| 0.0052444 | 475.16 | 1163.99 | 0.41 | A_51_P329711 | *Idi1* |
| 0.0324527 | 138.16 | 337.64 | 0.41 | A_52_P681495 | *Mtfr2* |
| 0.0035241 | 18.66 | 45.57 | 0.41 | A_52_P899565 | *Gm37125* |
| 0.0302679 | 51.01 | 124.49 | 0.41 | A_52_P415931 | *Cenpu* |
| 0.0210534 | 7.56 | 18.44 | 0.41 | A_51_P259186 | *Ppp1r42* |
| 0.011328 | 1128.35 | 2744.11 | 0.41 | A_51_P215077 | *Mgst3* |
| 0.009976 | 416.93 | 1012.99 | 0.41 | A_51_P372550 | *Cgref1* |
| 0.0228414 | 305.34 | 741.86 | 0.41 | A_51_P256706 | *Psmc3ip* |
| 0.0154533 | 1820.14 | 4410.67 | 0.41 | A_52_P481686 | *Wtip* |
| 0.0090029 | 187.3 | 452.95 | 0.41 | A_51_P230878 | *Mad2l1* |
| 0.0110532 | 1.77 | 4.28 | 0.41 | A_51_P183812 | *Slfn4* |
| 0.0343701 | 16.38 | 39.58 | 0.41 | A_52_P1196900 | *Gm36401* |
| 0.0092501 | 889.48 | 2147.81 | 0.41 | A_51_P232868 | *Neurl1b* |
| 0.005978 | 89.2 | 215.32 | 0.41 | A_51_P369252 | *Ddias* |
| 0.0199495 | 98.64 | 249.7 | 0.4 | A_51_P450623 | *Phlda2* |
| 0.0422598 | 11.54 | 29.2 | 0.4 | A_51_P463034 | *4930432K21Rik* |
| 0.0379458 | 1.66 | 4.2 | 0.4 | A_51_P423743 | *Cldn6* |
| 0.0194297 | 302.06 | 763.99 | 0.4 | A_51_P383489 | *Fignl1* |
| 0.0238218 | 3801.35 | 9598.39 | 0.4 | A_51_P270949 | *Hist1h1b* |
| 0.0207602 | 90.32 | 226.13 | 0.4 | A_51_P519791 | *Ska3* |
| 0.0070216 | 244.92 | 613.17 | 0.4 | A_51_P341041 | *Dscc1* |
| 0.0145087 | 45.07 | 112.53 | 0.4 | A_51_P195034 | *Esco2* |
| 0.0275234 | 11.12 | 27.71 | 0.4 | A_52_P627068 | *Disp2* |
| 0.0090047 | 30.08 | 74.51 | 0.4 | A_51_P459100 | *Stil* |
| 0.0133906 | 703.29 | 1741.86 | 0.4 | A_51_P487999 | *Sgo1* |
| 0.0132808 | 226.69 | 560.66 | 0.4 | A_52_P14686 | *Dtl* |
| 0.0057158 | 9884.53 | 24436.31 | 0.4 | A_51_P230103 | *Birc5* |
| 0.0166224 | 1.74 | 4.3 | 0.4 | A_51_P176042 | *Pklr* |
| 0.0167425 | 725.92 | 1883.66 | 0.39 | A_51_P455897 | *Pimreg* |
| 0.0035805 | 5510.58 | 14263.37 | 0.39 | A_51_P474643 | *Kpna2* |
| 0.0133039 | 8.27 | 21.21 | 0.39 | A_51_P355301 | *Cyp3a11* |
| 0.0057449 | 1.79 | 4.59 | 0.39 | A_51_P305896 | *Cftr* |
| 0.030255 | 2.81 | 7.2 | 0.39 | A_52_P1156085 | *AK047450* |
| 0.0096341 | 2.23 | 5.69 | 0.39 | A_52_P691728 | *AK086422* |
| 0.0247459 | 1.74 | 4.43 | 0.39 | A_51_P374981 | *Lvrn* |
| 0.0415879 | 2.23 | 5.66 | 0.39 | A_51_P501304 | *Plag1* |
| 0.0076895 | 47.97 | 127.84 | 0.38 | A_51_P217778 | *Aspm* |
| 0.0149308 | 455.99 | 1214.5 | 0.38 | A_51_P230098 | *Pbk* |
| 0.0184855 | 1499.29 | 3992.08 | 0.38 | A_51_P462249 | *Cks2* |
| 0.0040661 | 265.21 | 701.65 | 0.38 | A_52_P27602 | *Kif23* |
| 0.0106212 | 16.96 | 44.81 | 0.38 | A_51_P512627 | *Syngr4* |
| 0.0031302 | 3.22 | 8.46 | 0.38 | A_52_P540826 | *Zfp850* |
| 0.0070907 | 978.2 | 2569.94 | 0.38 | A_52_P30984 | *Cdkn3* |
| 0.0027149 | 5.15 | 13.53 | 0.38 | A_51_P487027 | *Kcnk2* |
| 0.0161637 | 63.01 | 165.23 | 0.38 | A_52_P244803 | *D630033O11Rik* |
| 0.0073821 | 44.44 | 116.42 | 0.38 | A_51_P483269 | *Prr11* |
| 0.0205373 | 1307.74 | 3422.62 | 0.38 | A_51_P450487 | *Sqle* |
| 0.0327244 | 33.43 | 87.45 | 0.38 | A_51_P386660 | *Faah* |
| 0.0086653 | 1.81 | 4.73 | 0.38 | A_51_P175601 | *4930578C19Rik* |
| 0.0230765 | 51.08 | 133.19 | 0.38 | A_51_P512969 | *Cilp2* |
| 0.0078898 | 601.29 | 1566.89 | 0.38 | A_51_P252157 | *Top2a* |
| 0.0038744 | 1730.98 | 4507.85 | 0.38 | A_52_P151320 | *Tnfaip8l1* |
| 0.0115541 | 46.47 | 120.95 | 0.38 | A_52_P673499 | *Shmt1* |
| 0.016622 | 1.73 | 4.74 | 0.37 | A_52_P1026762 | *NAP124210-1* |
| 0.0137121 | 89.52 | 245.27 | 0.37 | A_52_P463968 | *Nrtn* |
| 0.011375 | 216.59 | 591.41 | 0.37 | A_51_P520745 | *BC030867* |
| 0.00228 | 175.33 | 477.94 | 0.37 | A_51_P205242 | *Kif20b* |
| 0.0026905 | 35.71 | 97.03 | 0.37 | A_51_P357094 | *Ccsap* |
| 0.0367253 | 136.59 | 370.82 | 0.37 | A_51_P474169 | *Proser2* |
| 0.0064904 | 18.3 | 49.63 | 0.37 | A_52_P644114 | *Ccdc18* |
| 0.0066692 | 102.46 | 277.61 | 0.37 | A_52_P520466 | *Kif18b* |
| 0.0125677 | 3.04 | 8.21 | 0.37 | A_52_P1129952 | *NAP069146-1* |
| 0.0042619 | 1.65 | 4.44 | 0.37 | A_52_P651316 | *Cd79a* |
| 0.0043542 | 40.75 | 109.51 | 0.37 | A_52_P411003 | *Dlgap5* |
| 0.0256175 | 4.06 | 10.9 | 0.37 | A_51_P180423 | *Camp* |
| 0.0190953 | 7.21 | 20.28 | 0.36 | A_52_P844462 | *BB437157* |
| 0.0190108 | 600.08 | 1679.53 | 0.36 | A_52_P28806 | *Foxm1* |
| 0.003953 | 45.73 | 127.65 | 0.36 | A_51_P268673 | *2210016H18Rik* |
| 0.0251329 | 107.37 | 299.7 | 0.36 | A_51_P463087 | *Cenpm* |
| 0.0172601 | 1610.25 | 4491.05 | 0.36 | A_52_P628067 | *Cdca3* |
| 0.0437907 | 169.34 | 472.11 | 0.36 | A_52_P21 | *Ttc9* |
| 0.0134485 | 115.81 | 320.07 | 0.36 | A_51_P296796 | *Nt5dc2* |
| 0.0208429 | 2.85 | 7.85 | 0.36 | A_51_P470530 | *Tex45* |
| 0.0021158 | 467.97 | 1350.95 | 0.35 | A_51_P172085 | *Arhgdig* |
| 0.0038259 | 4.18 | 12.06 | 0.35 | A_51_P325223 | *Lin7b* |
| 0.0155696 | 75.84 | 218.14 | 0.35 | A_51_P318123 | *Ulbp1* |
| 0.0046749 | 41.33 | 118.77 | 0.35 | A_51_P362857 | *4930427A07Rik* |
| 0.0097391 | 1054.8 | 3014.47 | 0.35 | A_51_P472217 | *Sapcd2* |
| 0.0037537 | 1.93 | 5.51 | 0.35 | A_51_P432388 | *AK042221* |
| 0.0119383 | 58.22 | 165.18 | 0.35 | A_51_P279575 | *Cdc25c* |
| 0.012174 | 158.52 | 447.92 | 0.35 | A_51_P451338 | *Jag2* |
| 0.0079285 | 2.58 | 7.68 | 0.34 | A_52_P500418 | *LF194521* |
| 0.011058 | 11.17 | 33.12 | 0.34 | A_52_P221993 | *Klra6* |
| 0.0088176 | 1.32 | 3.91 | 0.34 | A_51_P152444 | *4930465K10Rik* |
| 0.0152666 | 14.61 | 42.98 | 0.34 | A_51_P142450 | *Tmem25* |
| 0.0018334 | 72.45 | 212.86 | 0.34 | A_51_P174943 | *Lamc2* |
| 0.0231706 | 3.94 | 11.48 | 0.34 | A_51_P231320 | *Mmp8* |
| 0.0066834 | 250.19 | 765.07 | 0.33 | A_51_P239984 | *Exo1* |
| 0.0073652 | 141.31 | 430.34 | 0.33 | A_52_P162099 | *Ckap2* |
| 0.0040977 | 1.32 | 3.98 | 0.33 | A_52_P667800 | *Gm41409* |
| 0.0421858 | 4.7 | 14.17 | 0.33 | A_52_P28548 | *A730090N16Rik* |
| 0.004931 | 394 | 1184.32 | 0.33 | A_51_P513682 | *Nuf2* |
| 0.0257021 | 98.11 | 294 | 0.33 | A_51_P446131 | *Gipc2* |
| 0.0064273 | 37.37 | 111.91 | 0.33 | A_51_P166873 | *Tmem79* |
| 0.0102107 | 100.22 | 300.07 | 0.33 | A_51_P273556 | *Fam83d* |
| 0.0031324 | 2.41 | 7.21 | 0.33 | A_52_P730743 | *AK046703* |
| 0.0043247 | 827.3 | 2469.95 | 0.33 | A_51_P161946 | *Epop* |
| 0.0073135 | 281.64 | 889.47 | 0.32 | A_51_P475523 | *Brca1* |
| 0.0233567 | 1.77 | 5.46 | 0.32 | A_52_P1075968 | *AK048224* |
| 0.0036491 | 1.43 | 4.66 | 0.31 | A_52_P305579 | *D330041H03Rik* |
| 0.0349902 | 34.06 | 115.41 | 0.3 | A_52_P338266 | *Rec114* |
| 0.0496239 | 69.69 | 235.88 | 0.3 | A_52_P571715 | *Troap* |
| 0.0085614 | 13.67 | 45.92 | 0.3 | A_52_P649568 | *Aipl1* |
| 0.0146175 | 17.29 | 57.98 | 0.3 | A_52_P360515 | *Celsr2* |
| 0.0423496 | 44.56 | 149.35 | 0.3 | A_51_P355629 | *Gas2* |
| 0.0392302 | 521.39 | 1743.83 | 0.3 | A_51_P221441 | *Mall* |
| 0.002751 | 1.43 | 4.77 | 0.3 | A_51_P274674 | *Olfr1282* |
| 0.0027306 | 9.87 | 32.77 | 0.3 | A_51_P306387 | *Klra4* |
| 0.0428888 | 30.95 | 102.72 | 0.3 | A_51_P147064 | *1600014C23Rik* |
| 0.0030962 | 107.9 | 357.11 | 0.3 | A_51_P118300 | *Sncg* |
| 0.0017937 | 3.74 | 12.33 | 0.3 | A_52_P418814 | *Pmch* |
| 0.0171879 | 5.92 | 19.43 | 0.3 | A_51_P128973 | *Apoa2* |
| 0.0351532 | 700.24 | 2430.43 | 0.29 | A_52_P598895 | *Npnt* |
| 0.0212428 | 69.35 | 239.95 | 0.29 | A_51_P340038 | *Sh2d5* |
| 0.0386833 | 227.65 | 777.56 | 0.29 | A_51_P349495 | *Mboat1* |
| 0.0266824 | 1.85 | 6.71 | 0.28 | A_52_P77732 | *Npn2* |
| 0.0130945 | 181.88 | 658.59 | 0.28 | A_51_P180091 | *Cyp2s1* |
| 0.0404097 | 928.37 | 3354.81 | 0.28 | A_52_P653654 | *Atp1a3* |
| 0.0468769 | 7.92 | 28.31 | 0.28 | A_51_P501735 | *Gria4* |
| 0.0257008 | 12.7 | 45.3 | 0.28 | A_51_P191909 | *Rem2* |
| 0.0124393 | 5.58 | 19.75 | 0.28 | A_51_P510156 | *Lcn2* |
| 0.0024233 | 37.64 | 132.63 | 0.28 | A_51_P518621 | *Hist1h3a* |
| 0.0163692 | 1555.33 | 5466.23 | 0.28 | A_51_P333622 | *2010300C02Rik* |
| 0.026711 | 12.06 | 42.34 | 0.28 | A_51_P419226 | *S100a14* |
| 0.0042465 | 100.67 | 376.42 | 0.27 | A_51_P501018 | *Nek2* |
| 0.0021641 | 3.38 | 12.63 | 0.27 | A_51_P159803 | *Nrg4* |
| 0.0027733 | 4.64 | 17.02 | 0.27 | A_52_P980341 | *AK084342* |
| 0.0061759 | 123.51 | 449.79 | 0.27 | A_52_P216672 | *Klk8* |
| 0.0297128 | 7.6 | 29.56 | 0.26 | A_52_P489202 | *4732465J04Rik* |
| 0.002975 | 658.13 | 2556.47 | 0.26 | A_51_P246119 | *Cenpf* |
| 0.0012348 | 559.77 | 2146.68 | 0.26 | A_52_P248513 | *Klra7* |
| 0.0032989 | 17.64 | 67.56 | 0.26 | A_52_P678053 | *Ush1g* |
| 0.0404066 | 123.46 | 471.01 | 0.26 | A_51_P208922 | *Stc2* |
| 0.0356486 | 293.75 | 1115.86 | 0.26 | A_51_P123745 | *Spns2* |
| 0.0014088 | 362.38 | 1469.79 | 0.25 | A_51_P132079 | *Klra22* |
| 0.0011572 | 21.16 | 83.45 | 0.25 | A_52_P380263 | *Podxl* |
| 0.0008465 | 125.03 | 527.67 | 0.24 | A_52_P288892 | *Klra12* |
| 0.0009314 | 1.47 | 6.18 | 0.24 | A_51_P101787 | *Hapln1* |
| 0.0028715 | 7.97 | 33.3 | 0.24 | A_51_P236755 | *Clic3* |
| 0.0004669 | 12.67 | 56 | 0.23 | A_51_P410703 | *Prss8* |
| 0.0120163 | 43.91 | 193.06 | 0.23 | A_51_P494655 | *Angptl8* |
| 0.0164643 | 5.58 | 24.47 | 0.23 | A_52_P184322 | *Trpm6* |
| 0.025623 | 3.9 | 17.08 | 0.23 | A_52_P134341 | *2010110K18Rik* |
| 0.0494563 | 12.64 | 54.99 | 0.23 | A_51_P429308 | *Neto2* |
| 0.0116808 | 32.45 | 141.1 | 0.23 | A_51_P249909 | *Lect2* |
| 0.0015599 | 917.16 | 3904.32 | 0.23 | A_52_P514407 | *Klra15* |
| 0.0473225 | 2.5 | 10.63 | 0.23 | A_51_P120688 | *AK086706* |
| 0.0103993 | 1.65 | 7.6 | 0.22 | A_51_P106900 | *Rax* |
| 0.0010677 | 988.97 | 4433.59 | 0.22 | A_52_P244193 | *Cd24a* |
| 0.0007011 | 84.89 | 402.5 | 0.21 | A_51_P298741 | *Klra20* |
| 0.0419019 | 23.13 | 109.27 | 0.21 | A_51_P173656 | *Tubg2* |
| 0.0121282 | 391.14 | 1970.87 | 0.2 | A_51_P324838 | *Evpl* |
| 0.0047505 | 267.87 | 1331.59 | 0.2 | A_52_P297009 | *Itpk1* |
| 0.0442195 | 462.3 | 2291.96 | 0.2 | A_51_P222773 | *Foxa2* |
| 0.0008215 | 6.18 | 30.34 | 0.2 | A_52_P541353 | *Il2* |
| 0.0202215 | 23.78 | 128.12 | 0.19 | A_51_P462533 | *Syt7* |
| 0.0161199 | 2.13 | 11.24 | 0.19 | A_52_P429450 | *Ngp* |
| 0.0004131 | 122.15 | 634.71 | 0.19 | A_52_P335218 | *A_52_P335218* |
| 0.0011852 | 22.56 | 127.45 | 0.18 | A_51_P448464 | *Masp2* |
| 0.0410484 | 24.96 | 136.58 | 0.18 | A_51_P302327 | *Lgals7* |
| 0.0024398 | 14.86 | 80.6 | 0.18 | A_51_P240986 | *Plekhg6* |
| 0.0451371 | 2.24 | 13.5 | 0.17 | A_51_P415455 | *6720473M11Rik* |
| 0.0266442 | 4.19 | 24.73 | 0.17 | A_52_P31370 | *Iqca* |
| 0.0113959 | 4.14 | 24.08 | 0.17 | A_51_P476518 | *Fam83g* |
| 0.002291 | 10.2 | 68.22 | 0.15 | A_52_P425839 | *Retnlg* |
| 0.0009236 | 19.04 | 136.04 | 0.14 | A_51_P443976 | *Ptprr* |
| 0.0186978 | 13.71 | 109.44 | 0.13 | A_52_P445360 | *Krt20* |
| 0.0344961 | 2.4 | 18.51 | 0.13 | A_51_P255238 | *Plac1* |
| 0.0018785 | 162.74 | 1238.03 | 0.13 | A_51_P235945 | *Hp* |
| 0.0130336 | 4.22 | 34.83 | 0.12 | A_51_P108226 | *Wfdc21* |
| 0.000494 | 30.19 | 265.36 | 0.11 | A_51_P227275 | *Csn3* |
| 0.0454209 | 6.37 | 62.07 | 0.1 | A_51_P296517 | *Esrp2* |
| 0.017203 | 57.21 | 550.19 | 0.1 | A_51_P256827 | *S100a8* |
| 0.000157 | 30.68 | 329.54 | 0.093 | A_51_P153683 | *Lrrc26* |
| 0.0310615 | 13.21 | 144.42 | 0.091 | A_52_P295432 | *Cxcl5* |
| 0.0402543 | 1.93 | 21.97 | 0.088 | A_51_P101347 | *Pls1* |
| 0.0139924 | 184.68 | 2163.56 | 0.085 | A_51_P402943 | *S100a9* |
| 0.0451091 | 1.51 | 18.81 | 0.08 | A_52_P559975 | *Cxcr2* |
| 0.016801 | 1080.71 | 18028.54 | 0.06 | A_51_P356642 | *Krt19* |
| 0.001037 | 3.07 | 57.13 | 0.054 | A_52_P672689 | *Btc* |
| 0.0130992 | 105.96 | 1986.26 | 0.053 | A_51_P473383 | *Tenm4* |
| 0.0086553 | 1.98 | 90.31 | 0.022 | A_51_P220278 | *Ppp2r2b* |
| 4.02E-05 | 86.96 | 4413.16 | 0.02 | A_51_P255875 | *Padi4* |
| 0.0309126 | 192.44 | 25000.19 | 0.0077 | A_51_P465211 | *Wfdc2* |
| 0.001995 | 4.1 | 699.72 | 0.0059 | A_51_P514270 | *Add2* |

| **Supplementary Table S5.** MetaCore Enrichment Analysis by Pathway Maps of 1684 genes upregulated in tumor tissue by CSTA (genes downregulated in tumor tissue without CSTA; gene set A). The top 50 pathway maps are listed (p-value < 1E−06, FDR < 1E−04). | | | | | | |
| --- | --- | --- | --- | --- | --- | --- |
| **#** | **Maps** | **Total** | **p-value** | **FDR** | **In Data** | **Network Objects from Active Data** |
| 1 | Immune response_IFN-gamma in macrophages activation | 50 | 1.051E-16 | 1.421E-13 | 24 | *CCL5, PU.1, COX-2 (PTGS2), IRF8, Fc gamma RI, MIG, CD40(TNFRSF5), iNOS, p67-phox, IP10, IRF1, C1qb, BATF2, CCL2, Thrombospondin 1, IL-10, HAF1, TLR4, Selenoprotein P, IFN-gamma, Gbp4 (mouse), gp91-phox, STAT1, C1qc* |
| 2 | COVID-19: immune dysregulation | 100 | 1.449E-15 | 9.795E-13 | 32 | *CCL5, MHC class I, MIP-1-beta, MIG, GATA Group, TIM-3, IL-10 receptor, HLA-E, MHC class II, UNC93B, CXCL16, CCR5, TLR8, iNOS, IL-4, F263, IP10, CSF1, IRF1, MDA-5, CCL2, IL-10, ITGAL, NKG2A, CCL8, Btk, Granzyme B, Perforin, IFN-gamma, FasR(CD95), HLA-DMA, CCL7* |
| 3 | Chemokines in inflammation in adipose tissue and liver in obesity, type 2 diabetes and metabolic syndrome X | 48 | 6.893E-15 | 1.975E-12 | 22 | *ITGAX, CCL5, Fc gamma RI, MANR, FCGR3A, MHC class II, VCAM1, CCR5, CD45, CX3CL1, ITGA4, EMR1, ICAM1, CCL2, ITGAL, CD34, PSGL-1, TLR4, CX3CR1, CD86, CXCR4, TLR2* |
| 4 | Immune response_Classical complement pathway | 53 | 7.303E-15 | 1.975E-12 | 23 | *C1r, C2, DAF, C4a, iC3b, C1, C3dg, C1q, C1 inhibitor, C6, C2b, C3a, C3, C3b, C4, C1s, C3aR, C1qRp, C2a, C3 convertase (C2aC4b), C5 convertase (C2aC4bC3b), C3c, C4b* |
| 5 | Immune response_Induction of the antigen presentation machinery by IFN-gamma | 53 | 7.303E-15 | 1.975E-12 | 23 | *CIITA, H2-Aa, H2-Eb1, MHC class I, PSMB9, TAP1 (PSF1), Beta-2-microglobulin, HLA-E, MHC class II, HLA-F, HLAC, CD74, PSMB8(LMP7), NLRC5, IRF1, Tapasin, Cathepsin S, PSMB10, IFN-gamma, TAP2 (PSF2), STAT1, ERAP1, HLA-DMA* |
| 6 | Putative pathways of activation of classical complement system in major depressive disorder | 28 | 4.110E-13 | 9.262E-11 | 16 | *C1qa, C1, C4A protein, C1qb, C1q, C6, C3, C4B protein, C3b, C4, IFN-gamma, C1qRp, C1qc, C3 convertase (C2aC4b), C5 convertase (C2aC4bC3b), C4b* |
| 7 | Down-regulation of mast cell functions through ITIM-containing inhibitory receptors in asthma | 37 | 5.568E-13 | 1.075E-10 | 18 | *Syk, MHC class I, SHPS-1, Fc epsilon RI, Fyn, CD72, SHP-1, Fc epsilon RI beta, SHIP, Csk, CD200 (OX-2), CCL2, PIRB, c-Kit, Fc epsilon RI gamma, LAIR1, Lyn, Fc epsilon RI alpha* |
| **Supplementary Table S5.** (Continued) | | | | | | |
| **#** | **Maps** | **Total** | **p-value** | **FDR** | **In Data** | **Network Objects from Active Data** |
| 8 | Immune response_Antigen presentation by MHC class I: cross-presentation | 99 | 2.680E-12 | 4.530E-10 | 28 | *Syk, MHC class I, Dectin-1, LY75, Fc gamma RI, Fc epsilon RI, TIM-3, SREC-I, MANR, FCGR3A, Rac2, DAP12, FCGRT, CD40(TNFRSF5), CD74, p67-phox, Cytochrome b-558, C1q, Fc epsilon RI gamma, SNAP-23, Cathepsin S, TAP, TLR4, IGTP, VAV-1, TLR2, gp91-phox, p47-phox* |
| 9 | Immune response_IFN-alpha/beta signaling via JAK/STAT | 62 | 3.850E-12 | 5.784E-10 | 22 | *MHC class I, TAP1 (PSF1), OAS1, MIG, STAT2, IRF9, IFI27, Apo-2L(TNFSF10), IP10, GBP4, IRF1, Mx1, CCL2, ISGF3, ISG54, XAF1, IFI47, IFN-gamma, STAT1, RIG-G, IFNAR2, STAT1/STAT2* |
| 10 | Role of B cells in SLE | 58 | 7.602E-12 | 1.028E-09 | 21 | *Syk, MHC class I, CD28, Fc gamma RII beta, TACI(TNFRSF13B), CD22, MHC class II, ICOS-L, PI3K cat class IA, SHP-1, CD40(TNFRSF5), CD45, SHIP, APRIL(TNFSF13), ICAM1, Lyn, Btk, CD86, IFN-gamma, TNF-beta, BAFF(TNFSF13B)* |
| 11 | Macrophage and dendritic cell phenotype shift in cancer | 100 | 2.182E-11 | 2.682E-09 | 27 | *COX-2 (PTGS2), MHC class I, EPAS1, IL-10 receptor, MHC class II, SHP-1, GM-CSF receptor, Gas6, CD40(TNFRSF5), iNOS, Apo-2L(TNFSF10), IL-4, SHIP, IP10, CSF1, ESR2 (nuclear), Thrombospondin 1, IRF4, IL-10, M-CSF receptor, TLR4, CD86, IFN-gamma, IRF5, TLR2, STAT1, SOCS1* |
| 12 | Immune response_Lectin induced complement pathway | 50 | 2.830E-11 | 3.189E-09 | 19 | *C2, DAF, C4a, iC3b, C3dg, C1 inhibitor, C6, C2b, C3a, C3, C3b, C4, C3aR, C1qRp, C2a, C3 convertase (C2aC4b), C5 convertase (C2aC4bC3b), C3c, C4b* |
| 13 | Dysregulation of germinal center response in SLE | 65 | 9.228E-11 | 9.597E-09 | 21 | *TGM2, Fc gamma RII beta, TACI(TNFRSF13B), IL-15RA, IRF9, VCAM1, CD40(TNFRSF5), IL-21 receptor, C3dg, IL-4, APRIL(TNFSF13), Oct-2, ICAM1, Bcl-6, IRF4, CD79B, PI3K cat class IA (p110-delta), FasR(CD95), STAT1, BAFF(TNFSF13B), CXCL13* |
| 14 | Attenuation of IFN type I signaling in melanoma cells | 37 | 1.040E-10 | 1.004E-08 | 16 | *MHC class I, PSMB9, TAP1 (PSF1), STAT2, IRF9, PSMB8(LMP7), Apo-2L(TNFSF10), IP10, IRF1, Tapasin, ISGF3, TAP, VAV-1, TAP2 (PSF2), STAT1, IFNAR2* |
| **Supplementary Table S5.** (Continued) | | | | | | |
| **#** | **Maps** | **Total** | **p-value** | **FDR** | **In Data** | **Network Objects from Active Data** |
| 15 | Common mechanisms of Th17 cell migration | 48 | 1.225E-10 | 1.104E-08 | 18 | *CCL5, CD161, MIP-1-beta, G-protein beta/gamma, MIG, CXCL16, VCAM1, CCR5, IP10, PLC-beta, ICAM1, CCL2, PI3K reg class IB (p101), PSGL-1, CCL8, G-protein alpha-i family, PI3K cat class IB (p110-gamma), CCL7* |
| 16 | Immune response_Antimicrobial actions of IFN-gamma | 43 | 1.507E-10 | 1.213E-08 | 17 | *MIP-1-beta, IIGP, GBP7, p67-phox, Factor B, IRF1, Tgtp1, p22-phox, LRG-47, Nod1, IGTP, IFI47, IFN-gamma, STAT1, Caspase-4, GBP2, C1RA* |
| 17 | Mast cell migration in asthma | 73 | 1.526E-10 | 1.213E-08 | 22 | *CCL5, G-protein beta/gamma, Fc epsilon RI, MIG, PTAFR, CXCL16, PI3K reg (p87-gamma), IP10, PDGF receptor, CCL2, TGF-beta 3, Fibronectin, c-Kit, Adenosine A3 receptor, C3a, PI3K reg class IB (p101), TGF-beta receptor type II, G-protein alpha-i family, C3aR, CXCR4, PI3K cat class IB (p110-gamma), IL-3 receptor* |
| 18 | Cooperative action of IFN-gamma and TNF-alpha on astrocytes in multiple sclerosis | 39 | 2.717E-10 | 2.025E-08 | 16 | *CIITA, MHC class I, HLA-E, MHC class II, PKA-reg (cAMP-dependent), Beta-2 adrenergic receptor, iNOS, IP10, IRF1, ICAM1, CCL2, Adenylate cyclase, CD86, IFN-gamma, STAT1, C/EBPdelta* |
| 19 | NK cells in allergic contact dermatitis | 34 | 2.846E-10 | 2.025E-08 | 15 | *CIITA, CCL5, MIG, MHC class II, CCR5, IP10, IRF1, ICAM1, CCL2, KLRK1 (NKG2D), NKG2A, Perforin, IFN-gamma, FasR(CD95), STAT1* |
| 20 | Role of integrins in eosinophil degranulation in asthma | 58 | 5.569E-10 | 3.765E-08 | 19 | *CCL5, FCGR3A, PI3K cat class IA, VCAM1, Hck, GM-CSF receptor, PKC, CCL13, ICAM1, Fibronectin, CysLT1 receptor, CSF2RB, C3b, Plastin, G-protein alpha-i family, PKC-beta2, IFN-gamma, PI3K cat class IB (p110-gamma), CCL7* |
| 21 | Role of Bregs in attenuation of T and NK cells mediated anti-tumor immune responses | 41 | 6.625E-10 | 4.265E-08 | 16 | *CD28, MIG, IL-10 receptor, 5'-NTD, IL-4, IP10, CD1d, IL-10, ENP1, TGF-beta receptor type II, Granzyme B, LAG3, CD86, IFN-gamma, FasR(CD95), PD-L1* |
| 22 | Basophil migration in asthma | 55 | 1.616E-09 | 9.501E-08 | 18 | *CCL5, FPRL1, G-protein beta/gamma, Fc epsilon RI, VCAM1, GM-CSF receptor, CCL13, ICAM1, CCL2, Fibronectin, PI3K reg class IB (p101), PSGL-1, CCL8, G-protein alpha-i family, PLAU (UPA), PI3K cat class IB (p110-gamma), IL-3 receptor, CCL7* |
| **Supplementary Table S5.** (Continued) | | | | | | |
| **#** | **Maps** | **Total** | **p-value** | **FDR** | **In Data** | **Network Objects from Active Data** |
| 23 | Immune response_Immunological synapse formation | 55 | 1.616E-09 | 9.501E-08 | 18 | *RASSF5, MHC class I, CD28, WASP, Fyn, MHC class II, PI3K cat class IA, VCAM1, Slp76, FYB1, PREL1, ICAM1, LAT, GRAP2, PDZ-GEF1, CD86, VAV-1, WaspIP* |
| 24 | Degranulation of lung mast cells | 63 | 2.705E-09 | 1.524E-07 | 19 | *Syk, G-protein beta/gamma, Fc epsilon RI, cPKC (conventional), Fyn, PTAFR, PI3K cat class IA, Beta-2 adrenergic receptor, Slp76, Tryptase, PGE2R3, c-Kit, Adenosine A3 receptor, Lyn, G-protein alpha-i family, VAV-1, PI3K cat class IB (p110-gamma), Chymase, PLC-beta2* |
| 25 | Cell adhesion_Integrin inside-out signaling in neutrophils | 77 | 3.044E-09 | 1.646E-07 | 21 | *Syk, Fc gamma RI, G-protein beta/gamma, PTAFR, Protein kinase G1, DAP12, Hck, iNOS, Slp76, FYB1, PREL1, ICAM1, PI3K reg class IB (p101), PSGL-1, Lyn, Btk, G-protein alpha-i family, PI3K cat class IB (p110-gamma), Protein kinase G, PREX1, PLC-beta2* |
| 26 | Glomerular injury in Lupus Nephritis | 92 | 3.469E-09 | 1.804E-07 | 23 | *CCL5, TGF-beta, Protein kinase G1, VCAM1, PDGF-B, iNOS, CX3CL1, PKC-beta1, IP10, CSF1, IRF1, ICAM1, MDA-5, CCL2, p22-phox, Fibronectin, TWEAK(TNFSF12), C3a, CaMK IV, PKC-beta2, IFN-gamma, FasR(CD95), STAT1* |
| 27 | IFN-gamma and Th2 cytokines-induced inflammatory signaling in normal and asthmatic airway epithelium | 40 | 4.383E-09 | 2.195E-07 | 15 | *CCL5, IL-2R gamma chain, MIG, CXCL16, IL-4, IP10, IRF1, ICAM1, CCL2, TSLP, IGF-1, Fibronectin, IFN-gamma, STAT1, SOCS1* |
| 28 | Oxidative stress_Activation of NADPH oxidase | 59 | 5.776E-09 | 2.789E-07 | 18 | *G-protein beta/gamma, cPKC (conventional), PI3K cat class IA, Rac2, PI3K reg (p87-gamma), p40-phox, PKC, p67-phox, PLC-beta, Cytochrome b-558, p22-phox, PI3K reg class IB (p101), PKC-beta2, VAV-1, PI3K cat class IB (p110-gamma), gp91-phox, p47-phox, PREX1* |
| 29 | Immune response_Antigen presentation by MHC class II | 118 | 6.079E-09 | 2.834E-07 | 26 | *MHC class II alpha chain, Syk, Dectin-1, LY75, Fc gamma RII beta, Cathepsin F, MANR, FCGR3A, MHC class II, PI3K cat class IA, Langerin, FCGRT, CD74, MARCH1, PKC, SPPL2a, MHC class II beta chain, Fc epsilon RI gamma, HCLS1, Cathepsin S, HLA-DM, TAP, TLR4, CD79B, TLR2, SWAP-70* |
| **Supplementary Table S5.** (Continued) | | | | | | |
| **#** | **Maps** | **Total** | **p-value** | **FDR** | **In Data** | **Network Objects from Active Data** |
| 30 | Immune response_PIP3 signaling in B lymphocytes | 42 | 9.596E-09 | 4.240E-07 | 15 | *Syk, Fc gamma RII beta, PKC-beta, G-protein beta/gamma, PI3K cat class IA, CD45, BCAP, SHIP, PLC-beta, PI3K reg class IB (p101), Lyn, Btk, VAV-1, PI3K cat class IB (p110-gamma), PREX1* |
| 31 | Oxidative stress_Role of IL-8 signaling pathway in respiratory burst | 48 | 9.721E-09 | 4.240E-07 | 16 | *G-protein beta/gamma, Rac2, PKC-beta1, p40-phox, p67-phox, Cytochrome b-558, p22-phox, PI3K reg class IB (p101), Btk, G-protein alpha-i family, PKC-beta2, PI3K cat class IB (p110-gamma), gp91-phox, p47-phox, PREX1, PLC-beta2* |
| 32 | Immune response_IL-9 signaling pathway | 61 | 1.044E-08 | 4.411E-07 | 18 | *CCL5, IL-2R gamma chain, Sca-1, PI3K cat class IA, MC-CPA, VCAM1, IL-4, CCL13, CCL2, Mcpt4 (rodent), Pim-1, Granzyme B, Mcpt2 (mouse), IFN-gamma, STAT1, Chymase, CCL7, Fc epsilon RI alpha* |
| 33 | Role of tumor-infiltrating B cells in anti-tumor immunity | 91 | 1.483E-08 | 6.076E-07 | 22 | *MHC class I, G-protein beta/gamma, MIG, NXF2, MHC class II, CD40(TNFRSF5), IL-21 receptor, Apo-2L(TNFSF10), IL-4, IP10, Bcl-6, IRF4, Btk, G-protein alpha-i family, Granzyme B, Perforin, CXCR4, IFN-gamma, FasR(CD95), STAT1, BLIMP1 (PRDI-BF1), CXCL13* |
| 34 | SLE genetic marker-specific pathways in B cells | 99 | 1.578E-08 | 6.275E-07 | 23 | *Syk, IRF8, Fc gamma RII beta, TACI(TNFRSF13B), CD22, STAT2, IRF9, Ikaros, SHP-1, p67-phox, NOX4, Bcl-6, p22-phox, Lyn, Btk, TLR4, VAV-1, IRF5, STAT1, BLIMP1 (PRDI-BF1), BLNK, BAFF(TNFSF13B), NOX4/p22-phox* |
| 35 | Populations of skin dendritic cells involved in contact hypersensitivity | 18 | 1.708E-08 | 6.599E-07 | 10 | *ITGAX, LY75, MHC class II, CD83, Langerin, CD40(TNFRSF5), CD45, EMR1, CD1d, CD86* |
| 36 | Role of CD8+ Tc1 cells in COPD | 44 | 1.996E-08 | 7.494E-07 | 15 | *CD69, MHC class I, MIG, CXCL16, CCR5, CX3CL1, IP10, CCL2, KLRK1 (NKG2D), Cathepsin S, Antileukoproteinase 1, Granzyme B, CX3CR1, Perforin, IFN-gamma* |
| 37 | Renal tubulointerstitial injury in Lupus Nephritis | 65 | 3.152E-08 | 1.152E-06 | 18 | *CCL5, TACI(TNFRSF13B), MHC class II, VCAM1, CCR5, CD40(TNFRSF5), APRIL(TNFSF13), CSF1, ICAM1, CCL2, Fibronectin, TWEAK(TNFSF12), M-CSF receptor, PLAU (UPA), IFN-gamma, BAFF(TNFSF13B), CXCL13, SOCS1* |
| **Supplementary Table S5.** (Continued) | | | | | | |
| **#** | **Maps** | **Total** | **p-value** | **FDR** | **In Data** | **Network Objects from Active Data** |
| 38 | Immune response_Fc epsilon RI pathway: Lyn-mediated cytokine production | 87 | 3.248E-08 | 1.155E-06 | 21 | *Syk, BFL1, PKC-beta, Fc epsilon RI beta, PKC-beta1, Slp76, IL-4, FYB1, PKC, CCL2, TSLP, LAT, GRAP2, Fc epsilon RI gamma, GATA-2, Lyn, Btk, VAV-1, NF-AT, MEF2C, Fc epsilon RI alpha* |
| 39 | Inhibition of mast cell functions by Siglecs in asthma | 30 | 6.790E-08 | 2.320E-06 | 12 | *Syk, Fc epsilon RI, CD22, SHP-1, Fc epsilon RI beta, Slp76, LAT, GRAP2, CD33, Fc epsilon RI gamma, Lyn, Fc epsilon RI alpha* |
| 40 | Signal transduction_MIF signaling pathway | 61 | 6.865E-08 | 2.320E-06 | 17 | *PU.1, Syk, COX-2 (PTGS2), G-protein beta/gamma, PI3K cat class IA, VCAM1, CD74, PLC-beta, SPPL2a, ICAM1, SFK, PI3K reg class IB (p101), Lyn, G-protein alpha-i family, TLR4, CXCR4, PI3K cat class IB (p110-gamma)* |
| 41 | Immune escape mechanisms in Prostate Cancer | 25 | 7.393E-08 | 2.438E-06 | 11 | *MHC class I, TAP1 (PSF1), Beta-2-microglobulin, TGF-beta, IL-4, CCL2, IL-10, TAP, TLR4, IFN-gamma, TAP2 (PSF2)* |
| 42 | Immune response _CCR3 signaling in eosinophils | 77 | 1.004E-07 | 3.231E-06 | 19 | *CCL5, G-protein beta/gamma, WASP, Rac2, Hck, iNOS, p67-phox, CCL13, Cytochrome b-558, p22-phox, MyHC, PI3K reg class IB (p101), CCL8, G-protein alpha-i family, PI3K cat class IB (p110-gamma), gp91-phox, p47-phox, CCL7, PLC-beta2* |
| 43 | Rheumatoid arthritis (general schema) | 50 | 1.389E-07 | 4.368E-06 | 15 | *CD28, Fc gamma RI, TGF-beta, FCGR3A, MHC class II, VCAM1, CD40(TNFRSF5), CSF1, ICAM1, MHC class II beta chain, TLR4, CD86, IFN-gamma, TLR2, BAFF(TNFSF13B)* |
| 44 | Development_Negative regulation of WNT/Beta-catenin signaling at the receptor level | 45 | 2.194E-07 | 6.741E-06 | 14 | *CDON, PEDF (serpinF1), Glypican-6, IBP4, SFRP, DAB2, ANGPTL4, WNT, WIF1, APCDD1, Axin, GRB10, Frizzled, Syndecans* |
| 45 | Immune response_IFN-gamma actions on blood cells | 28 | 3.012E-07 | 8.998E-06 | 11 | *PU.1, IRF8, K12, IL-4, IRF1, Bcl-6, IFN-gamma, STAT1, PD-L1, BAFF(TNFSF13B), SOCS1* |
| 46 | B-regulatory cells and tumor cells intercellular interaction | 67 | 3.061E-07 | 8.998E-06 | 17 | *FPRL1, CD28, gp130, GM-CSF receptor, CD40(TNFRSF5), IL-21 receptor, IL-4, EBI3, CCL2, Bcl-6, IL-10, TLR4, Granzyme B, CD86, FasR(CD95), STAT1, PD-L1* |
| **Supplementary Table S5.** (Continued) | | | | | | |
| **#** | **Maps** | **Total** | **p-value** | **FDR** | **In Data** | **Network Objects from Active Data** |
| 47 | Immune response_Alternative complement pathway | 53 | 3.254E-07 | 9.360E-06 | 15 | *DAF, PTX3, iC3b, C3dg, Factor B, C6, C3a, C3, C5 convertase (C3b2Bb), Factor Ba, C3b, Factor Bb, C3aR, C3 convertase (C3bBb), C3c* |
| 48 | Eosinophil adhesion and transendothelial migration in asthma | 68 | 3.856E-07 | 1.086E-05 | 17 | *CCL5, PTAFR, VCAM1, GM-CSF receptor, PKC, CCL13, ICAM1, Fibronectin, c-Kit, CysLT1 receptor, C3a, PSGL-1, C3aR, PLAU (UPA), PI3K cat class IB (p110-gamma), IL-3 receptor, CCL7* |
| 49 | Immune response_IL-3 signaling via JAK/STAT, p38, JNK and NF-kB | 93 | 5.212E-07 | 1.429E-05 | 20 | *CD69, PU.1, TACI(TNFRSF13B), MHC class II, PI3K cat class IA, IL3RA, CD40(TNFRSF5), IL-4, Spi2a, ICAM1, Bcl-6, Fibronectin, CSF2RB, Lyn, Pim-1, Granzyme B, C3aR, STAT1, IL-3 receptor, SOCS1* |
| 50 | Immune response_IL-10 signaling pathway | 62 | 5.310E-07 | 1.429E-05 | 16 | *COX-2 (PTGS2), Fc gamma RI, Fc gamma RII beta, IKBZ, IL-10 receptor, HPGD, MHC class II, PI3K cat class IA, iNOS, SHIP, IL10RA, ICAM1, IL10RB, IL-10, CD86, STAT1* |

| **Supplementary Table S6.** MetaCore Enrichment Analysis by Pathway Maps of 771 genes downregulated in tumor tissue by CSTA (genes upregulated in tumor tissue without CSTA; gene set B). The top 50 pathway maps are listed (p-value < 1E−02, FDR < 5E−02). | | | | | | |
| --- | --- | --- | --- | --- | --- | --- |
| **#** | **Maps** | **Total** | **p-value** | **FDR** | **In Data** | **Network Objects from Active Data** |
| 1 | Cell cycle_The metaphase checkpoint | 36 | 3.267E-21 | 3.113E-18 | 18 | *Nek2A, INCENP, CENP-A, DSN1, Aurora-A, PLK1, Aurora-B, HEC, CDCA1, CDC20, CENP-F, Zwilch, MAD2a, Survivin, CENP-H, CENP-E, NSL1, AF15q14* |
| 2 | Cell cycle_Chromosome condensation in prometaphase | 21 | 3.105E-19 | 1.480E-16 | 14 | *INCENP, CAP-G, Cyclin A, CNAP1, CAP-G/G2, Aurora-A, CAP-D2/D3, Aurora-B, CAP-E, Cyclin B, TOP2, Histone H1, Histone H3, CDK1 (p34)* |
| 3 | DNA damage_ATM/ATR regulation of G2/M checkpoint: nuclear signaling | 45 | 4.657E-16 | 1.479E-13 | 16 | *CDC25C, WDHD1, CDC18L (CDC6), Cyclin B1, p53, PALB2, Cyclin A, Claspin, Chk1, PLK1, GTSE1, Brca1, Cyclin B, Cyclin B2, TTK, CDK1 (p34)* |
| 4 | Cell cycle_Role of APC in cell cycle regulation | 32 | 1.020E-15 | 2.430E-13 | 14 | *Nek2A, CDC18L (CDC6), Tome-1, Geminin, Cyclin A, Aurora-A, PLK1, Aurora-B, Kid, CDC20, Cyclin B, MAD2a, CDK1 (p34), CKS1* |
| 5 | DNA damage_Intra S-phase checkpoint | 76 | 1.307E-15 | 2.490E-13 | 19 | *TOPBP1, CDC18L (CDC6), FANCD2, DTL (hCdt2), p53, RAD18, Cyclin A, Claspin, Chk1, FANCI (KIAA1794), p38 MAPK, MCM7, Brca1, BRIP1, CDC7, MCM2, Histone H3, MCM5, PIDD* |
| 6 | Cell cycle_Spindle assembly and chromosome separation | 33 | 5.855E-14 | 9.300E-12 | 13 | *Nek2A, Importin (karyopherin)-alpha, TPX2, Aurora-A, KNSL1, Aurora-B, HEC, Kid, CDC20, Cyclin B, MAD2a, Separase, CDK1 (p34)* |
| 7 | Cell cycle_Initiation of mitosis | 26 | 6.766E-11 | 9.211E-09 | 10 | *CDC25C, Cyclin B1, PLK1, KNSL1, Cyclin B2, FOXM1, Kinase MYT1, Histone H1, Histone H3, CDK1 (p34)* |
| 8 | DNA damage_ATM/ATR regulation of G2/M checkpoint: cytoplasmic signaling | 51 | 5.519E-10 | 6.575E-08 | 12 | *CDC25C, Cyclin B1, BORA, PP2A regulatory, Chk1, Aurora-A, PLK1, Aurora-B, p38 MAPK, Brca1, Histone H3, CDK1 (p34)* |
| 9 | Cell cycle_Role of Nek in cell cycle regulation | 32 | 7.336E-10 | 7.768E-08 | 10 | *Nek2A, Tubulin gamma, Cyclin B1, TPX2, Aurora-A, HEC, MAD2a, Histone H1, Histone H3, CDK1 (p34)* |
| 10 | Abnormalities in cell cycle in SCLC | 29 | 5.724E-09 | 5.455E-07 | 9 | *Cyclin B1, p53, Cyclin A, Cyclin D1, Aurora-B, Histone H3, CDK1 (p34), CKS1, CDK6* |
| **Supplementary Table S6.** (Continued) | | | | | | |
| **#** | **Maps** | **Total** | **p-value** | **FDR** | **In Data** | **Network Objects from Active Data** |
| 11 | NETosis in SLE | 31 | 1.110E-08 | 9.615E-07 | 9 | *IFN-alpha, PAD4, Histone H2, Histone H2A, p38 MAPK, Histone H4, CAMP, Histone H1, Histone H3* |
| 12 | Cell cycle_Transition and termination of DNA replication | 26 | 4.473E-08 | 3.552E-06 | 8 | *TOP2 alpha, Cyclin A, Brca1, MCM2, TOP2, CDK1 (p34), POLD cat (p125), DNA ligase I* |
| 13 | DNA damage_G2 checkpoint in response to DNA mismatches | 30 | 1.556E-07 | 1.140E-05 | 8 | *CDC25C, TOPBP1, p53, Claspin, Chk1, EXO1, Brca1, CDK1 (p34)* |
| 14 | Cell cycle_Nucleocytoplasmic transport of CDK/Cyclins | 14 | 2.378E-07 | 1.619E-05 | 6 | *Importin (karyopherin)-alpha, Cyclin B1, Cyclin A, Cyclin D1, Cyclin D, CDK1 (p34)* |
| 15 | Role of histone modificators in progression of multiple myeloma | 30 | 2.558E-06 | 1.625E-04 | 7 | *EZH2, Cyclin D1, Jagged2, Histone H4, Caspase-3, Histone H3, CDK6* |
| 16 | Cell cycle_Cell cycle (generic schema) | 21 | 3.788E-06 | 2.256E-04 | 6 | *CDC25C, Cyclin A, Cyclin B, Cyclin D, CDK1 (p34), CDK6* |
| 17 | Cell cycle_Start of DNA replication in early S phase | 32 | 4.077E-06 | 2.286E-04 | 7 | *CDC18L (CDC6), Geminin, ORC6L, CDC7, MCM2, Histone H1, MCM5* |
| 18 | SCAP/SREBP Transcriptional Control of Cholesterol and FA Biosynthesis | 45 | 4.337E-06 | 2.296E-04 | 8 | *SREBP2 (nuclear), SCD, ERG1, SREBP2 precursor, FDFT1, SREBP2 (Golgi membrane), FDPS, IDI1* |
| 19 | Cell cycle_Sister chromatid cohesion | 23 | 6.796E-06 | 3.409E-04 | 6 | *Cyclin B, DCC1, Separase, Histone H1, Histone H3, CDK1 (p34)* |
| 20 | Reproduction_Progesterone-mediated oocyte maturation | 40 | 1.951E-05 | 9.296E-04 | 7 | *CDC25C, Cyclin B1, Aurora-A, PLK1, CDC20, Kinase MYT1, CDK1 (p34)* |
| 21 | Brca1 and Brca2 in breast cancer | 19 | 3.954E-05 | 1.795E-03 | 5 | *Tubulin gamma, Rad51, Chk1, Brca1, BRIP1* |
| 22 | Main chemotherapy drugs and their action in SCLC cells | 100 | 5.471E-05 | 2.370E-03 | 10 | *TOP2 alpha, p53, Cyclin A, Cyclin D1, JNK1(MAPK8), JNK(MAPK8-10), p38 MAPK, Caspase-3, Cyclin D, CDK1 (p34)* |
| 23 | Epigenetic alterations in ovarian cancer | 82 | 6.217E-05 | 2.576E-03 | 9 | *GATA-6, Aurora-A, EZH2, Aurora-B, CDC20, Brca1, Histone H3, Gamma-synuclein, CDK6* |
| 24 | Inhibition of tumor suppressive pathways in pancreatic cancer | 21 | 6.681E-05 | 2.653E-03 | 5 | *p53, Rad51, Cyclin D1, PIDD, CDK6* |
| 25 | Cell cycle_Role of 14-3-3 proteins in cell cycle regulation | 22 | 8.496E-05 | 3.239E-03 | 5 | *CDC25C, p53, Chk1, p38 MAPK, CDK1 (p34)* |
| 26 | IL-6 signaling in colorectal cancer | 37 | 1.217E-04 | 4.460E-03 | 6 | *Cyclin B1, p53, Cyclin D1, Cyclin B, Survivin, CDK1 (p34)* |
| **Supplementary Table S6.** (Continued) | | | | | | |
| **#** | **Maps** | **Total** | **p-value** | **FDR** | **In Data** | **Network Objects from Active Data** |
| 27 | Cell cycle_Regulation of G1/S transition (part 1) | 38 | 1.419E-04 | 5.010E-03 | 6 | *PP2A regulatory, Cyclin A, Cyclin D1, Brca1, Cyclin D, CDK6* |
| 28 | Apoptosis and survival_DNA-damage-induced apoptosis | 15 | 2.335E-04 | 7.948E-03 | 4 | *FANCD2, p53, Chk1, Brca1* |
| 29 | Cell cycle_Regulation of G1/S transition (part 2) | 28 | 2.854E-04 | 9.380E-03 | 5 | *Cyclin A2, Cyclin A, Cyclin D1, Cyclin D, CDK6* |
| 30 | DNA damage_ATM/ATR regulation of G1/S checkpoint | 44 | 3.259E-04 | 1.009E-02 | 6 | *p53, PP2A regulatory, Cyclin A, Chk1, Cyclin D1, Brca1* |
| 31 | Cell cycle_Role of SCF complex in cell cycle regulation | 29 | 3.389E-04 | 1.009E-02 | 5 | *Chk1, PLK1, Cyclin D1, CDK1 (p34), CKS1* |
| 32 | Apoptosis and survival_p53-dependent apoptosis | 29 | 3.389E-04 | 1.009E-02 | 5 | *p53, JNK(MAPK8-10), Brca1, Caspase-3, CDK1 (p34)* |
| 33 | DNA damage_Role of Brca1 and Brca2 in DNA repair | 30 | 3.997E-04 | 1.154E-02 | 5 | *FANCD2, p53, Rad51, Brca1, BRIP1* |
| 34 | Development_Histone acetylation in embryonic stem cells | 34 | 7.277E-04 | 2.016E-02 | 5 | *p53, GATA-6, HNF3-beta, Histone H4, Histone H3* |
| 35 | DNA damage_DNA-damage-induced responses | 9 | 7.426E-04 | 2.016E-02 | 3 | *p53, Chk1, Brca1* |
| 36 | Development_H3K36 demethylation in stem cell maintenance | 20 | 7.617E-04 | 2.016E-02 | 4 | *p53, EZH2, Histone H3, CDK6* |
| 37 | Cell cycle_ESR1 regulation of G1/S transition | 35 | 8.343E-04 | 2.149E-02 | 5 | *Cyclin A2, Cyclin A, Cyclin D1, CKS1, CDK6* |
| 38 | Notch signaling in breast cancer | 53 | 9.026E-04 | 2.264E-02 | 6 | *Cyclin B1, Cyclin A, Cyclin D1, HURP, Jagged2, Survivin* |
| 39 | Cytoskeleton remodeling_Keratin filaments | 36 | 9.522E-04 | 2.327E-02 | 5 | *Envoplakin, JNK(MAPK8-10), Keratin 19, Keratin 13, CDK1 (p34)* |
| 40 | dCTP/dUTP metabolism | 74 | 1.014E-03 | 2.416E-02 | 7 | *POLE1, PD-ECGF (TdRPase), DCTD, TK1, NDPK 7, POLE2, POLD cat (p125)* |
| 41 | Inhibition of RUNX3 signaling in gastric cancer | 22 | 1.112E-03 | 2.585E-02 | 4 | *EZH2, Cyclin D1, Caspase-3, Histone H3* |
| **Supplementary Table S6.** (Continued) | | | | | | |
| **#** | **Maps** | **Total** | **p-value** | **FDR** | **In Data** | **Network Objects from Active Data** |
| 42 | Regulation of lipid metabolism_Regulation of lipid metabolism via LXR, NF-Y and SREBP | 38 | 1.224E-03 | 2.778E-02 | 5 | *CYP51A1, SREBP2 (nuclear), SCD, SREBP2 precursor, SREBP2 (Golgi membrane)* |
| 43 | HGF signaling in melanoma | 39 | 1.380E-03 | 2.989E-02 | 5 | *MC1R, Cyclin D1, JNK(MAPK8-10), p38 MAPK, Plakoglobin* |
| 44 | Cell cycle progression in Prostate Cancer | 39 | 1.380E-03 | 2.989E-02 | 5 | *Cyclin D1, p38 MAPK, Cyclin B, CDK1 (p34), CDK6* |
| 45 | Cholesterol Biosynthesis | 103 | 1.644E-03 | 3.481E-02 | 8 | *CYP51A1, SC4MOL, ERG1, FDFT1, DHC24, MVD, FDPS, IDI1* |
| 46 | DNA damage_p53 activation by DNA damage | 60 | 1.738E-03 | 3.600E-02 | 6 | *p53, PP2A regulatory, Chk1, JNK(MAPK8-10), p38 MAPK, Brca1* |
| 47 | GLP-1 in inhibition of beta cell proliferation and function in type 2 diabetes | 42 | 1.936E-03 | 3.925E-02 | 5 | *Cyclin A2, Pdx-1 (IPF1), Cyclin D1, HNF3-beta, Betacellulin* |
| 48 | Transcription_Sirtuin6 regulation and functions | 63 | 2.236E-03 | 4.439E-02 | 6 | *SREBP2 (nuclear), SCD, SREBP2 precursor, KPYR, SREBP2 (Golgi membrane), Histone H3* |
| 49 | Leptin signaling in colorectal cancer | 44 | 2.387E-03 | 4.642E-02 | 5 | *Cyclin B1, Cyclin D1, JNK(MAPK8-10), Survivin, CDK1 (p34)* |
| 50 | Brca1 in ovarian cancer | 27 | 2.453E-03 | 4.675E-02 | 4 | *p53, Brca1, BRIP1, Caspase-3* |

| **Supplementary Table S7.** Geneset Class Comparison Analysis by cell type-related gene sets (BRB-ArrayTools and referenced curated gene sets) that were upregulated in tumor tissue with CSTA (downregulated in tumor tissues without CSTA). Differentially expressed gene sets were identified by the LS/KS permutation test or the Efron-Tibshirani test (p < 0.05). Heatmaps for each resultant gene set are depicted in online supplementary figure S4. | | | | | |
| --- | --- | --- | --- | --- | --- |
| **GeneList GeneSets** | **Number of genes** | **LS permutation p-value** | **KS permutation p-value** | **Efron-Tibshirani's GSA test p-value** | **Annotation/Gene Set collection Reference** |
| M1 biomarkers | 17 | < 0.00001 | 0.00001 | < 0.005 | [1, 2] |
| Neutrophils Garley2018 | 42 | < 0.00001 | 0.00002 | < 0.005 | [3] |
| Thymic_SP_CD4+Tcell_gt_Thymic_DP_Tcell | 84 | < 0.00001 | < 0.00001 | < 0.005 | [4, 5] |
| Th1 Radens2020 | 67 | 0.00001 | 0.00008 | 0.13 | [6] |
| HAY_BONE_MARROW_CD8_T_CELL | 50 | 0.00002 | 0.00206 | < 0.005 | [7] |
| Th2 Radens2020 | 118 | 0.00002 | 0.00026 | 0.135 | [6] |
| DC1_CD141+CLEC9A+_Villani_2017 | 85 | 0.00003 | 0.00233 | < 0.005 | [8] |
| Palmer_2006_CD8+T cell | 14 | 0.0003 | 0.00005 | < 0.005 | [9] |
| Dendritic_cell_CD16pos_blood | 36 | 0.00277 | 0.00096 | 0.17 | [4, 10] |
| DC5_AXL+SIGLEC6+_Villani_2017 | 73 | 0.00297 | 0.07746 | 0.185 | [8] |
| DC2_CD1C_A+_Villani_2017 | 55 | 0.003 | 0.02221 | 0.305 | [8] |
| Resting_panT_cell_CD4+CD8+_GNF | 55 | 0.00982 | 0.00241 | 0.13 | [4, 11] |
| Dendritic_cell_BDCA3pos_blood | 59 | 0.012 | 0.10163 | 0.13 | [4, 10] |
| M2 biomarkers | 20 | 0.0347 | 0.13667 | 0.305 | [1, 2] |
| T_NK_Node5808 | 11 | 0.04071 | 0.01987 | 0.13 | [4, 11] |
| DC1, dendritic cell 1; DC2, dendritic cell 2; DC5, dendritic cell 5.[8] | | | | | |

# **Supplementary References**

1. Nasti A, Sakai Y, Seki A, et al (2017) The CD45 ^+^ fraction in murine adipose tissue derived stromal cells harbors immune-inhibitory inflammatory cells: Immunomodulation and immune therapies. Eur J Immunol 47:2163–2174

2. Jablonski KA, Amici SA, Webb LM, Ruiz-Rosado J de D, Popovich PG, Partida-Sanchez S, Guerau-de-Arellano M (2015) Novel markers to delineate murine M1 and M2 macrophages. PloS One 10:e0145342

3. Garley M, Jabłońska E (2018) Heterogeneity among neutrophils. Arch Immunol Ther Exp (Warsz) 66:21–30

4. Staudt LM (Lab) Signature Database. In: Staudt. https://lymphochip.nih.gov/signaturedb/. Accessed 28 Jun 2023

5. Lee MS, Hanspers K, Barker CS, Korn AP, McCune JM (2004) Gene expression profiles during human CD4+ T cell differentiation. Int Immunol 16:1109–1124

6. Radens CM, Blake D, Jewell P, Barash Y, Lynch KW (2020) Meta-analysis of transcriptomic variation in T-cell populations reveals both variable and consistent signatures of gene expression and splicing. RNA N Y N 26:1320–1333

7. Hay SB, Ferchen K, Chetal K, Grimes HL, Salomonis N (2018) The Human Cell Atlas bone marrow single-cell interactive web portal. Exp Hematol 68:51–61

8. Villani A-C, Satija R, Reynolds G, Sarkizova S, Shekhar K, Fletcher J, Griesbeck M, Butler A, Zheng S, Lazo S (2017) Single-cell RNA-seq reveals new types of human blood dendritic cells, monocytes, and progenitors. Science 356:eaah4573

9. Palmer C, Diehn M, Alizadeh AA, Brown PO (2006) Cell-type specific gene expression profiles of leukocytes in human peripheral blood. BMC Genomics 7:115

10. Lindstedt M, Lundberg K, Borrebaeck CAK (2005) Gene family clustering identifies functionally associated subsets of human in vivo blood and tonsillar dendritic cells. J Immunol Baltim Md 1950 175:4839–4846

11. Su AI, Wiltshire T, Batalov S, Lapp H, Ching KA, Block D, Zhang J, Soden R, Hayakawa M, Kreiman G (2004) A gene atlas of the mouse and human protein-encoding transcriptomes. Proc Natl Acad Sci 101:6062–6067

# **Supplementary Figure Legends**

**Supplementary Figure S1.** Establishment of the PAN02-CSTA-GFP cell line. (A) Flow cytometry scatter plots showing the successful expression of *CSTA-GFP* in the PAN02 cell line (left panel) compared with the control PAN02 cell line (right panel). (B) Comparison of cell proliferation.

**Supplementary Figure S2.** Intraperitoneal (IP) pancreatic ductal adenocarcinoma (PDAC) model mice established with PAN02-CSTA-GFP or PAN02 cell lines. (A) Survival curves of IP PDAC model mice. The following conditions were compared: PAN02-CSTA-GFP IP PDAC model of wild-type (WT) mice (n = 10), PAN02-CSTA-GFP IP PDAC model of nude mice (n = 10), and PAN02 IP PDAC model of WT mice (n = 10). The log-rank test was performed to determine p-values; *p < 0.05, ***p<0.001, ****p < 0.0001. (B-D) Representative images, taken on day 65 from establishment, of the IP dissemination in the PAN02-CSTA-GFP IP PDAC model of WT mice (B), the PAN02-CSTA-GFP IP PDAC model of nude mice (C), and the PAN02 IP PDAC model of WT mice (D) (n = 2). Images show the abdominal cavity of mice, specifically the spleen and pancreas (square) and tumor masses in the mesentery and along the intestine (arrow).

**Supplementary Figure S3.** Establishment of Retro-PAN02-CSTA and Retro-PAN02-CSTA-GFP cell lines. (A) *Cystatin A1* (*Csta1*) gene expression assessment by quantitative real-time PCR (qRT-PCR) in the Retro-PAN02-CSTA cell line tested with progressive concentrations of doxycycline (DOX; n = 1). (B) Testing of *CSTA-GFP* induction after the addition of 1 μg/mL doxycycline to the medium of the Retro-PAN02-CSTA-GFP cell line; three time points were assessed: 24, 48, and 72 h (n = 2). (C) Scheme of the *in vivo* establishment of the Retro-PAN02-CSTA-GFP SC PDAC model and sample collection after induction of *CSTA-GFP*. (D) *Csta1* gene expression assessment by qRT-PCR in isolated tumors of Retro-PAN02-CSTA-GFP induced or not by doxycycline (n = 4). (B, D) Bars represent the mean ± SD. (D) Student’s t-test was used for the statistical analysis; ****p < 0.0001.

**Supplementary Figure S4.** DNA microarray analysis of PDAC tumor tissues in the presence or not of *cystatin A* (*CSTA*) expression. Doxycycline was supplied in water to the Retro-PAN02-CSTA SC PDAC model mice from day 65 to day 84 for the induction of *CSTA*; the tumors were sampled on day 84 and RNA was isolated and used for DNA microarray analysis. Geneset Class Comparison Analysis by cell type-related gene sets: differentially expressed gene sets were identified by the log score/Kolmogorov-Smirnov (LS/KS) permutation test or the Efron-Tibshirani test (p < 0.05); heatmaps for each resultant gene set are depicted.

**Supplementary Figure S5.** Flow cytometry (FCM) analysis of lymphoid and myeloid lineage cells in tumor-infiltrating inflammatory cells (TICs) isolated from subcutaneous (SC) pancreatic ductal adenocarcinoma (PDAC) tumors of mice where *cystatin A* (*CSTA*) expression was either induced or not. The representative scatter plots of FCM measurements depicted in figure 4 are illustrated for each immune cell type: (A) CD3e+CD45+ TICs, (B) CD4+ subpopulation in CD3e+CD45+ TICs, and (C) T-bet+ (T helper type 1; Th1) subpopulation in CD4+CD3e+CD45+ TICs and IFN-γ+ subpopulation in Th1 TICs. (D) Gata3+ (T helper type 2; Th2) subpopulation in CD4+CD3e+CD45+ TICs and IL-5+ subpopulation in Th2 TICs. (E) CD8+ subpopulation in CD3e+CD45+ TICs and IFN-γ subpopulation in CD8+CD3e+CD45+ TICs. (F) CD86+ subpopulation in CD11b+ TICs (M1 macrophages) and CD206+ subpopulation in CD11b+ TICs (M2 macrophages). (G) CD11c+MHCII+ subpopulation in CD45+ TICs (dendritic cells; DCs) and activated CD83+ subpopulation in DCs TICs.

**Supplementary Figure S6.** Gene expression analysis of tumor-infiltrating inflammatory cells (TICs) from subcutaneous (SC) pancreatic ductal adenocarcinoma (PDAC) model mice by quantitative real-time PCR (qRT-PCR). Doxycycline was supplied in water from day 65 to day 84 to the Retro-PAN02-CSTA SC PDAC model mice for the induction of *cystatin A* (*CSTA*); the tumors were sampled and TICs isolated on day 84, followed by RNA isolation. Quantitative RT-PCR showing the expression of (A) T cell activation markers (*Il2ra* and *Cd69*), (B) immune checkpoint markers (*Pdcd1*, *Havcr2*, *Lag3*, and *Tigit*); *CSTA* not induced group, n = 7, *CSTA* induced group, n = 6; bars represent the mean ± SEM; a two-tailed Mann–Whitney test was used for statistical analysis; *p < 0.05.

**Supplementary Figure S7.** Flow cytometry (FCM) analysis of splenocytes (SPCs) for CD4+ immune lineage cells and the expression of their immune checkpoint molecules. Briefly, doxycycline was supplied in water from day 65 to day 84 to Retro-PAN02-CSTA SC PDAC model mice for the induction of *cystatin A* (*CSTA*); the spleens were sampled on day 84 for SPC isolation, followed by FCM analysis. (A) Representative scatter plots of FCM measurements are illustrated: CD45+ subpopulation in SPCs, CD4+ subpopulation in CD45+ SPCs, PD-1+ subpopulation in CD4+CD45+ SPCs, LAG-3+ subpopulation in CD4+CD45+ SPCs, and TIM-3+ subpopulation in CD4+CD45+ SPCs. (B) Graphs with data plots showing the percentage frequency of PD-1+, LAG-3+, and TIM-3+ subpopulations in CD4+CD45+ SPCs; *CSTA* induced condition, n = 6; *CSTA* not induced condition, n = 7. Bars represent the mean ± SEM; Student’s t-test was used for statistical analysis.

**Supplementary Figure S8.** Flow cytometry (FCM) analysis of splenocytes (SPCs) for CD8+ immune lineage cells and the expression of their immune checkpoint molecules. Briefly, doxycycline was supplied in water from day 65 to day 84 to Retro-PAN02-CSTA SC PDAC model mice for the induction of *cystatin A* (*CSTA*); the spleens were sampled on day 84 for SPC isolation, followed by FCM analysis. (A) Representative scatter plots of FCM measurements are illustrated: CD45+ subpopulation in SPCs, CD8+ subpopulation in CD45+ SPCs, PD-1+ subpopulation in CD8+CD45+ SPCs, LAG-3+ subpopulation in CD8+CD45+ SPCs, and TIM-3+ subpopulation in CD8+CD45+ SPCs. (B) Graphs with data plots showing the percentage frequency of PD-1+, LAG-3+, and TIM-3+ subpopulations in CD8+CD45+ SPCs; *CSTA* induced condition, n = 6; *CSTA* not induced condition, n = 7. Bars represent the mean ± SEM; Student’s t-test was used for statistical analysis.

**Supplementary Figure S9.** Flow cytometry (FCM) analysis of splenocytes (SPCs) for CD335+ natural killer (NK) cells and the expression of their immune checkpoint molecules. Briefly, doxycycline was supplied in water from day 65 to day 84 to Retro-PAN02-CSTA SC PDAC model mice for the induction of *cystatin A* (*CSTA*); the spleens were sampled on day 84 for SPC isolation, followed by FCM analysis. (A) Representative scatter plots of FCM measurements are illustrated: CD45+ subpopulation in SPCs and CD335+ subpopulation in CD45+ SPCs (NK cells). (B) Graphs with data plots showing the percentage frequency of the CD335+ subpopulation in CD45+ SPCs. (C) Representative scatter plots of FCM measurements are illustrated: CD45+ subpopulation in SPCs, CD8+ subpopulation in CD45+ SPCs, PD-1+ subpopulation in CD8+CD45+ SPCs, LAG-3+ subpopulation in CD8+CD45+ SPCs, and TIM-3+ subpopulation in CD8+CD45+ SPCs. (D) Graphs with data plots showing the percentage frequency of PD-1+, LAG-3+, and TIM-3+ subpopulations in CD8+CD45+ SPCs. (B, D) *CSTA* induced condition, n = 6; *CSTA* not induced condition, n = 7. Bars represent the mean ± SEM; Student’s t-test was used for statistical analysis.

**Supplementary Figure S10.** Flow cytometry (FCM) analysis of splenocytes (SPCs) for B cells, activation status, and PD-1 immune checkpoint expression. Briefly, doxycycline was supplied in water from day 65 to day 84 to Retro-PAN02-CSTA SC PDAC model mice for *cystatin A* (*CSTA*) induction; the spleens were sampled on day 84 for SPC isolation, followed by FCM analysis. (A) Representative scatter plots of FCM measurements are illustrated: (A) CD45+ subpopulation in SPCs, CD19+ subpopulation in CD45+ SPCs (B cells), CD138+CD45R+ subpopulation in CD19+CD45+ SPCs (plasma cells), CD138-CD45R+ subpopulation in CD19+CD45+ SPCs (non-plasma cells), PD-1+ subpopulation in plasma cells, and PD-1+ subpopulation in non-plasma cells. (B) Graphs with data plots showing the percentage frequency of plasma cells, non-plasma cells, and the PD-1+ subpopulation in plasma cells or in non-plasma cells. *CSTA* induced condition, n = 6; *CSTA* not induced condition, n = 7. Bars represent the mean ± SEM; Student’s t-test was used for statistical analysis.
